# Supplementary material for: HAND1 level controls the specification of multipotent cardiac and extraembryonic progenitors from human pluripotent stem cells
Source: EMBO J. 2025 Mar 31;44(9):2541–65. doi: 10.1038/s44318-025-00409-0 (PMC12048643; doi:10.1038/s44318-025-00409-0)
Supplement: Supplementary file 1 — Appendix [file 44318_2025_409_MOESM1_ESM.pdf]

Appendix

HAND1 level controls the specification of multipotent cardiac and extraembryonic progenitors from human pluripotent stem cells

Adam T Lynch[1,4], Naomi Phillips[1,4], Megan Douglas[1], Marta Dorgnach[1], I-Hsuan Lin[1], Antony D Adamson[1], Zoulfia Darieva[1], Jessica Whittle[1], Neil A Hanley[1,2,3], Nicoletta Bobola[1] and Matthew J Birket*[1]

[1] Faculty of Biology, Medicine and Health, University of Manchester, UK.

[2] College of Medicine & Health, University of Birmingham, Edgbaston, Birmingham, B15 2TT, UK.

[3] University Hospitals Birmingham NHS Foundation Trust, Birmingham, B15 2GW, UK.

[4] These authors contributed equally.

* Corresponding author and lead contact: matthew.birket@manchester.ac.uk

Table of Contents:

Appendix Figure S1 (Page 2-3)
Appendix Figure S2 (Page 4-5)
Appendix Figure S3 (Page 6)
Appendix Figure S4 (Page 7)
Appendix Figure S5 (Page 8)
Appendix Figure S6 (Page 9-10)
Appendix Table S1 (Page 11)
Appendix Table S2 (Page 11)
Appendix Table S3 (Page 11-12)
Appendix Table S4 (Page 12)
Appendix Table S5 (Page 13)

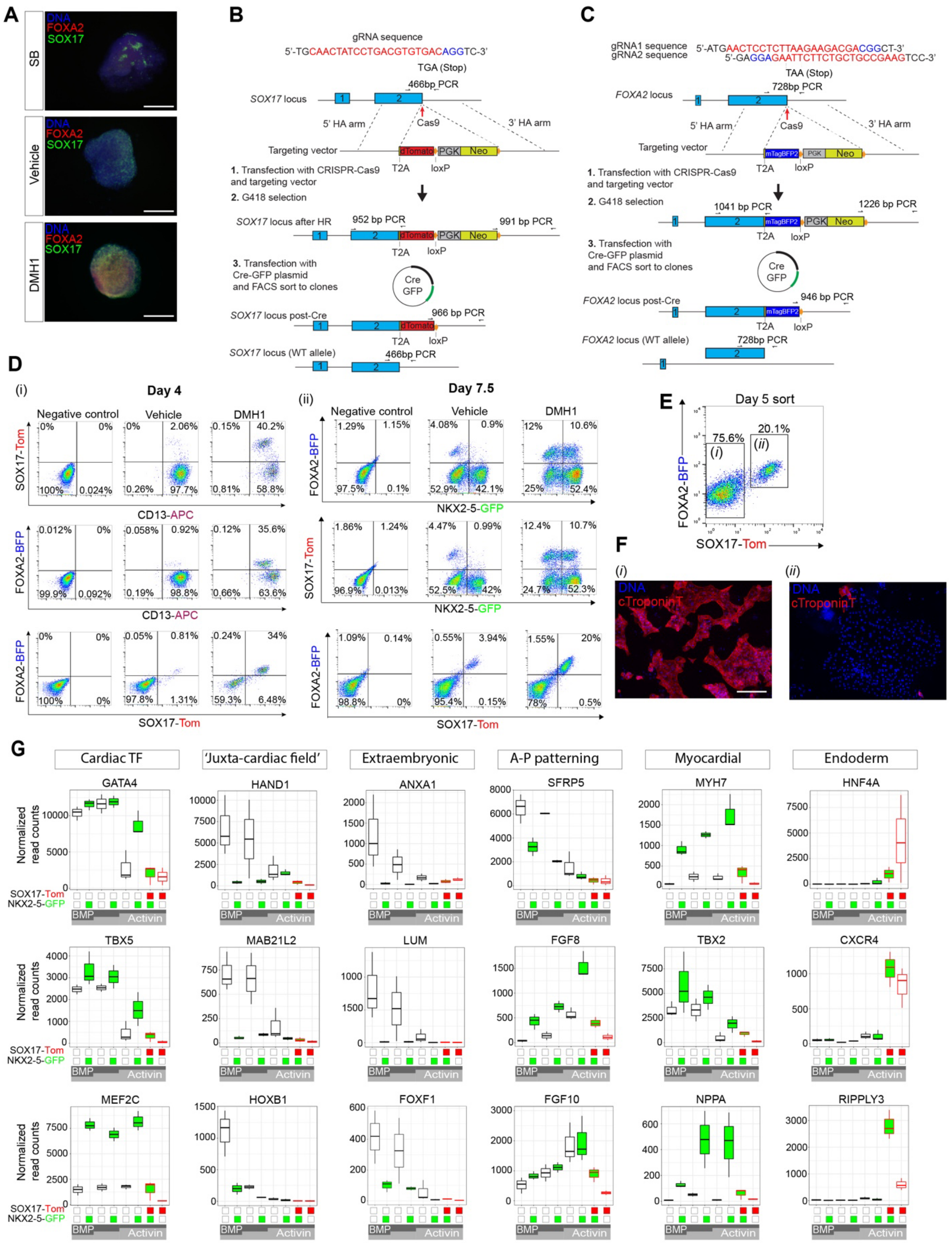

Appendix Fig. S1 │ Resolving cell diversity in mesendoderm differentiation. A, Immunostaining of wholemount day 4 EBs for endoderm markers SOX17 and FOXA2, treated with either SB, vehicle or DMH1 from day 2-3. **B**, Generation of *SOX17-T2A-dTomato* reporter knock-in hESCs by CRISPR-Cas9 gene targeting. A single gRNA was used to target the stop

codon of *SOX17*. After homologous recombination, the integrated selection cassette was removed by Cre recombinase. One allele remained unedited. **C**, Generation of *FOXA2-T2A-mTagBFP* reporter knock-in hESCs by CRISPR-Cas9 gene targeting. Two gRNAs were used to target the stop codon of *FOXA2*. After homologous recombination, the integrated selection cassette was removed by Cre recombinase. One allele remained unedited. **D**, Flow cytometric analyses at day 4 (*i*) and day 7.5 (*ii*) of differentiation of *FOXA2-BFP SOX17-Tom NKX2-5-GFP* triple reporter hESCs in control and DMH1-treated (day 2−3) conditions. The surface marker CD13-APC was included in the analysis at day 4. **E**, Flow cytometric sorting at day 5 by FOXA2-BFP and SOX17-Tomato into double negative (*i*) and double positive (*ii*) populations for differentiation. **F**, Immunostaining of cTroponinT from the sorted populations as in **D**. **G**, Gene expression by RNA-seq of selected markers in the 8 sorted populations based on SOX17-Tom and NKX2-5-GFP at day 7.5. The boxplots follow standard Tukey representations and are coloured by the lineage markers. Scale bars represent 150 μm for **E** and 300 μm for **A**. A-P, anterior-posterior.

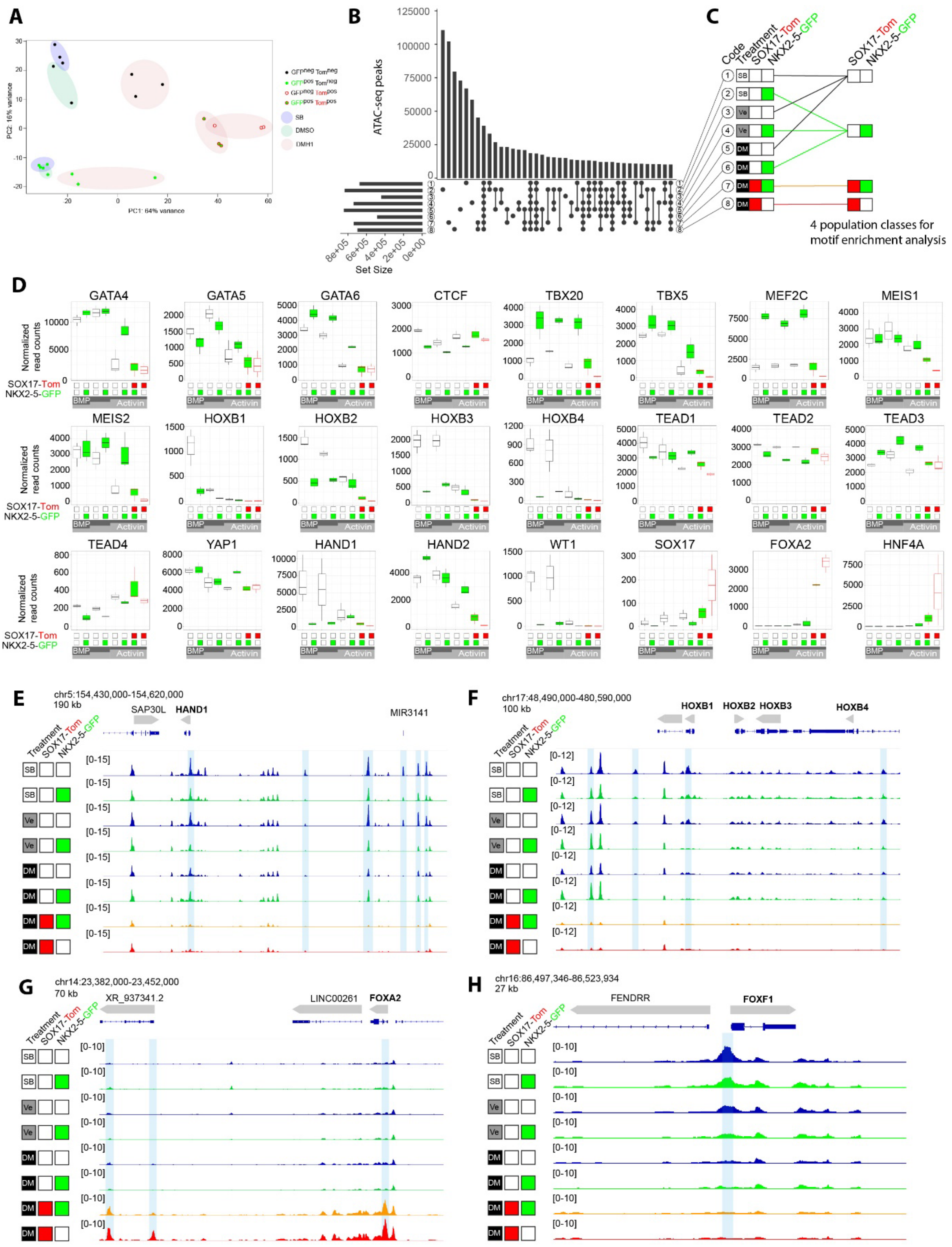

Appendix Fig. S2 │ ATAC-seq and transcription factor motif enrichment analysis with differentiation. **A**, PCA plot of RNA-seq data from the 8 sorted populations sorted based on SOX17-Tomato and NKX2-5-GFP at day 7.5. **B**, Upset plot of ATAC-seq peaks from the 8 sorted

populations sorted based on SOX17-Tomato and NKX2-5-GFP at day 7.5 (number coded based on identity as indicated). Connections indicate common peak sets. **C**, Schematic showing how the 8 samples were divided into 4 population classes for peak set motif enrichment analysis. **D**, Gene expression by RNA-seq of transcription factors relevant to the motif enrichment analysis. Normalized read counts are shown. The boxplots follow standard Tukey representations and are coloured by the lineage markers. **E**–**H**) ATAC-seq tracks coloured according to marker identity around **E**, *HAND1*, **F**, *HOXB1–4*, **G**, *FOXA2* and **H**, *FOXF1* loci (RNA expression of these genes is shown in **D** or **Fig. S1G**. Some differentially accessible regions are highlighted in blue. Genomic coordinates are shown top left.

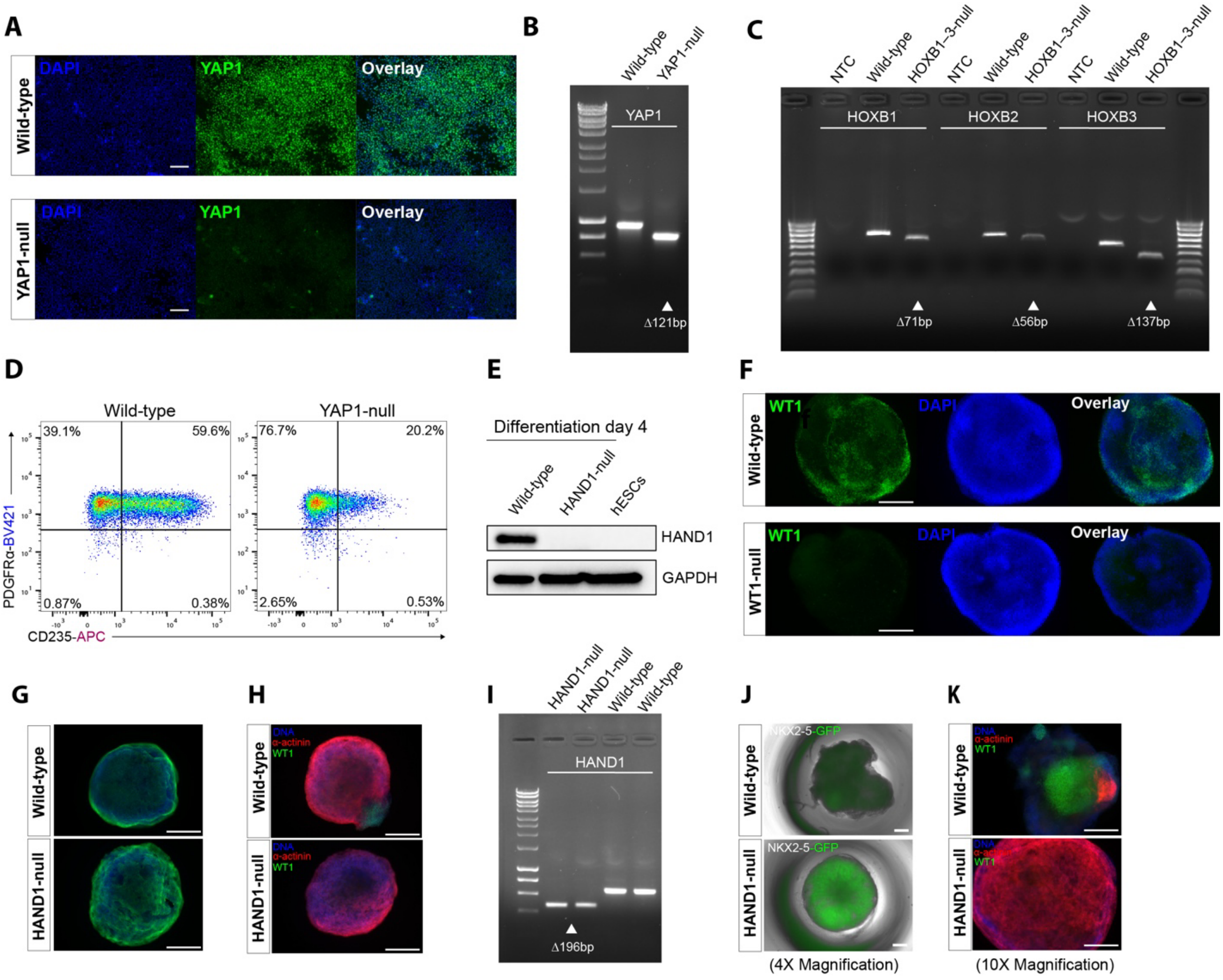

Appendix Fig. S3 │ Validation of hESC gene knockouts by CRISPR-Cas9. **A**, Immunostaining of YAP1 in wild-type and YAP1-null hESCs after CRISPR-Cas9 mutagenesis. **B**, PCR showing expected 121 bp frameshift-causing deletion in *YAP1* in YAP1-null hESCs. **C**, PCR showing expected 71 bp, 56 bp, and 137 bp frameshift-causing deletions in *HOXB1*, *HOXB2*, and *HOXB3* respectively in HOXB1–3-null hESCs. **D**, Flow cytometric analysis of PDGFRα and CD235 in day 4 EBs derived from wild-type and YAP1-null hESCs. **E**, HAND1 western blot in day 4 EBs derived from wild-type and HAND1-null hESCs. Undifferentiated wild-type hESCs are shown as a negative control. **F**, Immunostaining of WT1 in wild-type and WT1-null day 10 EBs. **G**, Immunostaining for cTroponinT in whole mount day 12 EBs. **H,** Immunostaining for α-actinin (red) and WT1 (green) in whole mount day 12 EBs comparing an additional HAND1-null clone of the HES3 hESC line. **I**, PCR showing expected 196 bp frameshift-causing deletion in *HAND1* in HAND1-null in an independent hESC line (UMANe002-A-1) carrying an NKX2-5-GFP reporter. **J,** Typical EBs at day 10 from wild-type and HAND1-null UMANe002-A-1 hESCs. The brightfield image is overlayed by the NKX2-5-GFP signal. **K,** Immunostaining for α-actinin (red) and WT1 (green) in whole mount day 12 EBs from wild-type and HAND1-null UMANe002-A-1 hESCs. Scale bars represent 150 μm in **A**, 300 μm in **F, G, H** and **K,** and 750 μm in **J.** NTC, no template control. EB, embryoid body.

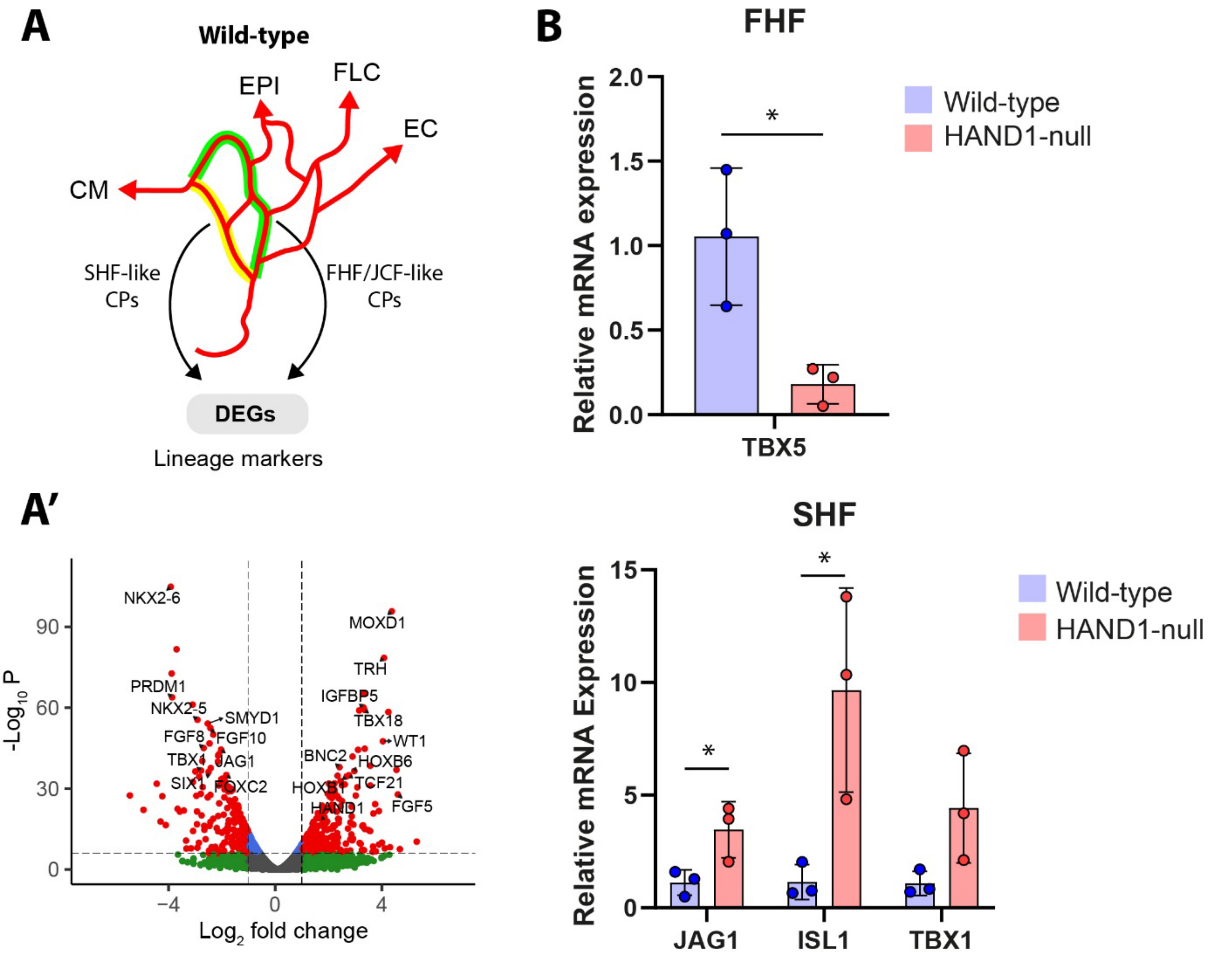

Appendix Fig. S4 │ Gene expression analysis in progenitors. **A**, Differential gene expression of cardiac progenitors extracted from the two lineage trajectories of the wild-type differentiation. **A'**, A volcano plot displaying the results of the DE analysis (FHF/JCF-like vs SHF-like) with some significant genes highlighted. **B**, RT-qPCR of *TBX5* (FHF) and *ISL1, TBX1* and *JAG1* (SHF) in day 5 EBs. Statistical analysis in A' was performed using the "nbTestSH" function (FDR < 0.05) from the sSeq package (Yu et al., 2013). In B, data are represented as mean ± SEM (n=3 independent biological experiments) and statistical analysis was performed using a two-tailed t-test. * denotes $p<0.05$.

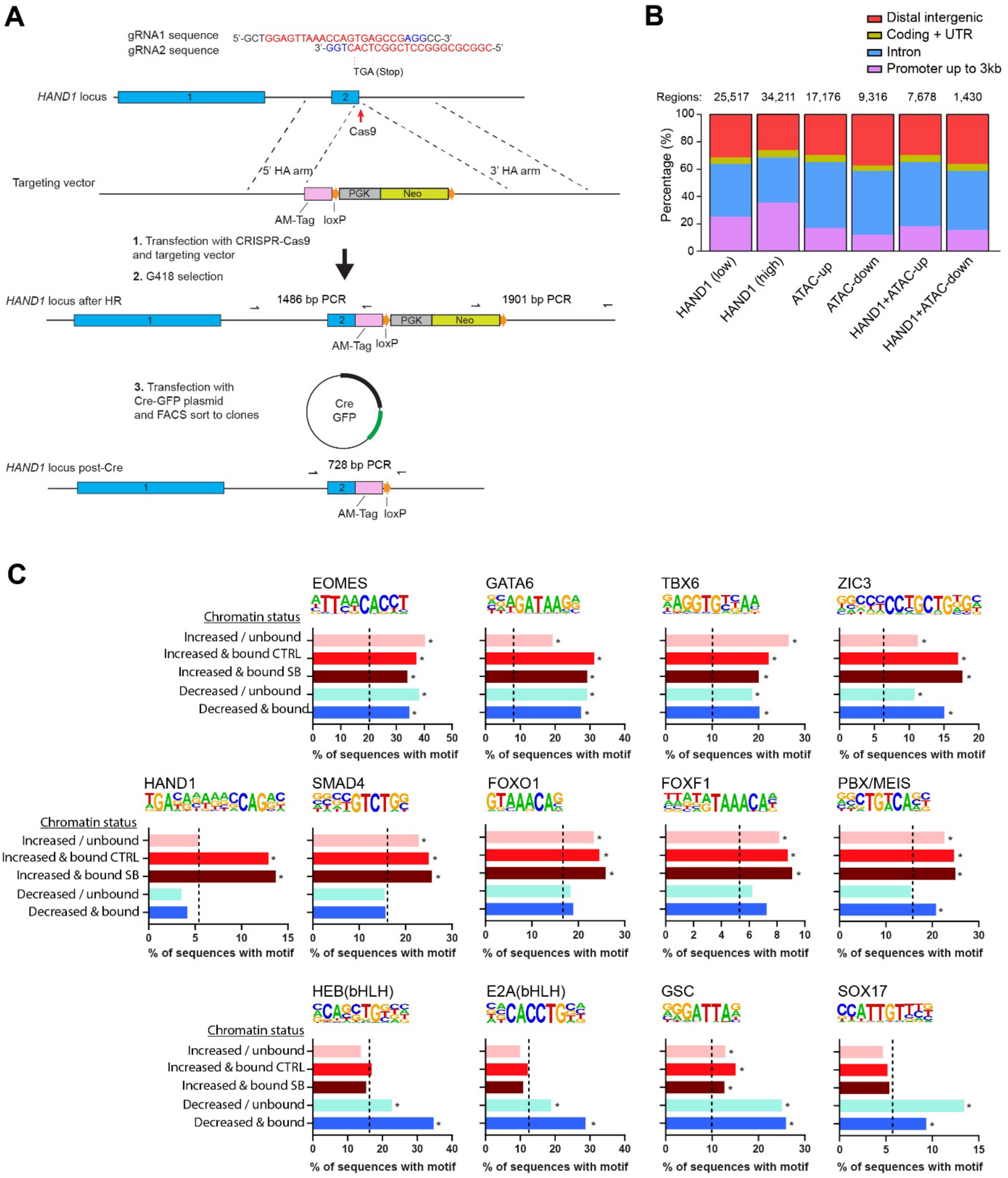

Appendix Fig. S5 │ The impact of HAND1 on chromatin in mesoderm. **A**, Generation of *HAND1-AM-Tag* (Active Motif) knock-in hESCs by CRISPR-Cas9 gene targeting. Two gRNAs were used to target the stop codon of *HAND1*. After homologous recombination, the integrated selection cassette was removed by Cre recombinase. The knock-in was biallelic. **B**, Annotation of ATAC-seq (increased or decreased accessibility by HAND1 and HAND1 ChIP-seq peaks (all peaks or intersected with the ATAC peaks). **C**, Motif enrichment analysis of the regions indicated in **Fig. 5C'**. The percentage of sequences with each motif is shown. * Significance above background (p<1e-6) assessed by binomial statistic. UTR, untranslated region.

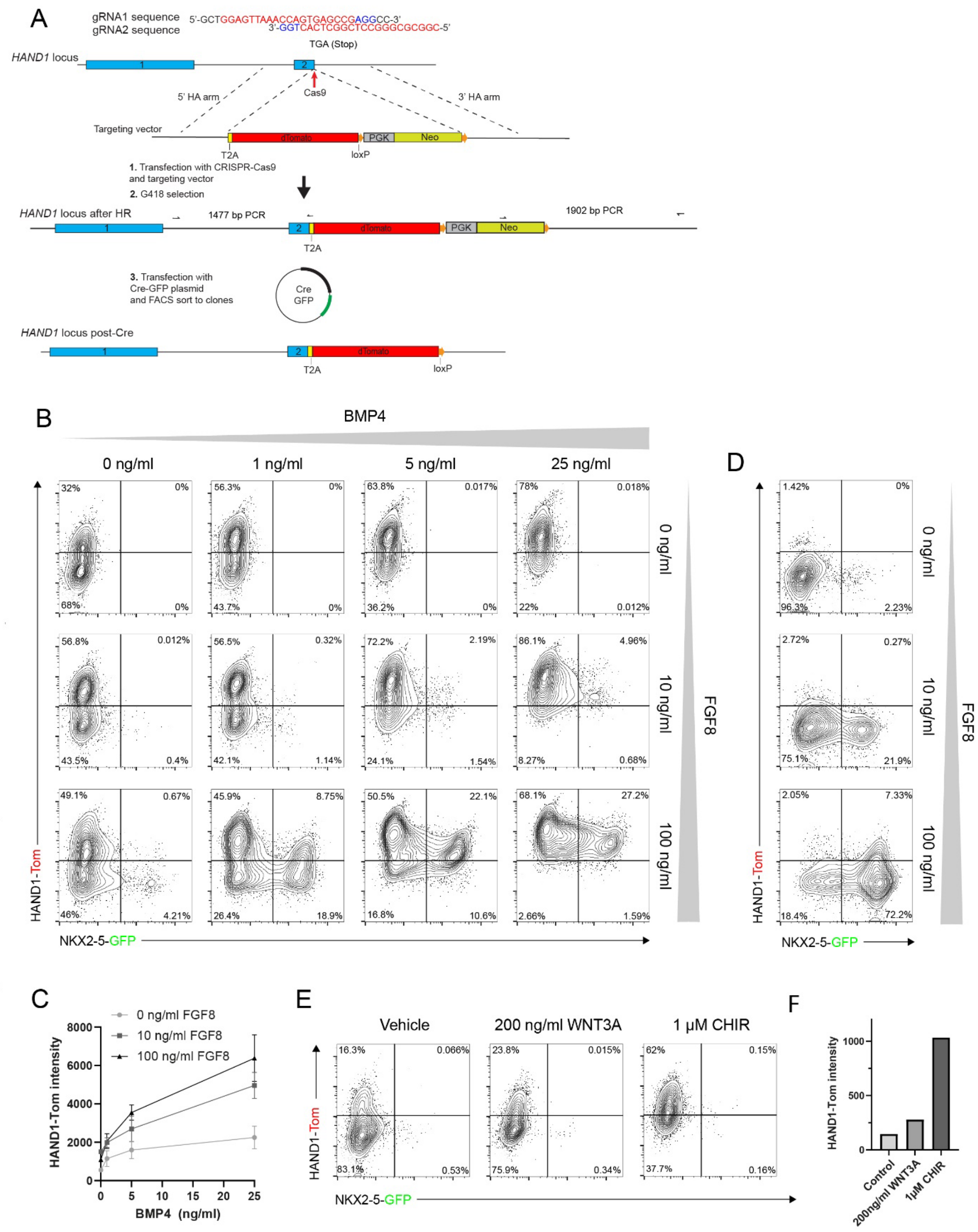

Appendix Fig. S6 │ BMP, FGF and WNT signalling pathways regulate the expression of *HAND1*. A, Generation of *HAND1-T2A-dTomato* reporter knock-in hESCs by CRISPR-Cas9 gene targeting. Two gRNAs were used to target the stop codon of HAND1. After homologous recombination, the integrated selection cassette was removed by Cre recombinase. The knock-in was biallelic. **B**, Flow cytometric analysis of HAND1-Tom and NKX2-5-GFP in progenitor cells derived from SB-treated EBs and cultured in different concentrations of BMP4 and FGF8. **C**,

Median fluorescence intensity values of HAND1-Tomato with FGF8 and BMP4 exposure. **D**, Flow cytometric analysis of HAND1-Tom and NKX2-5-GFP in progenitor cells derived from DMH1-treated EBs and cultured in different concentrations of FGF8. **E**, Flow cytometric analysis of HAND1-Tom and NKX2-5-GFP in progenitor cells derived from SB-treated EBs and cultured in WNT3A or CHIR99021. **F**, Median fluorescence intensity values of HAND1-Tom with WNT3A and CHIR exposure. Data in **C** represent mean ± SD, n = 3 independent biological experiments. EB, embryoid body.

Appendix Table S1. gRNAs sequences for fluorescent reporter knock-ins

gRNA	Sequence 5'-3'
SOX17 KI 1	CAACTATCCTGACGTGTGAC
FOXA2 KI 1	AACTCCTCTTAAGAAGACGA
FOXA2 KI 2	GAAGCCGTCGTCTTCTTAAG
HAND1 KI 1	GGAGTTAAACCAGTGAGCCG
HAND1 KI 2	CGGCGCGGGCCTCGGCTCAC

Appendix Table S2. gRNA sequences for gene knockouts

gRNA	Sequence 5'-3'
HAND1 KO 1	AGCGCGAGGCCGGACCGAAG
HAND1 KO 2	CGCTTGGCGGCCGTCTTGGC
WT1 KO 1	CGCTCCCGCAGGTTACAGCA
WT1 KO 2	GATCCTCATGCTTGAATGAG
HOXB1 KO 1	ACAGAGTGGGTACTCTAAGA
HOXB1 KO 2	ACCGCCTGAGCCGAGCTTGG
HOXB2 KO 1	CACCAGCCTCCGGCAGTCCC
HOXB2 KO 2	CAGTTCCAGCAGCTGCGTGT
HOXB3 KO 1	GAGGGGACAAGAGCCCCCCG
HOXB3 KO 2	TCTTGATCTGCCGCTCGCTG
YAP1 KO 1	GCTGCGAAGGCGGCTGCCCT
YAP1 KO 2	ATCAGATCGTGCACGTCCGC

Appendix Table S3. Antibodies

Antibody	Supplier	Identifier
Polyclonal Goat anti-HAND1	R & D Systems	#AF3168
Monoclonal Mouse anti-PDGFRα-BV421	BD Biosciences	#526799
Monoclonal Mouse anti-CD235a-APC	BD Biosciences	#561775
Monoclonal Mouse anti-ACTN2	Sigma-Aldrich	#A7811
Monoclonal Rabbit anti-WT1	Abcam	#ab89901
Monoclonal Mouse anti-CD31 (PECAM-1)	ThermoFisher	#BMS137
Monoclonal Mouse anti-FOXA2	BD Biosciences	#561580
Monoclonal Mouse anti-CD13-APC	BioLegend	#301705
Polyclonal Rabbit anti-Cardiac Troponin T	Proteintech	#15513-1-AP
Monoclonal Rabbit anti-YAP	Abcam	#ab205270
Polyclonal Goat anti-SOX17	R & D	#AF1924

Mouse AbFlex® AM-Tag (Recombinant)	Active Motif	#91111
Polyclonal Alexa Fluor™-488 Donkey Anti-Rabbit IgG	Invitrogen	#A21206
Polyclonal Alexa Fluor™-555 Donkey Anti-Mouse IgG	Invitrogen	#A31570
Polyclonal Alexa Fluor™-594 Donkey Anti-Mouse IgG	Invitrogen	#A21203
Polyclonal Alexa Fluor™-594 Goat Anti-Rabbit IgG	Invitrogen	#A-11012
Polyclonal Alexa Fluor™-488 Donkey Anti-Goat IgG	Invitrogen	#A-11055
Monoclonal Rabbit anti-SMAD1	Cell Signalling Technology	#6944
Monoclonal Rabbit anti-Phospho-SMAD1/5/9	Cell Signalling Technology	#13820
Monoclonal Rabbit anti-SMAD2	Cell Signalling Technology	#5339
Monoclonal Rabbit anti-Phospho-SMAD2	Cell Signalling Technology	#3108
Anti-rabbit IgG, HRP-linked	Cell Signalling Technology	#7074
Polyclonal HRP-linked Rabbit Anti-Goat IgG	Abcam	#ab97100
Monoclonal HRP-linked Rabbit Anti-GAPDH	Abcam	#ab204481

Appendix Table S4. RT-qPCR Primers

Gene	Forward 5'-3'	Reverse 5'-3'
FOXA2	GCTGGTCGTTTGTTGTGGC	TTCATGCCGTTCATCCCCAG
GUSB	CCACCTAGAATCTGCTGGCTAC	GTGCCCGTAGTCGTGATACCAA
HOXB1	TTCAGCAGAACTCCGGCTAT	CCTCCGTCTCCTTCTGATTG
ISL1	TCCCTATGTGTTGGTTGCGG	GCATTTGATCCCGTACAACCTGA
JAG1	GATCGCCTGCTCAAAGGTCT	GACTGGAAGACCGACACTCG
MESP1	CTCTGTTGGAGACCTGGATG	CCTGCTTGCCTCAAAGTG
MIXL1	GGTACCCCGACATCCACTT	GAGACTTGGCACGCCTGT
RPLPO	CACCATTGAAATCCTGAGTGATGT	TGACCAGCCCAAAGGAGAAG
SOX2	CCCAGCAGACTTCACATGT	CCTCCCATTTCCCTCGTTTT
TBXT	ATCACCAGCCACTGCTTC	GGGTTCCTCCATCATCTCTT
TBX1	GGACGACAACGGCCACATTA	GGTTCTGGTAGGCAGTGACC
TBX5	AAGGCGGATGTTTCCCAGTT	TTGCCCGTCACAGACCATTT

Appendix Table S5. Barcoding primers for ATAC-Seq

Barcoding Primer	Sequence 5'-3'
N701	CAAGCAGAAGACGGCATACGAGATTCGCCTTAGTCTCGTGGGCTCGGAGATGT
N702	CAAGCAGAAGACGGCATACGAGATCTAGTACGGTCTCGTGGGCTCGGAGATGT
N703	CAAGCAGAAGACGGCATACGAGATTTCTGCCTGTCTCGTGGGCTCGGAGATGT
N704	CAAGCAGAAGACGGCATACGAGATGCTCAGGAGTCTCGTGGGCTCGGAGATGT
N501	AATGATACGGCGACCACCGAGATCTACACTAGATCGCTCGTCGGCAGCGTC
N502	AATGATACGGCGACCACCGAGATCTACACCTCTCTATTCGTCGGCAGCGTC
N503	AATGATACGGCGACCACCGAGATCTACACTATCCTCTTCGTCGGCAGCGTC
N504	AATGATACGGCGACCACCGAGATCTACACAGAGTAGATCGTCGGCAGCGTC
