# Supplementary figures and images for: HAND1 level controls the specification of multipotent cardiac and extraembryonic progenitors from human pluripotent stem cells (part 1 of 2)
Source: EMBO J. 2025 Mar 31;44(9):2541–65. doi: 10.1038/s44318-025-00409-0 (PMC12048643; doi:10.1038/s44318-025-00409-0)

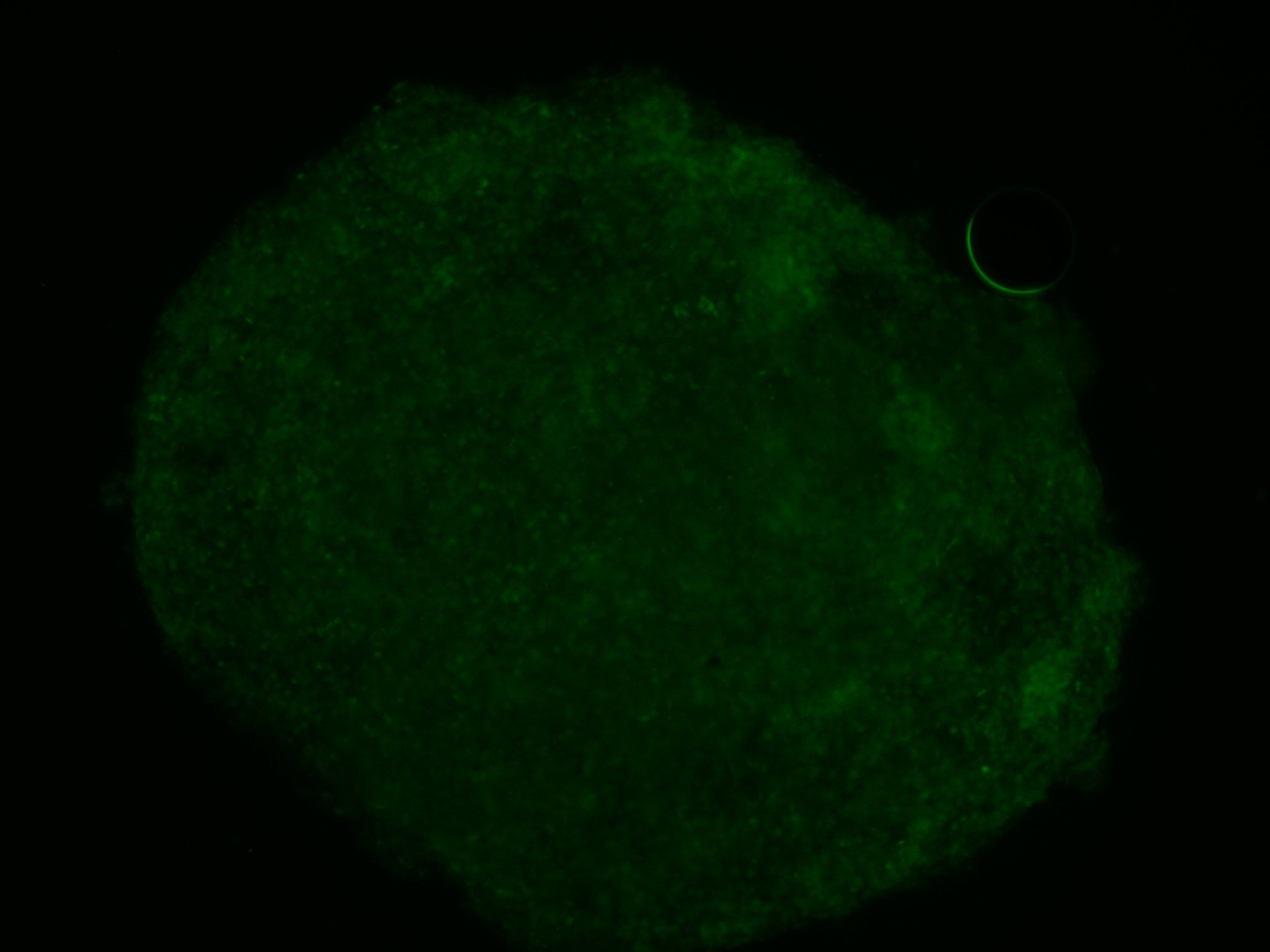

Supplement: Supplementary file 4 — Source data Fig. 1 [file 44318_2025_409_MOESM4_ESM.zip › EMBOJ-2024-118939R-Figure_1_Source_Data-sd/EMBOJ-2024-118939_Fig1H/D12_DMH1_WT1.jpg]

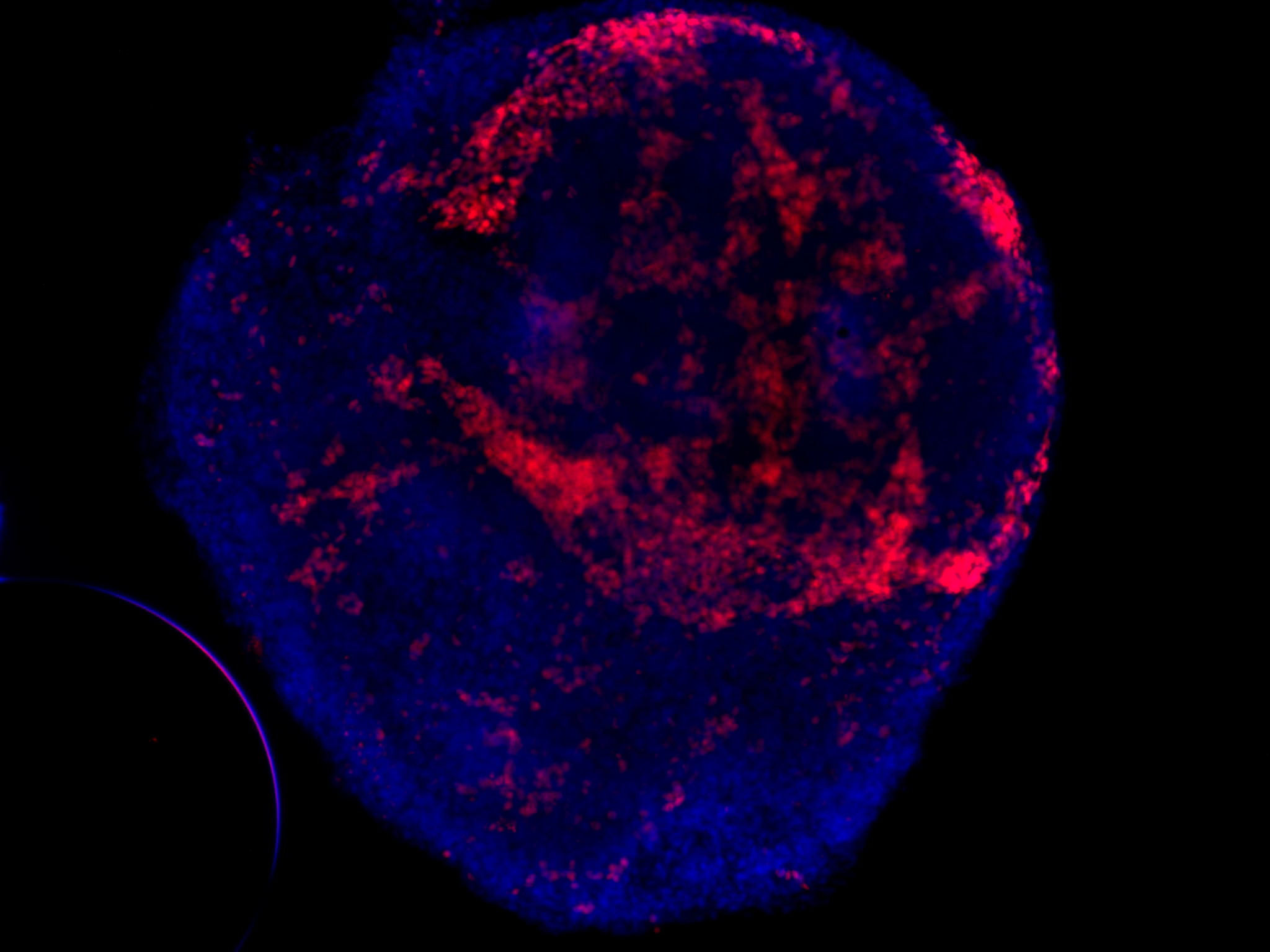

Supplement: Supplementary file 4 — Source data Fig. 1 [file 44318_2025_409_MOESM4_ESM.zip › EMBOJ-2024-118939R-Figure_1_Source_Data-sd/EMBOJ-2024-118939_Fig1H/D4_Veh_overlay.tif]

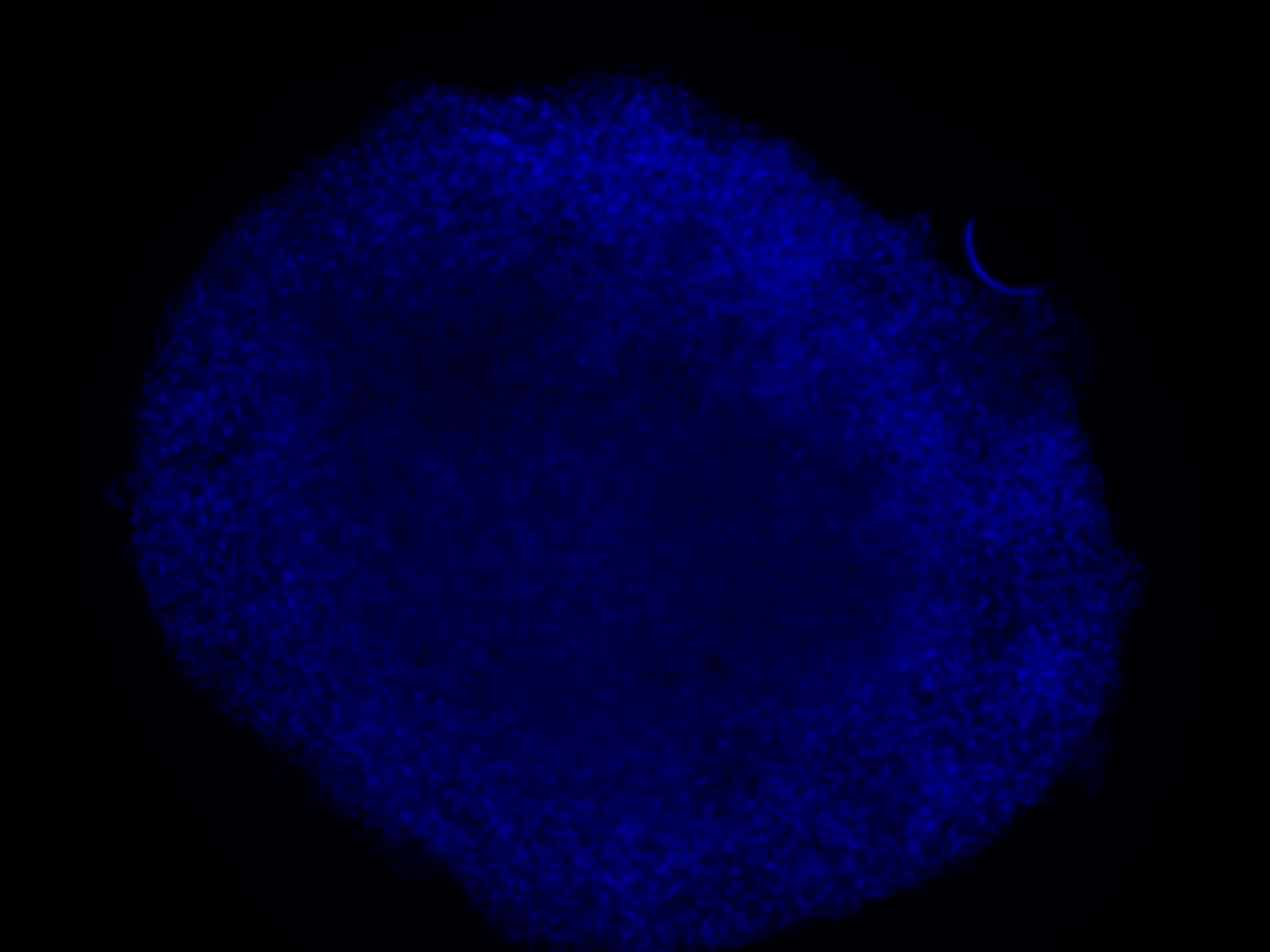

Supplement: Supplementary file 4 — Source data Fig. 1 [file 44318_2025_409_MOESM4_ESM.zip › EMBOJ-2024-118939R-Figure_1_Source_Data-sd/EMBOJ-2024-118939_Fig1H/D12_DMH1_DNA.jpg]

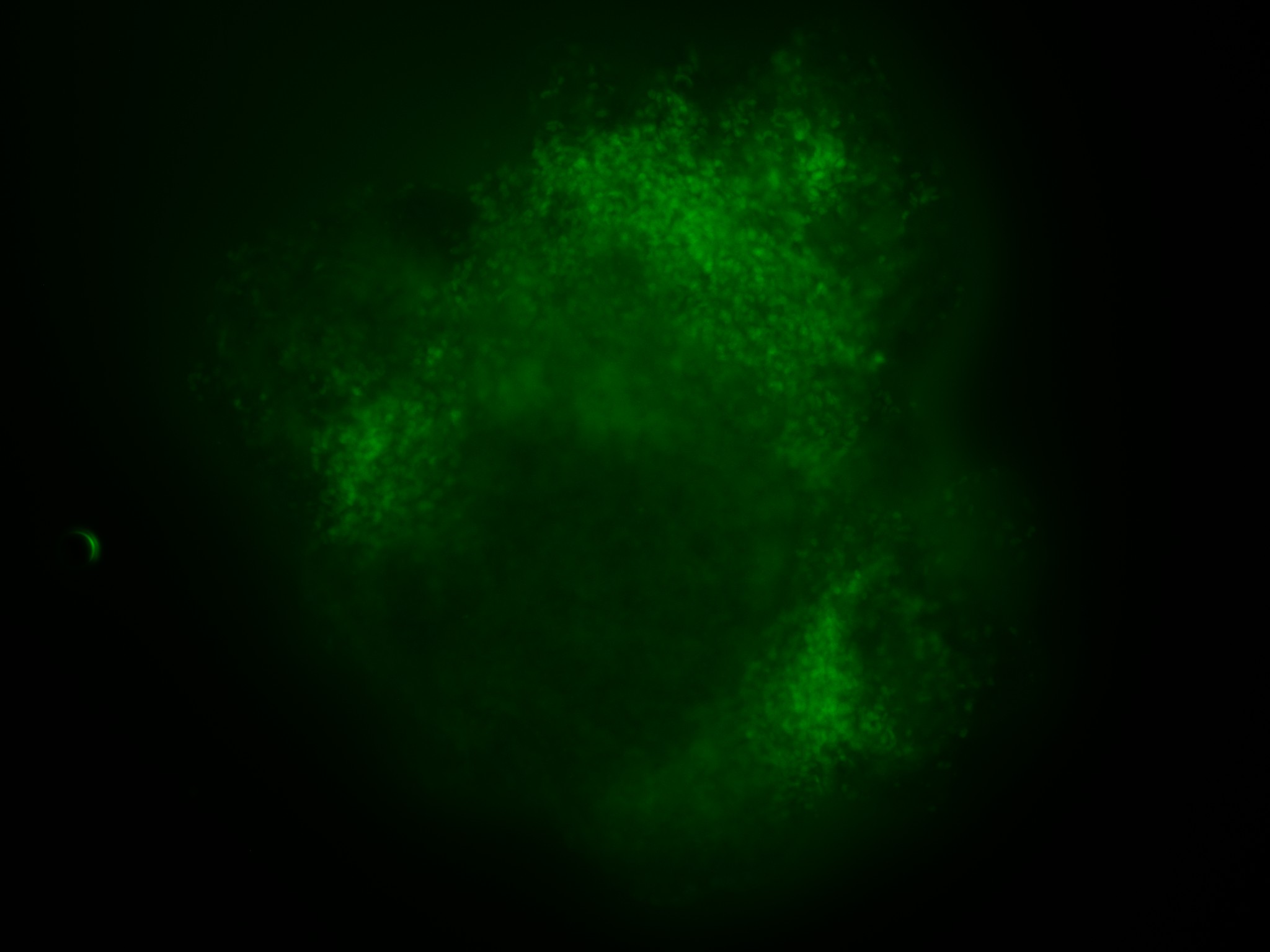

Supplement: Supplementary file 4 — Source data Fig. 1 [file 44318_2025_409_MOESM4_ESM.zip › EMBOJ-2024-118939R-Figure_1_Source_Data-sd/EMBOJ-2024-118939_Fig1H/D12_SB_WT1.jpg]

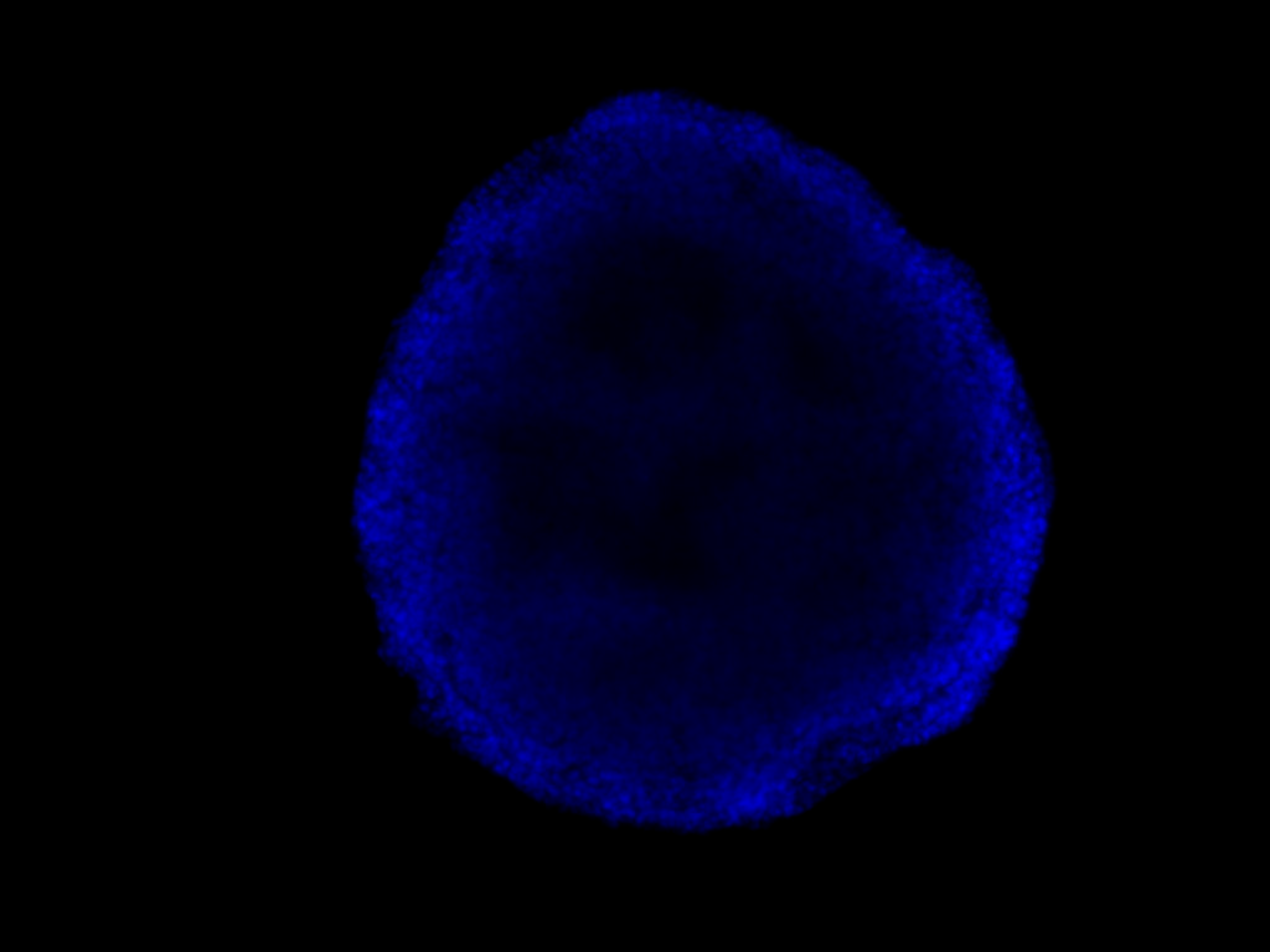

Supplement: Supplementary file 4 — Source data Fig. 1 [file 44318_2025_409_MOESM4_ESM.zip › EMBOJ-2024-118939R-Figure_1_Source_Data-sd/EMBOJ-2024-118939_Fig1H/D4_DMH1_DNA.tif]

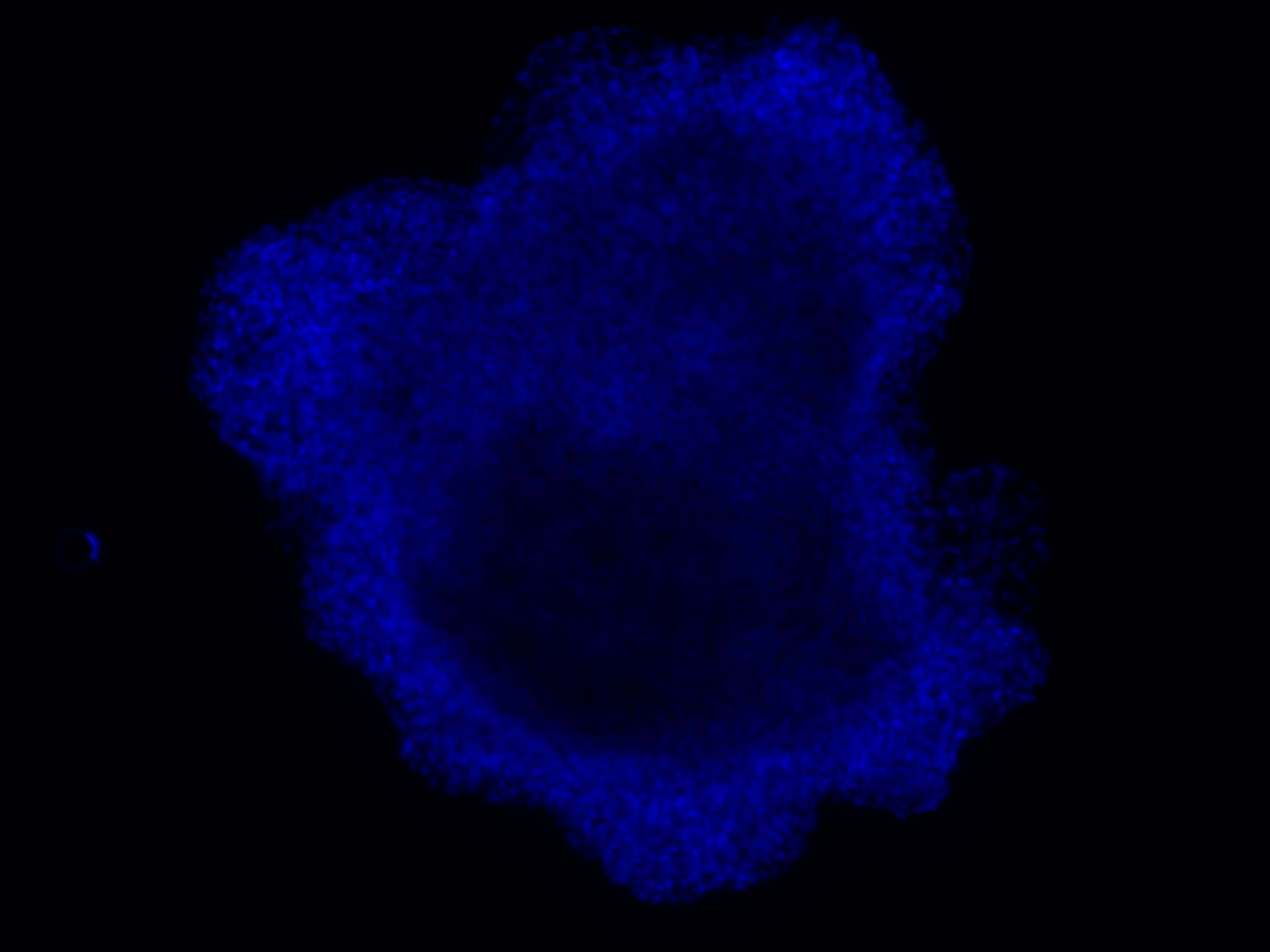

Supplement: Supplementary file 4 — Source data Fig. 1 [file 44318_2025_409_MOESM4_ESM.zip › EMBOJ-2024-118939R-Figure_1_Source_Data-sd/EMBOJ-2024-118939_Fig1H/D12_SB_DNA.jpg]

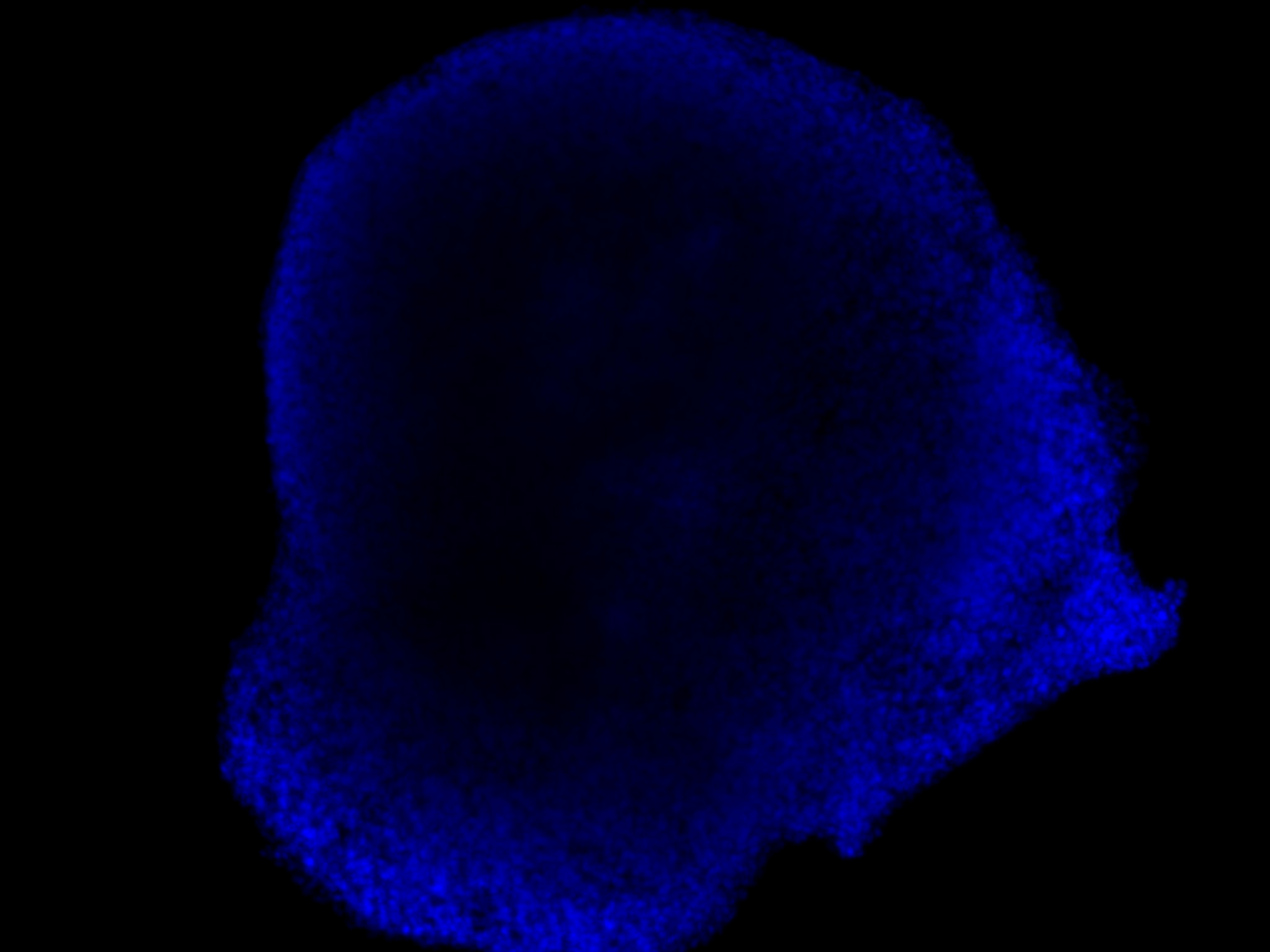

Supplement: Supplementary file 4 — Source data Fig. 1 [file 44318_2025_409_MOESM4_ESM.zip › EMBOJ-2024-118939R-Figure_1_Source_Data-sd/EMBOJ-2024-118939_Fig1H/D4_SB_DNA.tif]

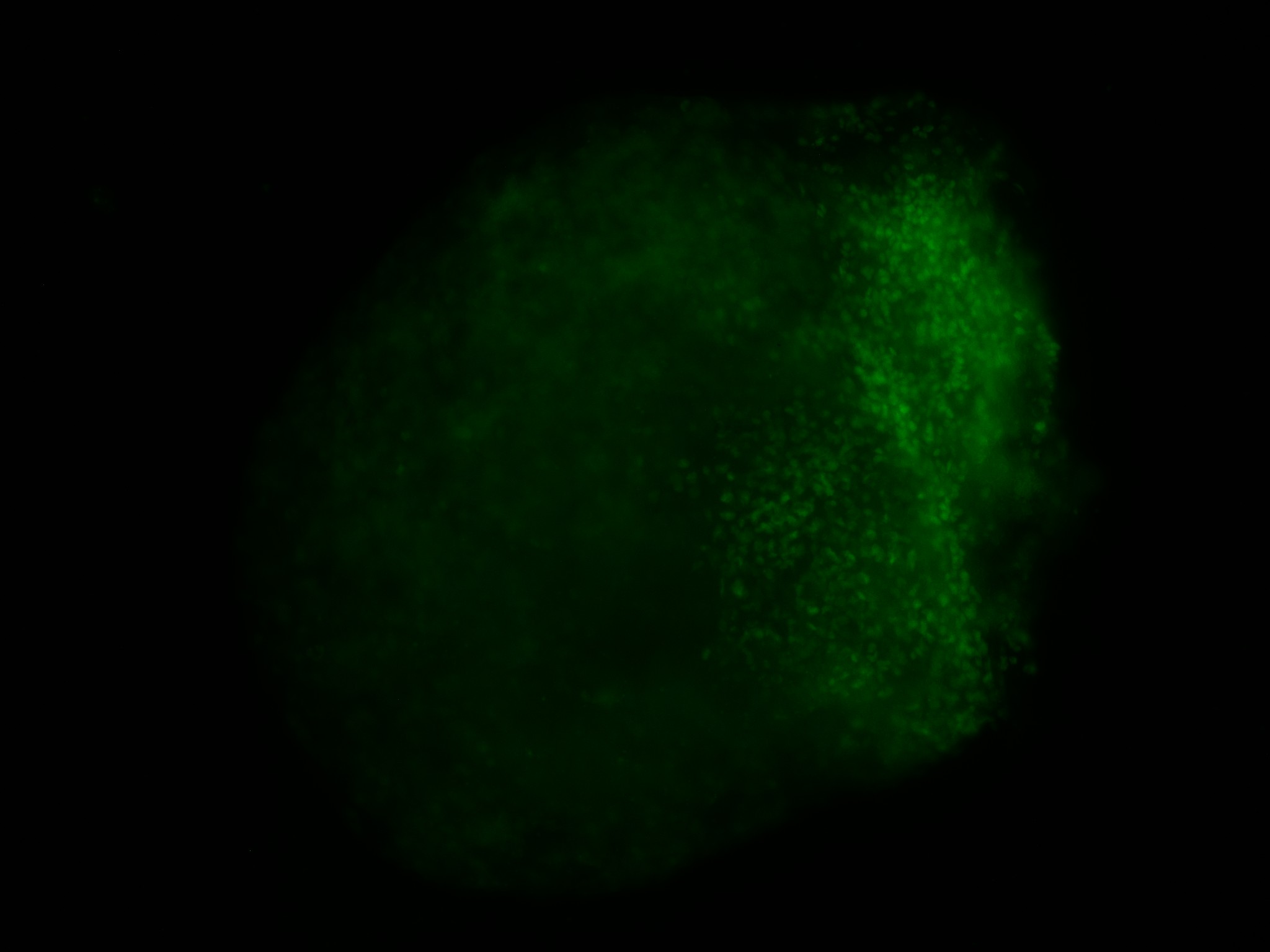

Supplement: Supplementary file 4 — Source data Fig. 1 [file 44318_2025_409_MOESM4_ESM.zip › EMBOJ-2024-118939R-Figure_1_Source_Data-sd/EMBOJ-2024-118939_Fig1H/D12 DMSO_WT1.jpg]

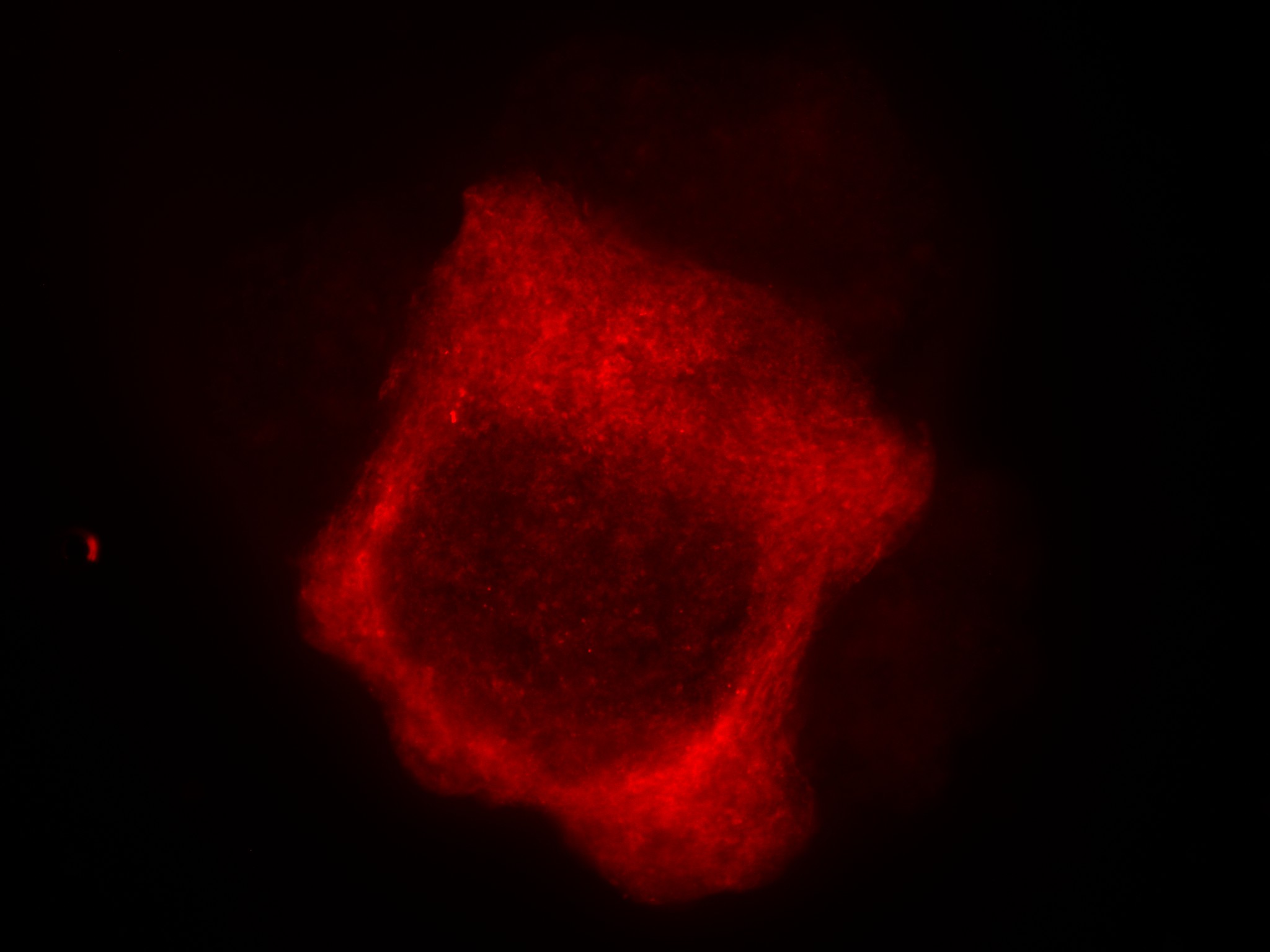

Supplement: Supplementary file 4 — Source data Fig. 1 [file 44318_2025_409_MOESM4_ESM.zip › EMBOJ-2024-118939R-Figure_1_Source_Data-sd/EMBOJ-2024-118939_Fig1H/D12_SB_ACTN2.jpg]

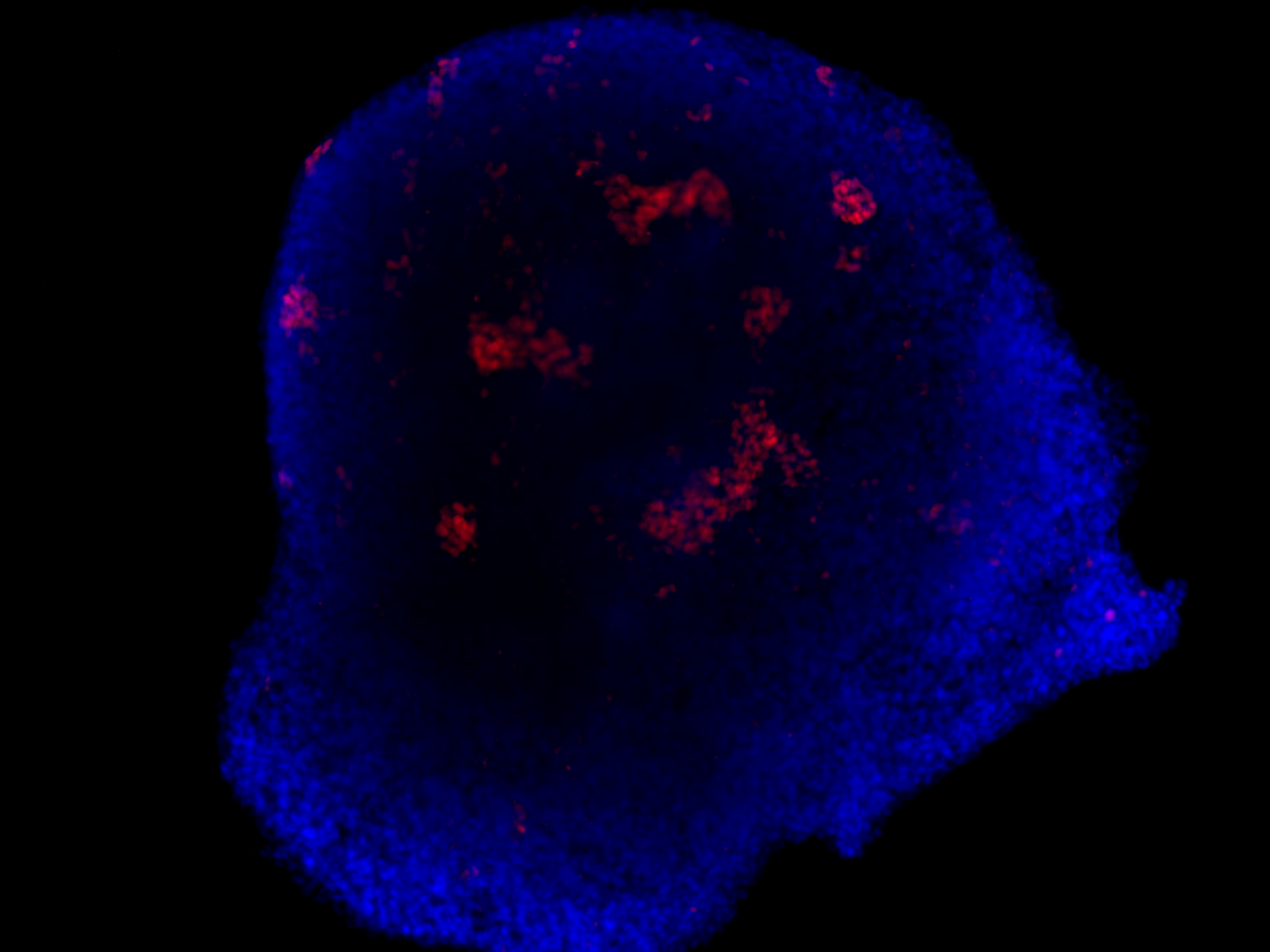

Supplement: Supplementary file 4 — Source data Fig. 1 [file 44318_2025_409_MOESM4_ESM.zip › EMBOJ-2024-118939R-Figure_1_Source_Data-sd/EMBOJ-2024-118939_Fig1H/D4_SB_overlay.tif]

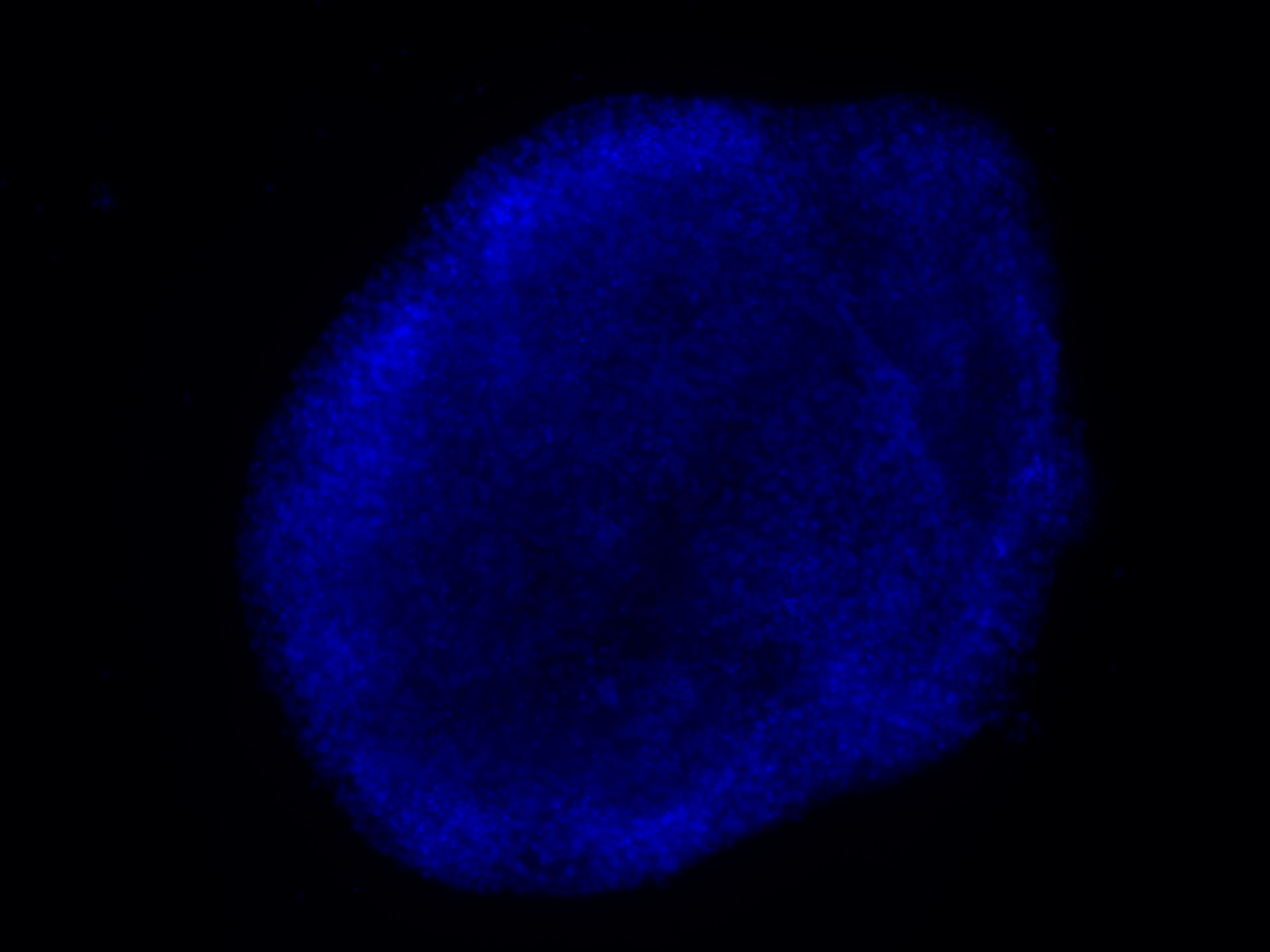

Supplement: Supplementary file 4 — Source data Fig. 1 [file 44318_2025_409_MOESM4_ESM.zip › EMBOJ-2024-118939R-Figure_1_Source_Data-sd/EMBOJ-2024-118939_Fig1H/D12_Veh_DNA.jpg]

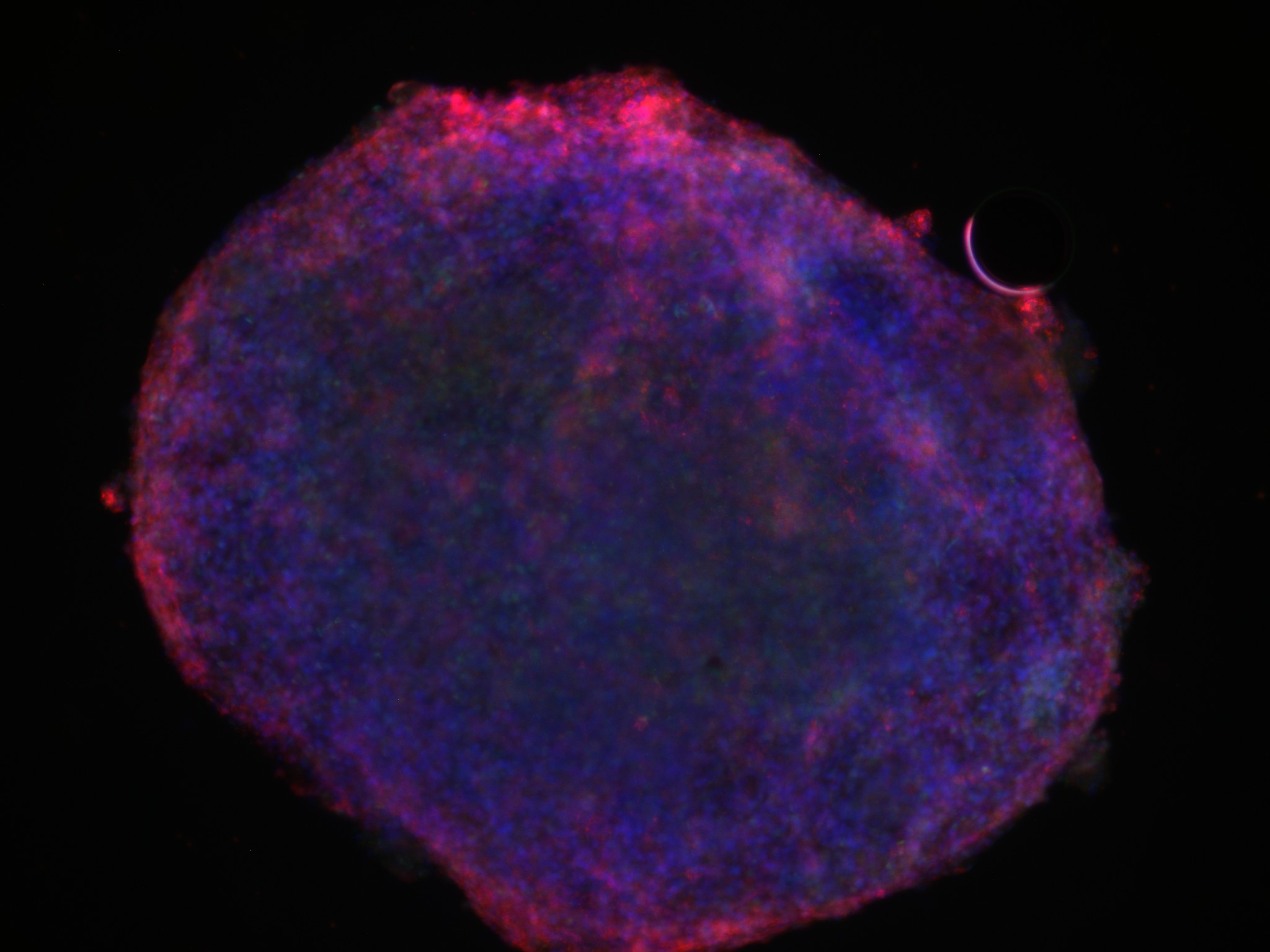

Supplement: Supplementary file 4 — Source data Fig. 1 [file 44318_2025_409_MOESM4_ESM.zip › EMBOJ-2024-118939R-Figure_1_Source_Data-sd/EMBOJ-2024-118939_Fig1H/D12_DMH1_overlay.jpg]

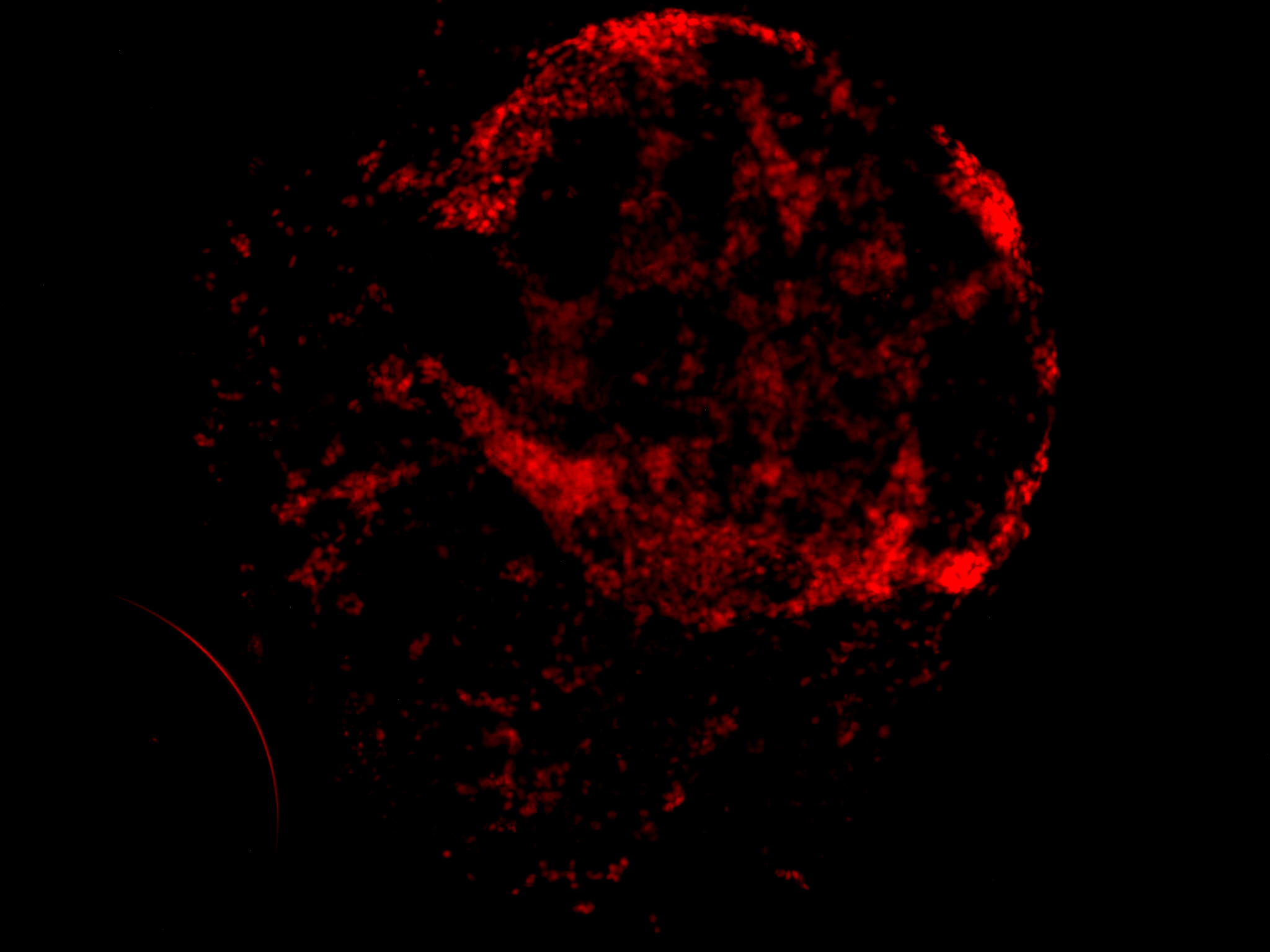

Supplement: Supplementary file 4 — Source data Fig. 1 [file 44318_2025_409_MOESM4_ESM.zip › EMBOJ-2024-118939R-Figure_1_Source_Data-sd/EMBOJ-2024-118939_Fig1H/D4_Veh_FOXA2.tif]

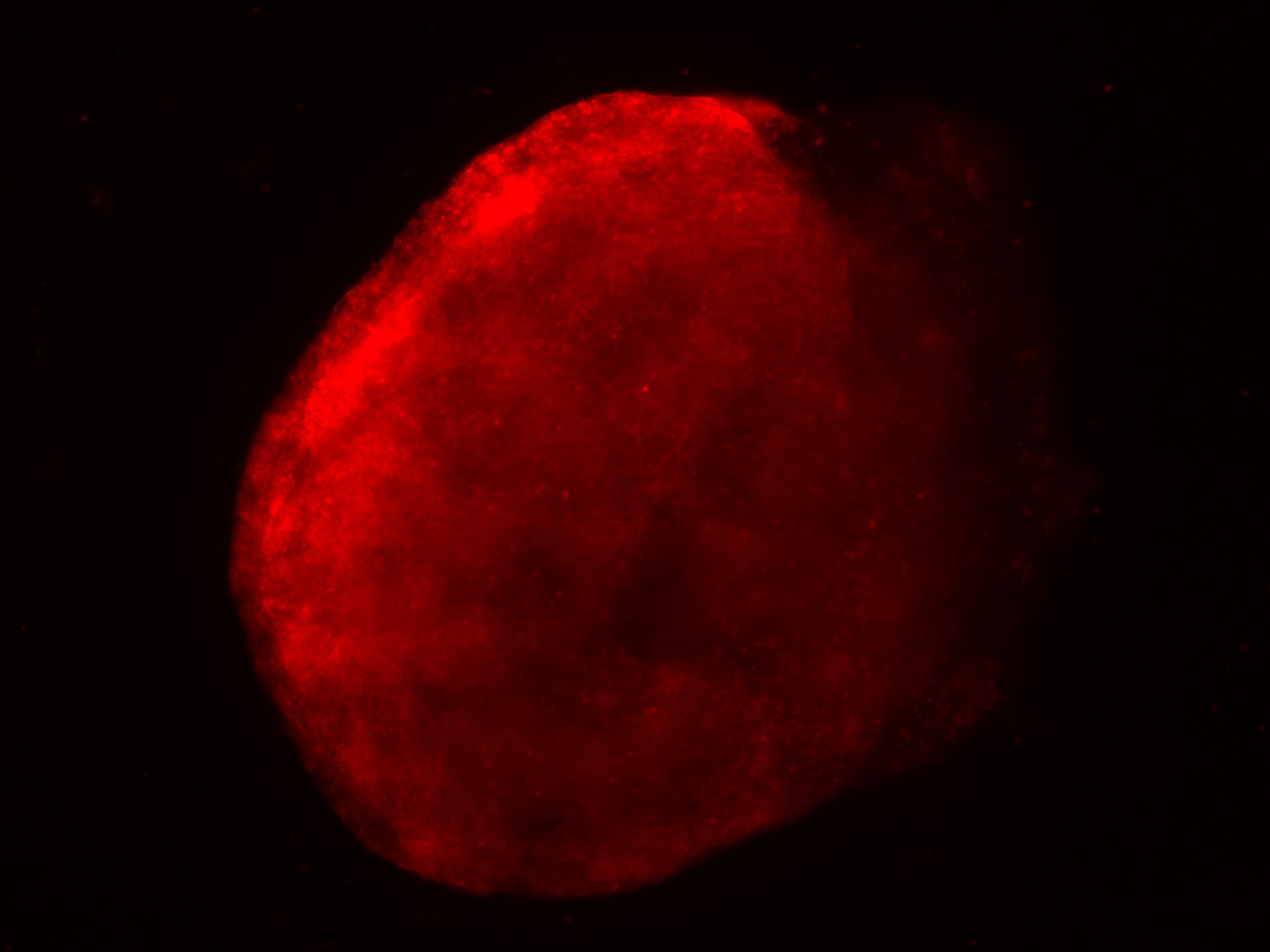

Supplement: Supplementary file 4 — Source data Fig. 1 [file 44318_2025_409_MOESM4_ESM.zip › EMBOJ-2024-118939R-Figure_1_Source_Data-sd/EMBOJ-2024-118939_Fig1H/D12_Veh_ACTN2.jpg]

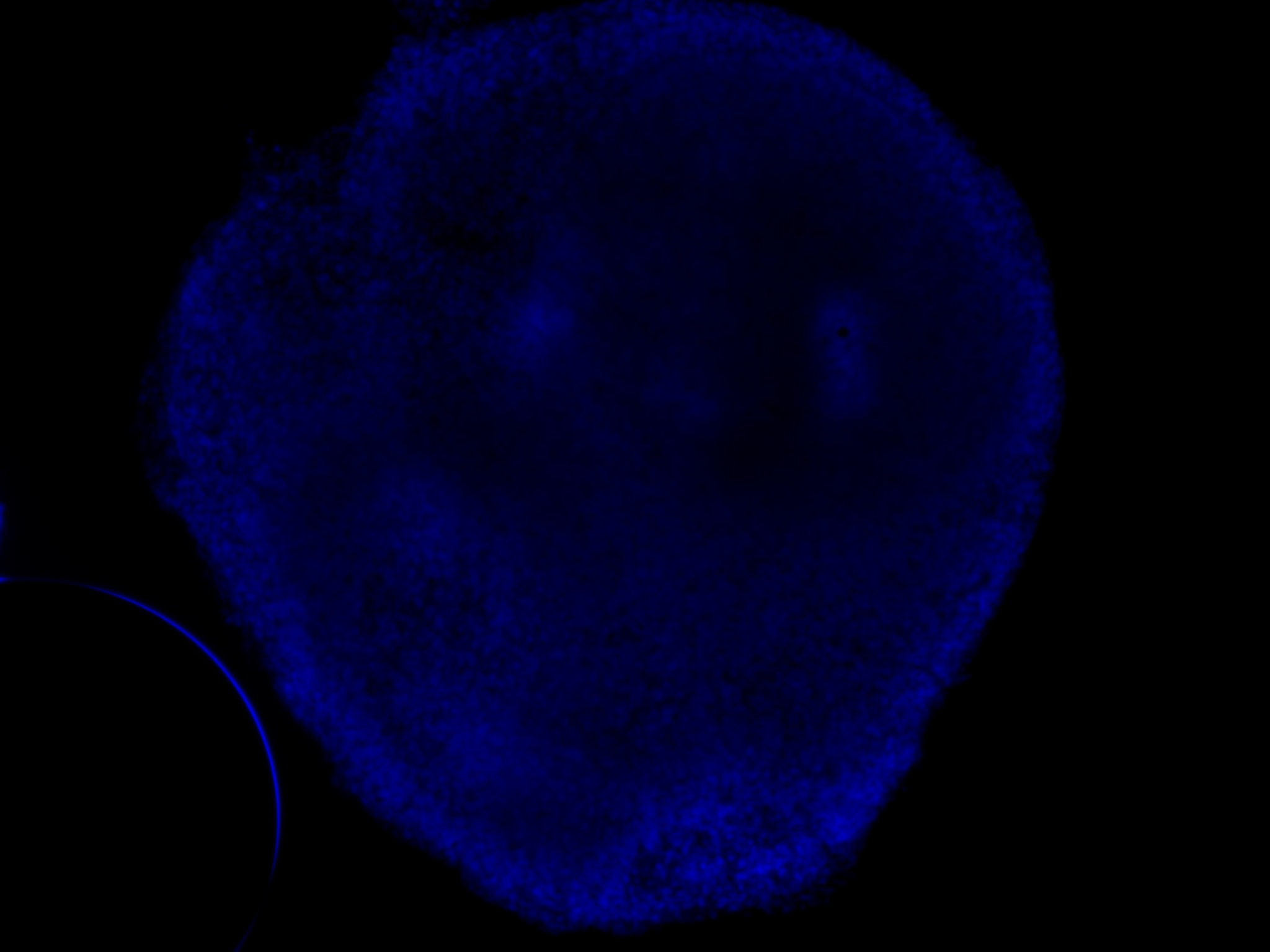

Supplement: Supplementary file 4 — Source data Fig. 1 [file 44318_2025_409_MOESM4_ESM.zip › EMBOJ-2024-118939R-Figure_1_Source_Data-sd/EMBOJ-2024-118939_Fig1H/D4_Veh_DNA.tif]

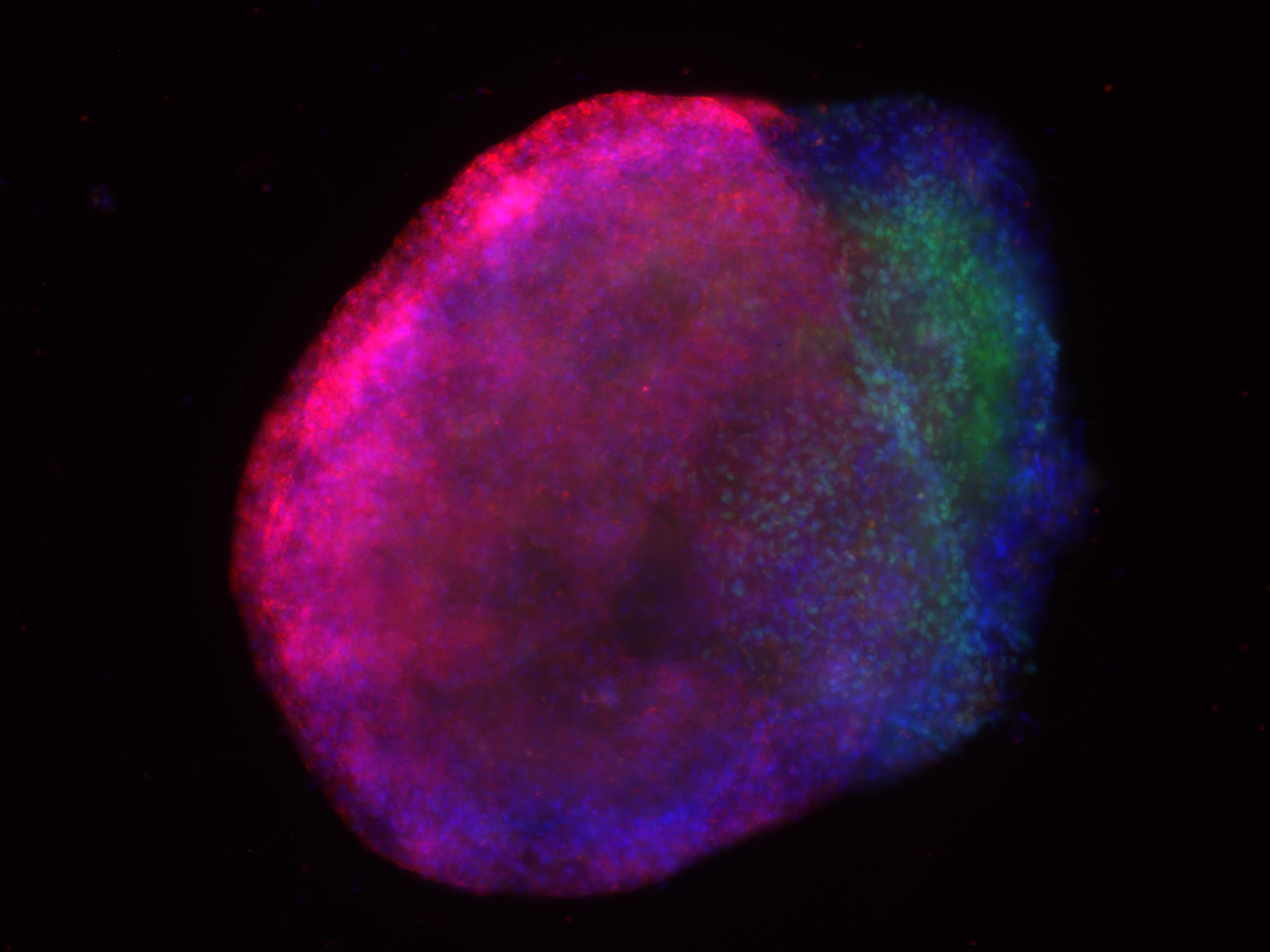

Supplement: Supplementary file 4 — Source data Fig. 1 [file 44318_2025_409_MOESM4_ESM.zip › EMBOJ-2024-118939R-Figure_1_Source_Data-sd/EMBOJ-2024-118939_Fig1H/D12 DMSO_Merge.jpg]

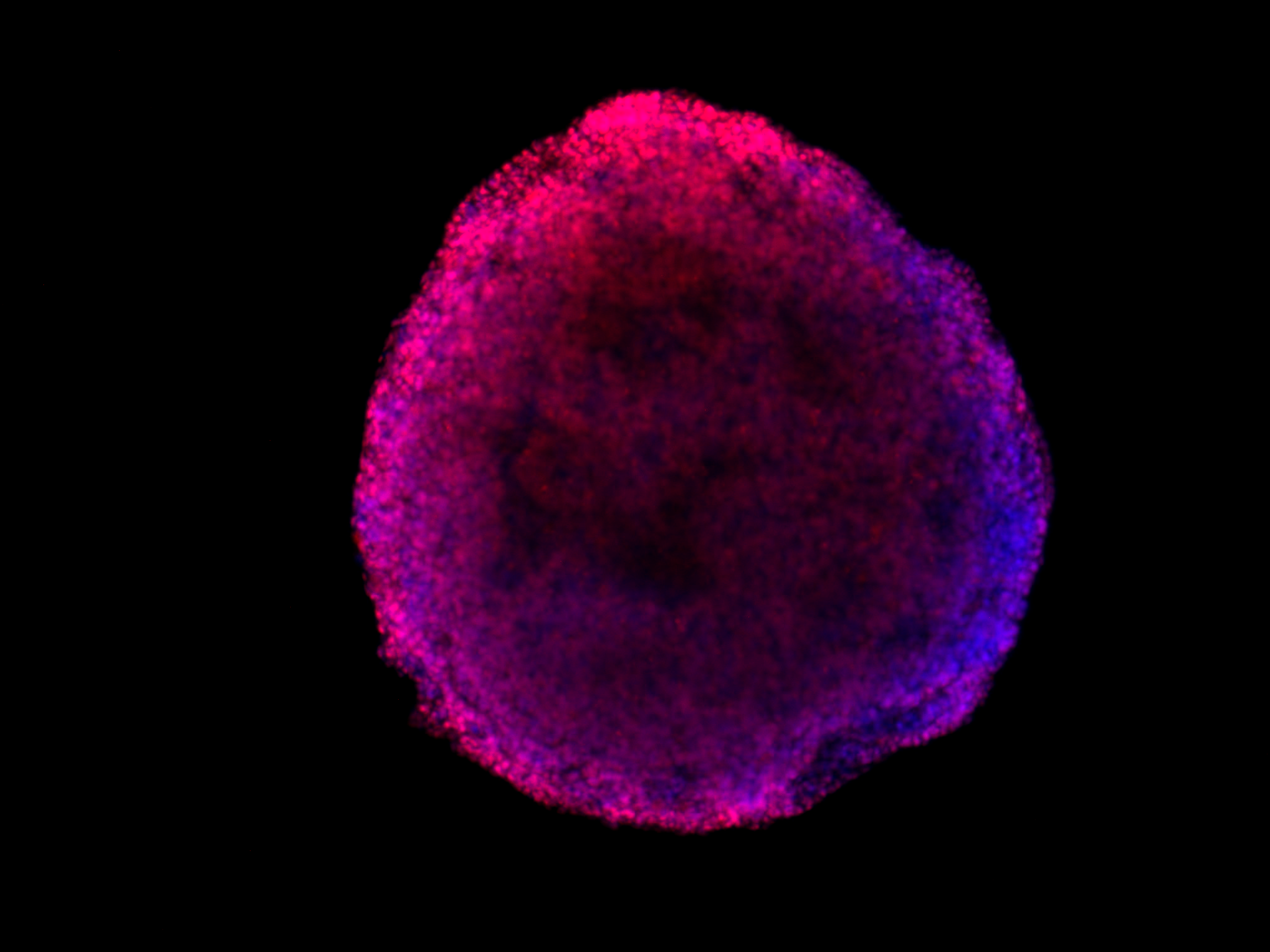

Supplement: Supplementary file 4 — Source data Fig. 1 [file 44318_2025_409_MOESM4_ESM.zip › EMBOJ-2024-118939R-Figure_1_Source_Data-sd/EMBOJ-2024-118939_Fig1H/D4_DMH1_overlay.tif]

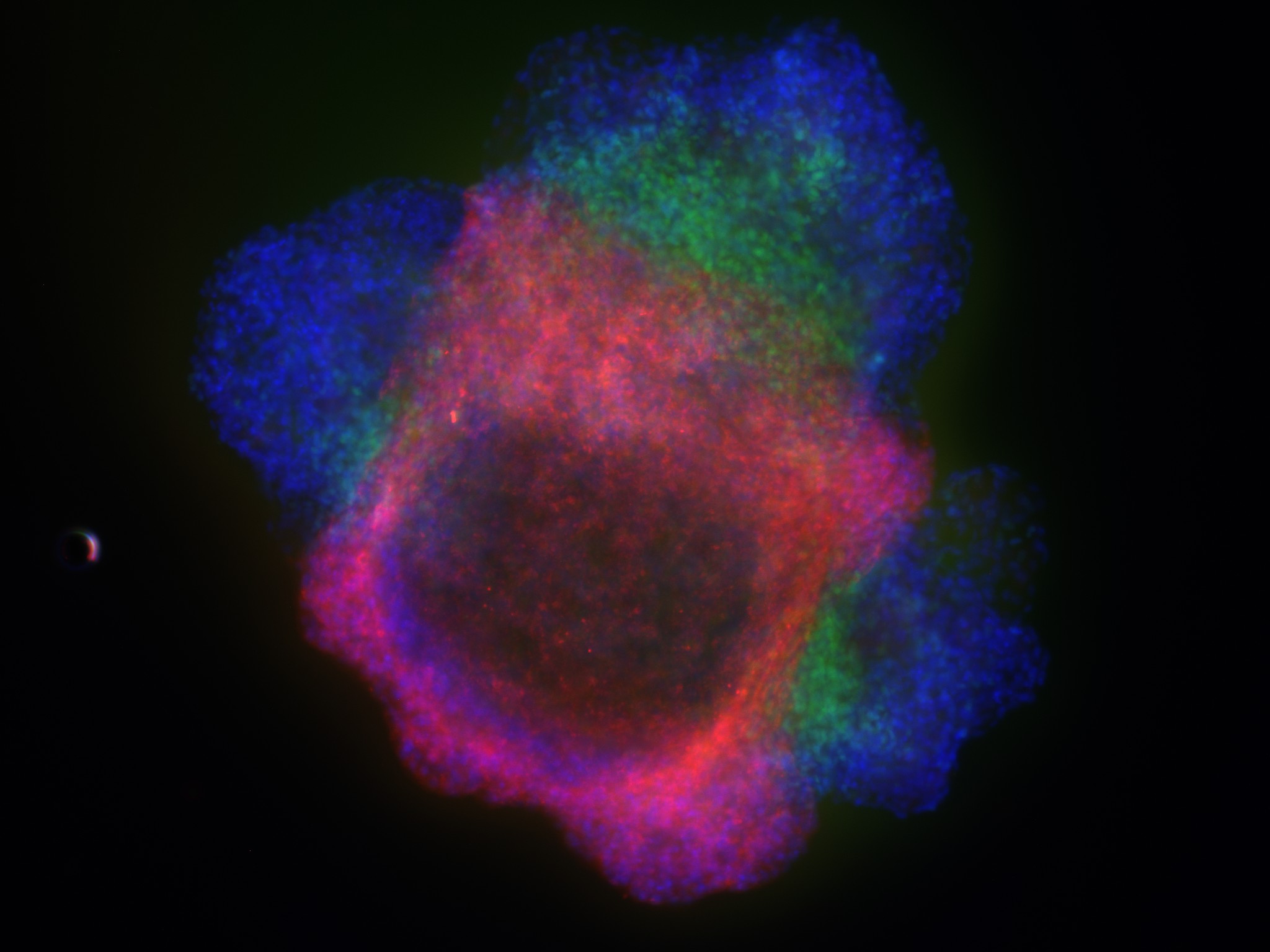

Supplement: Supplementary file 4 — Source data Fig. 1 [file 44318_2025_409_MOESM4_ESM.zip › EMBOJ-2024-118939R-Figure_1_Source_Data-sd/EMBOJ-2024-118939_Fig1H/D12_SB_overlay.jpg]

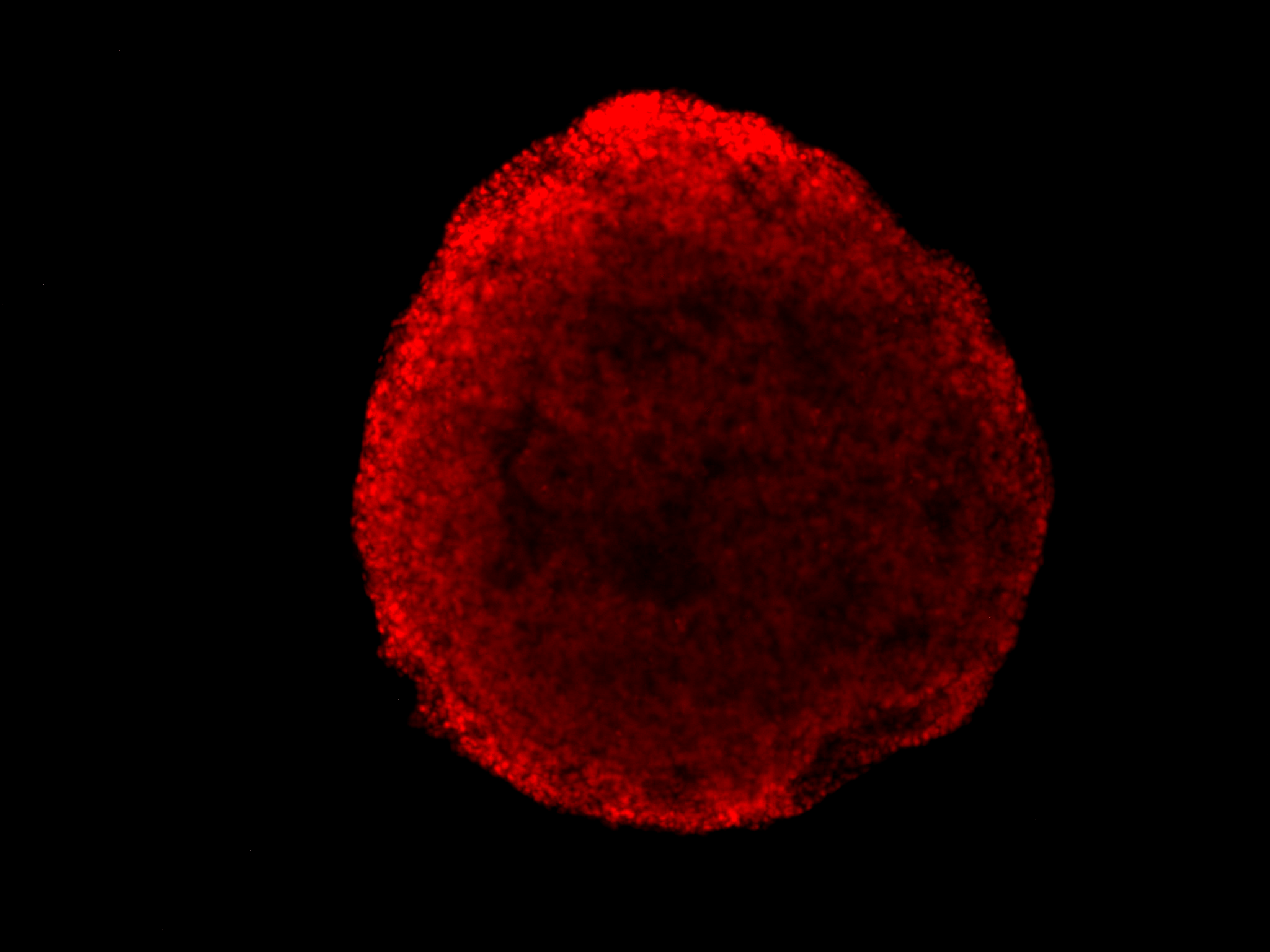

Supplement: Supplementary file 4 — Source data Fig. 1 [file 44318_2025_409_MOESM4_ESM.zip › EMBOJ-2024-118939R-Figure_1_Source_Data-sd/EMBOJ-2024-118939_Fig1H/D4_DMH1_FOXA2.tif]

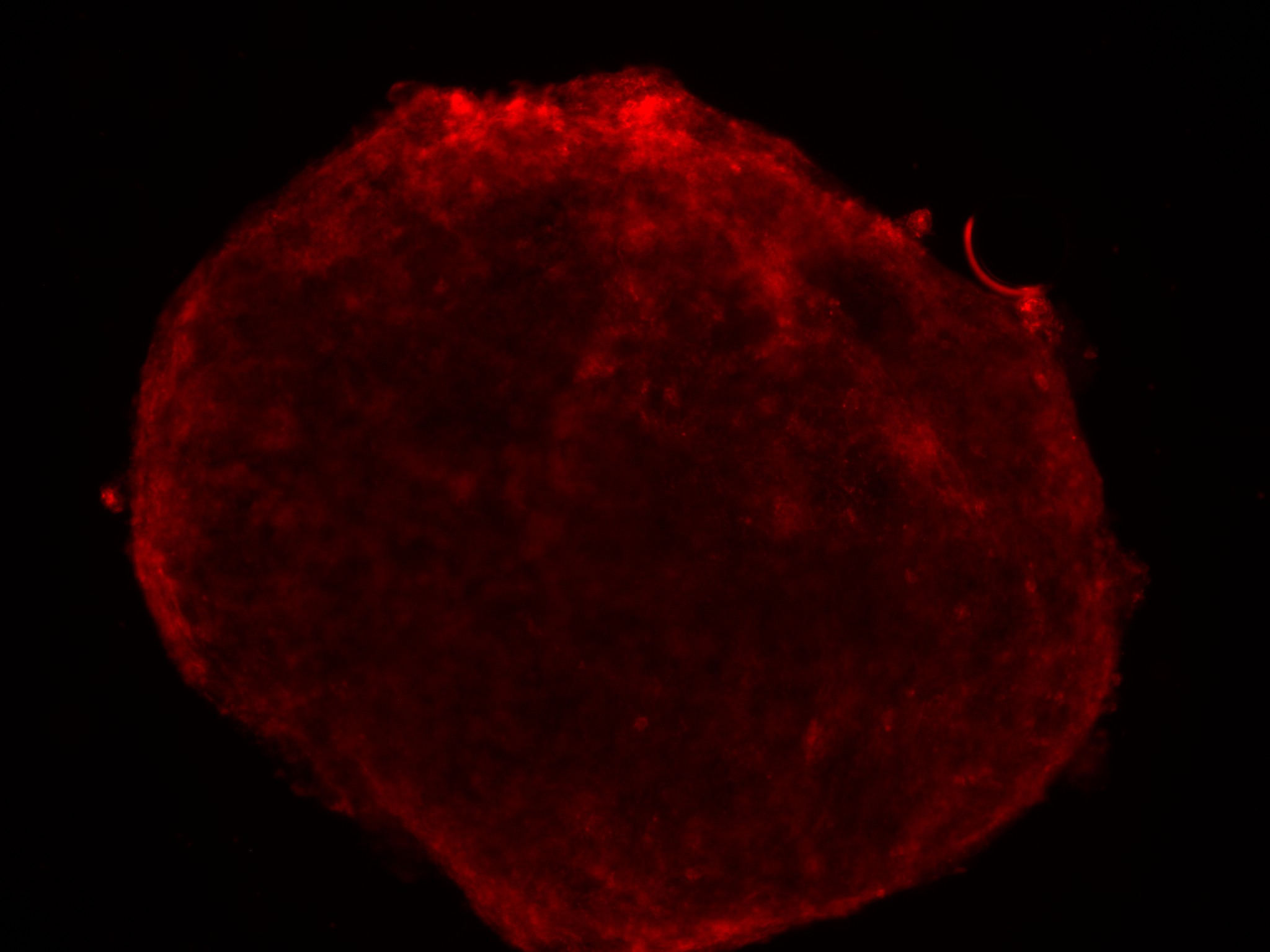

Supplement: Supplementary file 4 — Source data Fig. 1 [file 44318_2025_409_MOESM4_ESM.zip › EMBOJ-2024-118939R-Figure_1_Source_Data-sd/EMBOJ-2024-118939_Fig1H/D12_DMH1_ACTN2.jpg]

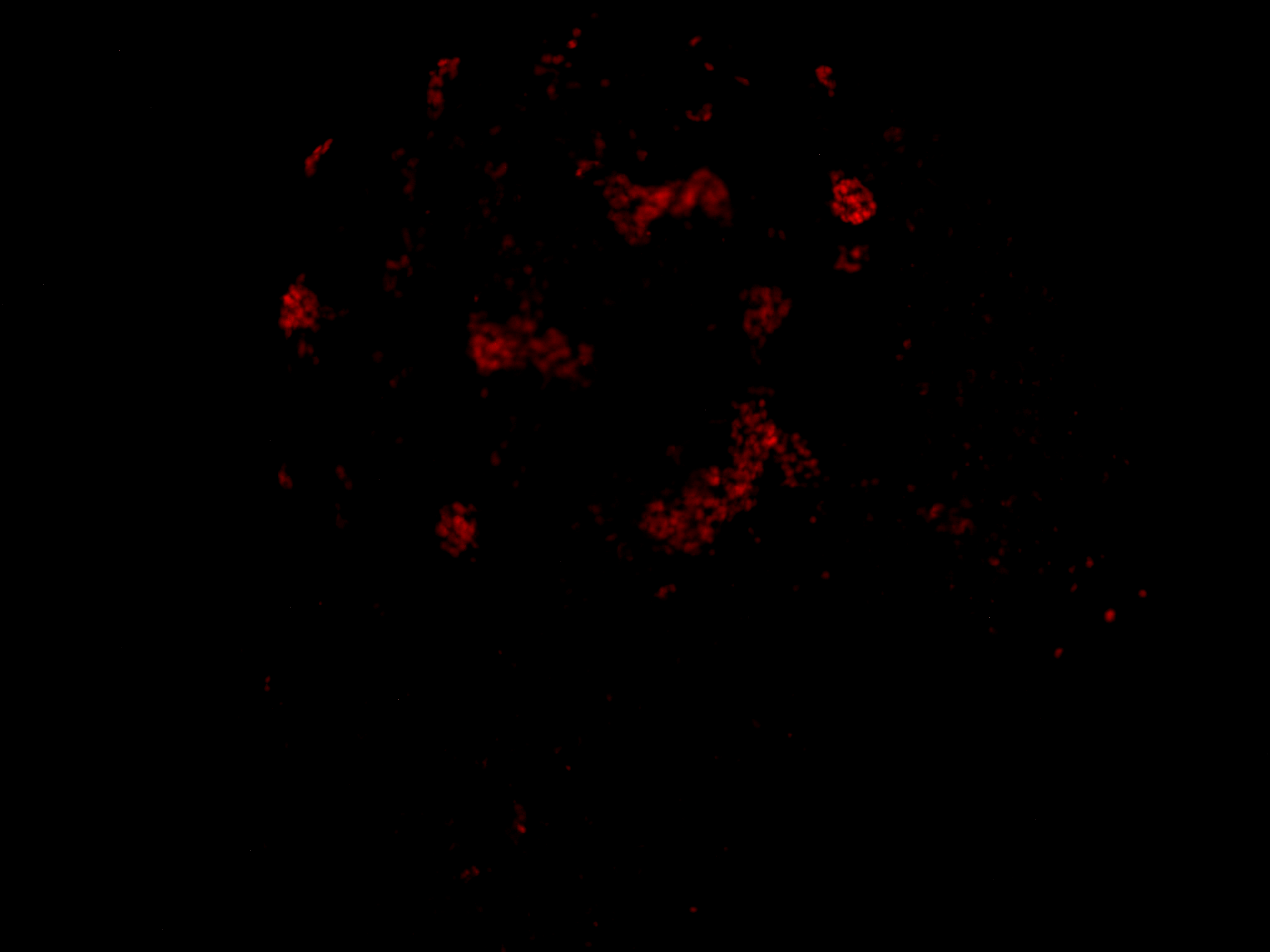

Supplement: Supplementary file 4 — Source data Fig. 1 [file 44318_2025_409_MOESM4_ESM.zip › EMBOJ-2024-118939R-Figure_1_Source_Data-sd/EMBOJ-2024-118939_Fig1H/D4_SB_FOXA2.tif]

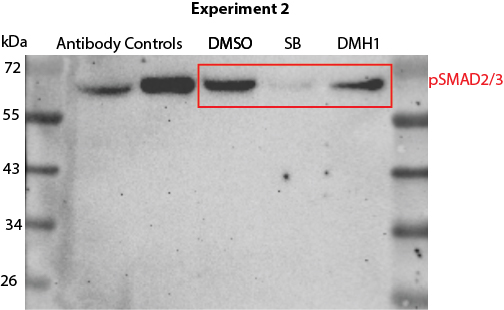

Supplement: Supplementary file 4 — Source data Fig. 1 [file 44318_2025_409_MOESM4_ESM.zip › EMBOJ-2024-118939R-Figure_1_Source_Data-sd/EMBOJ-2024-118939_Fig1B/pSMAD2_Experiment_2.jpg]

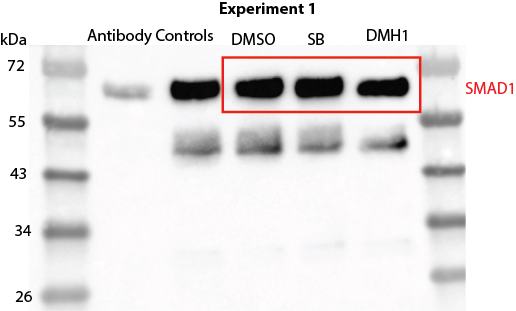

Supplement: Supplementary file 4 — Source data Fig. 1 [file 44318_2025_409_MOESM4_ESM.zip › EMBOJ-2024-118939R-Figure_1_Source_Data-sd/EMBOJ-2024-118939_Fig1B/SMAD1_Experiment_1.jpg]

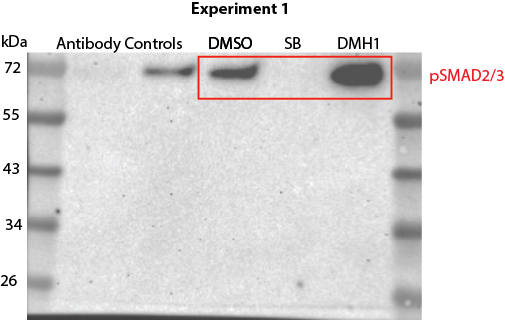

Supplement: Supplementary file 4 — Source data Fig. 1 [file 44318_2025_409_MOESM4_ESM.zip › EMBOJ-2024-118939R-Figure_1_Source_Data-sd/EMBOJ-2024-118939_Fig1B/pSMAD2_Experiment_1.jpg]

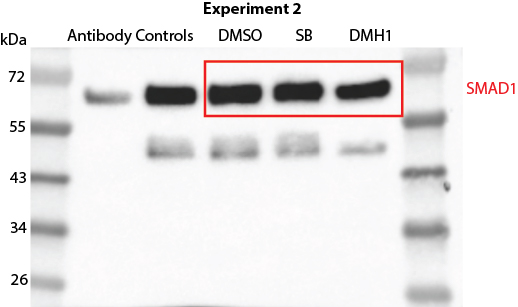

Supplement: Supplementary file 4 — Source data Fig. 1 [file 44318_2025_409_MOESM4_ESM.zip › EMBOJ-2024-118939R-Figure_1_Source_Data-sd/EMBOJ-2024-118939_Fig1B/SMAD1_Experiment_2.jpg]

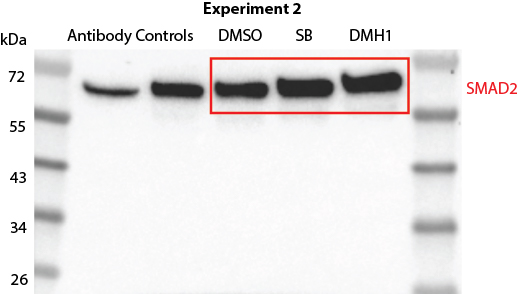

Supplement: Supplementary file 4 — Source data Fig. 1 [file 44318_2025_409_MOESM4_ESM.zip › EMBOJ-2024-118939R-Figure_1_Source_Data-sd/EMBOJ-2024-118939_Fig1B/SMAD2_Experiment_2.jpg]

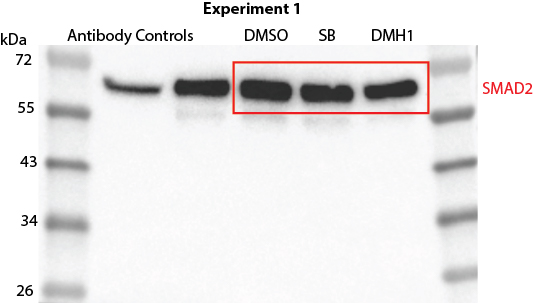

Supplement: Supplementary file 4 — Source data Fig. 1 [file 44318_2025_409_MOESM4_ESM.zip › EMBOJ-2024-118939R-Figure_1_Source_Data-sd/EMBOJ-2024-118939_Fig1B/SMAD2_Experiment_1.jpg]

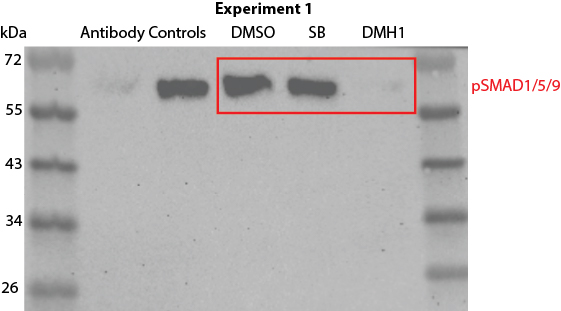

Supplement: Supplementary file 4 — Source data Fig. 1 [file 44318_2025_409_MOESM4_ESM.zip › EMBOJ-2024-118939R-Figure_1_Source_Data-sd/EMBOJ-2024-118939_Fig1B/pSMAD159_Experiment_1.jpg]

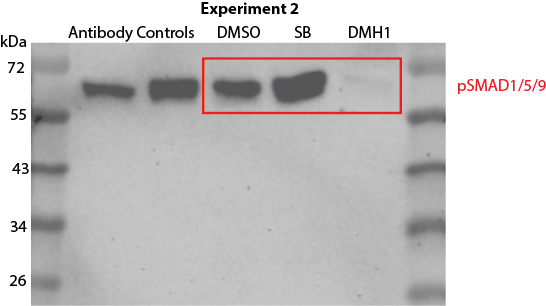

Supplement: Supplementary file 4 — Source data Fig. 1 [file 44318_2025_409_MOESM4_ESM.zip › EMBOJ-2024-118939R-Figure_1_Source_Data-sd/EMBOJ-2024-118939_Fig1B/pSMAD159_Experiment_2.jpg]

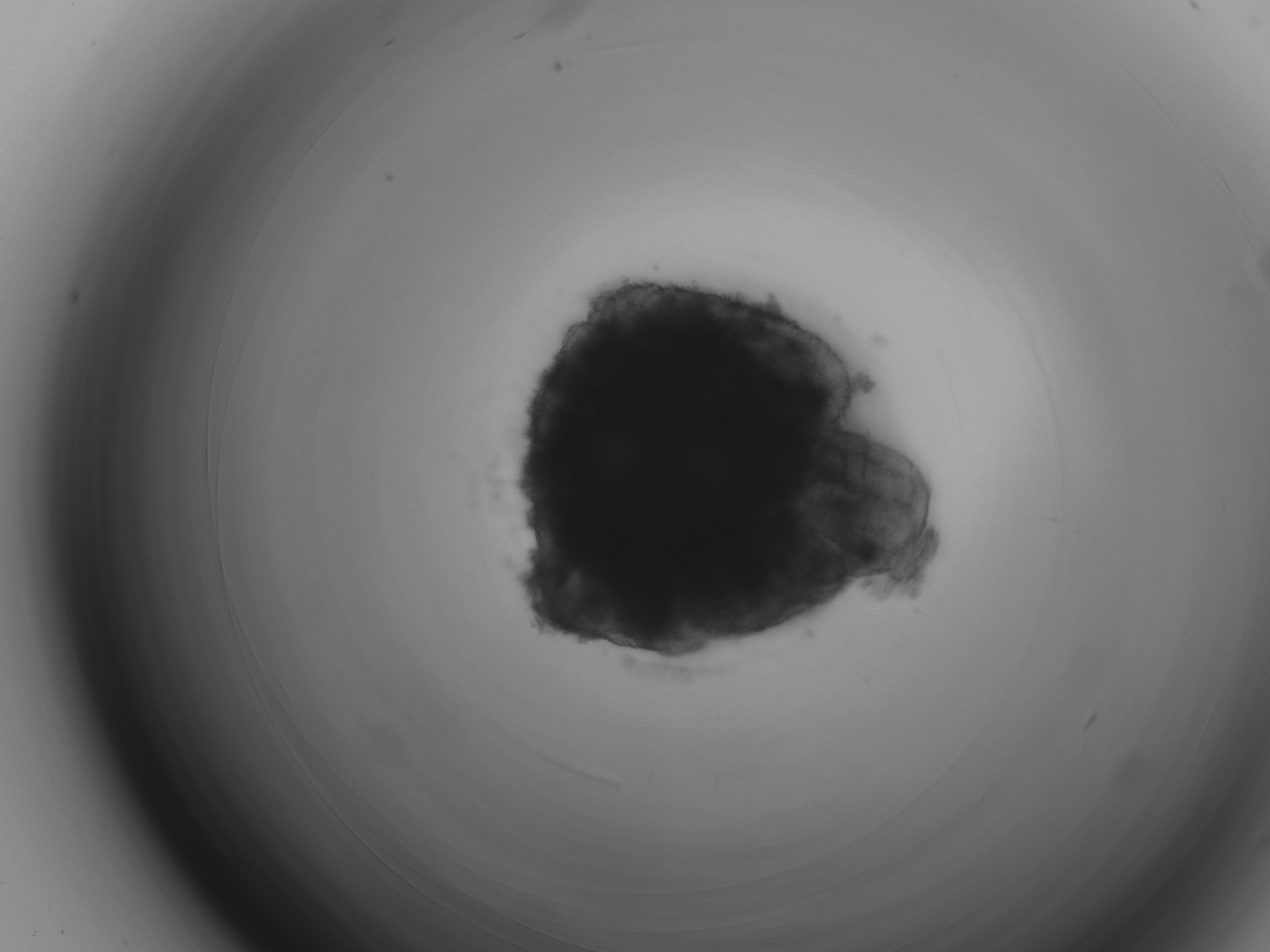

Supplement: Supplementary file 4 — Source data Fig. 1 [file 44318_2025_409_MOESM4_ESM.zip › EMBOJ-2024-118939R-Figure_1_Source_Data-sd/EMBOJ-2024-118939_Fig1D/D8_DMH1_Trans.tif]

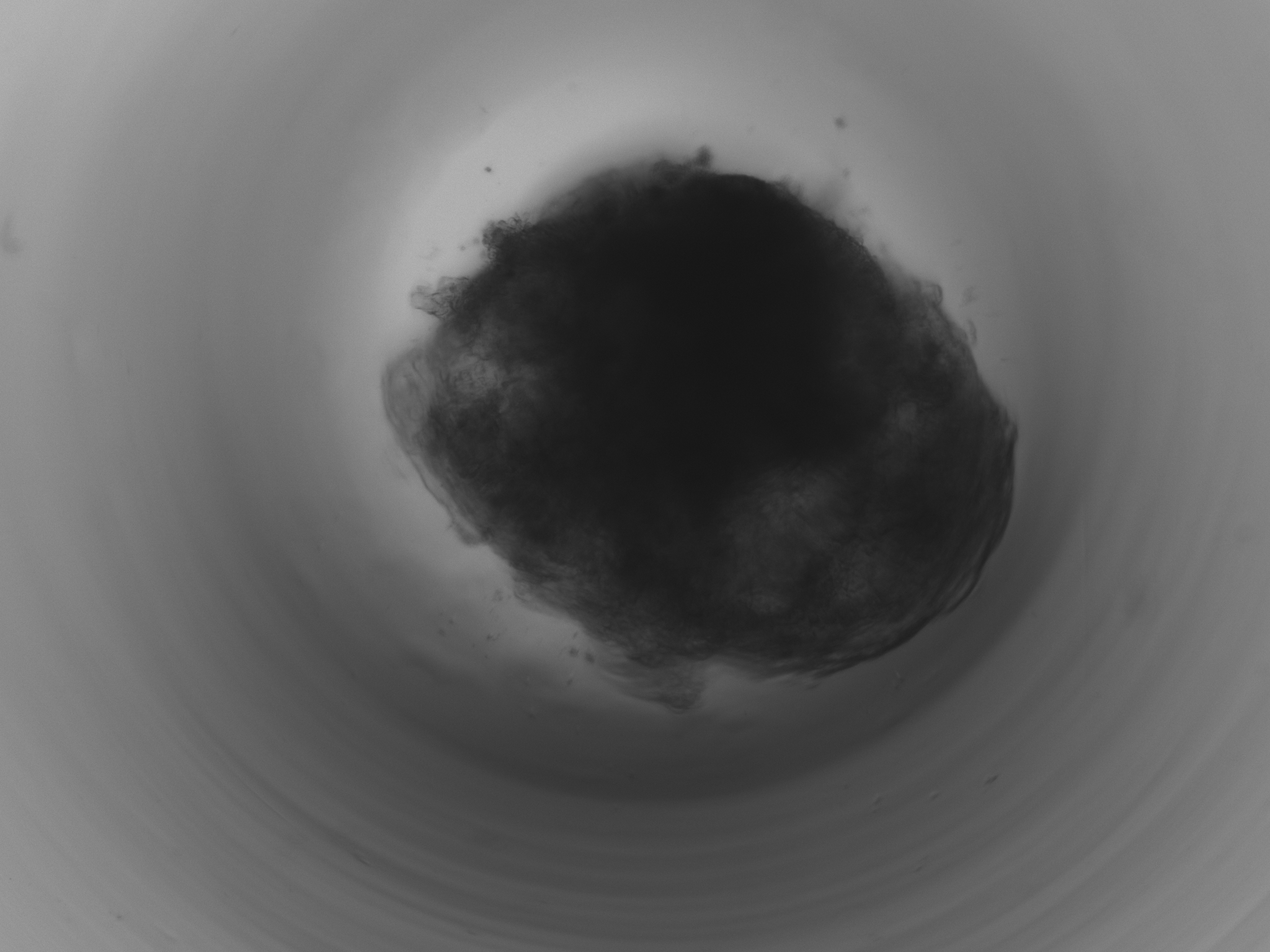

Supplement: Supplementary file 4 — Source data Fig. 1 [file 44318_2025_409_MOESM4_ESM.zip › EMBOJ-2024-118939R-Figure_1_Source_Data-sd/EMBOJ-2024-118939_Fig1D/D12_SB_Trans.tif]

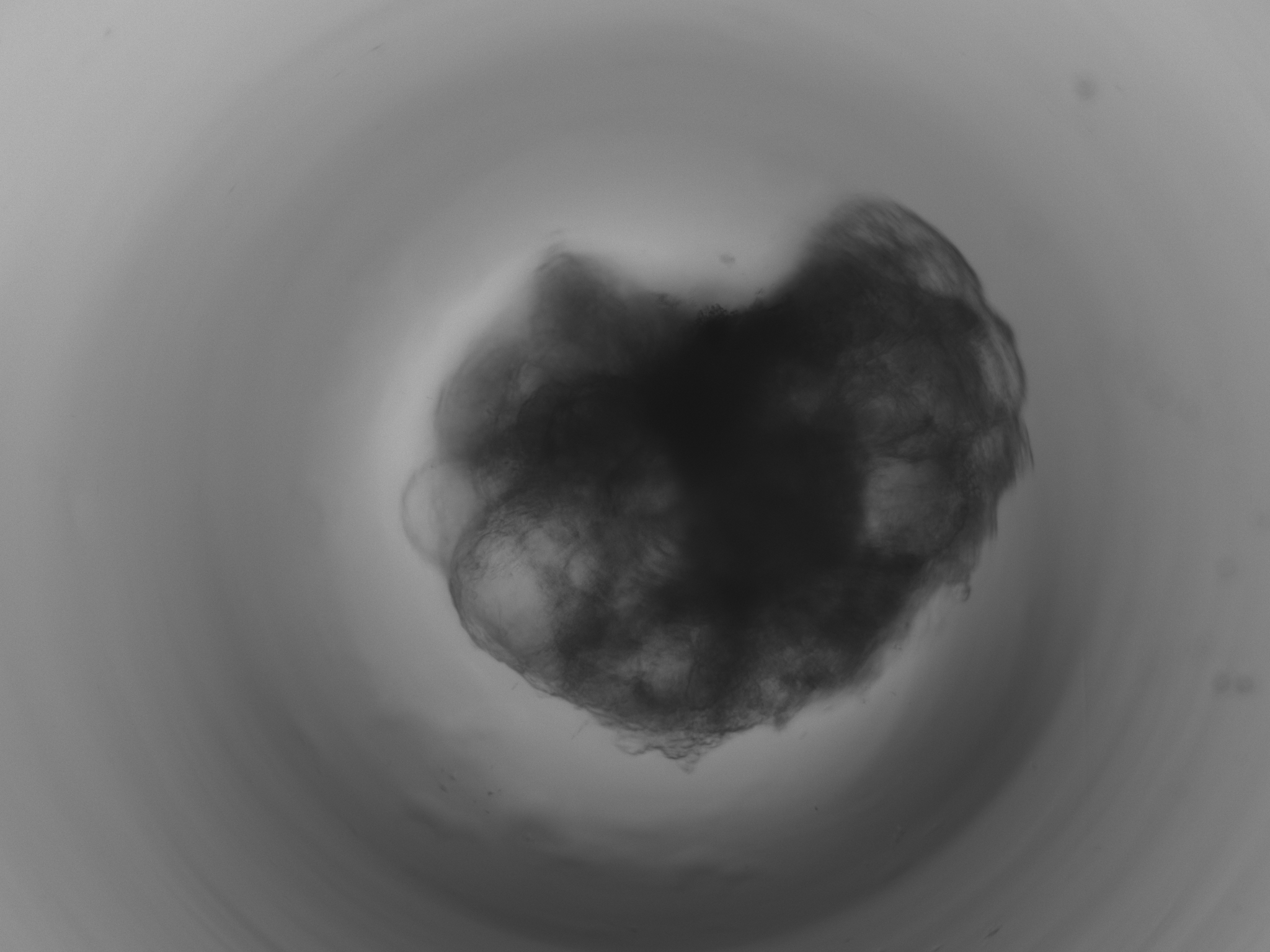

Supplement: Supplementary file 4 — Source data Fig. 1 [file 44318_2025_409_MOESM4_ESM.zip › EMBOJ-2024-118939R-Figure_1_Source_Data-sd/EMBOJ-2024-118939_Fig1D/D8_SB_Trans.tif]

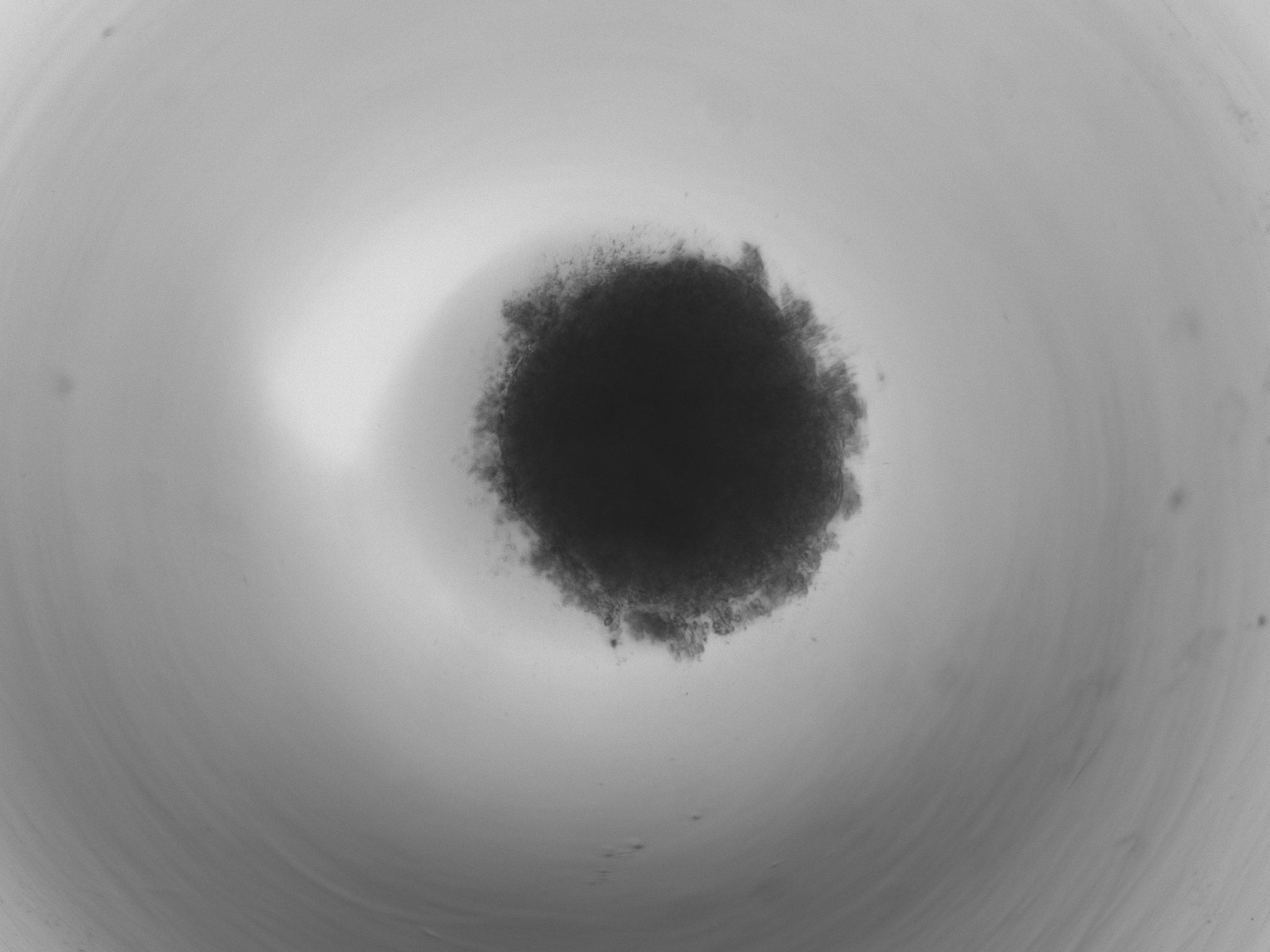

Supplement: Supplementary file 4 — Source data Fig. 1 [file 44318_2025_409_MOESM4_ESM.zip › EMBOJ-2024-118939R-Figure_1_Source_Data-sd/EMBOJ-2024-118939_Fig1D/D4_Veh_Trans.tif]

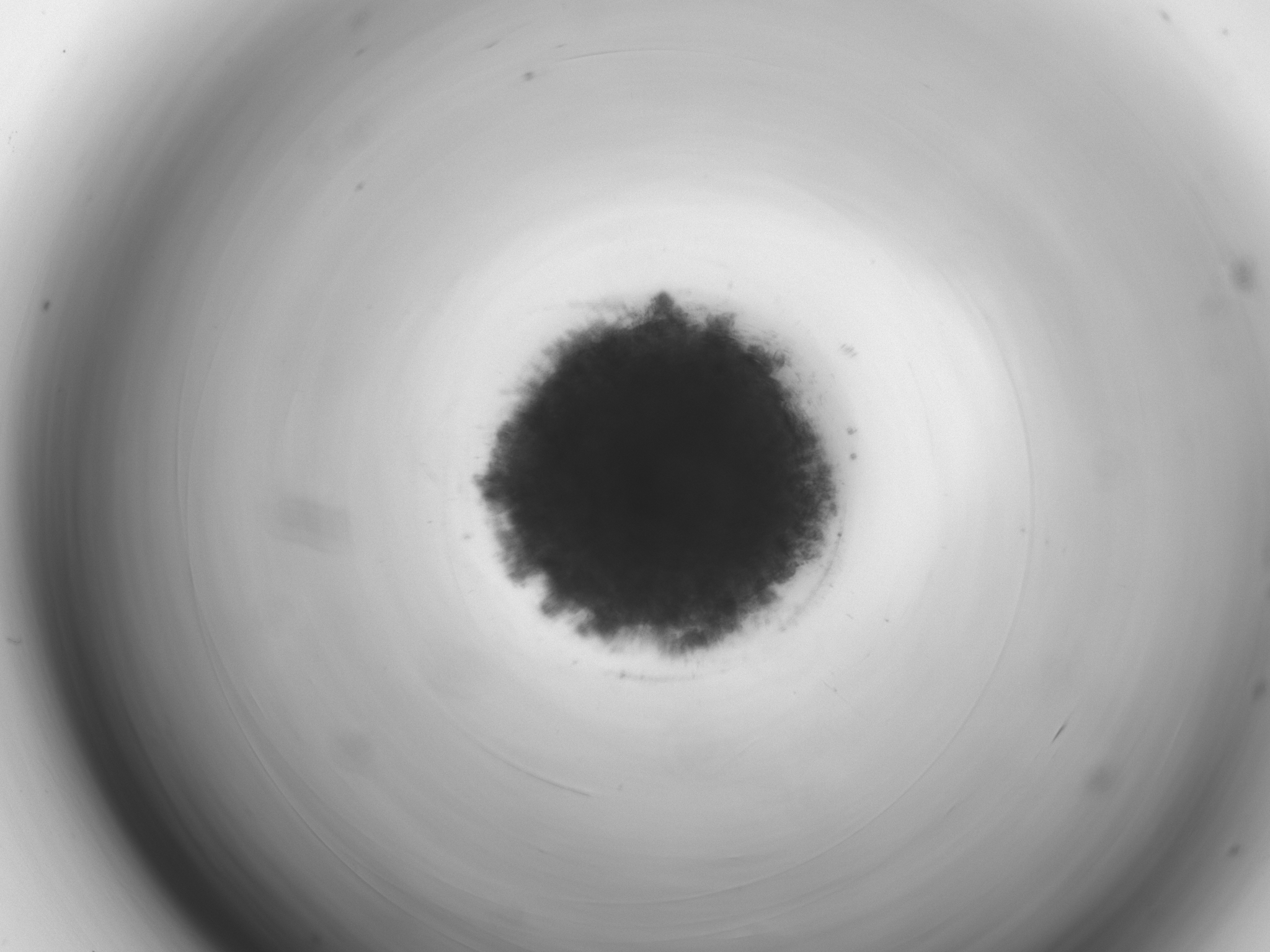

Supplement: Supplementary file 4 — Source data Fig. 1 [file 44318_2025_409_MOESM4_ESM.zip › EMBOJ-2024-118939R-Figure_1_Source_Data-sd/EMBOJ-2024-118939_Fig1D/D4_DMH1_Trans.tif]

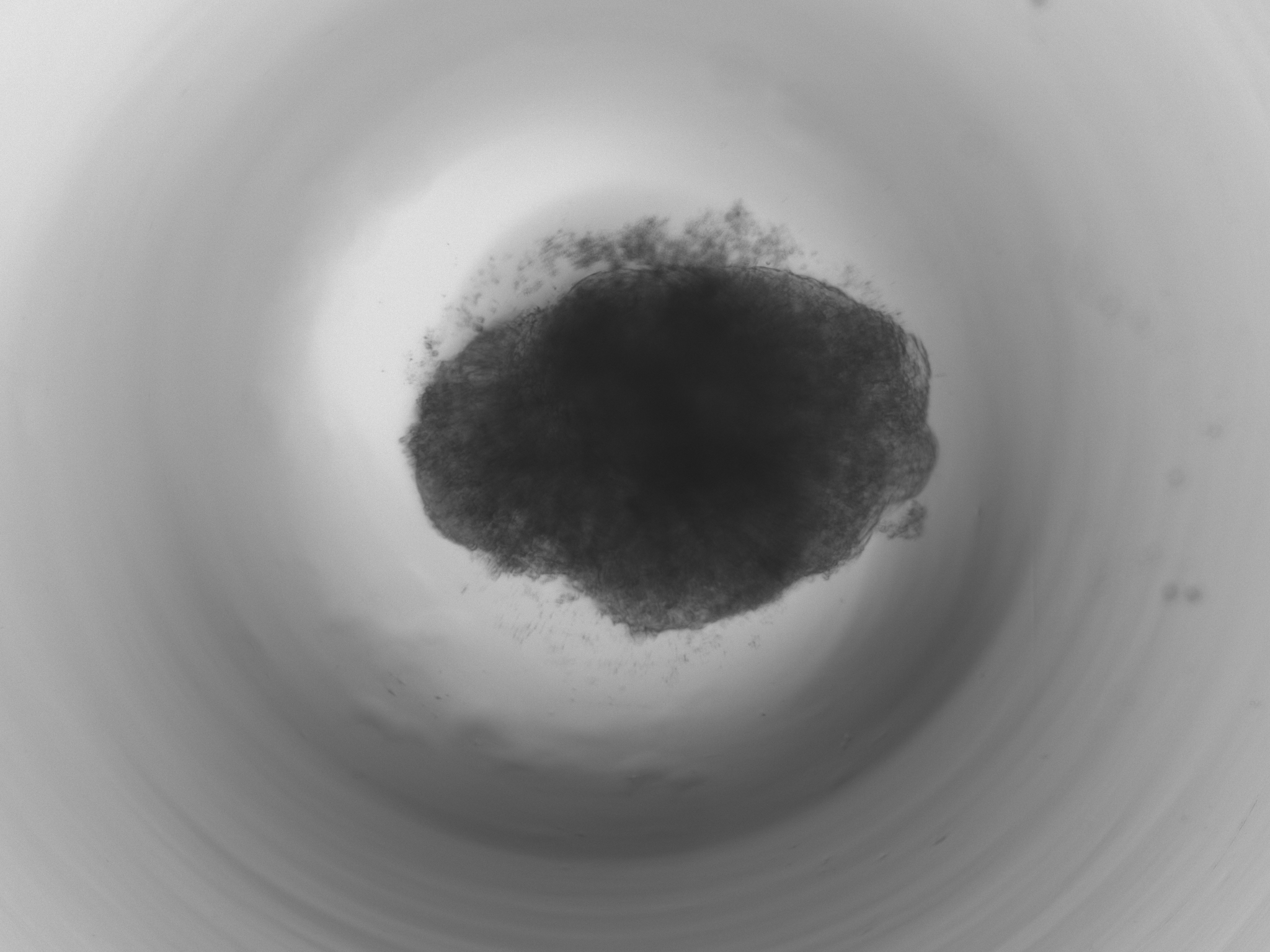

Supplement: Supplementary file 4 — Source data Fig. 1 [file 44318_2025_409_MOESM4_ESM.zip › EMBOJ-2024-118939R-Figure_1_Source_Data-sd/EMBOJ-2024-118939_Fig1D/D4_SB_Trans.tif]

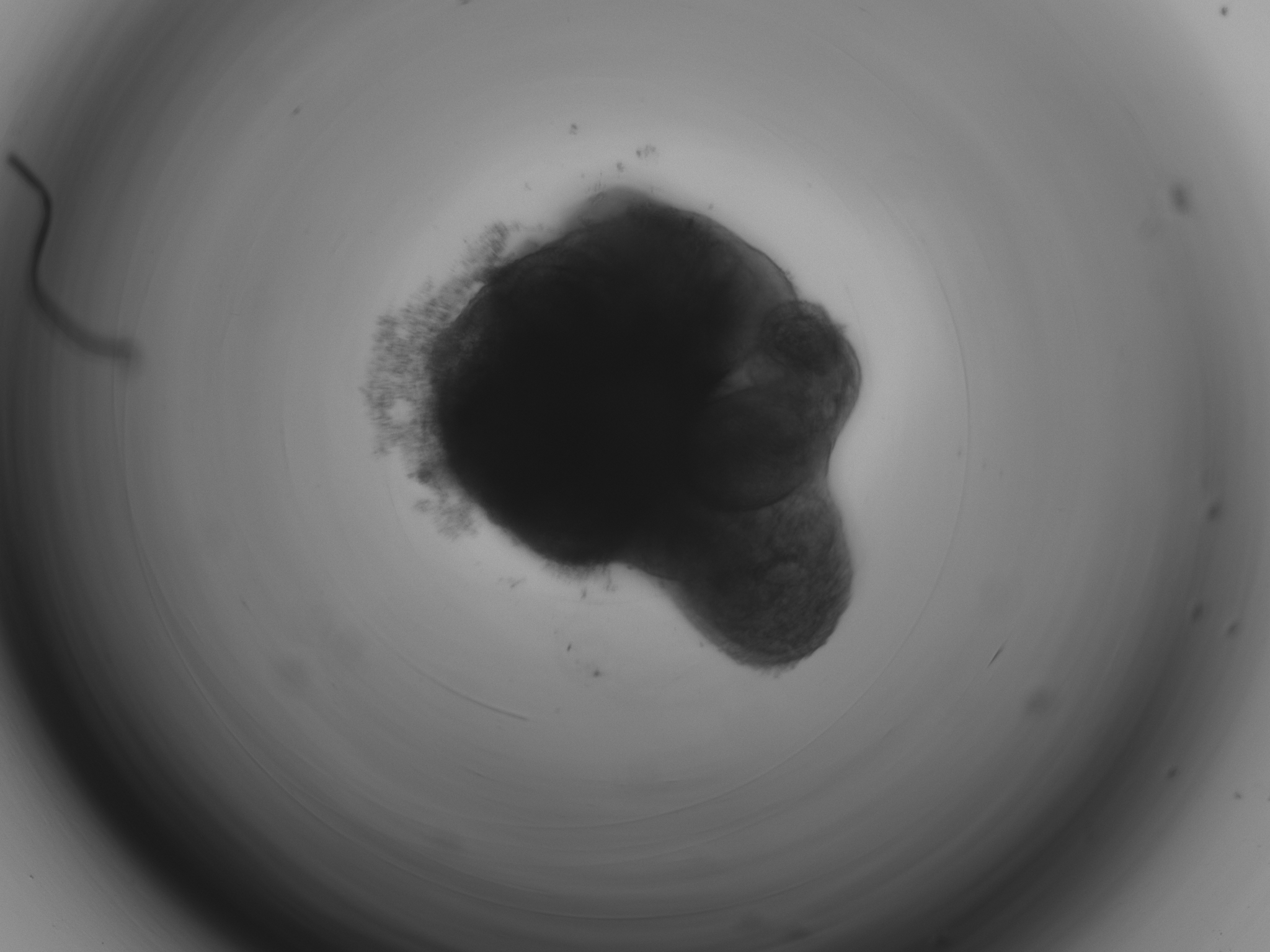

Supplement: Supplementary file 4 — Source data Fig. 1 [file 44318_2025_409_MOESM4_ESM.zip › EMBOJ-2024-118939R-Figure_1_Source_Data-sd/EMBOJ-2024-118939_Fig1D/D12_DMH1_Trans.tif]

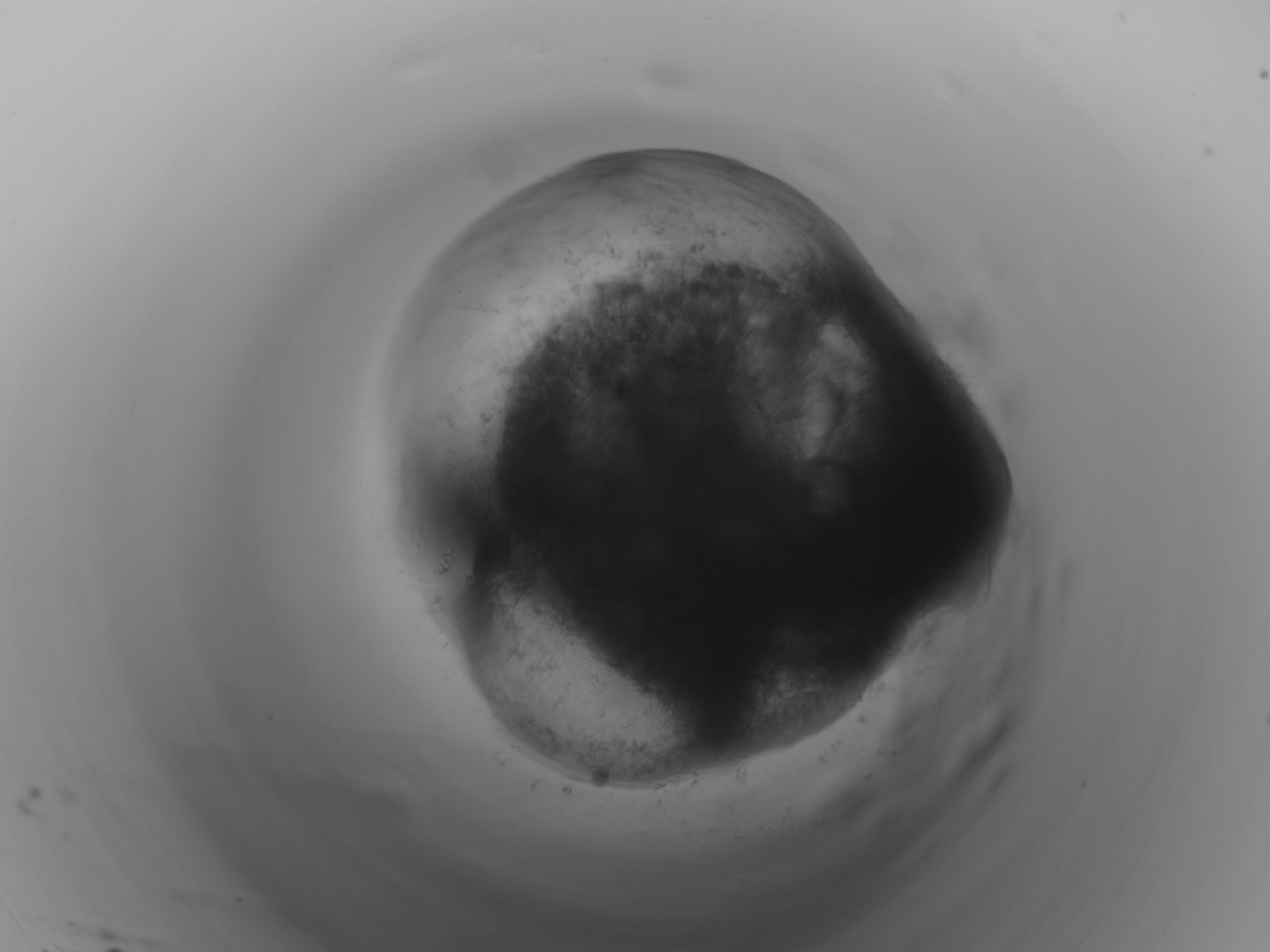

Supplement: Supplementary file 4 — Source data Fig. 1 [file 44318_2025_409_MOESM4_ESM.zip › EMBOJ-2024-118939R-Figure_1_Source_Data-sd/EMBOJ-2024-118939_Fig1D/D8_Veh_Trans.tif]

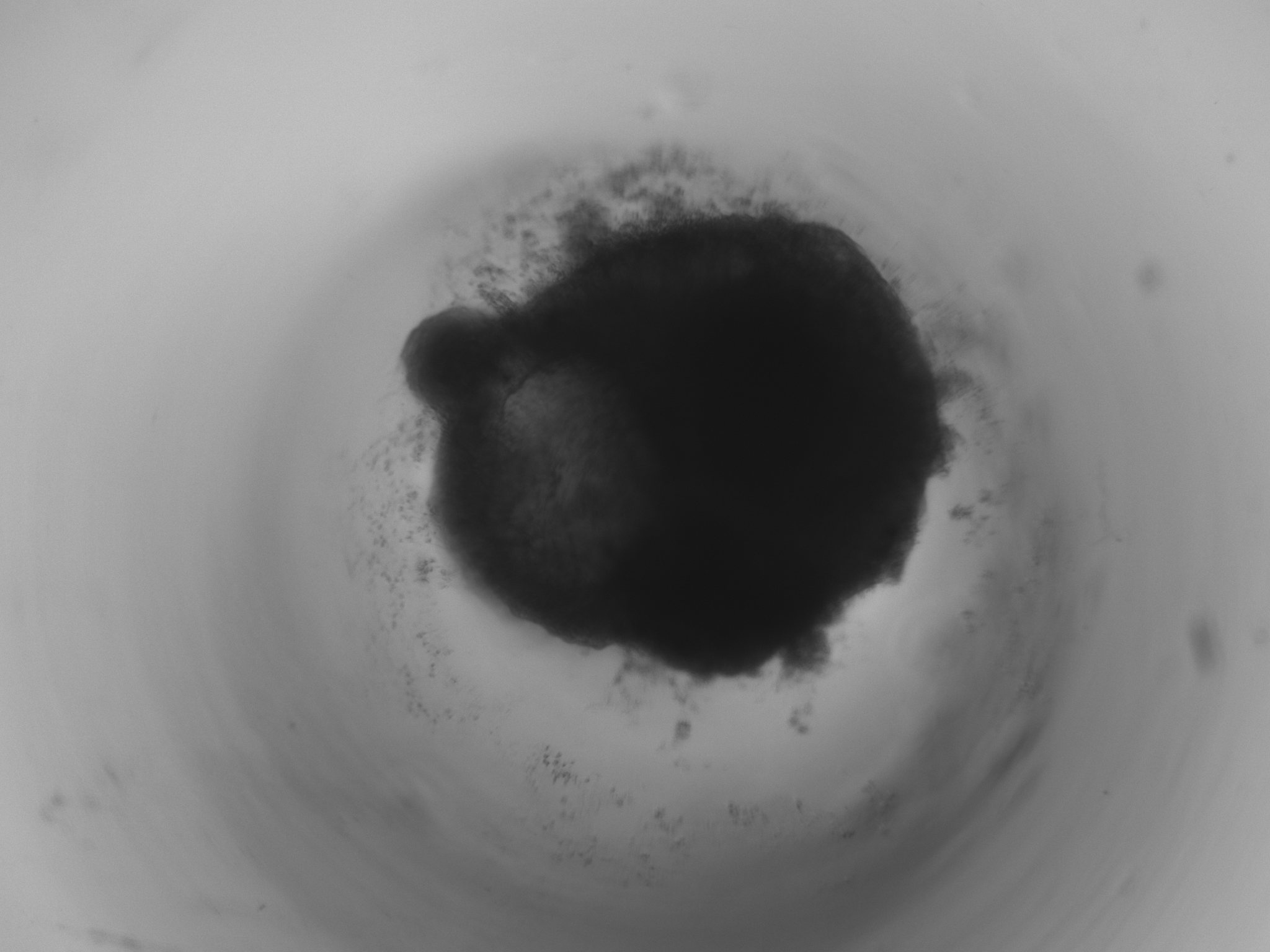

Supplement: Supplementary file 4 — Source data Fig. 1 [file 44318_2025_409_MOESM4_ESM.zip › EMBOJ-2024-118939R-Figure_1_Source_Data-sd/EMBOJ-2024-118939_Fig1D/D12_Veh_Trans.tif]

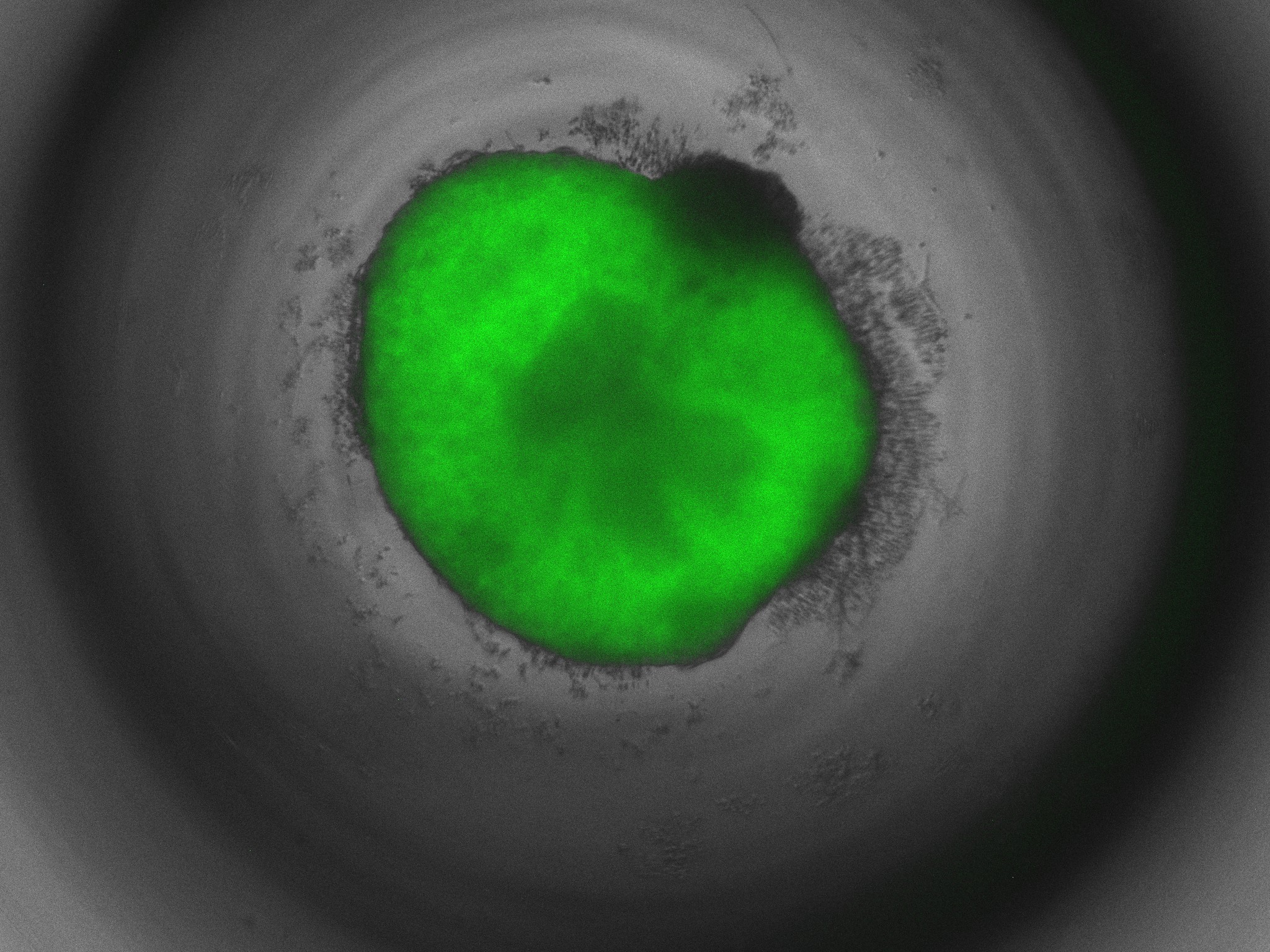

Supplement: Supplementary file 5 — Source data Fig. 2 [file 44318_2025_409_MOESM5_ESM.zip › EMBOJ-2024-118939R-Figure_2_Source_Data-sd/EMBOJ-2024-118939_Fig2B/WT1_Veh_4.jpg]

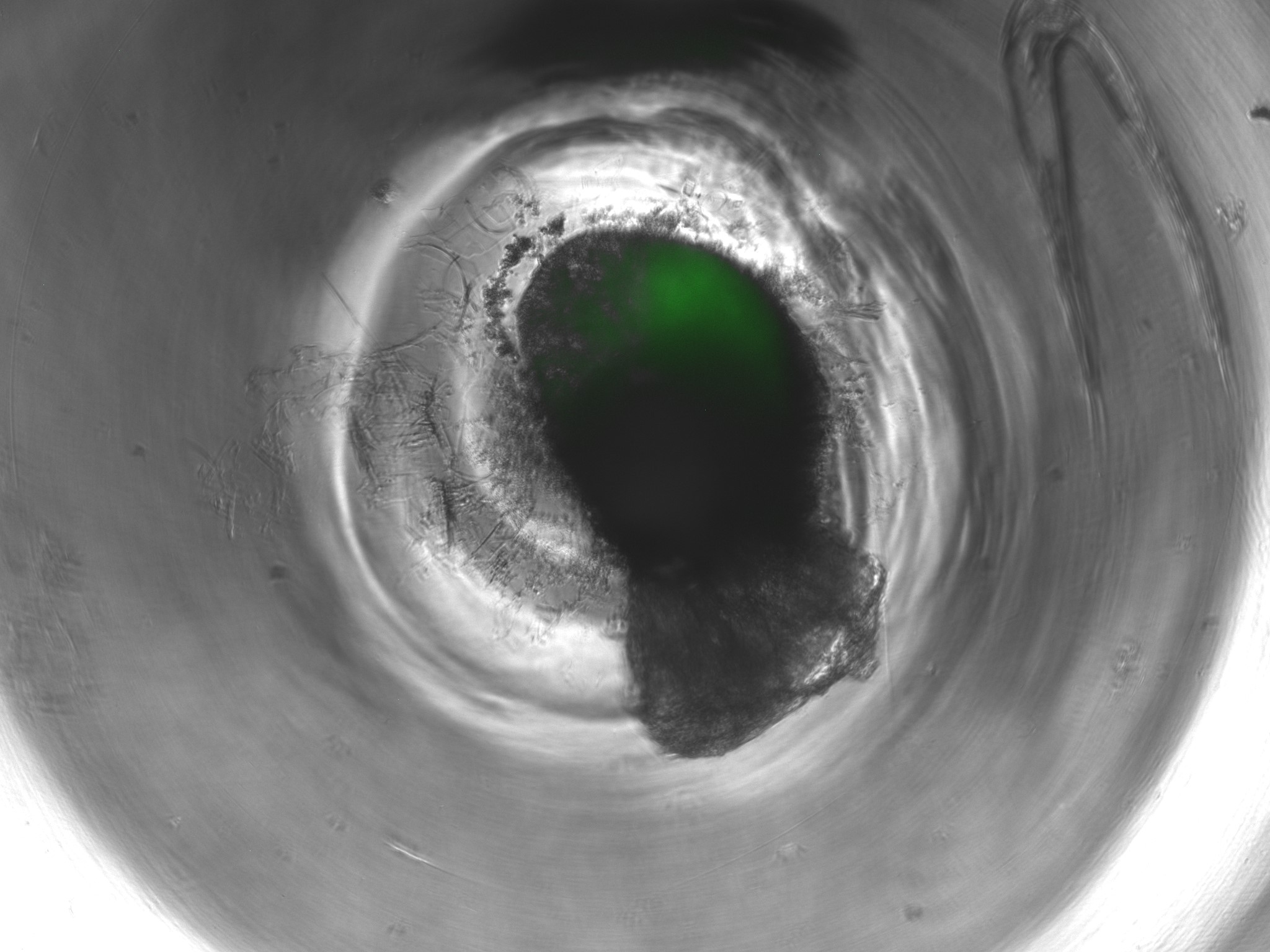

Supplement: Supplementary file 5 — Source data Fig. 2 [file 44318_2025_409_MOESM5_ESM.zip › EMBOJ-2024-118939R-Figure_2_Source_Data-sd/EMBOJ-2024-118939_Fig2B/WT_SB_1.jpg]

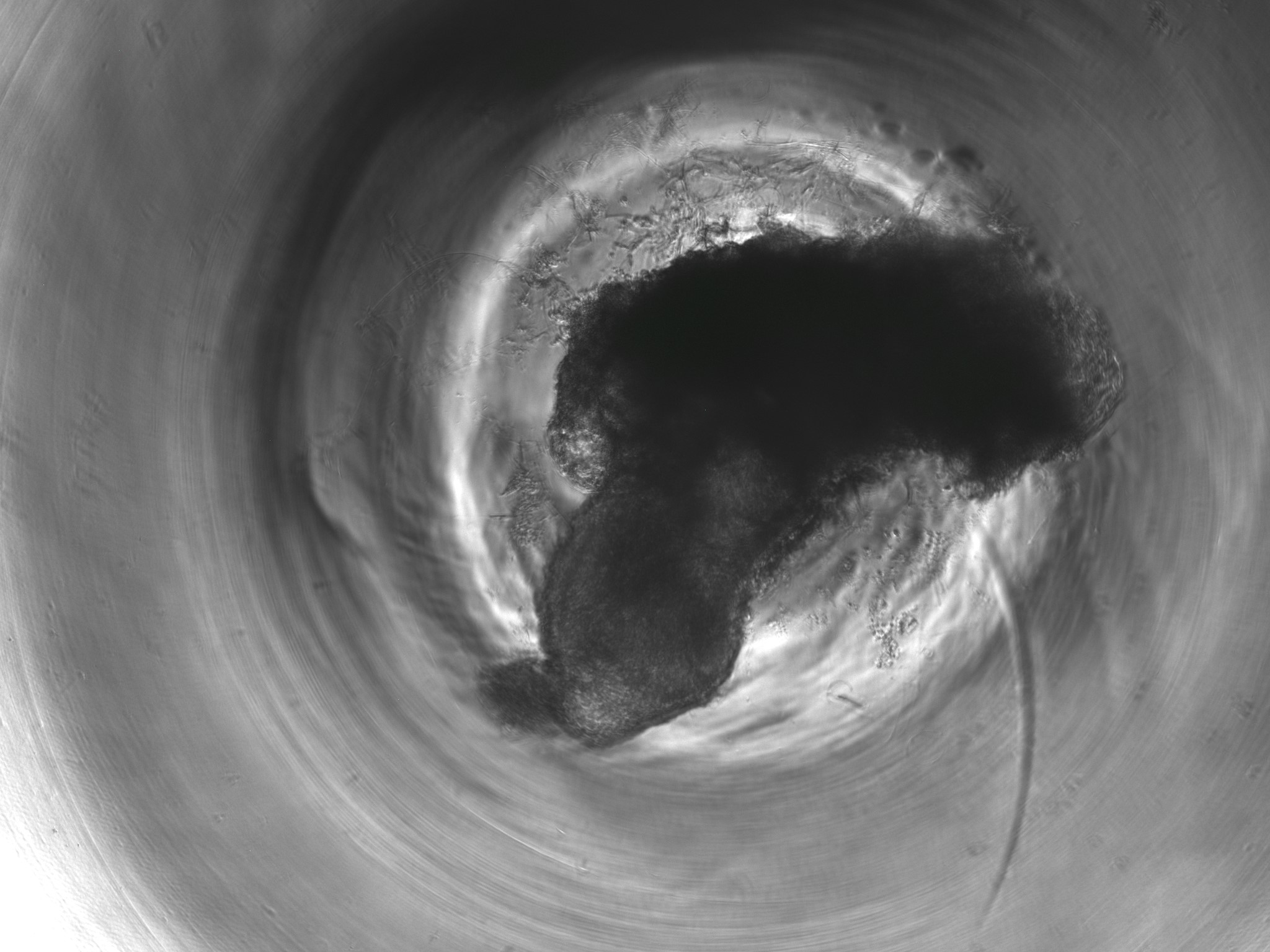

Supplement: Supplementary file 5 — Source data Fig. 2 [file 44318_2025_409_MOESM5_ESM.zip › EMBOJ-2024-118939R-Figure_2_Source_Data-sd/EMBOJ-2024-118939_Fig2B/WT_SB_3.jpg]

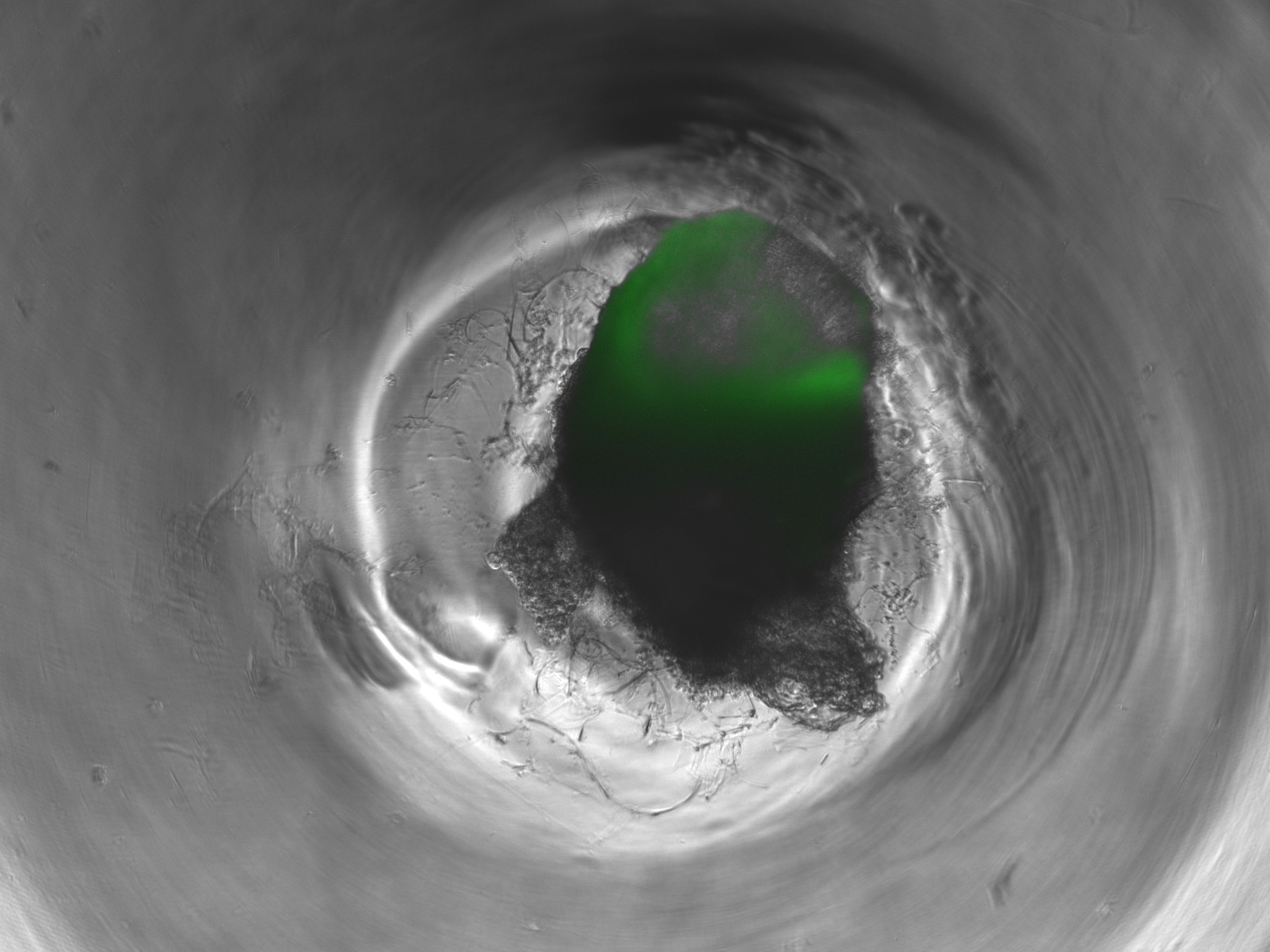

Supplement: Supplementary file 5 — Source data Fig. 2 [file 44318_2025_409_MOESM5_ESM.zip › EMBOJ-2024-118939R-Figure_2_Source_Data-sd/EMBOJ-2024-118939_Fig2B/WT_SB_2.jpg]

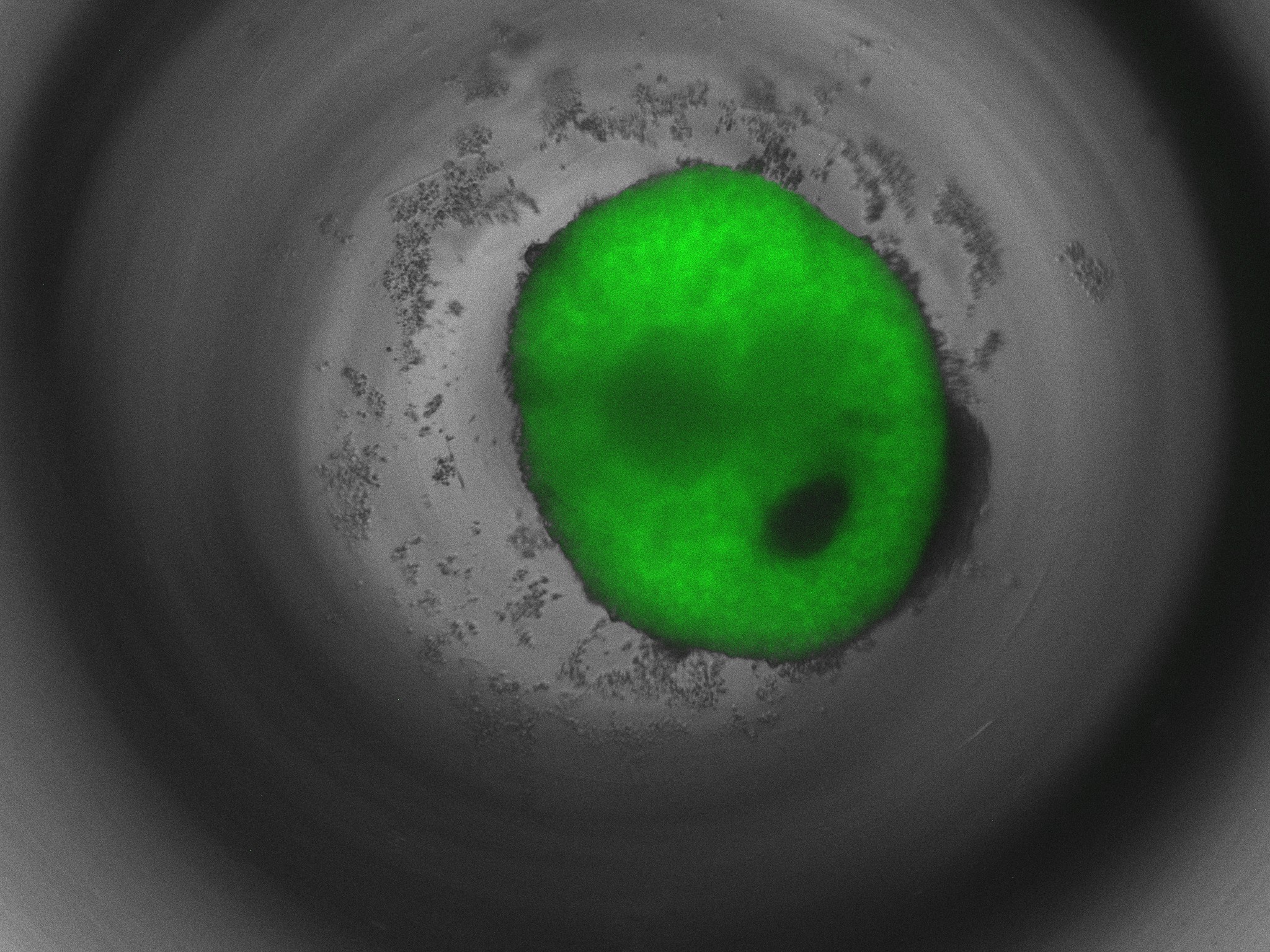

Supplement: Supplementary file 5 — Source data Fig. 2 [file 44318_2025_409_MOESM5_ESM.zip › EMBOJ-2024-118939R-Figure_2_Source_Data-sd/EMBOJ-2024-118939_Fig2B/WT1_Veh_2.jpg]

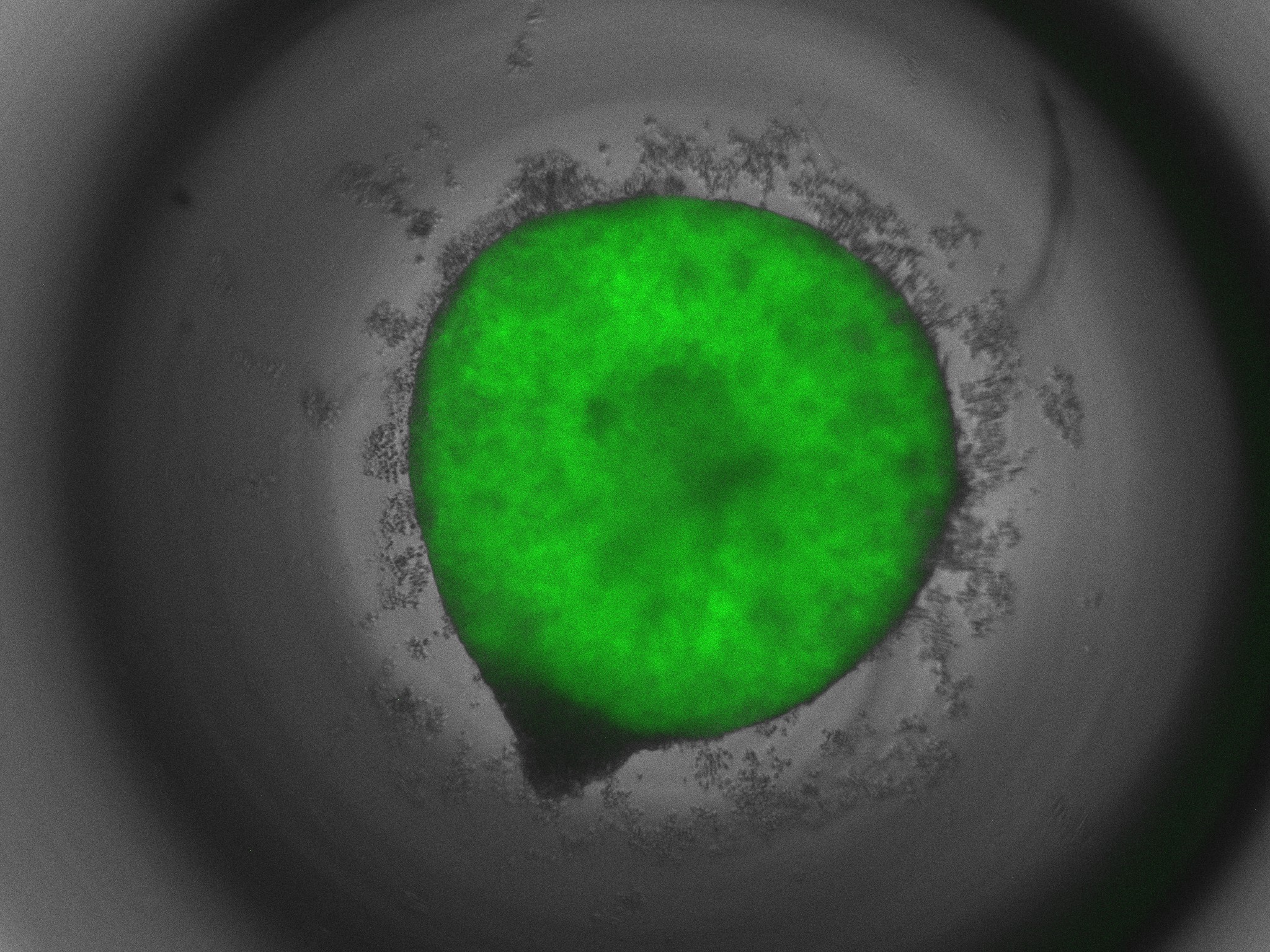

Supplement: Supplementary file 5 — Source data Fig. 2 [file 44318_2025_409_MOESM5_ESM.zip › EMBOJ-2024-118939R-Figure_2_Source_Data-sd/EMBOJ-2024-118939_Fig2B/WT1_Veh_3.jpg]

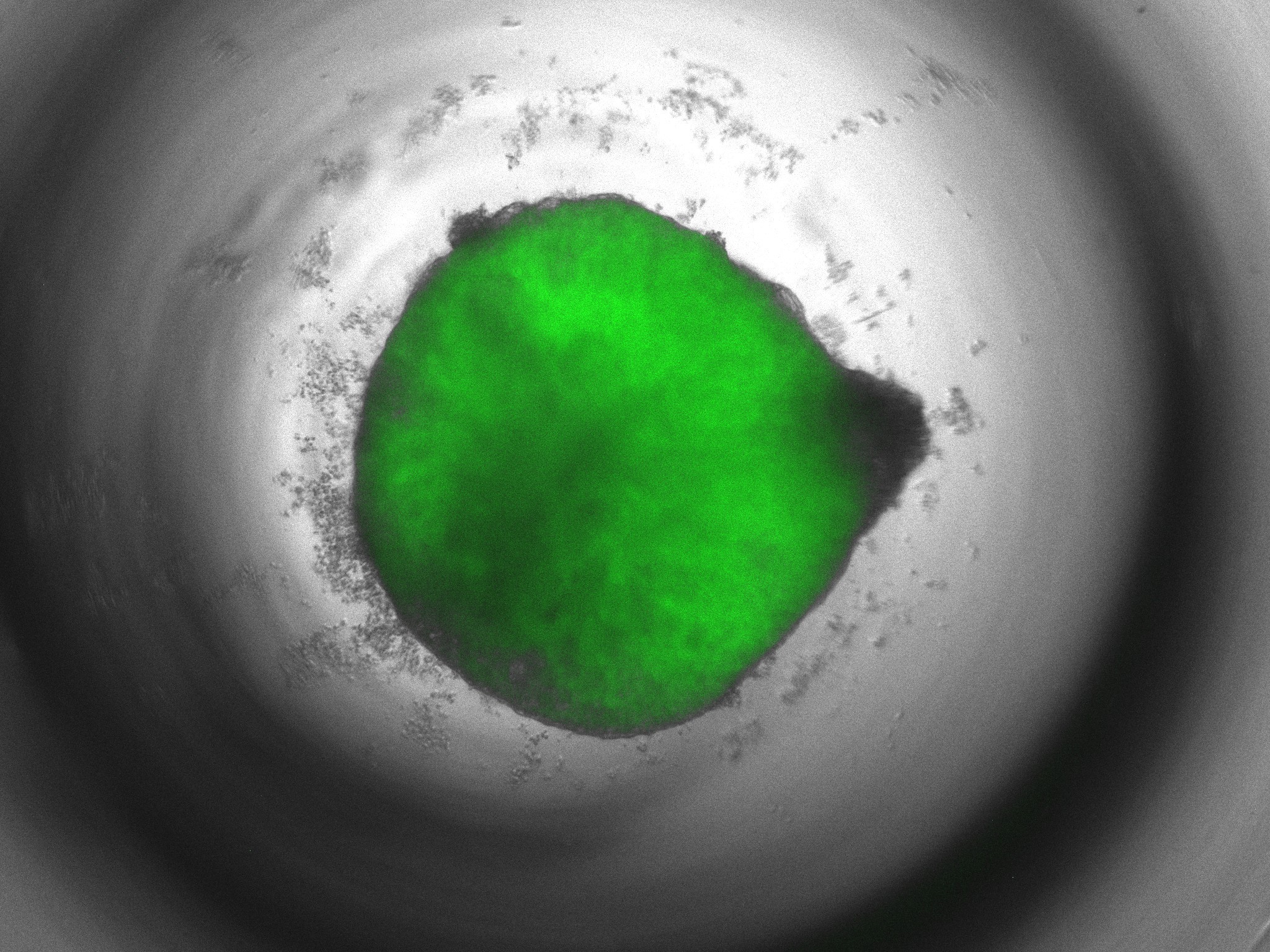

Supplement: Supplementary file 5 — Source data Fig. 2 [file 44318_2025_409_MOESM5_ESM.zip › EMBOJ-2024-118939R-Figure_2_Source_Data-sd/EMBOJ-2024-118939_Fig2B/WT1_Veh_1.jpg]

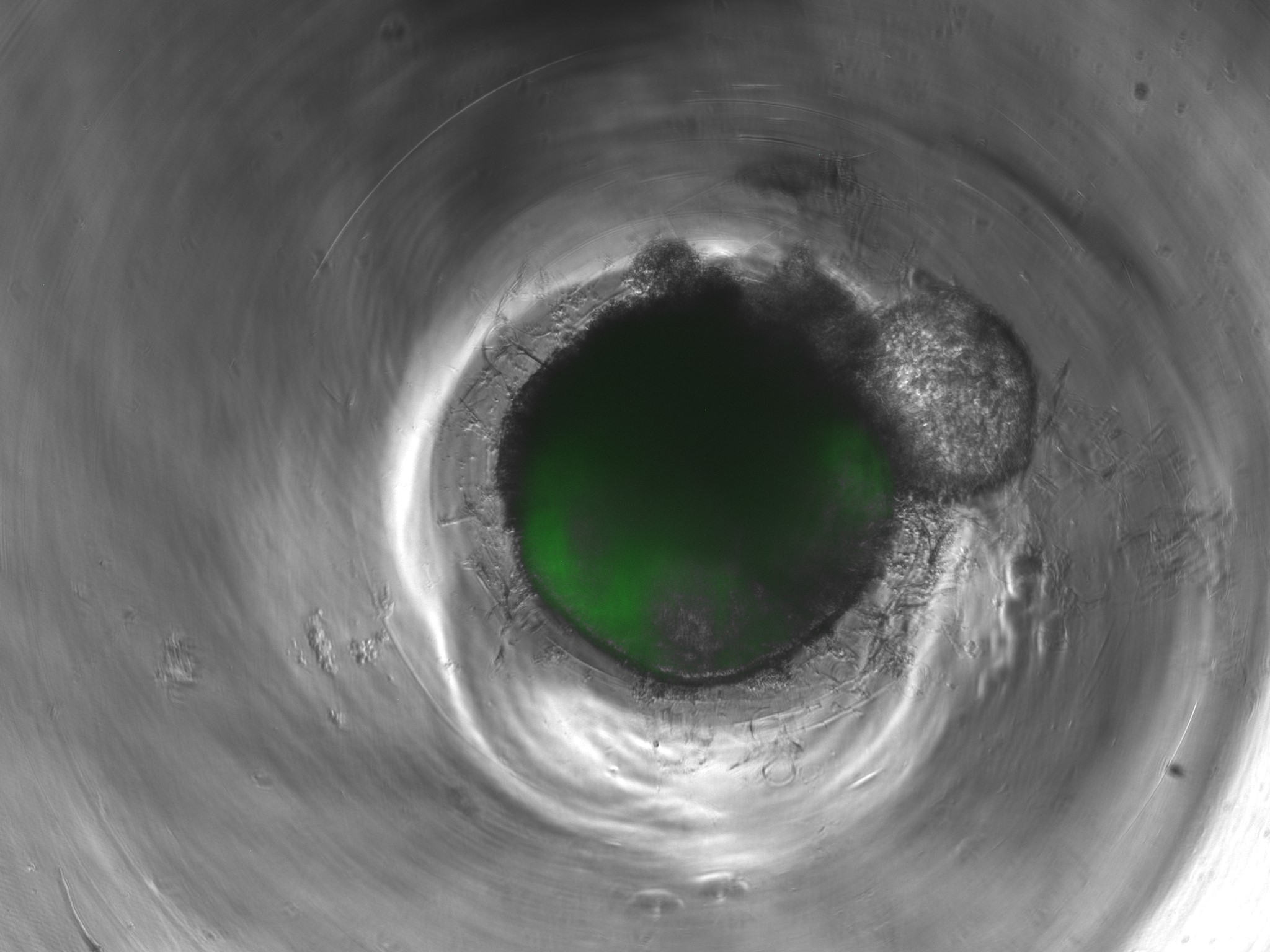

Supplement: Supplementary file 5 — Source data Fig. 2 [file 44318_2025_409_MOESM5_ESM.zip › EMBOJ-2024-118939R-Figure_2_Source_Data-sd/EMBOJ-2024-118939_Fig2B/WT_SB_4.jpg]

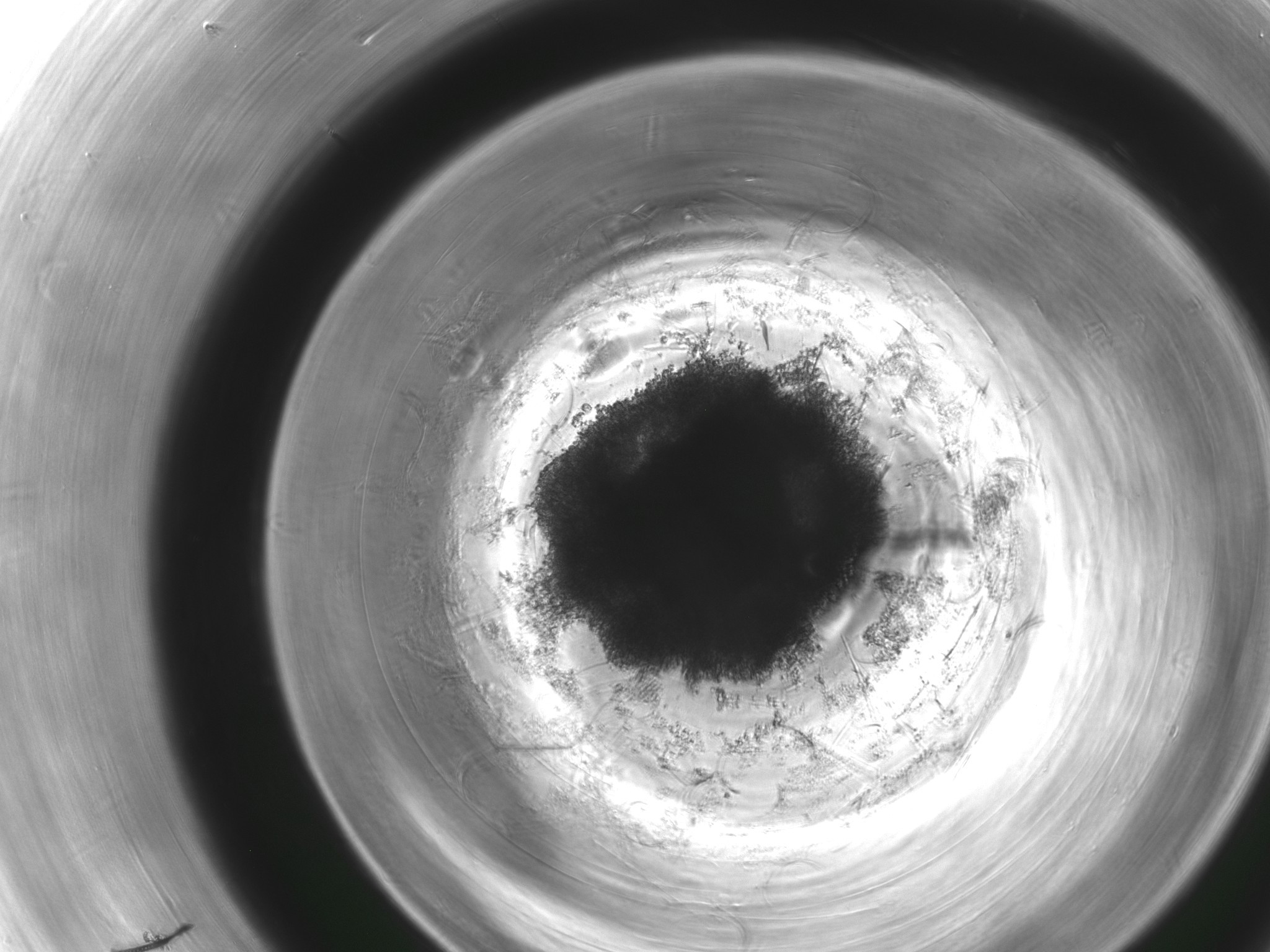

Supplement: Supplementary file 5 — Source data Fig. 2 [file 44318_2025_409_MOESM5_ESM.zip › EMBOJ-2024-118939R-Figure_2_Source_Data-sd/EMBOJ-2024-118939_Fig2B/YAP1_SB_1.jpg]

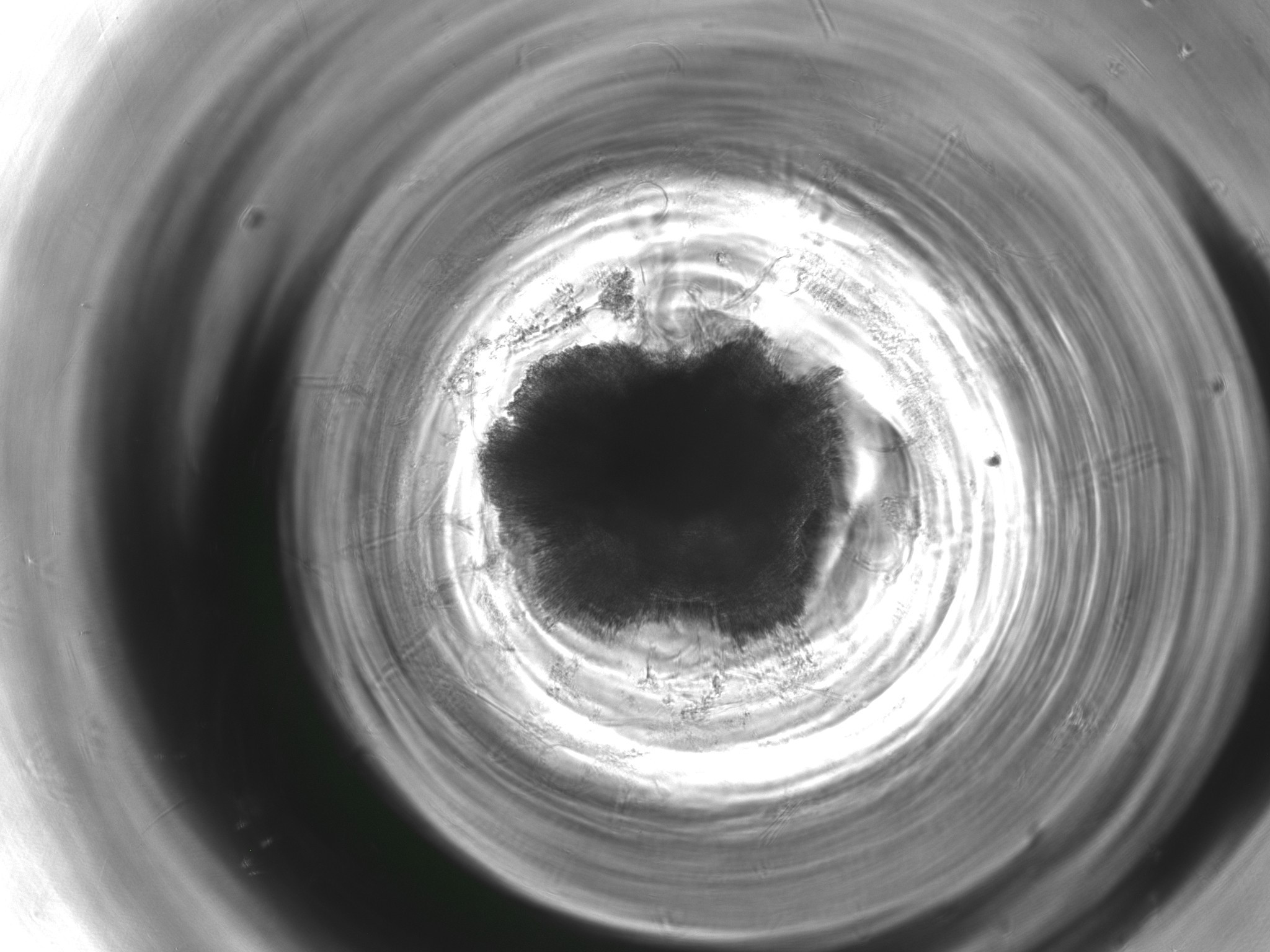

Supplement: Supplementary file 5 — Source data Fig. 2 [file 44318_2025_409_MOESM5_ESM.zip › EMBOJ-2024-118939R-Figure_2_Source_Data-sd/EMBOJ-2024-118939_Fig2B/YAP1_Veh_2.jpg]

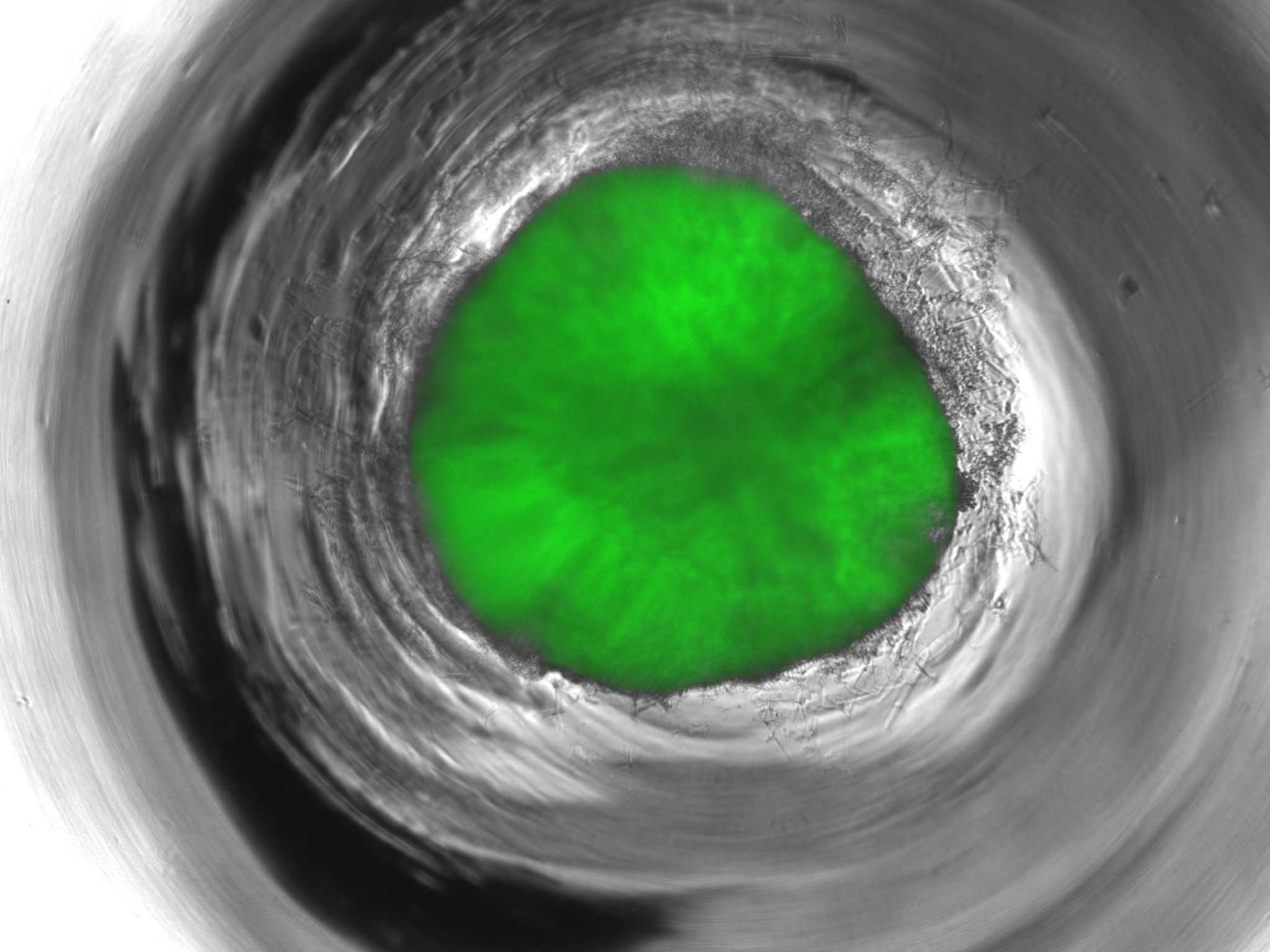

Supplement: Supplementary file 5 — Source data Fig. 2 [file 44318_2025_409_MOESM5_ESM.zip › EMBOJ-2024-118939R-Figure_2_Source_Data-sd/EMBOJ-2024-118939_Fig2B/HAND1_Veh_2.jpg]

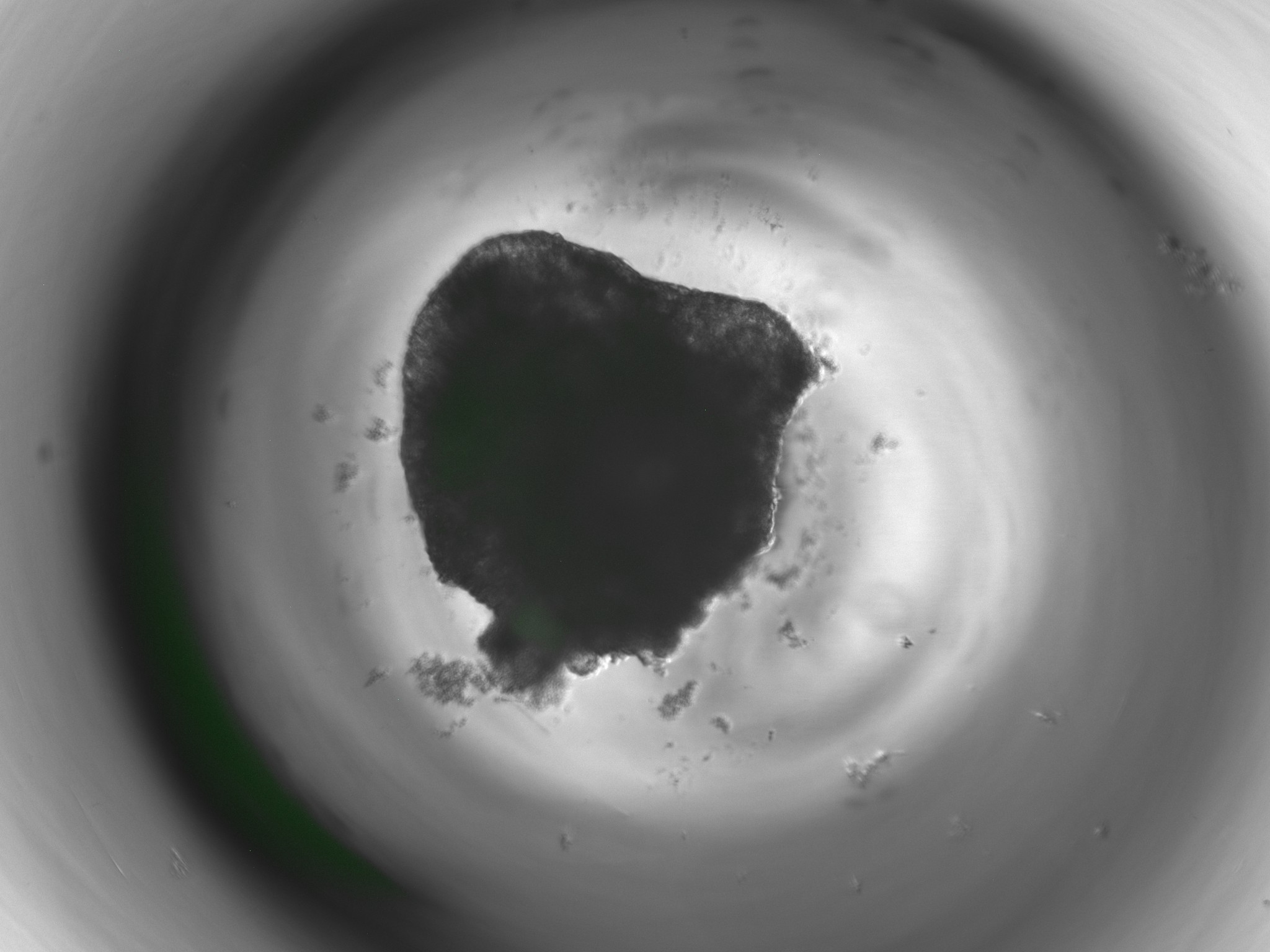

Supplement: Supplementary file 5 — Source data Fig. 2 [file 44318_2025_409_MOESM5_ESM.zip › EMBOJ-2024-118939R-Figure_2_Source_Data-sd/EMBOJ-2024-118939_Fig2B/WT1_SB_4.jpg]

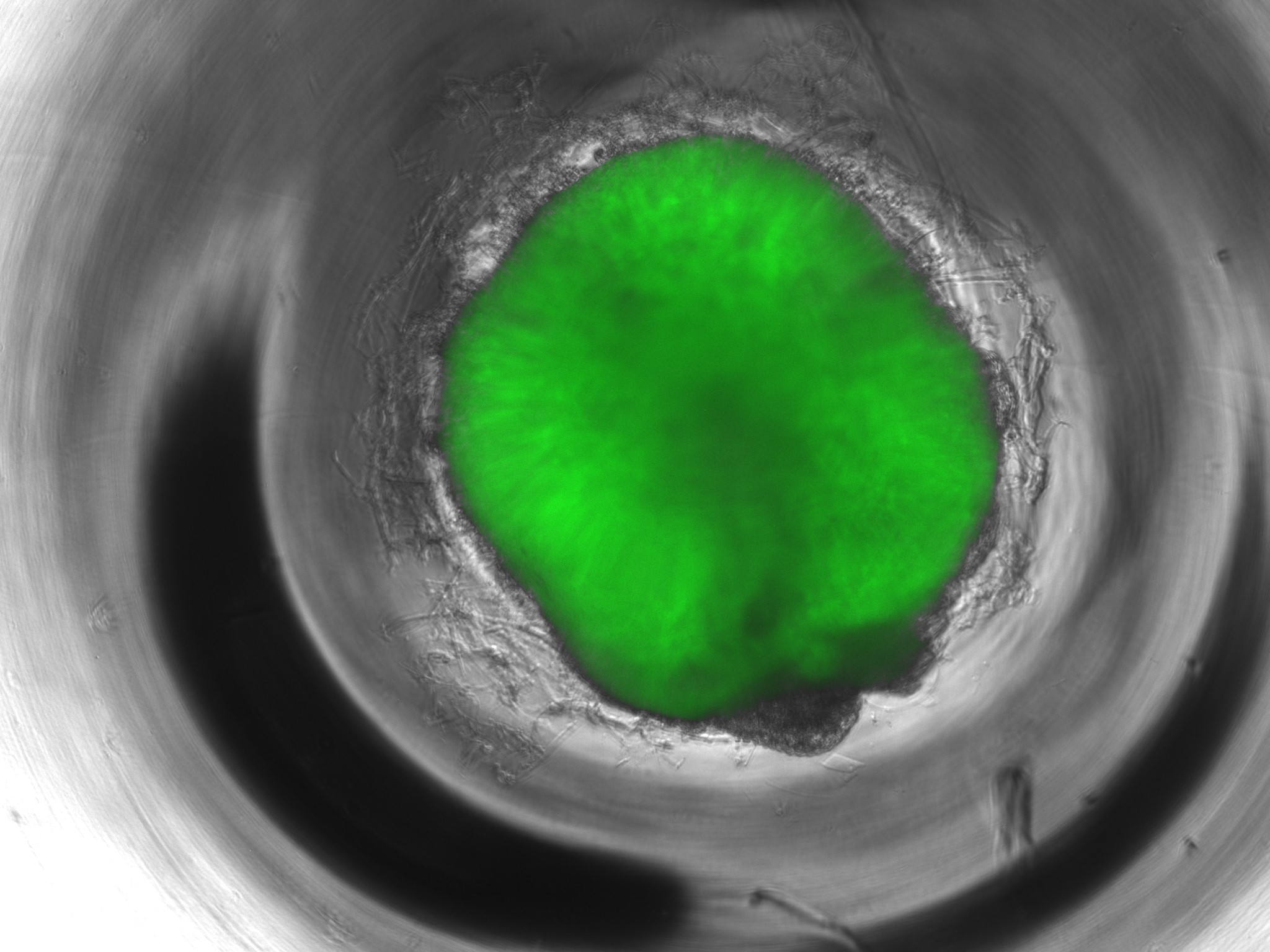

Supplement: Supplementary file 5 — Source data Fig. 2 [file 44318_2025_409_MOESM5_ESM.zip › EMBOJ-2024-118939R-Figure_2_Source_Data-sd/EMBOJ-2024-118939_Fig2B/HAND1_Veh_3.jpg]

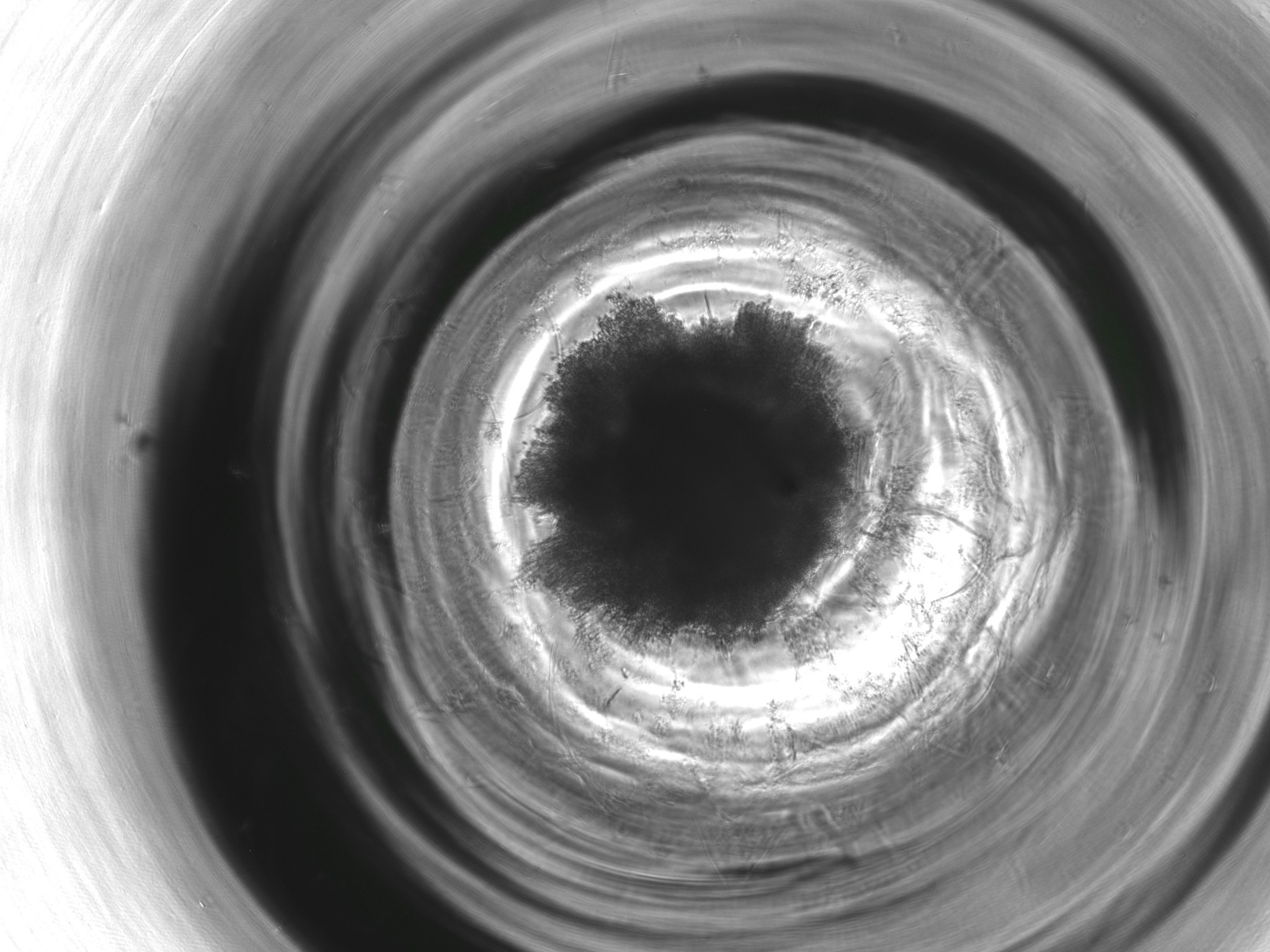

Supplement: Supplementary file 5 — Source data Fig. 2 [file 44318_2025_409_MOESM5_ESM.zip › EMBOJ-2024-118939R-Figure_2_Source_Data-sd/EMBOJ-2024-118939_Fig2B/YAP1_Veh_3.jpg]

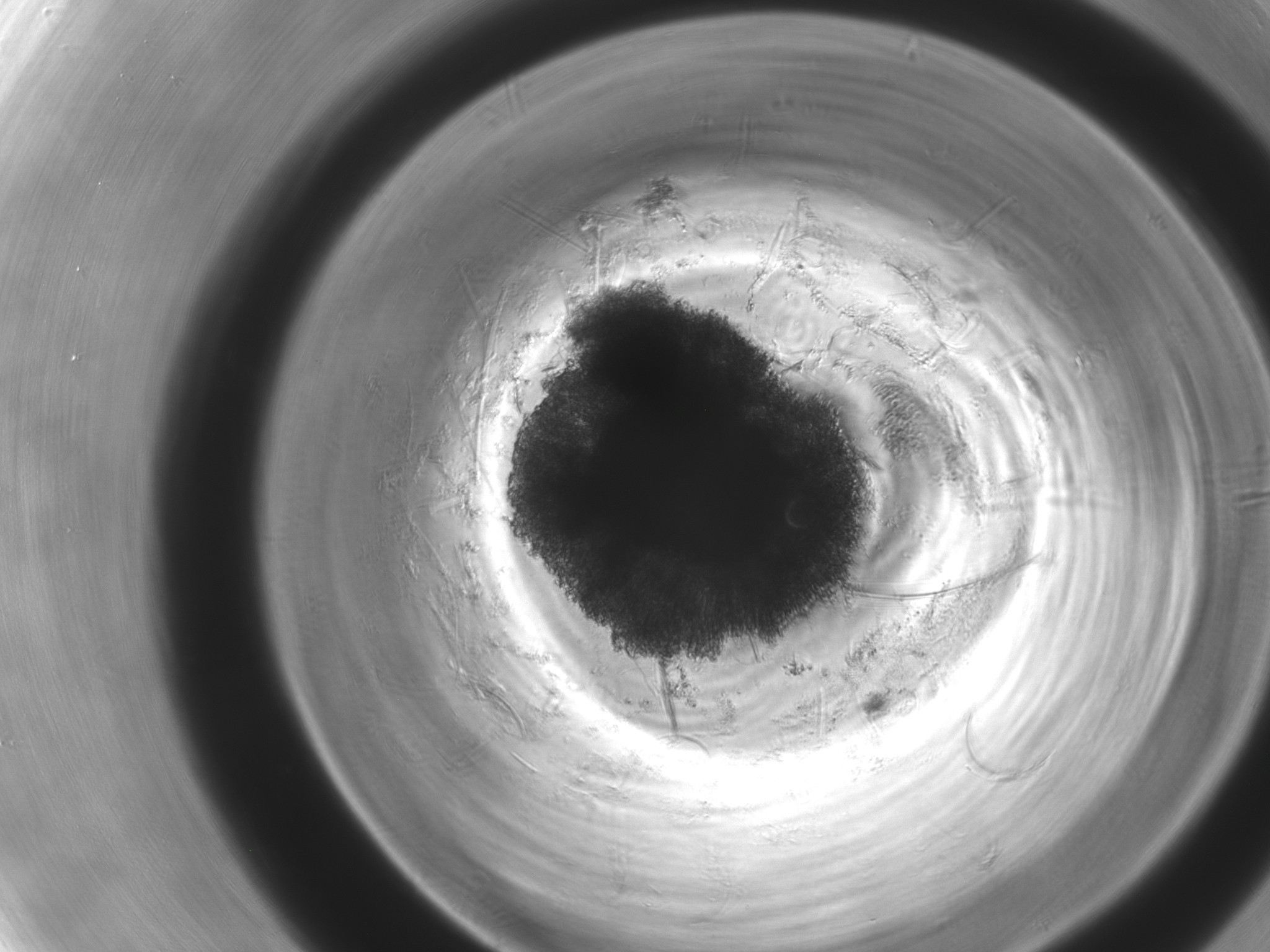

Supplement: Supplementary file 5 — Source data Fig. 2 [file 44318_2025_409_MOESM5_ESM.zip › EMBOJ-2024-118939R-Figure_2_Source_Data-sd/EMBOJ-2024-118939_Fig2B/YAP1_SB_2.jpg]

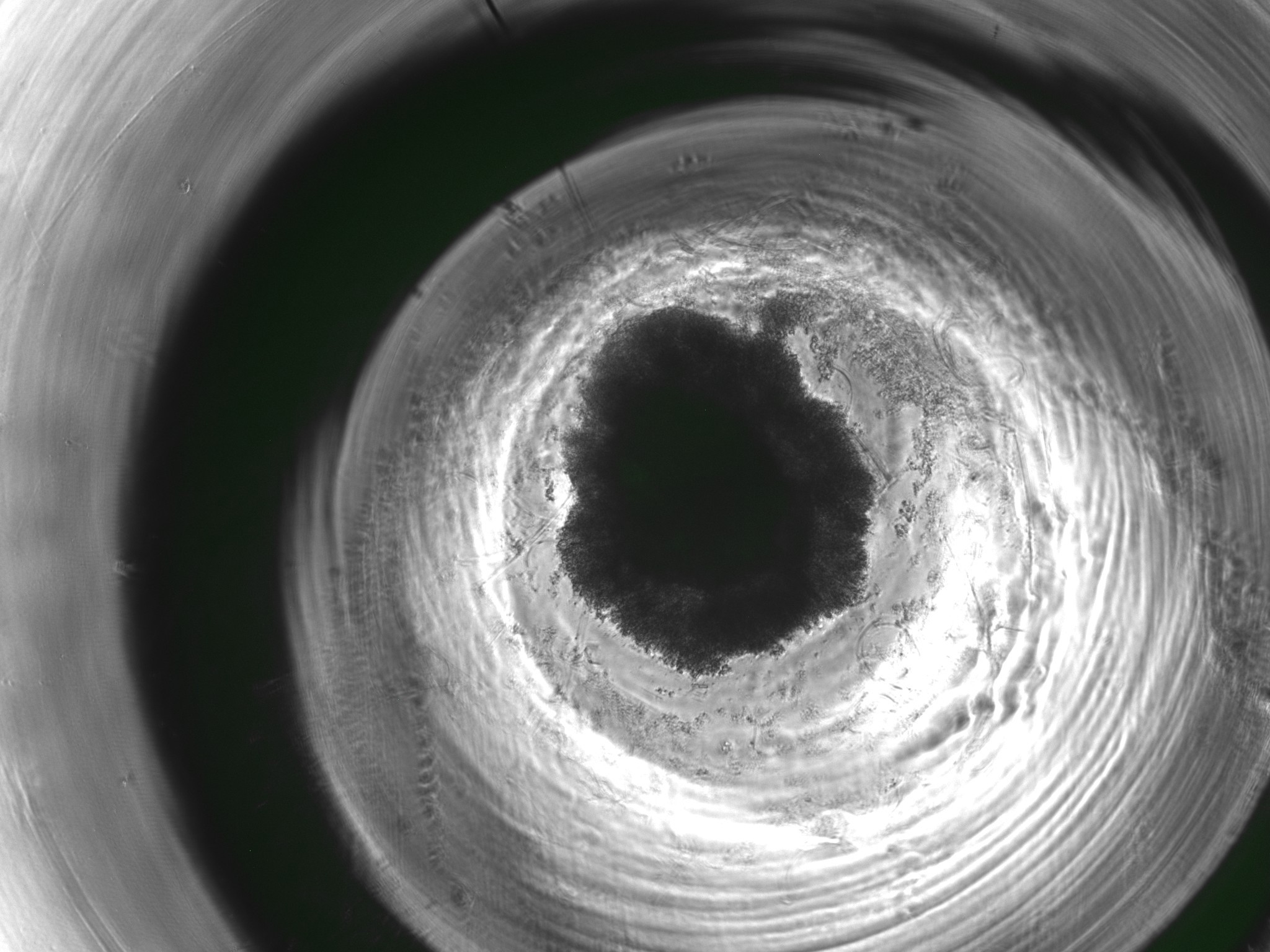

Supplement: Supplementary file 5 — Source data Fig. 2 [file 44318_2025_409_MOESM5_ESM.zip › EMBOJ-2024-118939R-Figure_2_Source_Data-sd/EMBOJ-2024-118939_Fig2B/YAP1_Veh_1.jpg]

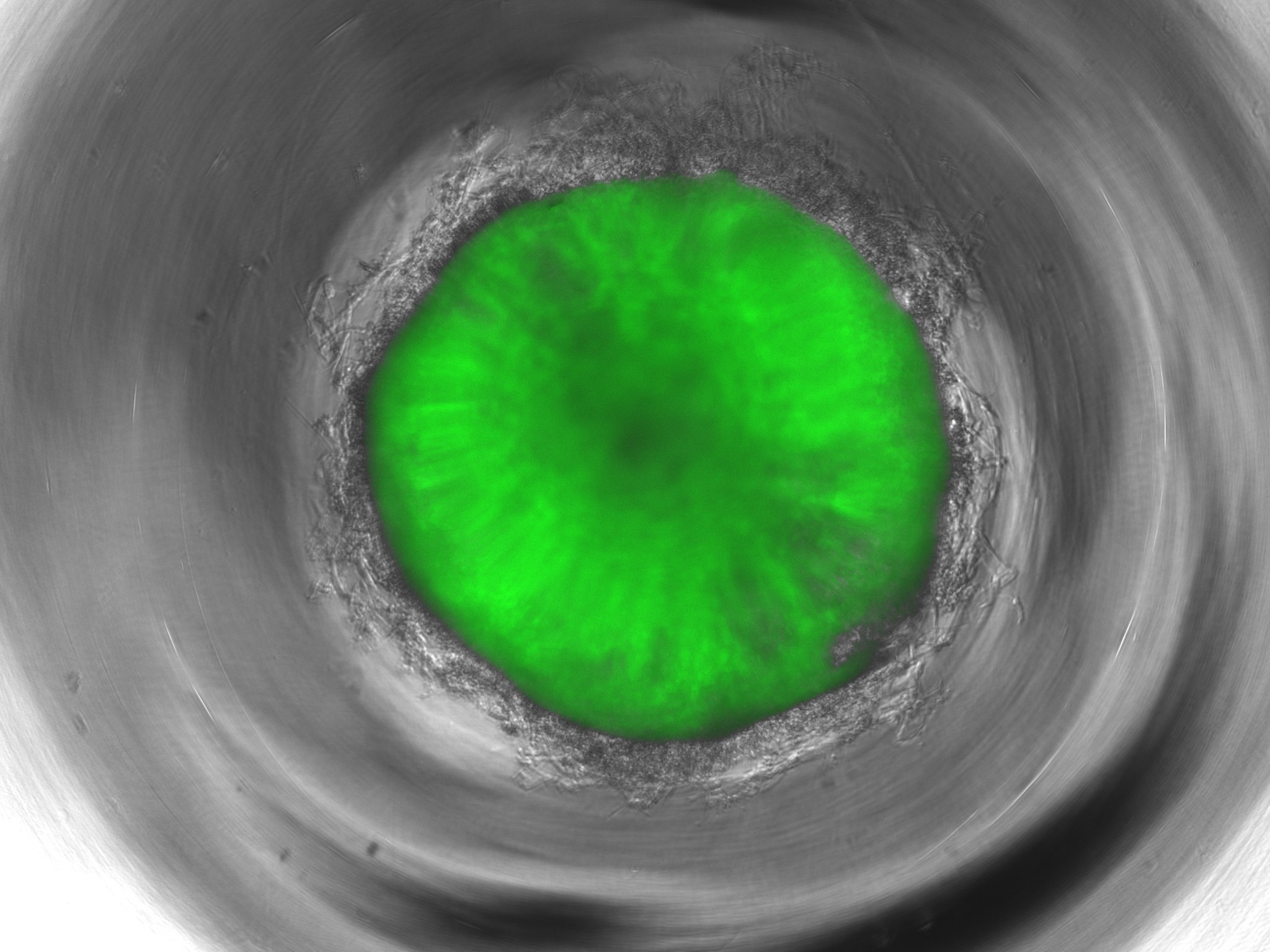

Supplement: Supplementary file 5 — Source data Fig. 2 [file 44318_2025_409_MOESM5_ESM.zip › EMBOJ-2024-118939R-Figure_2_Source_Data-sd/EMBOJ-2024-118939_Fig2B/HAND1_Veh_1.jpg]

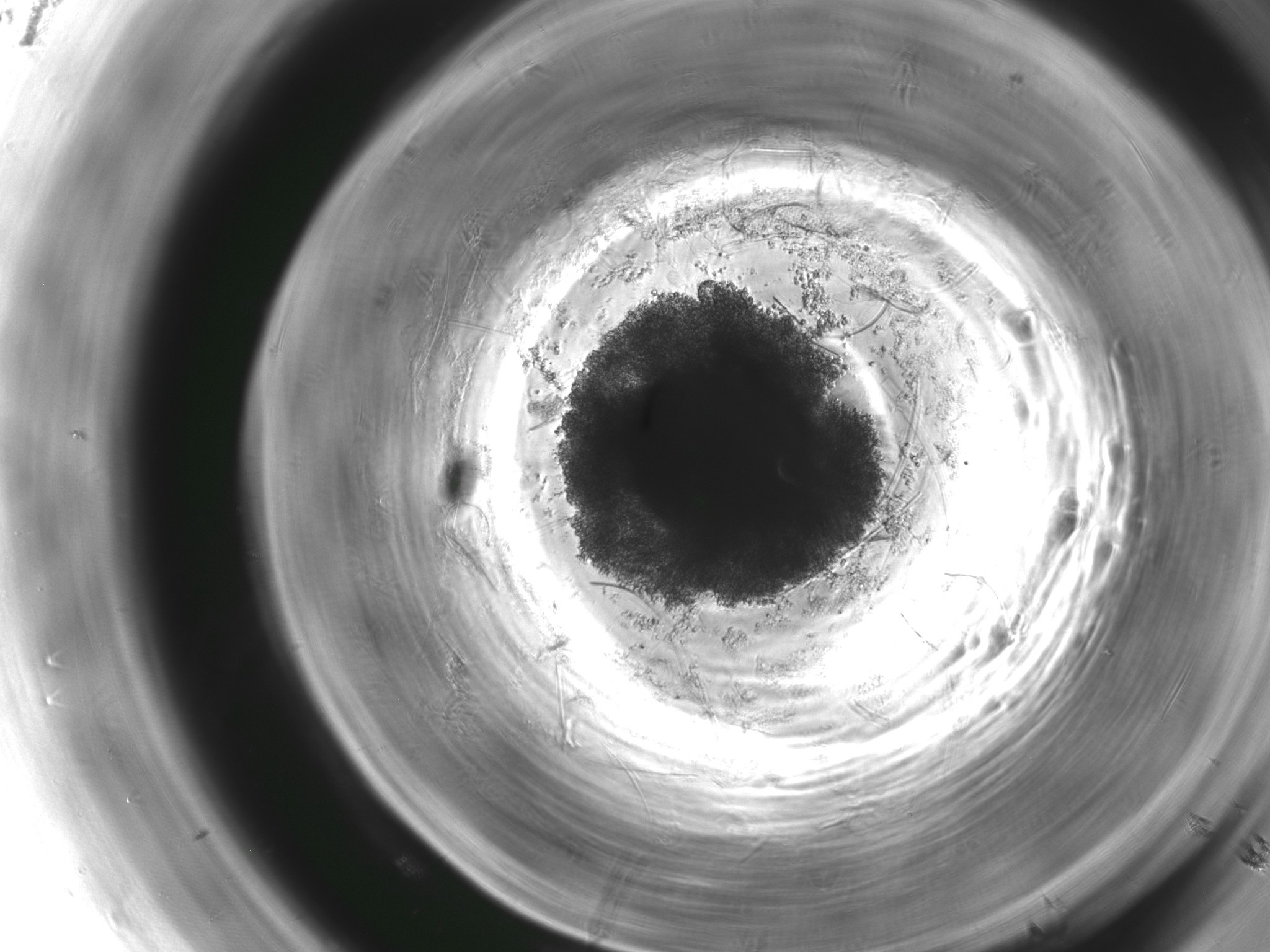

Supplement: Supplementary file 5 — Source data Fig. 2 [file 44318_2025_409_MOESM5_ESM.zip › EMBOJ-2024-118939R-Figure_2_Source_Data-sd/EMBOJ-2024-118939_Fig2B/YAP1_SB_3.jpg]

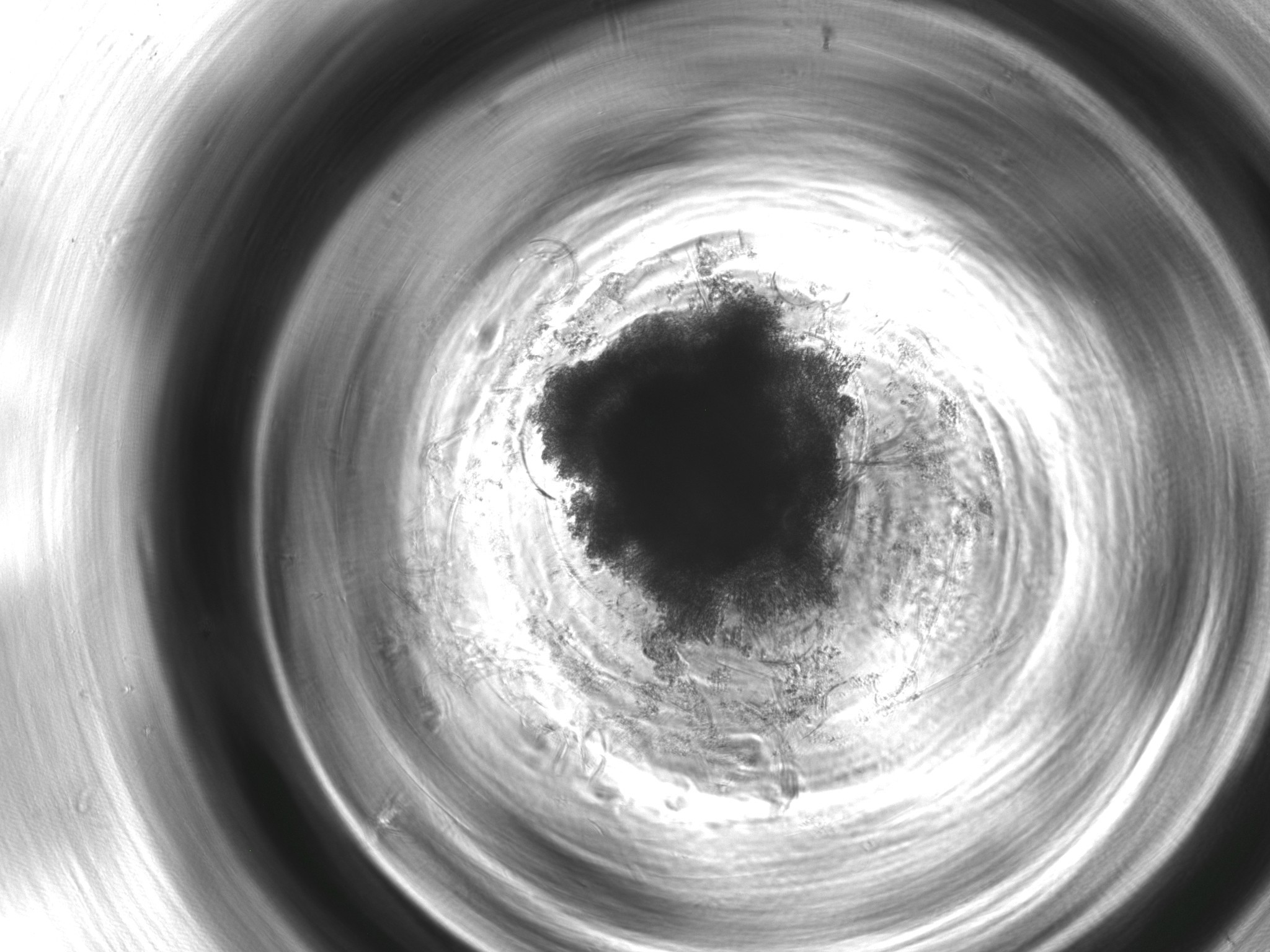

Supplement: Supplementary file 5 — Source data Fig. 2 [file 44318_2025_409_MOESM5_ESM.zip › EMBOJ-2024-118939R-Figure_2_Source_Data-sd/EMBOJ-2024-118939_Fig2B/YAP1_Veh_4.jpg]

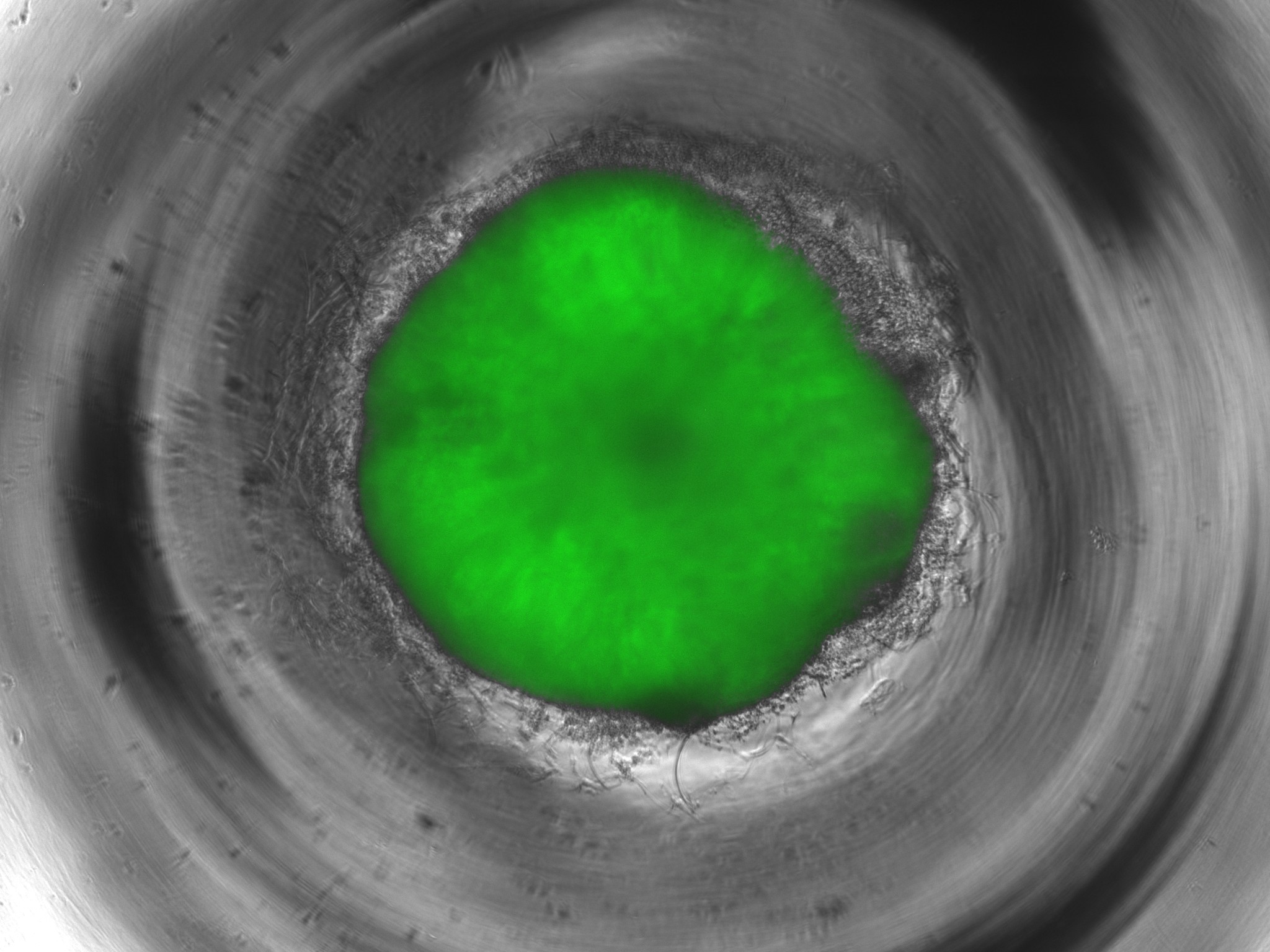

Supplement: Supplementary file 5 — Source data Fig. 2 [file 44318_2025_409_MOESM5_ESM.zip › EMBOJ-2024-118939R-Figure_2_Source_Data-sd/EMBOJ-2024-118939_Fig2B/HAND1_Veh_4.jpg]

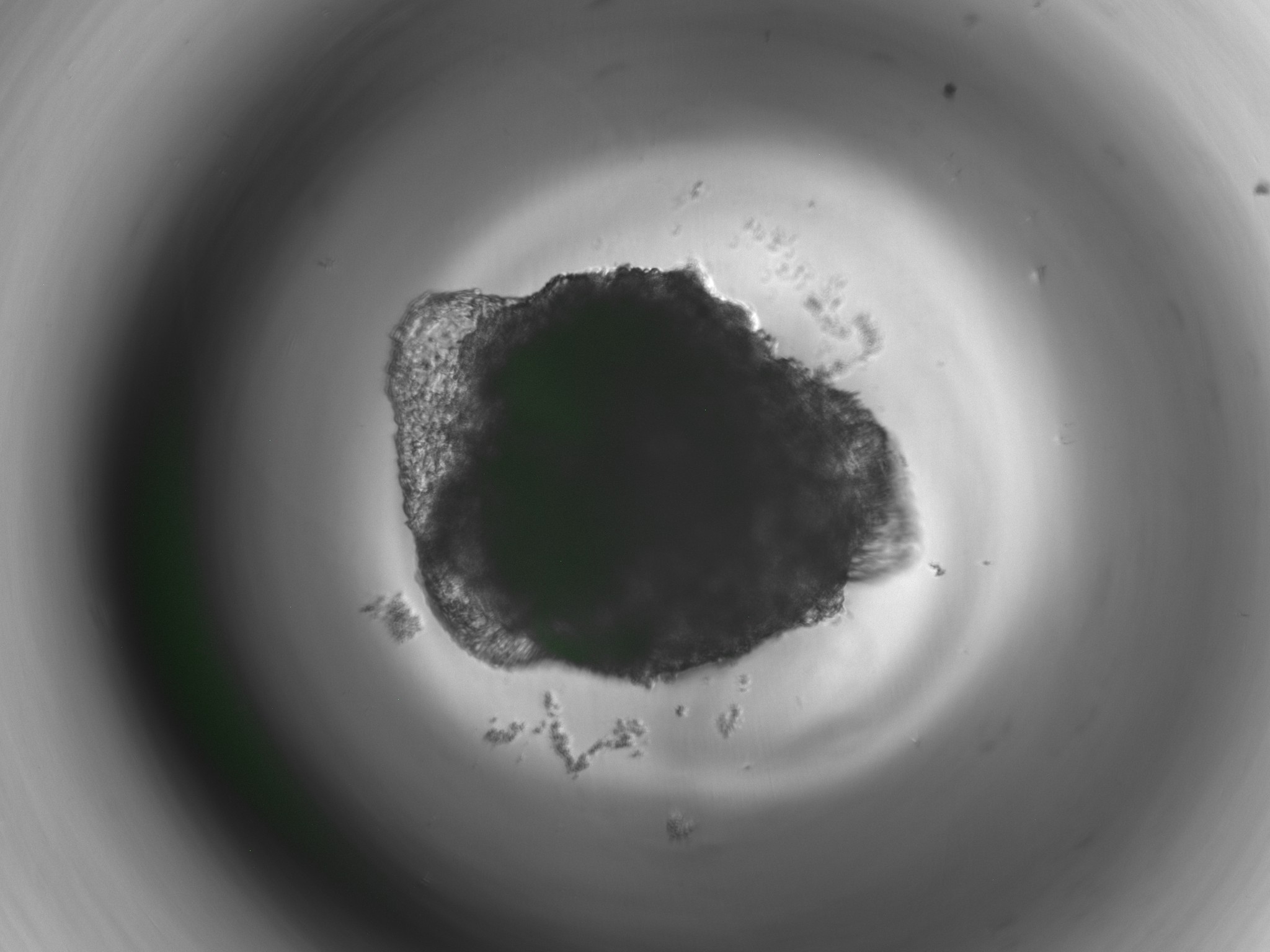

Supplement: Supplementary file 5 — Source data Fig. 2 [file 44318_2025_409_MOESM5_ESM.zip › EMBOJ-2024-118939R-Figure_2_Source_Data-sd/EMBOJ-2024-118939_Fig2B/WT1_SB_2.jpg]

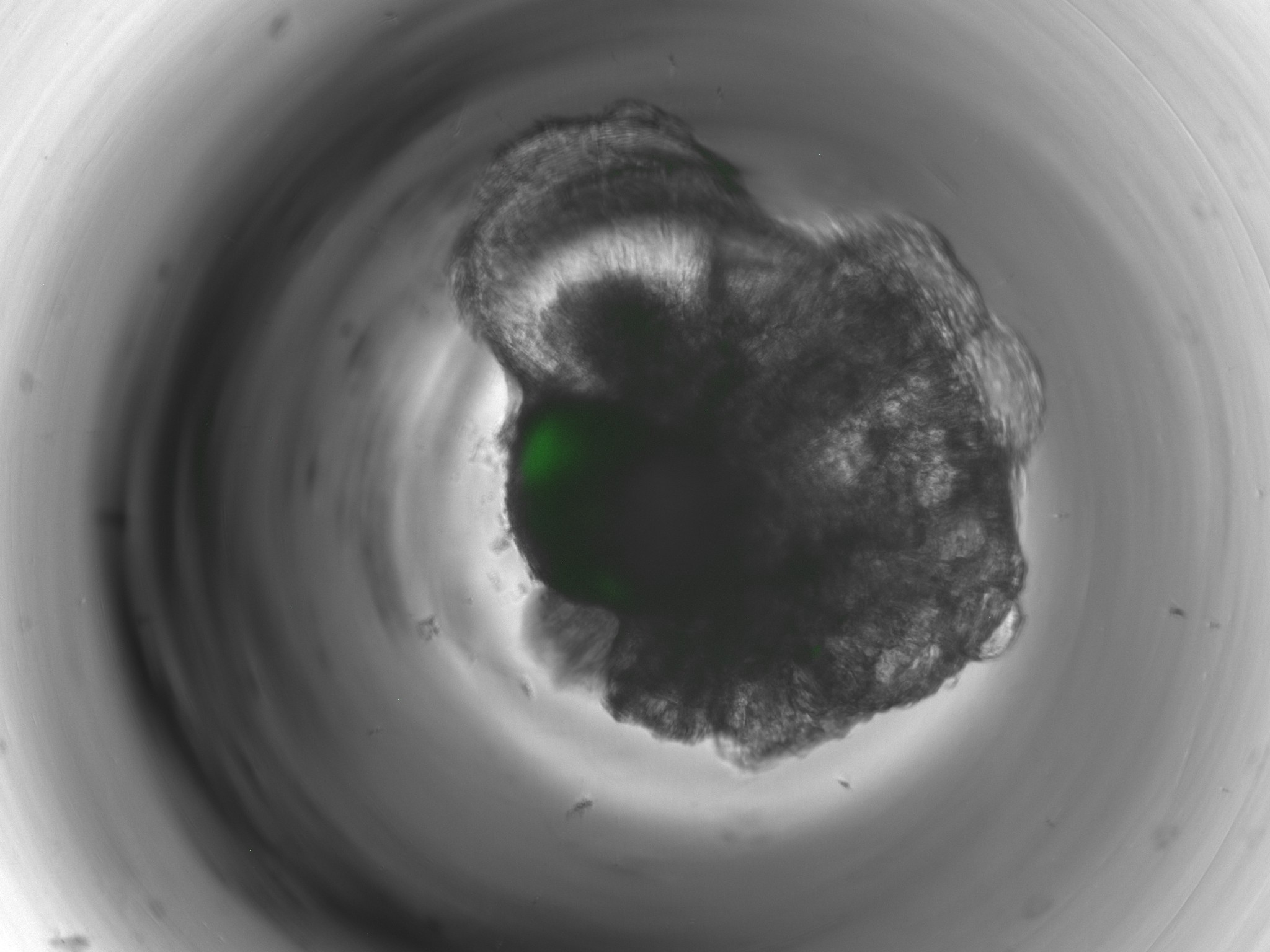

Supplement: Supplementary file 5 — Source data Fig. 2 [file 44318_2025_409_MOESM5_ESM.zip › EMBOJ-2024-118939R-Figure_2_Source_Data-sd/EMBOJ-2024-118939_Fig2B/WT1_SB_3.jpg]

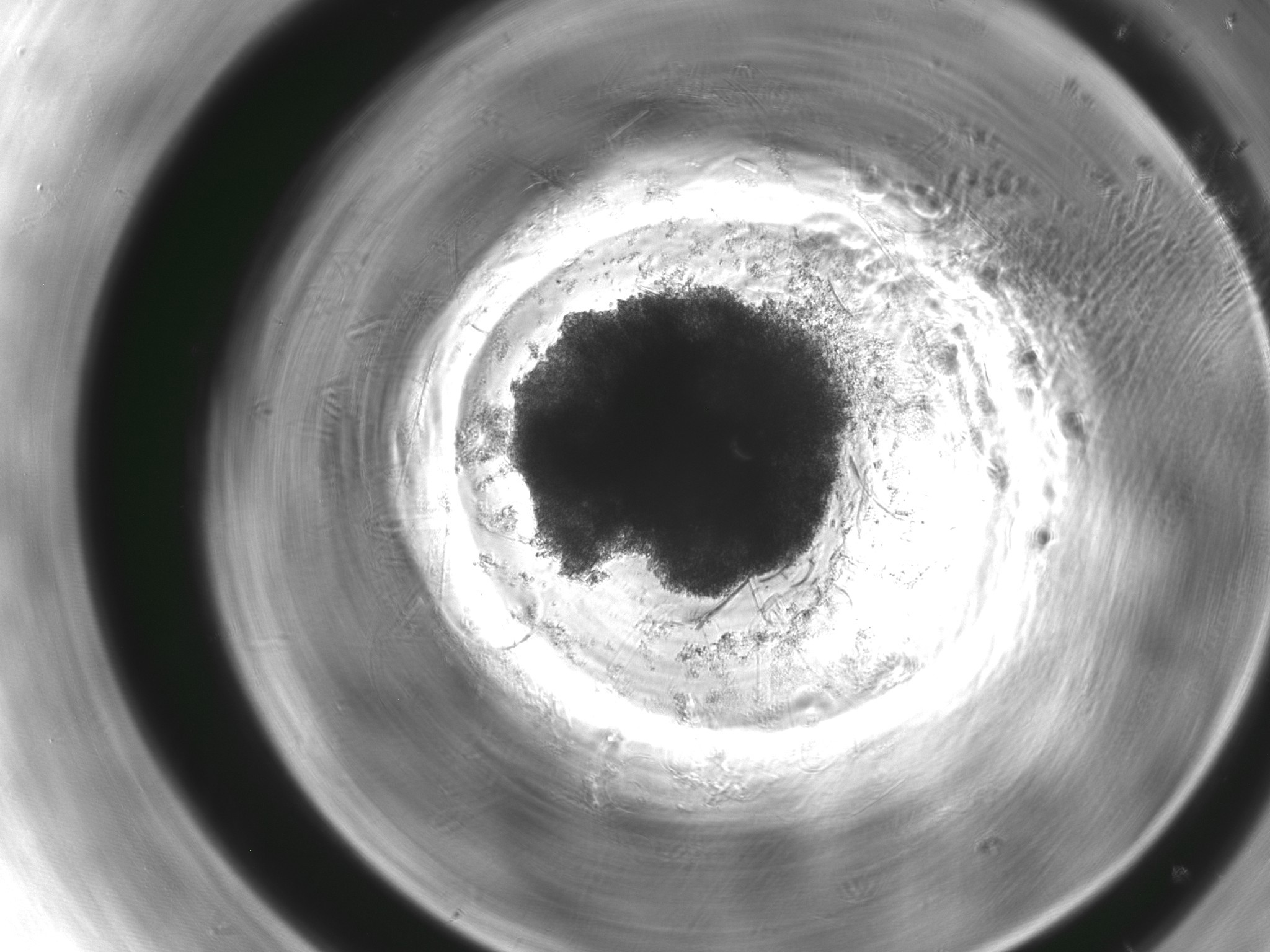

Supplement: Supplementary file 5 — Source data Fig. 2 [file 44318_2025_409_MOESM5_ESM.zip › EMBOJ-2024-118939R-Figure_2_Source_Data-sd/EMBOJ-2024-118939_Fig2B/YAP1_SB_4.jpg]

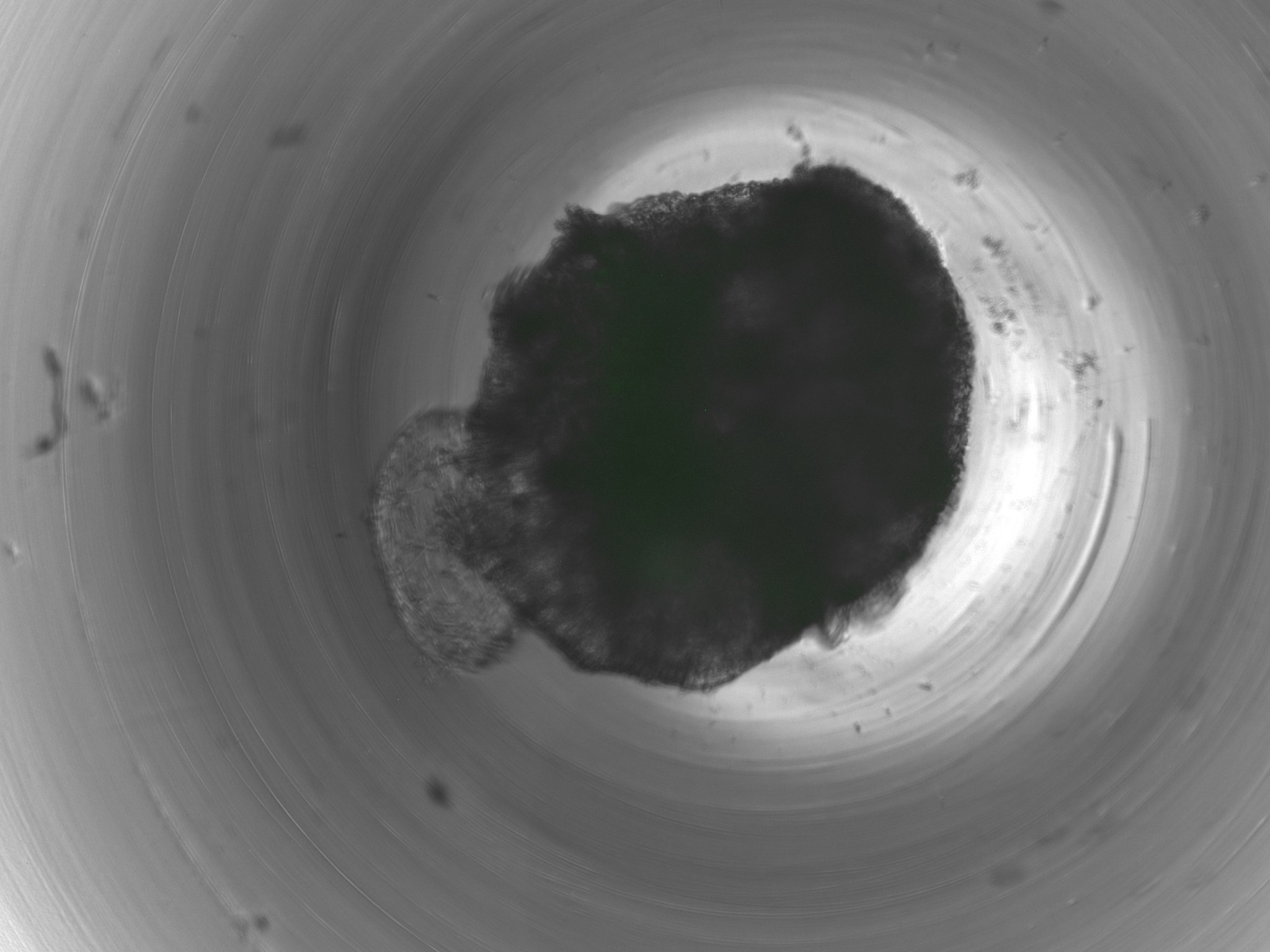

Supplement: Supplementary file 5 — Source data Fig. 2 [file 44318_2025_409_MOESM5_ESM.zip › EMBOJ-2024-118939R-Figure_2_Source_Data-sd/EMBOJ-2024-118939_Fig2B/WT1_SB_1.jpg]

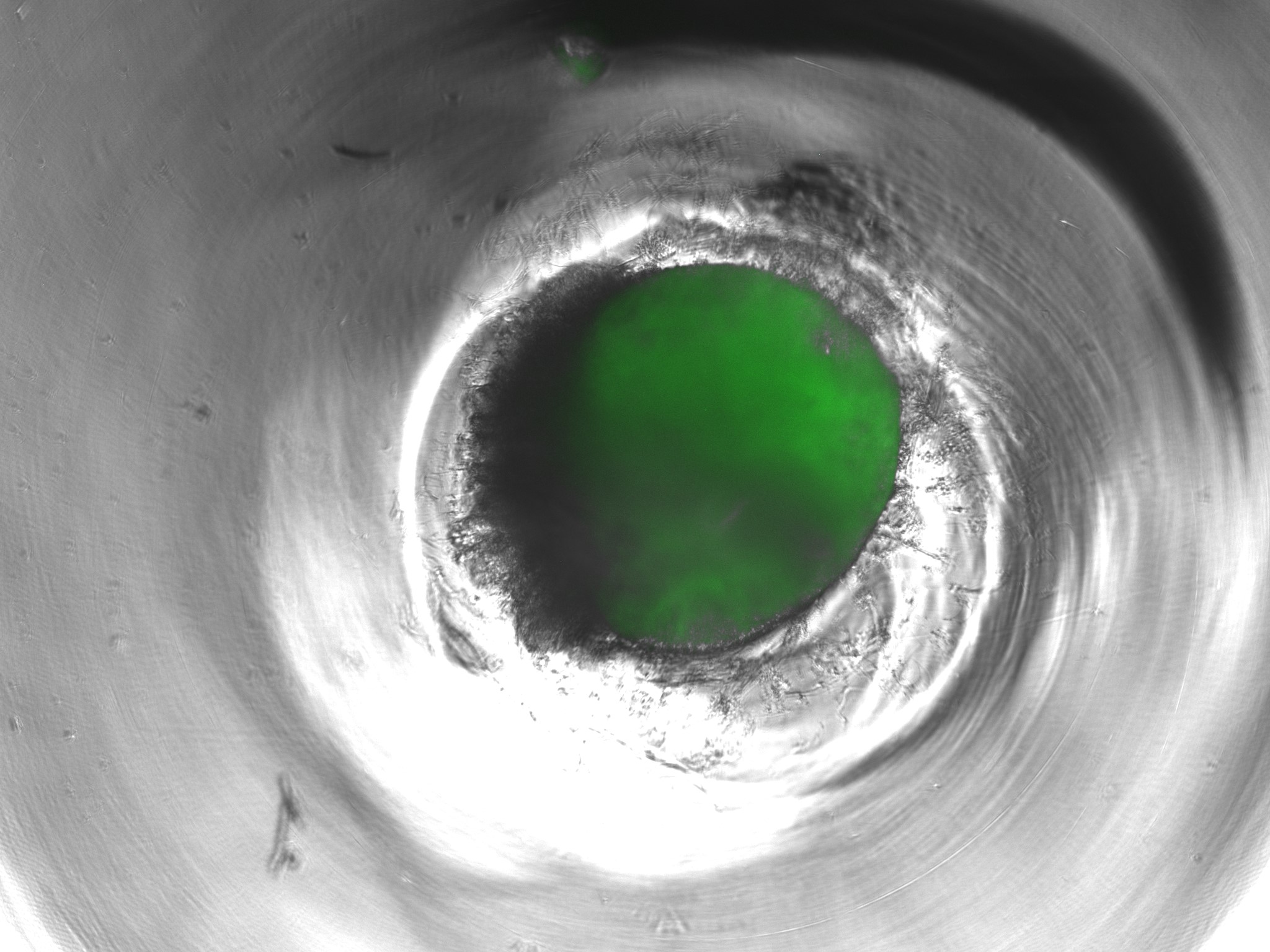

Supplement: Supplementary file 5 — Source data Fig. 2 [file 44318_2025_409_MOESM5_ESM.zip › EMBOJ-2024-118939R-Figure_2_Source_Data-sd/EMBOJ-2024-118939_Fig2B/HOXB_Veh_4.jpg]

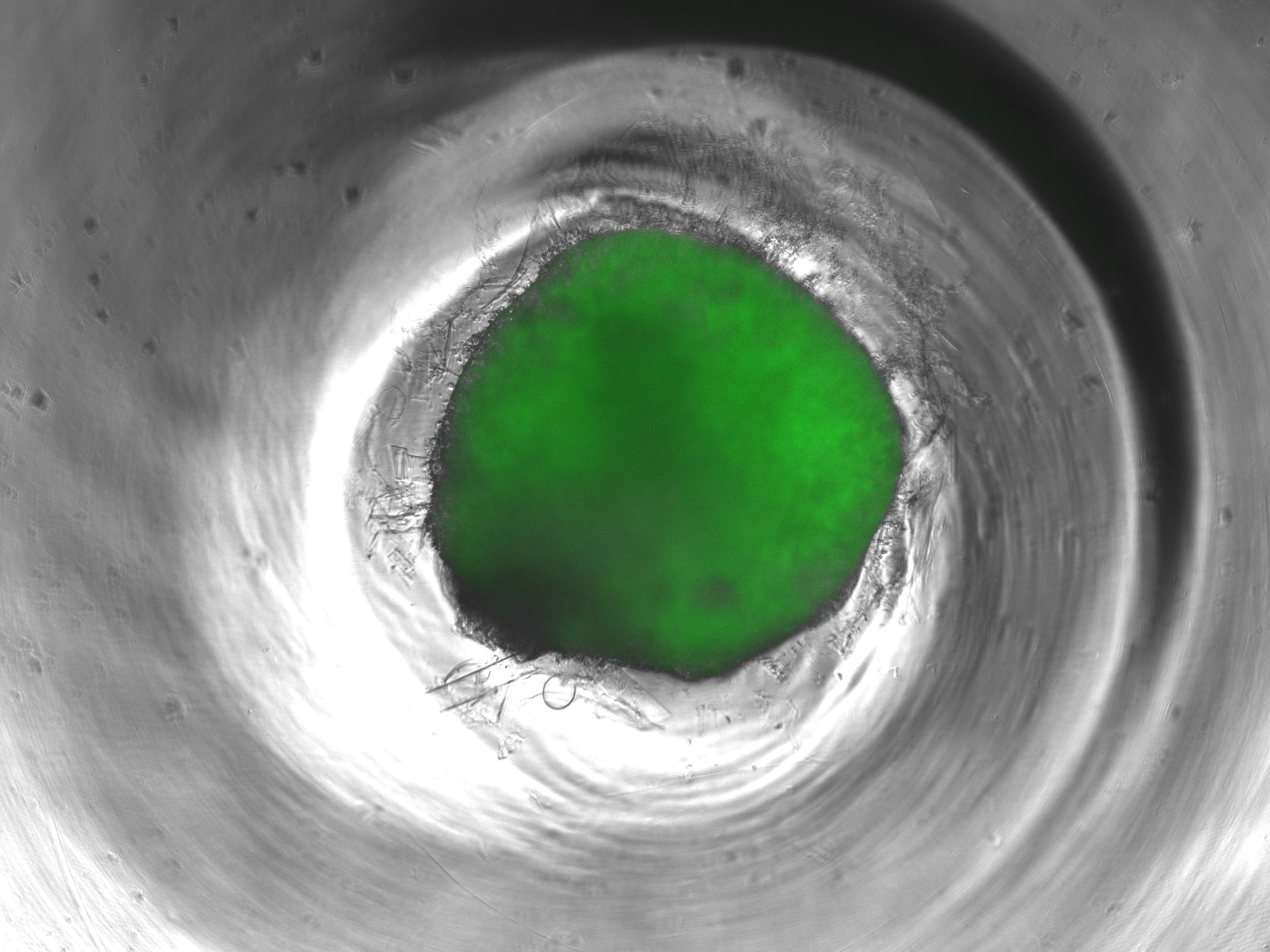

Supplement: Supplementary file 5 — Source data Fig. 2 [file 44318_2025_409_MOESM5_ESM.zip › EMBOJ-2024-118939R-Figure_2_Source_Data-sd/EMBOJ-2024-118939_Fig2B/HOXB_Veh_1.jpg]

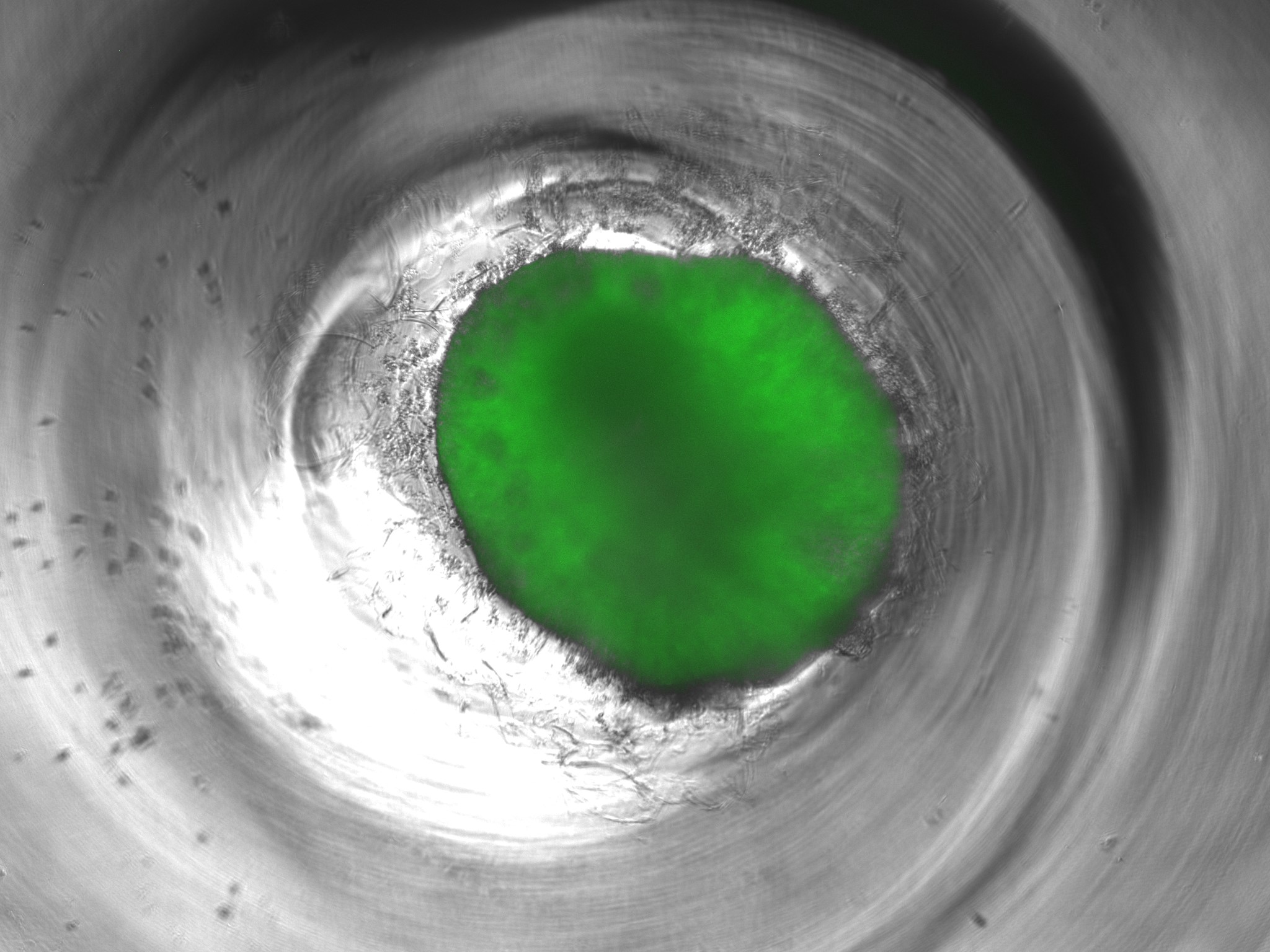

Supplement: Supplementary file 5 — Source data Fig. 2 [file 44318_2025_409_MOESM5_ESM.zip › EMBOJ-2024-118939R-Figure_2_Source_Data-sd/EMBOJ-2024-118939_Fig2B/HOXB_Veh_2.jpg]

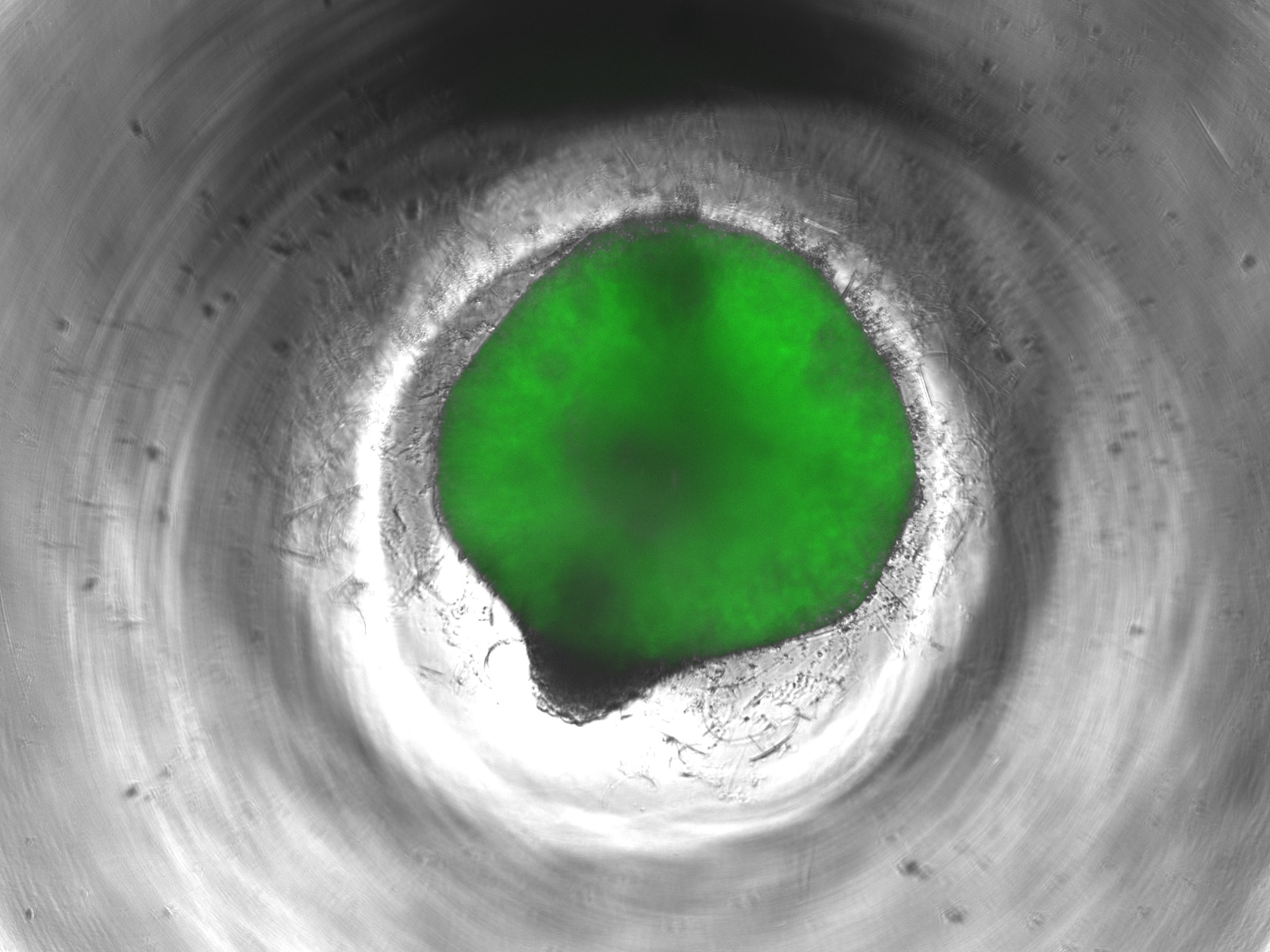

Supplement: Supplementary file 5 — Source data Fig. 2 [file 44318_2025_409_MOESM5_ESM.zip › EMBOJ-2024-118939R-Figure_2_Source_Data-sd/EMBOJ-2024-118939_Fig2B/HOXB_Veh_3.jpg]

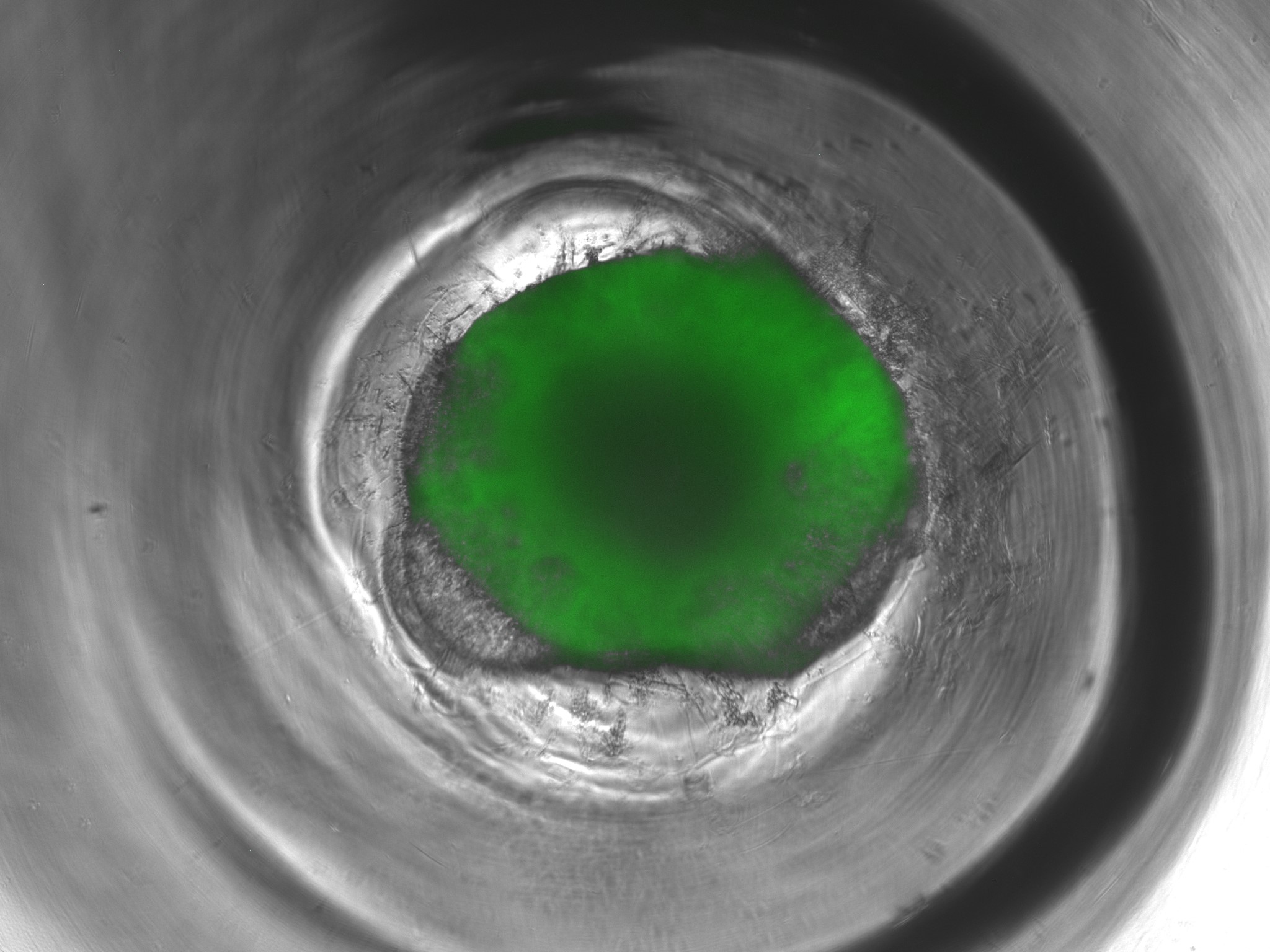

Supplement: Supplementary file 5 — Source data Fig. 2 [file 44318_2025_409_MOESM5_ESM.zip › EMBOJ-2024-118939R-Figure_2_Source_Data-sd/EMBOJ-2024-118939_Fig2B/WT_Veh_2.jpg]

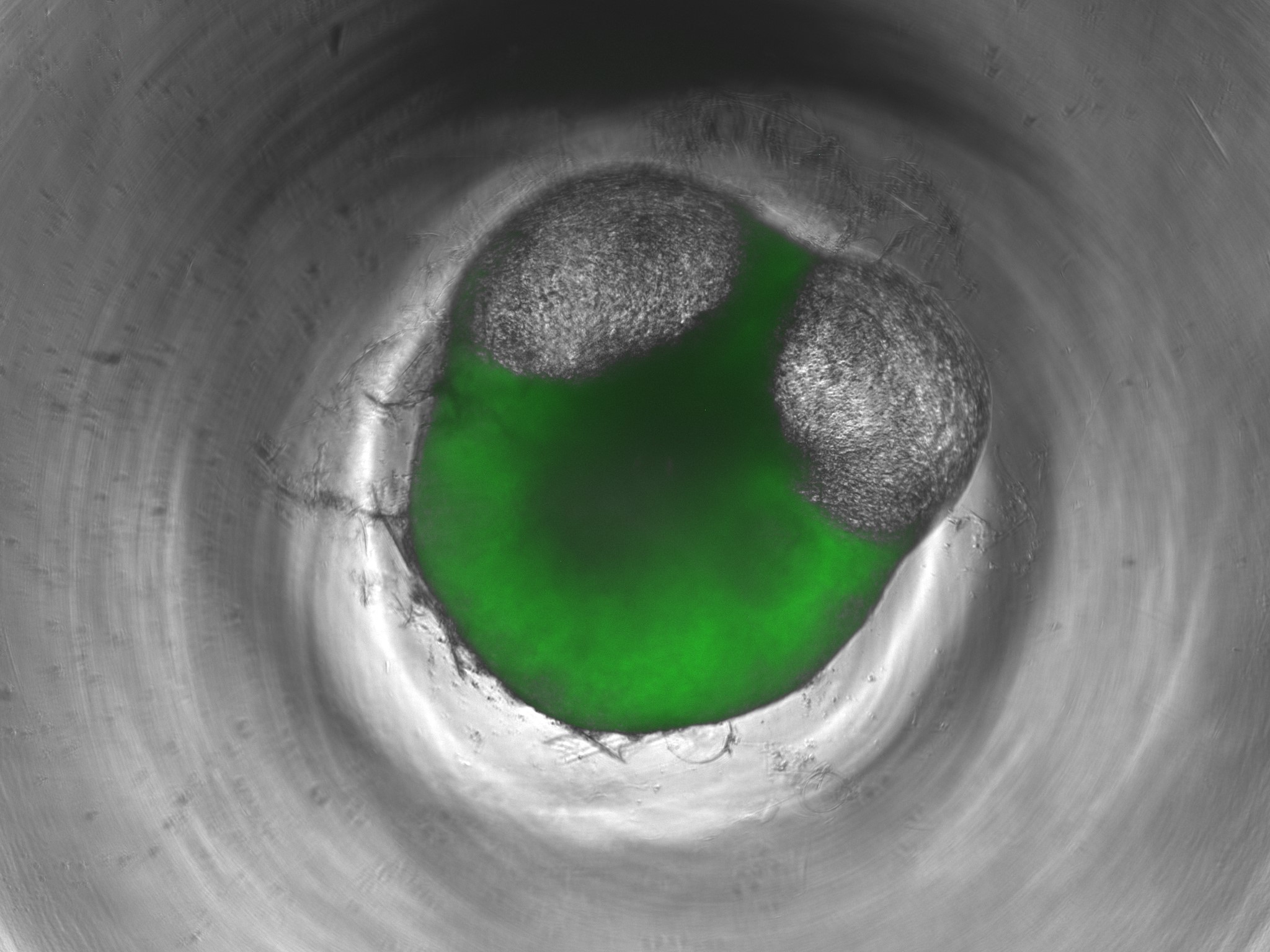

Supplement: Supplementary file 5 — Source data Fig. 2 [file 44318_2025_409_MOESM5_ESM.zip › EMBOJ-2024-118939R-Figure_2_Source_Data-sd/EMBOJ-2024-118939_Fig2B/WT_Veh_3.jpg]

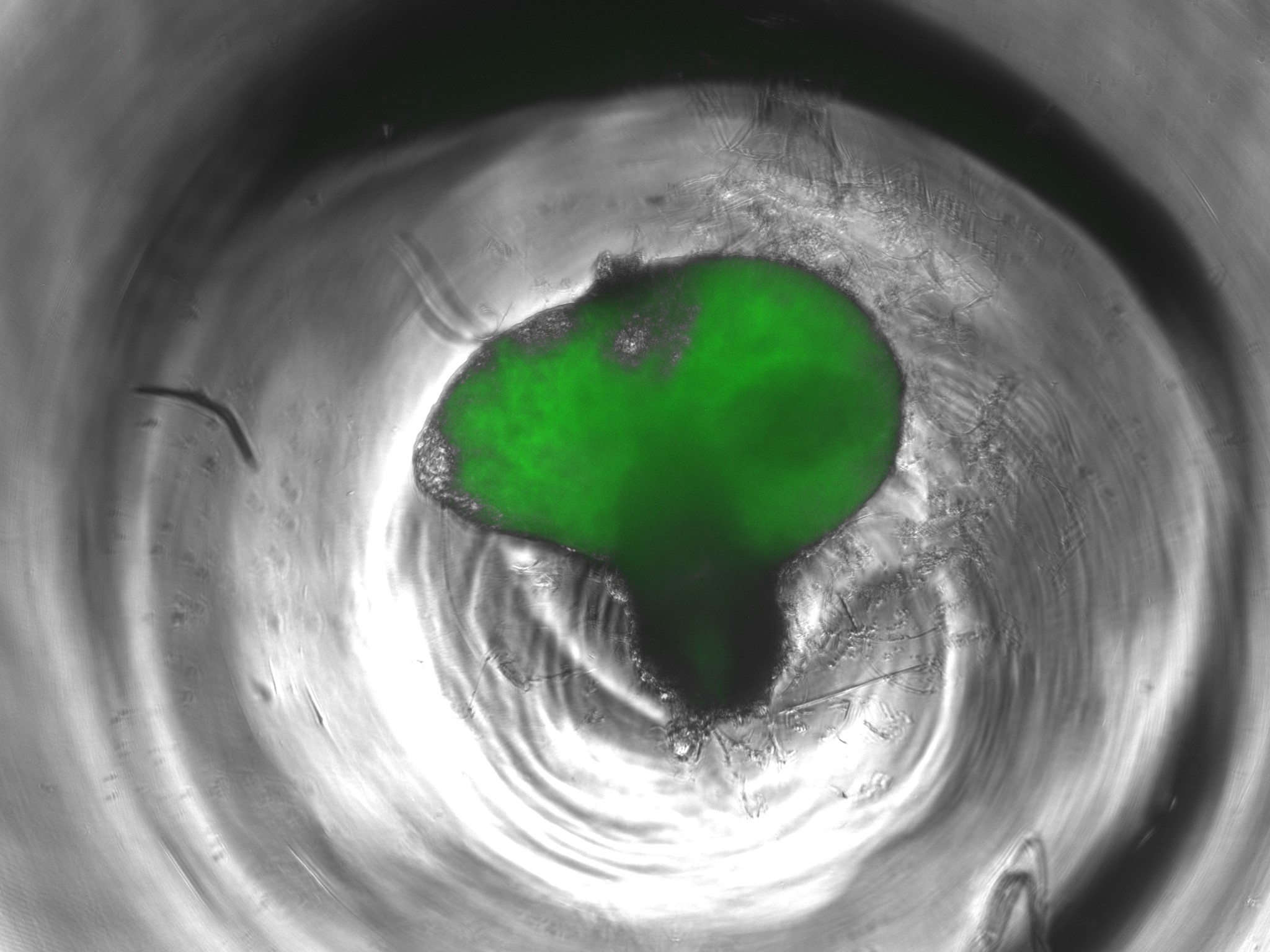

Supplement: Supplementary file 5 — Source data Fig. 2 [file 44318_2025_409_MOESM5_ESM.zip › EMBOJ-2024-118939R-Figure_2_Source_Data-sd/EMBOJ-2024-118939_Fig2B/HOXB_SB_1.jpg]

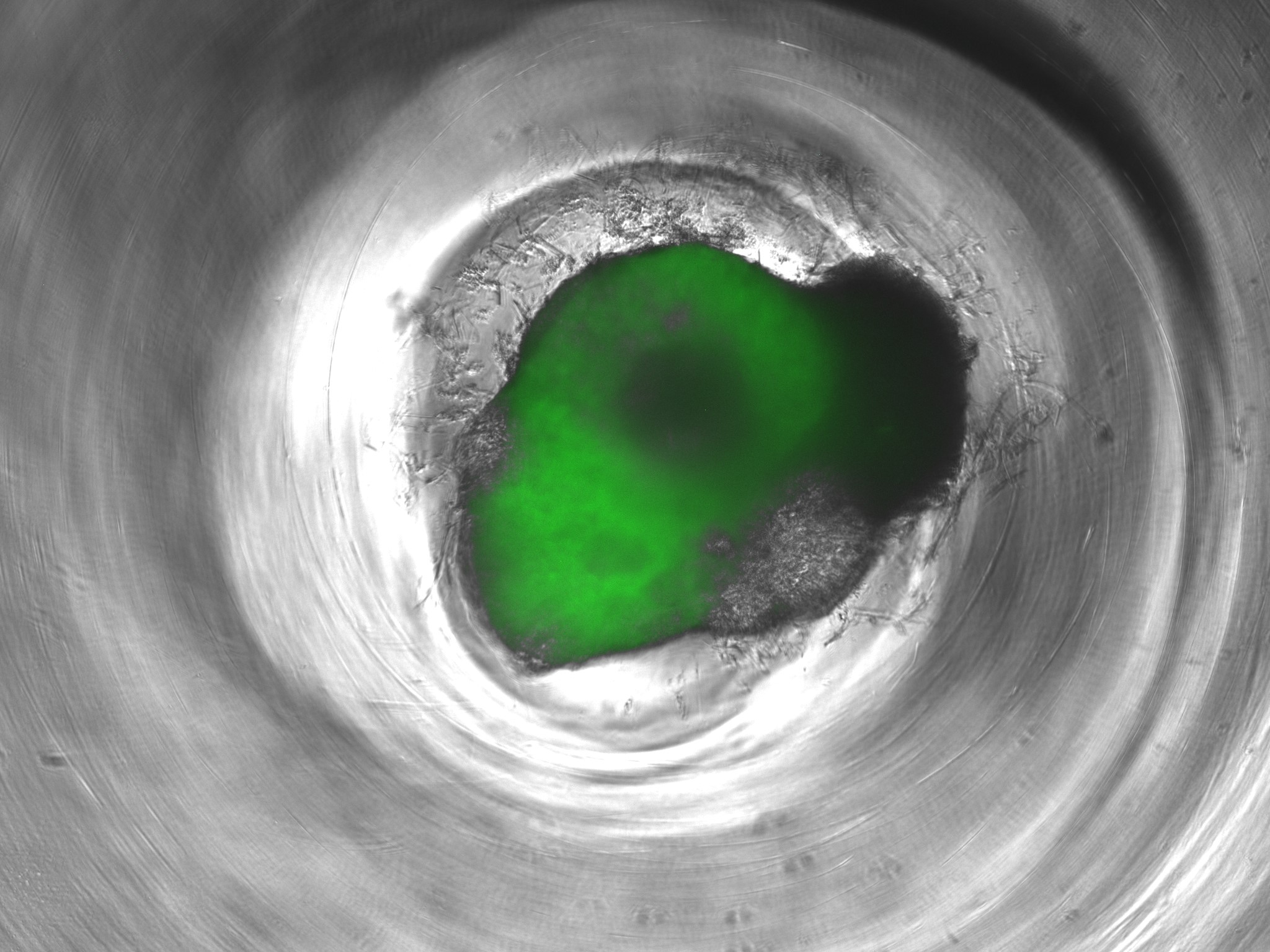

Supplement: Supplementary file 5 — Source data Fig. 2 [file 44318_2025_409_MOESM5_ESM.zip › EMBOJ-2024-118939R-Figure_2_Source_Data-sd/EMBOJ-2024-118939_Fig2B/HOXB_SB_3.jpg]

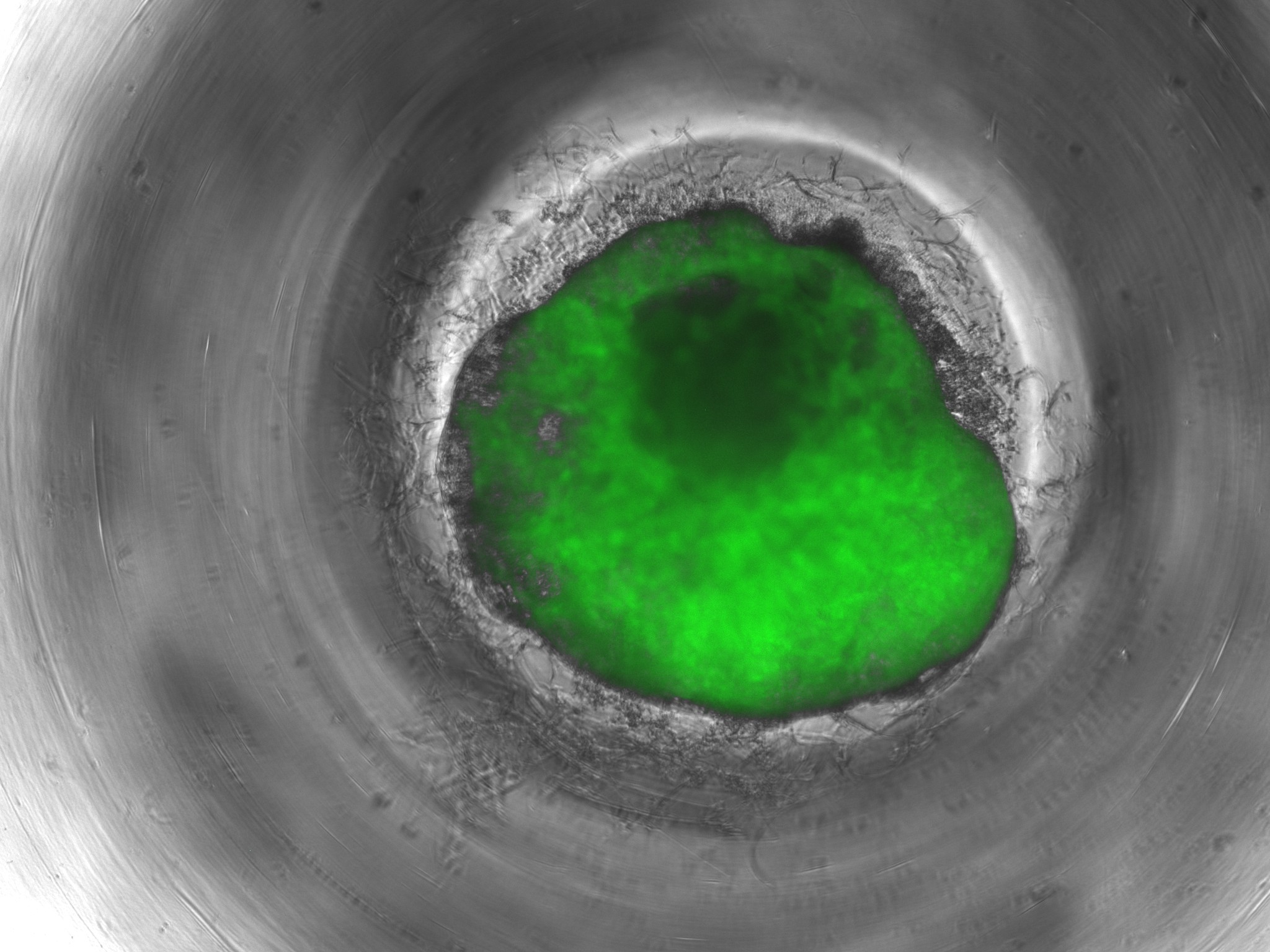

Supplement: Supplementary file 5 — Source data Fig. 2 [file 44318_2025_409_MOESM5_ESM.zip › EMBOJ-2024-118939R-Figure_2_Source_Data-sd/EMBOJ-2024-118939_Fig2B/HAND1_SB_4.jpg]

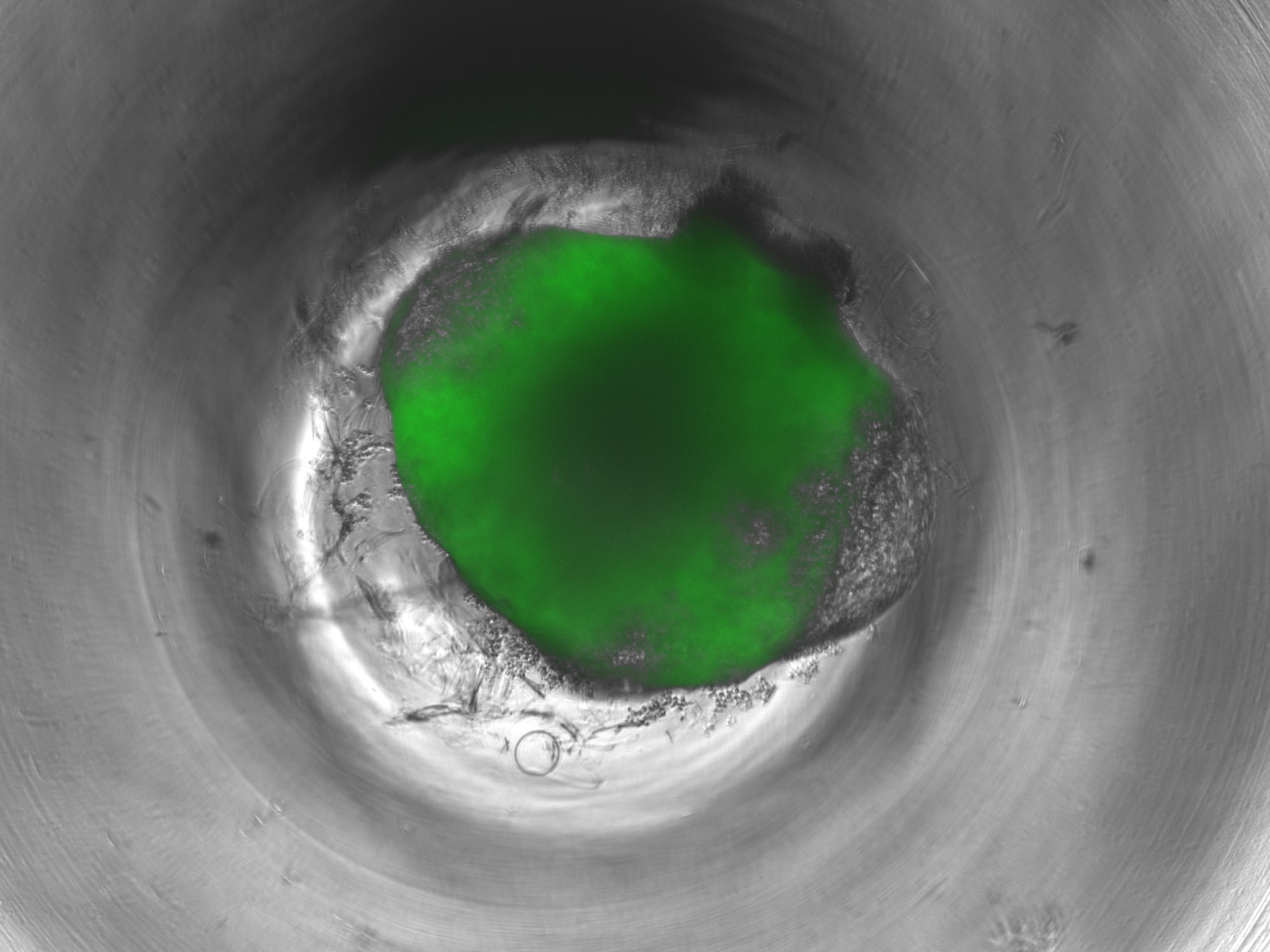

Supplement: Supplementary file 5 — Source data Fig. 2 [file 44318_2025_409_MOESM5_ESM.zip › EMBOJ-2024-118939R-Figure_2_Source_Data-sd/EMBOJ-2024-118939_Fig2B/WT_Veh_1.jpg]

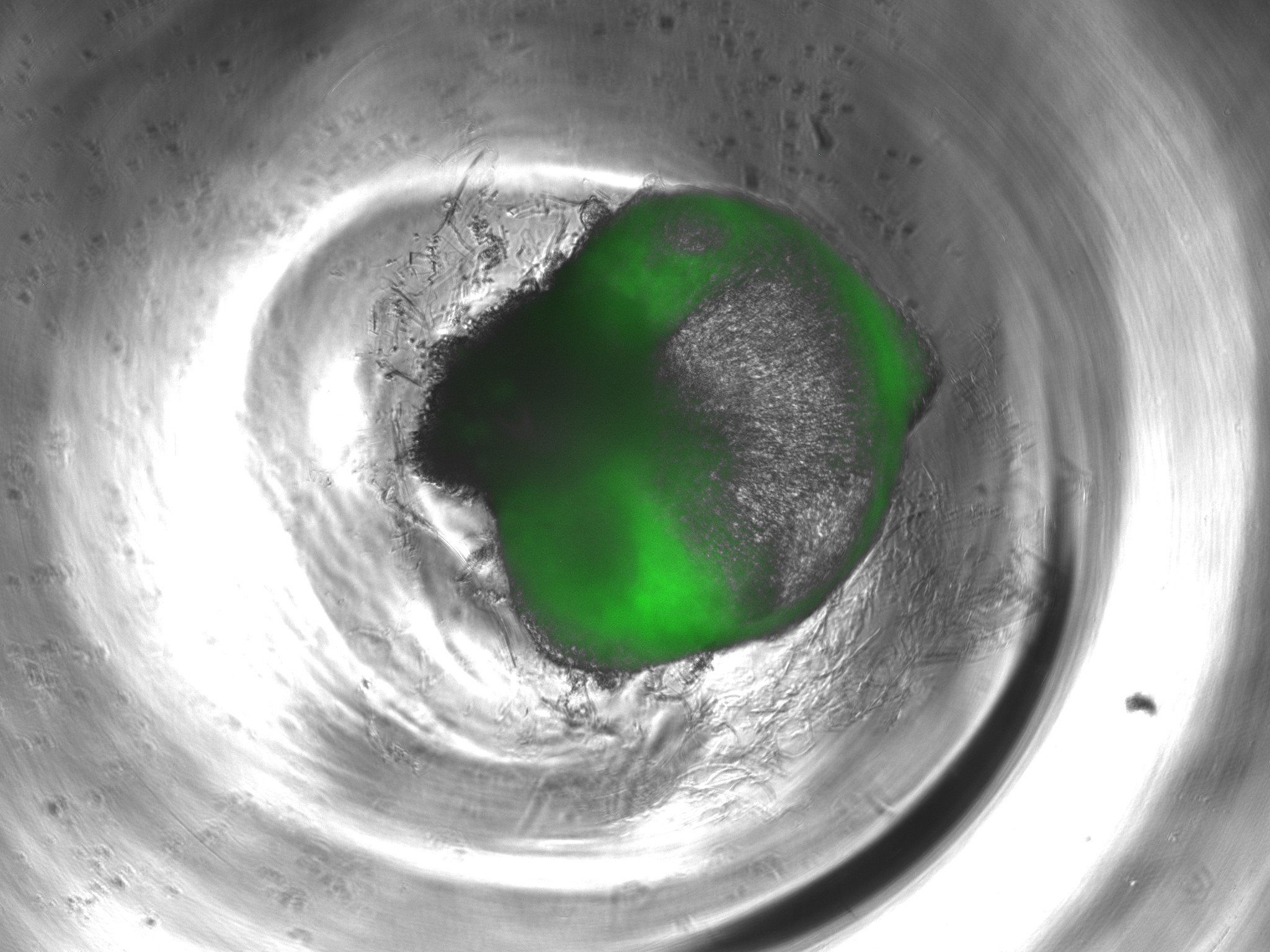

Supplement: Supplementary file 5 — Source data Fig. 2 [file 44318_2025_409_MOESM5_ESM.zip › EMBOJ-2024-118939R-Figure_2_Source_Data-sd/EMBOJ-2024-118939_Fig2B/HOXB_SB_2.jpg]

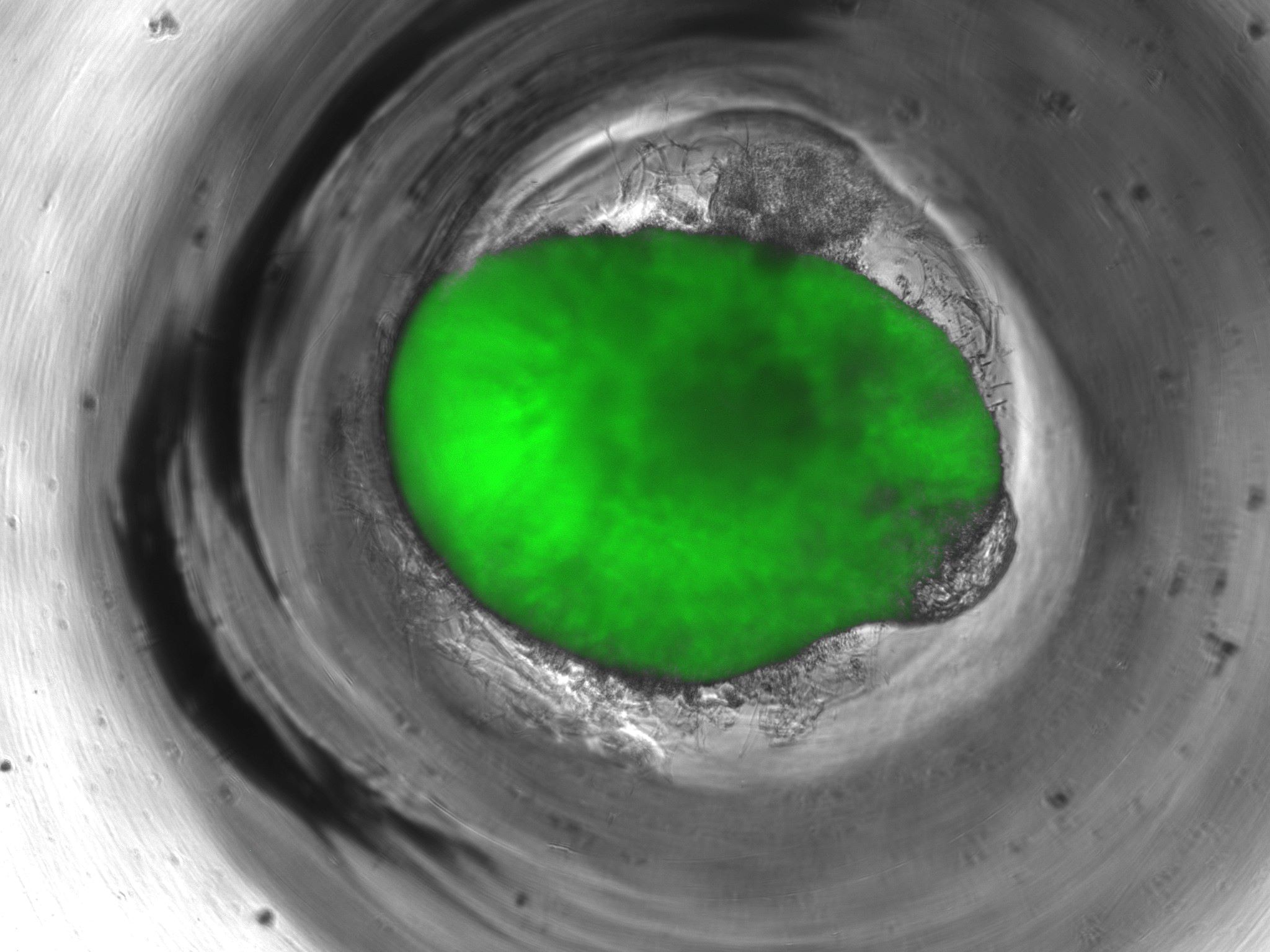

Supplement: Supplementary file 5 — Source data Fig. 2 [file 44318_2025_409_MOESM5_ESM.zip › EMBOJ-2024-118939R-Figure_2_Source_Data-sd/EMBOJ-2024-118939_Fig2B/HAND1_SB_1.jpg]

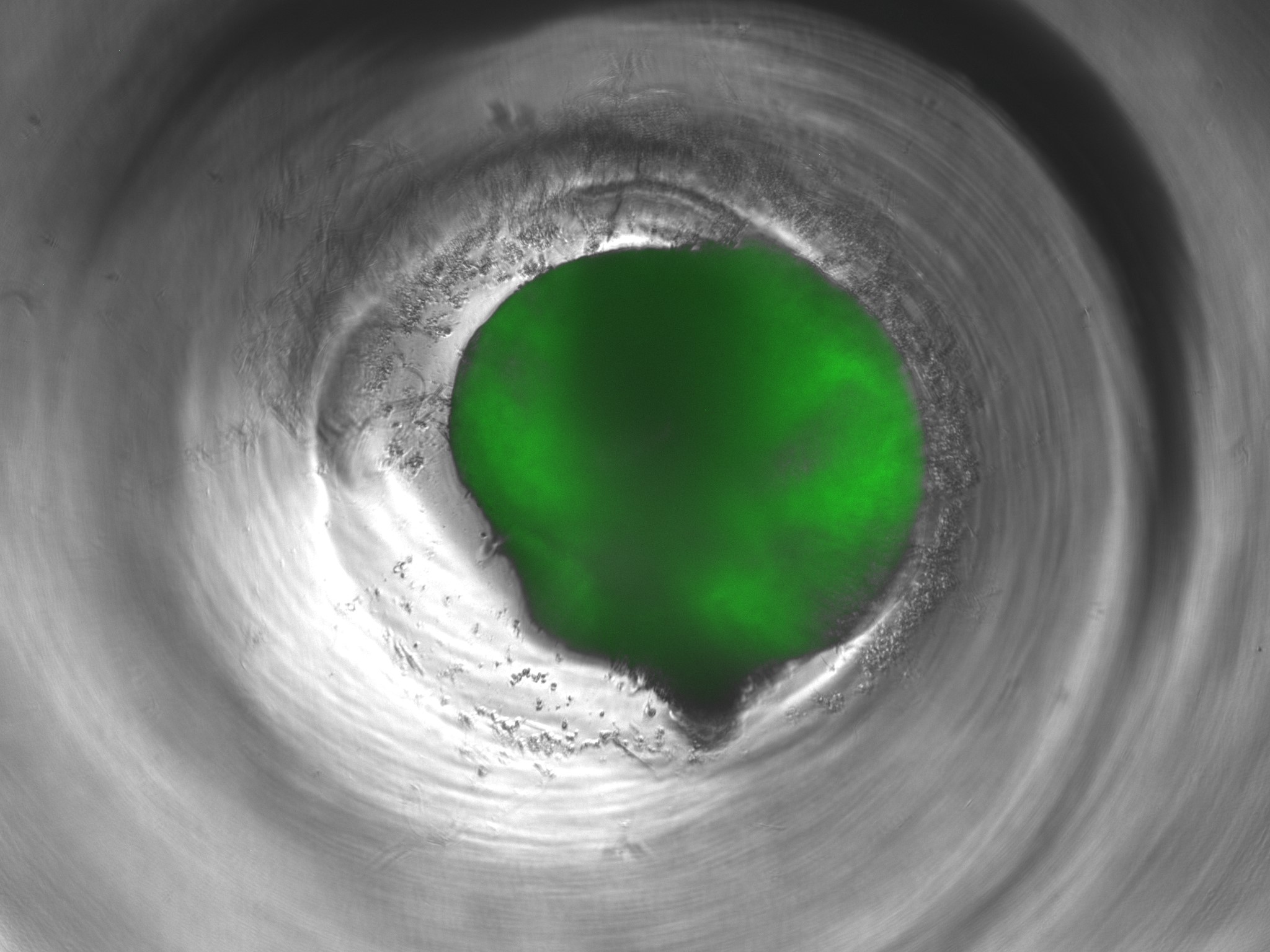

Supplement: Supplementary file 5 — Source data Fig. 2 [file 44318_2025_409_MOESM5_ESM.zip › EMBOJ-2024-118939R-Figure_2_Source_Data-sd/EMBOJ-2024-118939_Fig2B/WT_Veh_4.jpg]

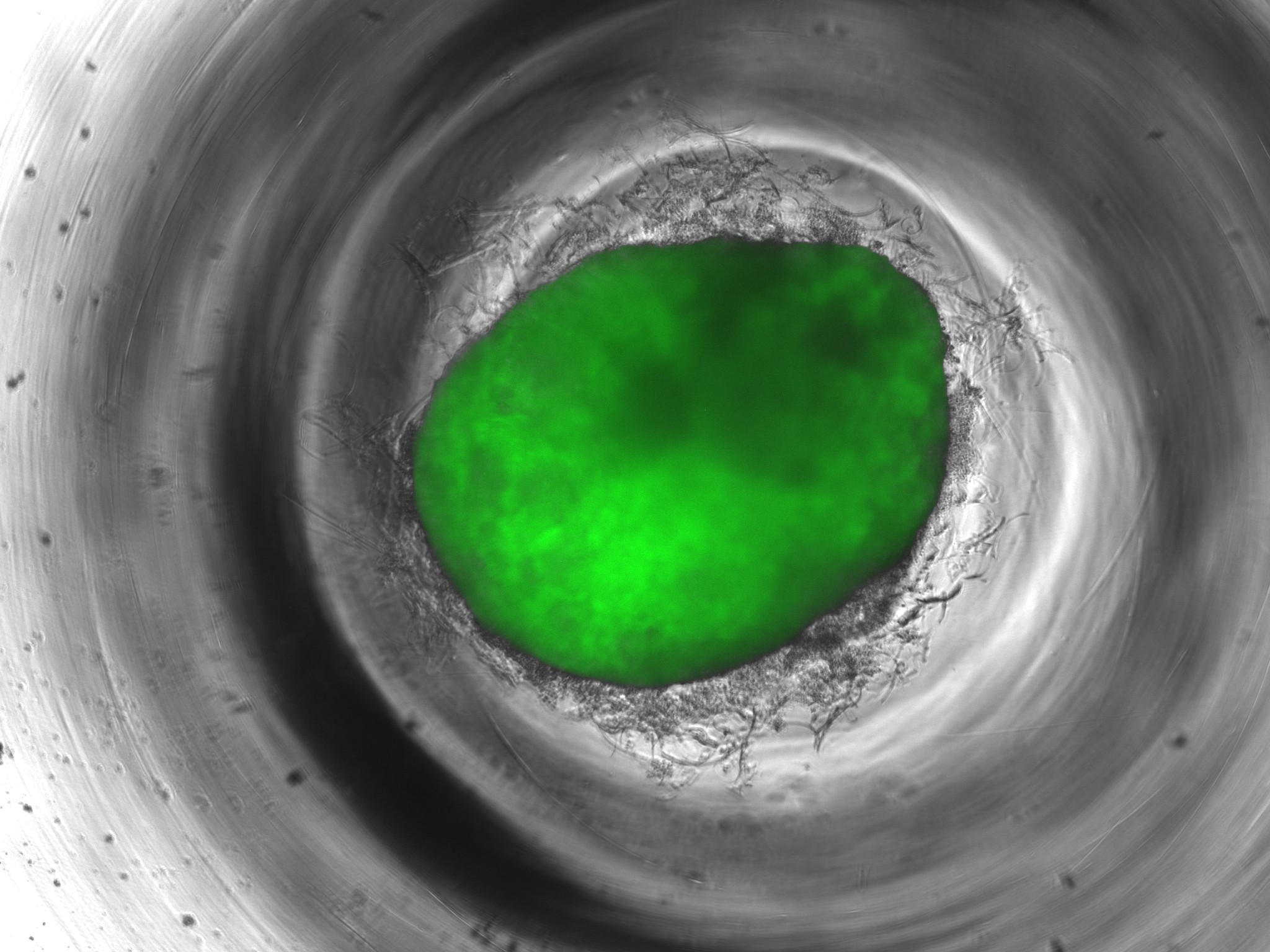

Supplement: Supplementary file 5 — Source data Fig. 2 [file 44318_2025_409_MOESM5_ESM.zip › EMBOJ-2024-118939R-Figure_2_Source_Data-sd/EMBOJ-2024-118939_Fig2B/HAND1_SB_2.jpg]

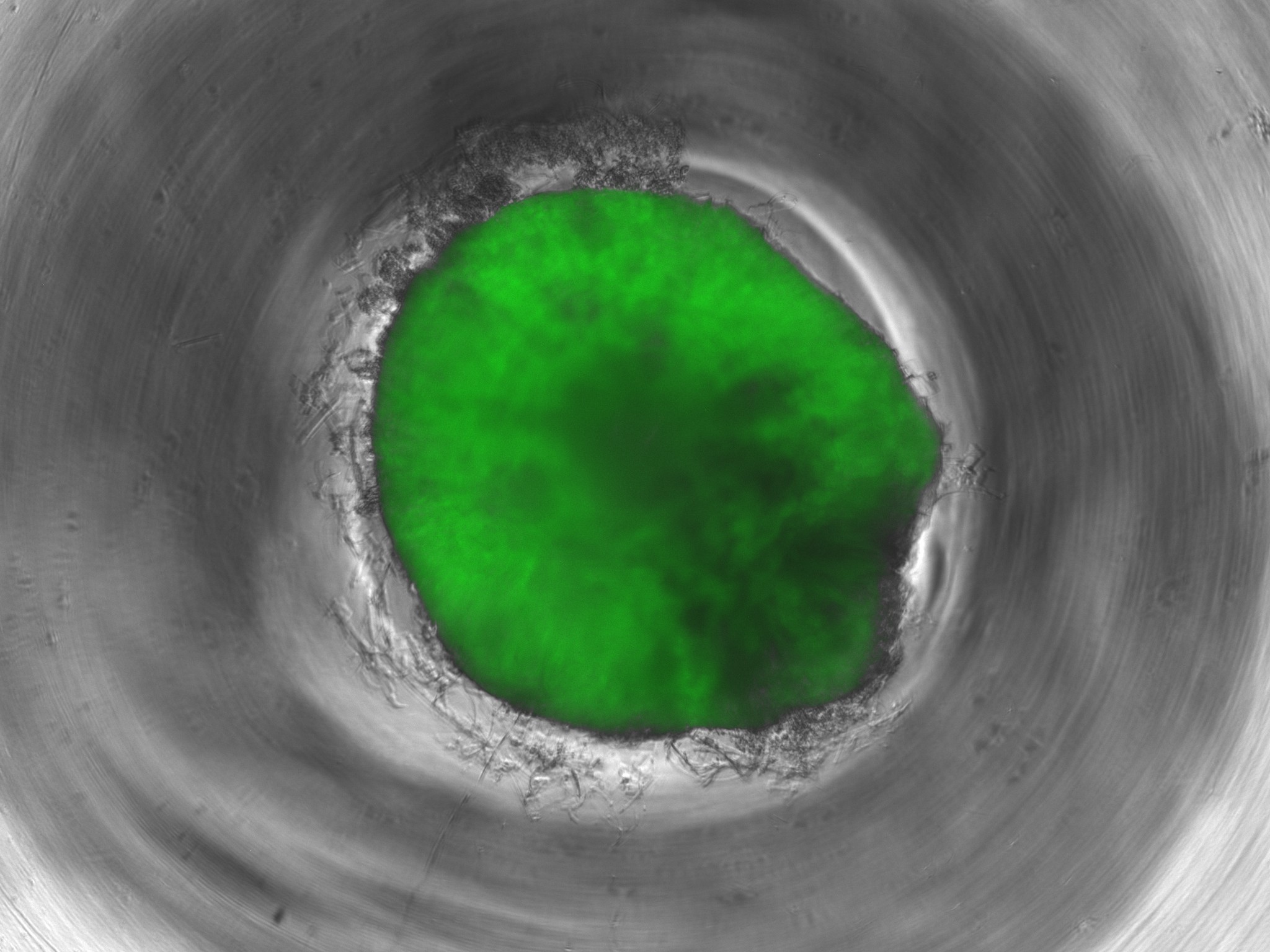

Supplement: Supplementary file 5 — Source data Fig. 2 [file 44318_2025_409_MOESM5_ESM.zip › EMBOJ-2024-118939R-Figure_2_Source_Data-sd/EMBOJ-2024-118939_Fig2B/HAND1_SB_3.jpg]

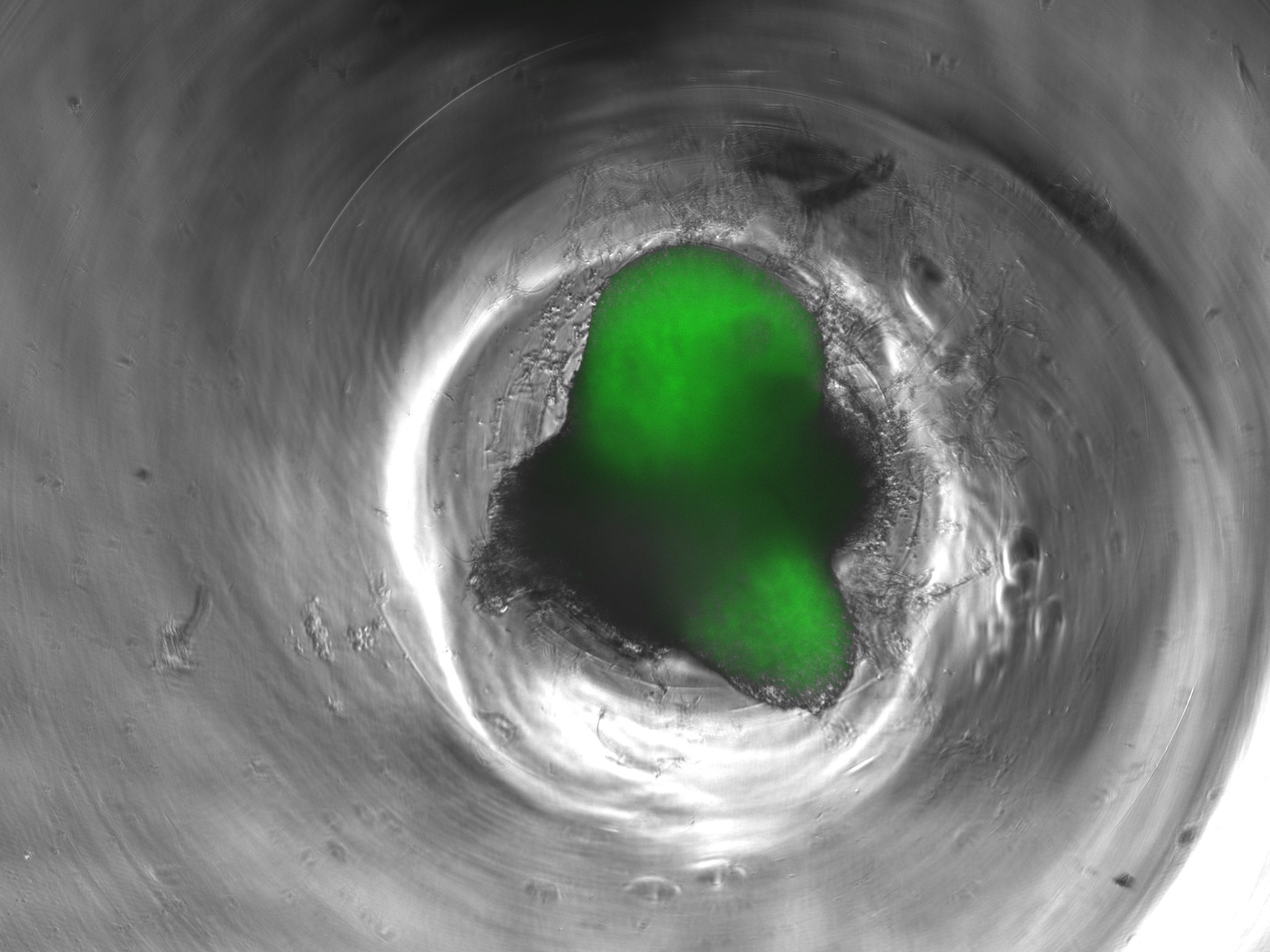

Supplement: Supplementary file 5 — Source data Fig. 2 [file 44318_2025_409_MOESM5_ESM.zip › EMBOJ-2024-118939R-Figure_2_Source_Data-sd/EMBOJ-2024-118939_Fig2B/HOXB_SB_4.jpg]

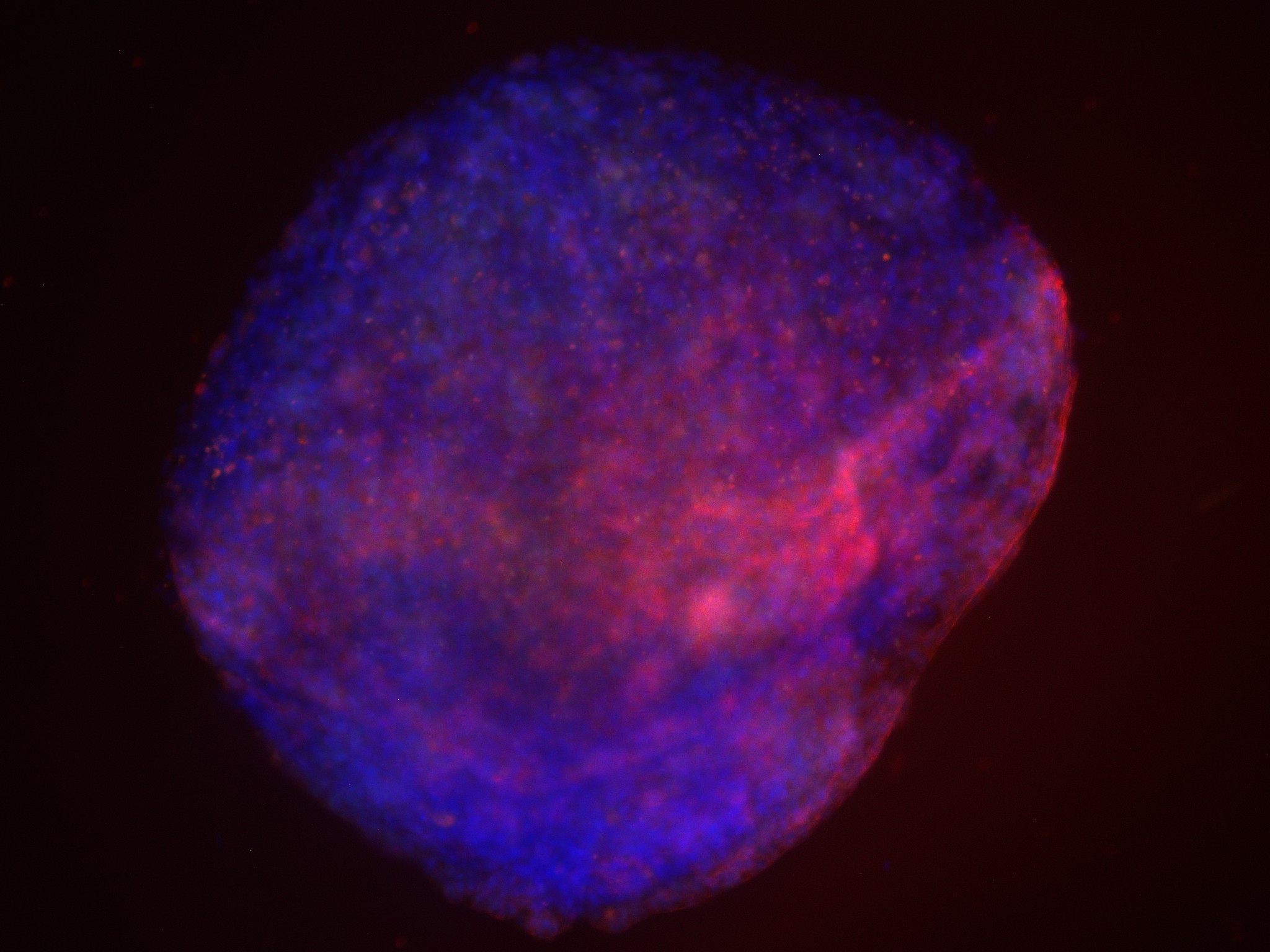

Supplement: Supplementary file 5 — Source data Fig. 2 [file 44318_2025_409_MOESM5_ESM.zip › EMBOJ-2024-118939R-Figure_2_Source_Data-sd/EMBOJ-2024-118939_Fig2E/HAND1-null_Merge.jpg]

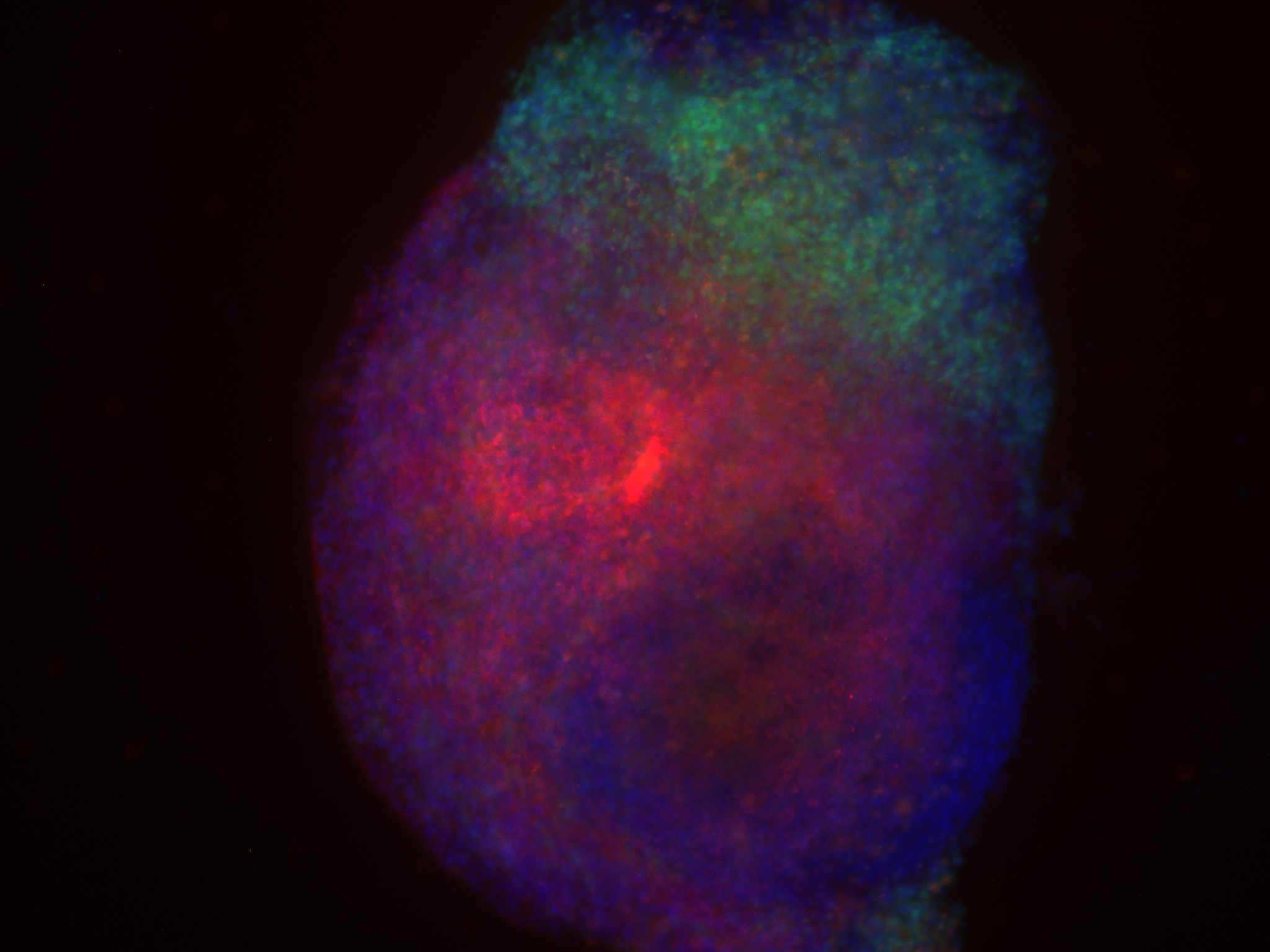

Supplement: Supplementary file 5 — Source data Fig. 2 [file 44318_2025_409_MOESM5_ESM.zip › EMBOJ-2024-118939R-Figure_2_Source_Data-sd/EMBOJ-2024-118939_Fig2E/Wild-Type_Merge.jpg]

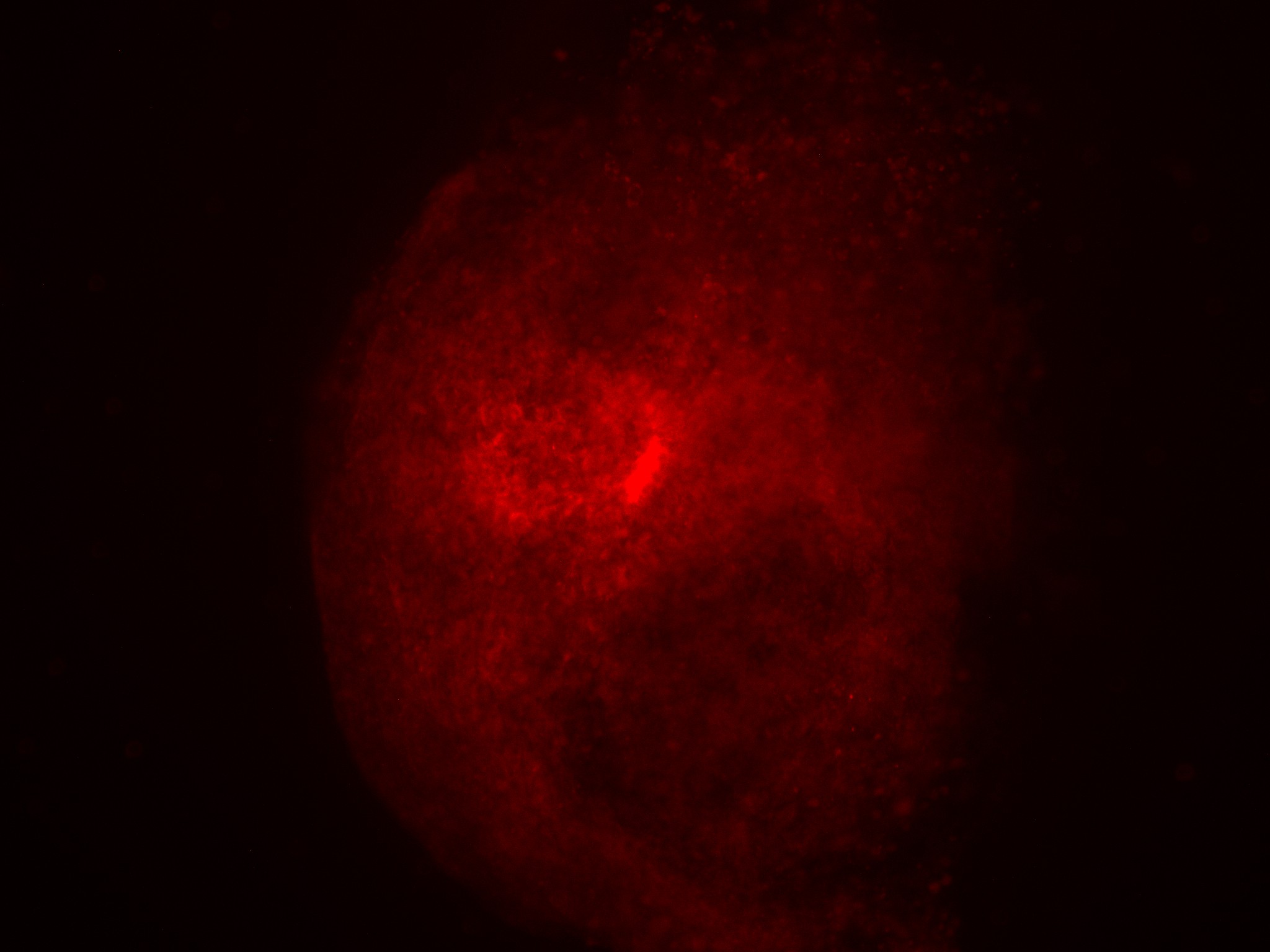

Supplement: Supplementary file 5 — Source data Fig. 2 [file 44318_2025_409_MOESM5_ESM.zip › EMBOJ-2024-118939R-Figure_2_Source_Data-sd/EMBOJ-2024-118939_Fig2E/Wild-Type aACTININ.jpg]

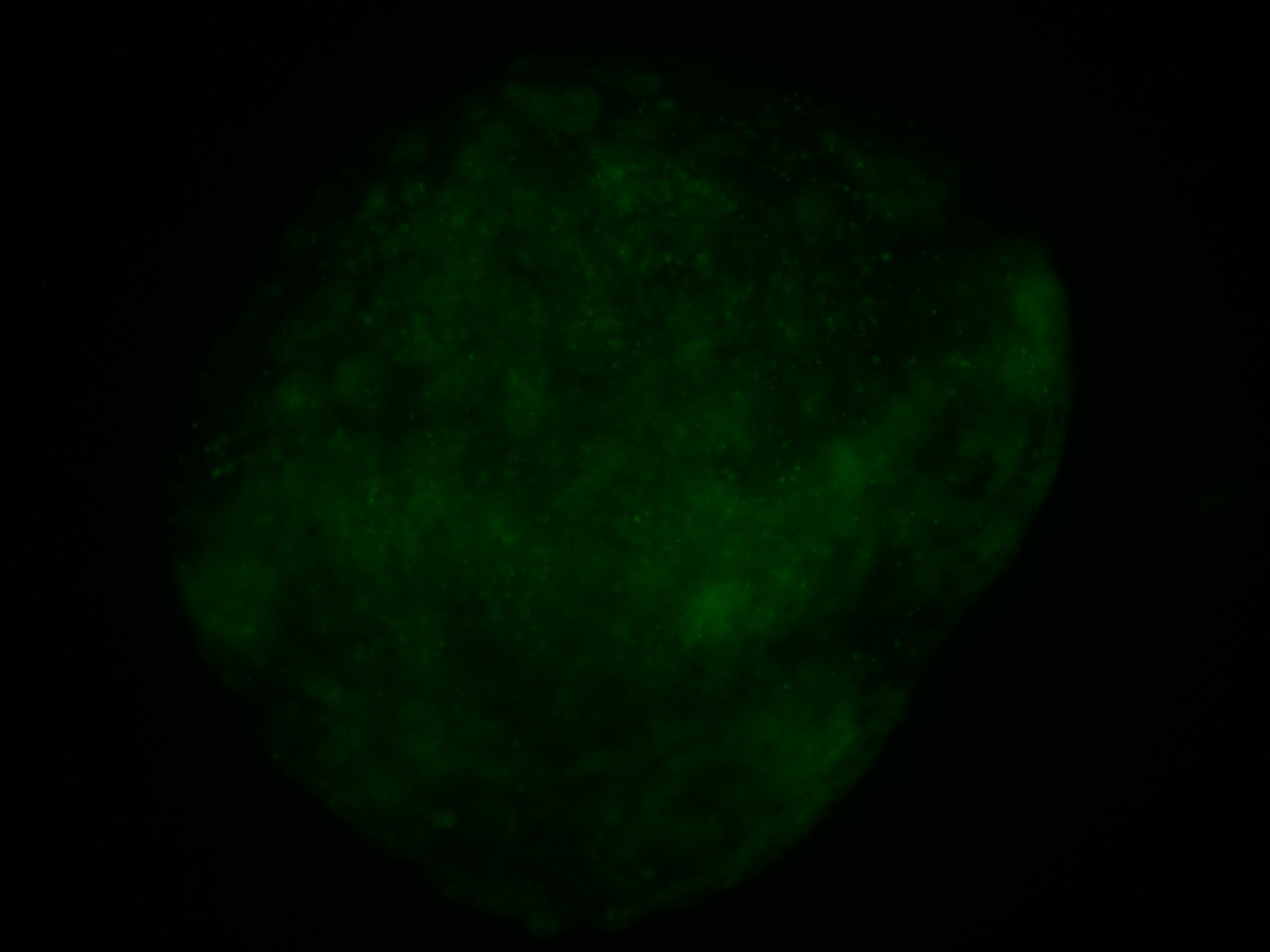

Supplement: Supplementary file 5 — Source data Fig. 2 [file 44318_2025_409_MOESM5_ESM.zip › EMBOJ-2024-118939R-Figure_2_Source_Data-sd/EMBOJ-2024-118939_Fig2E/HAND1-Null_WT1.jpg]

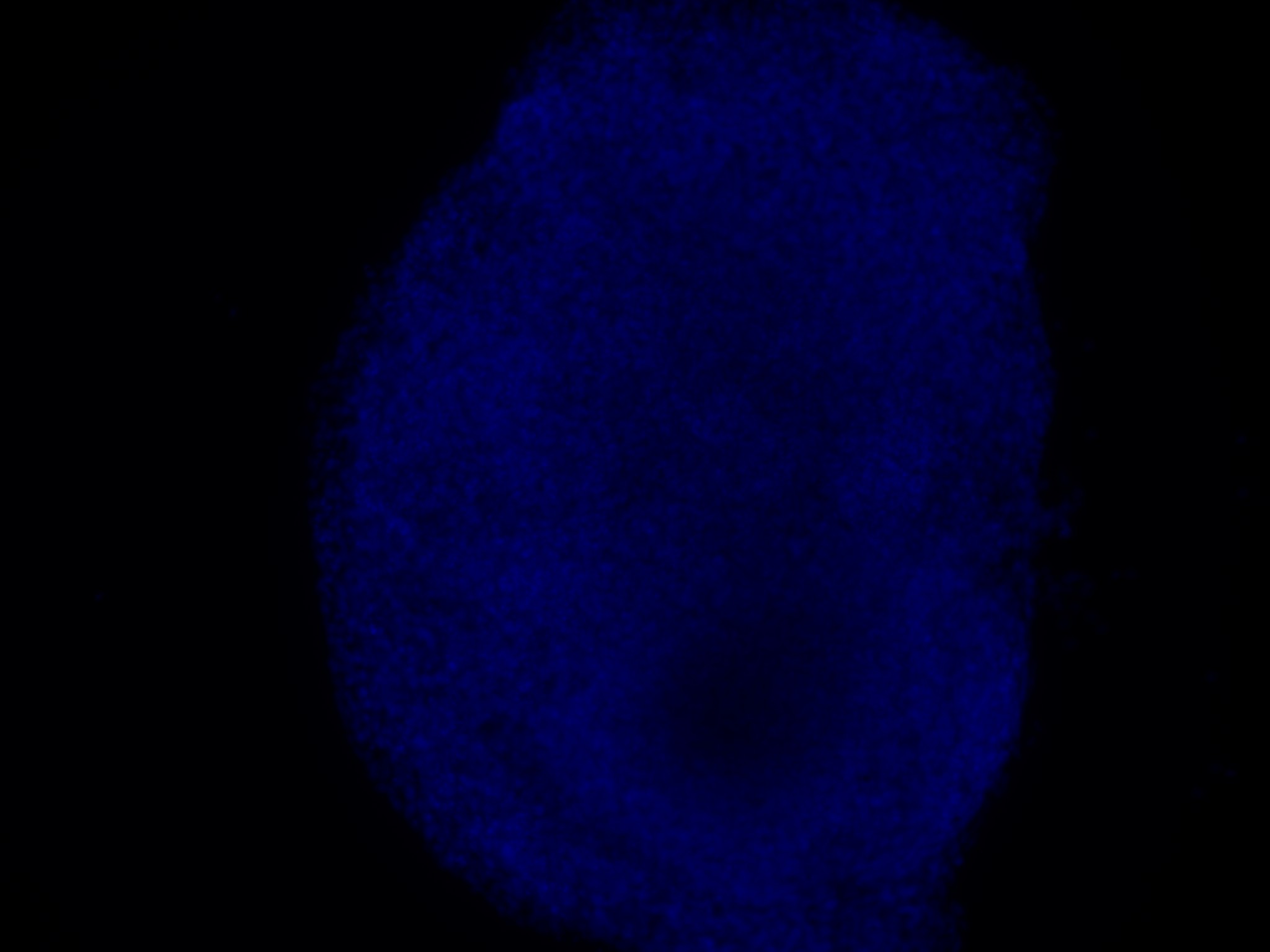

Supplement: Supplementary file 5 — Source data Fig. 2 [file 44318_2025_409_MOESM5_ESM.zip › EMBOJ-2024-118939R-Figure_2_Source_Data-sd/EMBOJ-2024-118939_Fig2E/Wild-Type DNA.jpg]

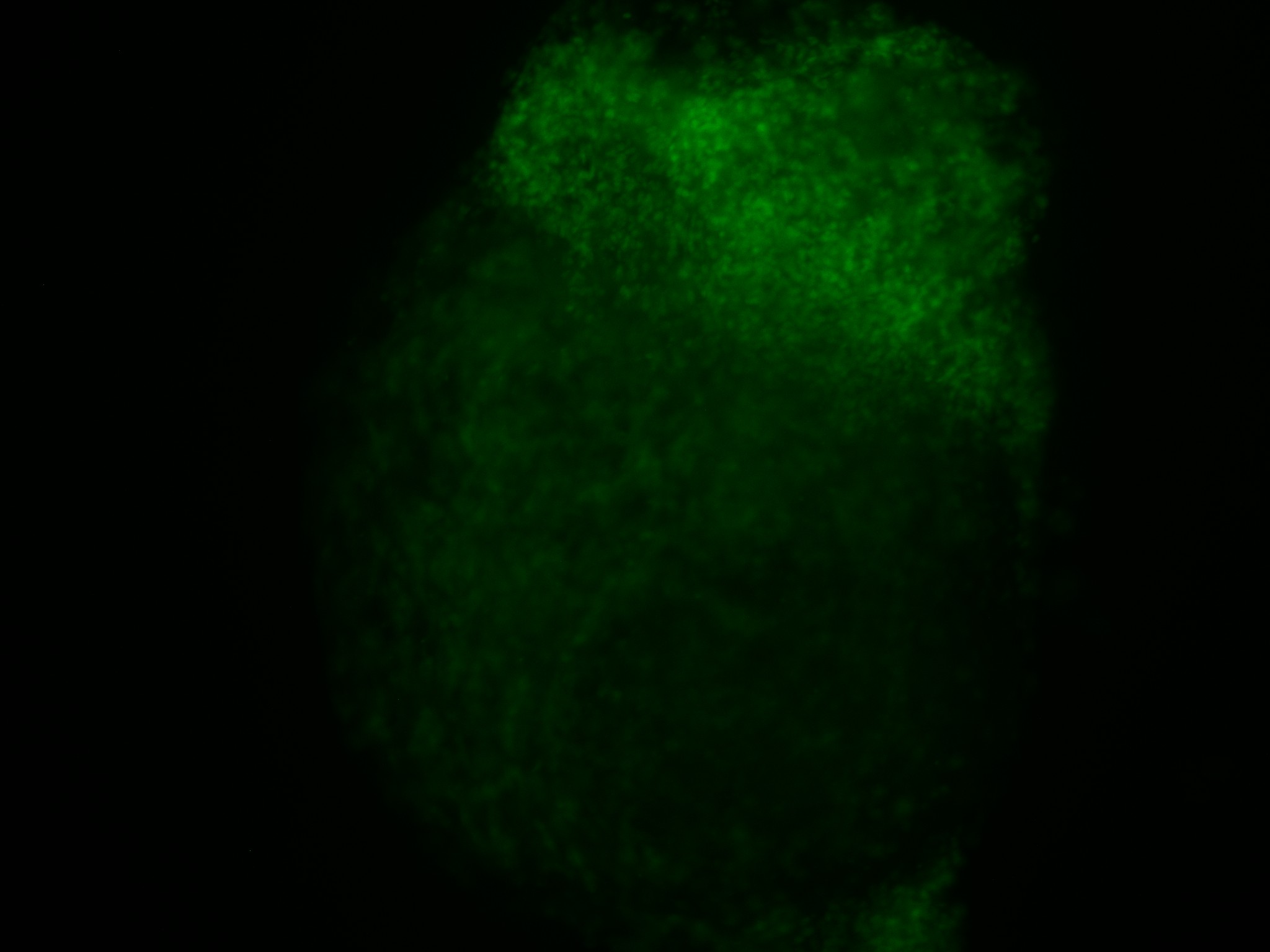

Supplement: Supplementary file 5 — Source data Fig. 2 [file 44318_2025_409_MOESM5_ESM.zip › EMBOJ-2024-118939R-Figure_2_Source_Data-sd/EMBOJ-2024-118939_Fig2E/Wild-Type WT1.jpg]

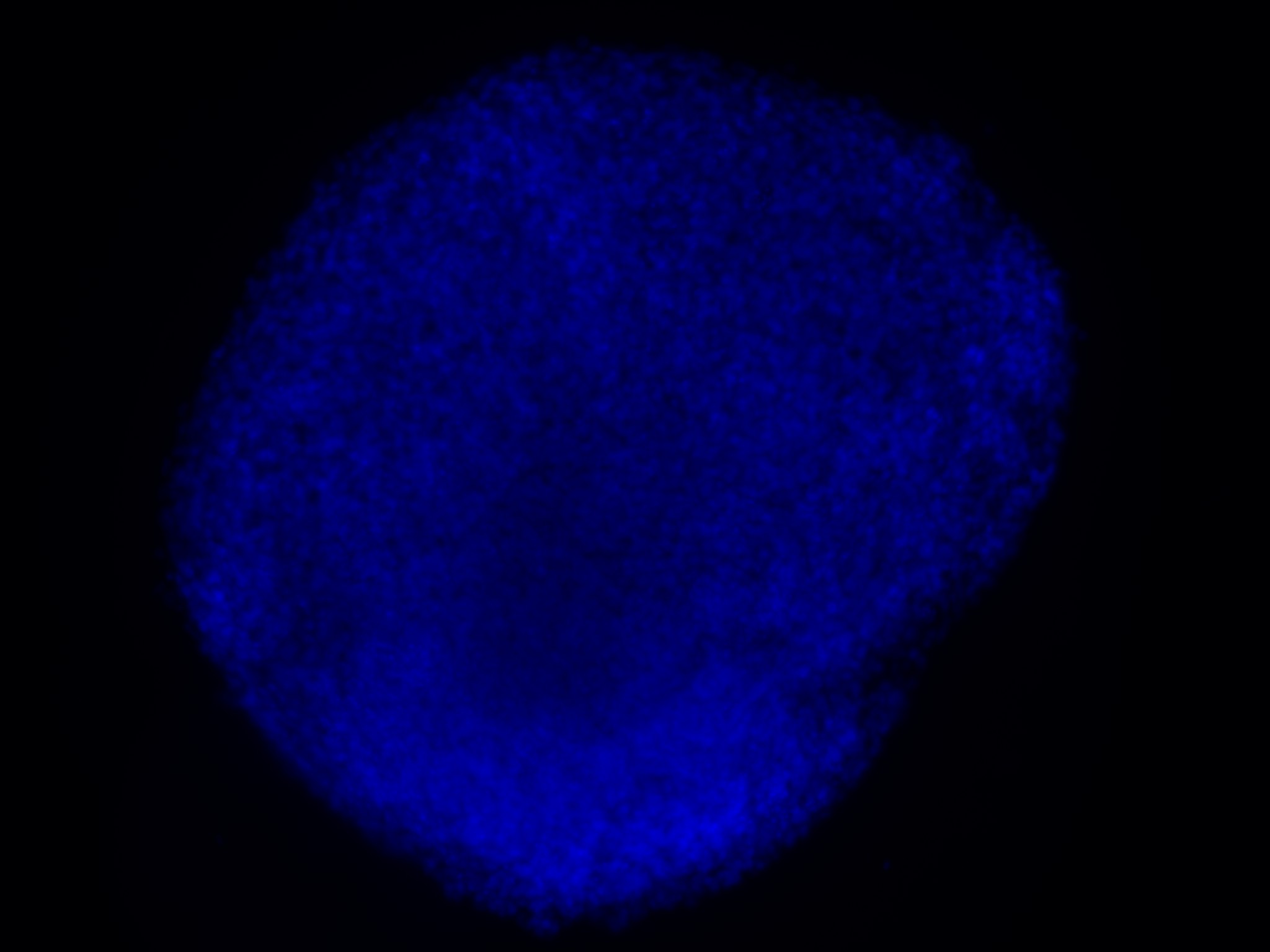

Supplement: Supplementary file 5 — Source data Fig. 2 [file 44318_2025_409_MOESM5_ESM.zip › EMBOJ-2024-118939R-Figure_2_Source_Data-sd/EMBOJ-2024-118939_Fig2E/HAND1-Null_DNA.jpg]

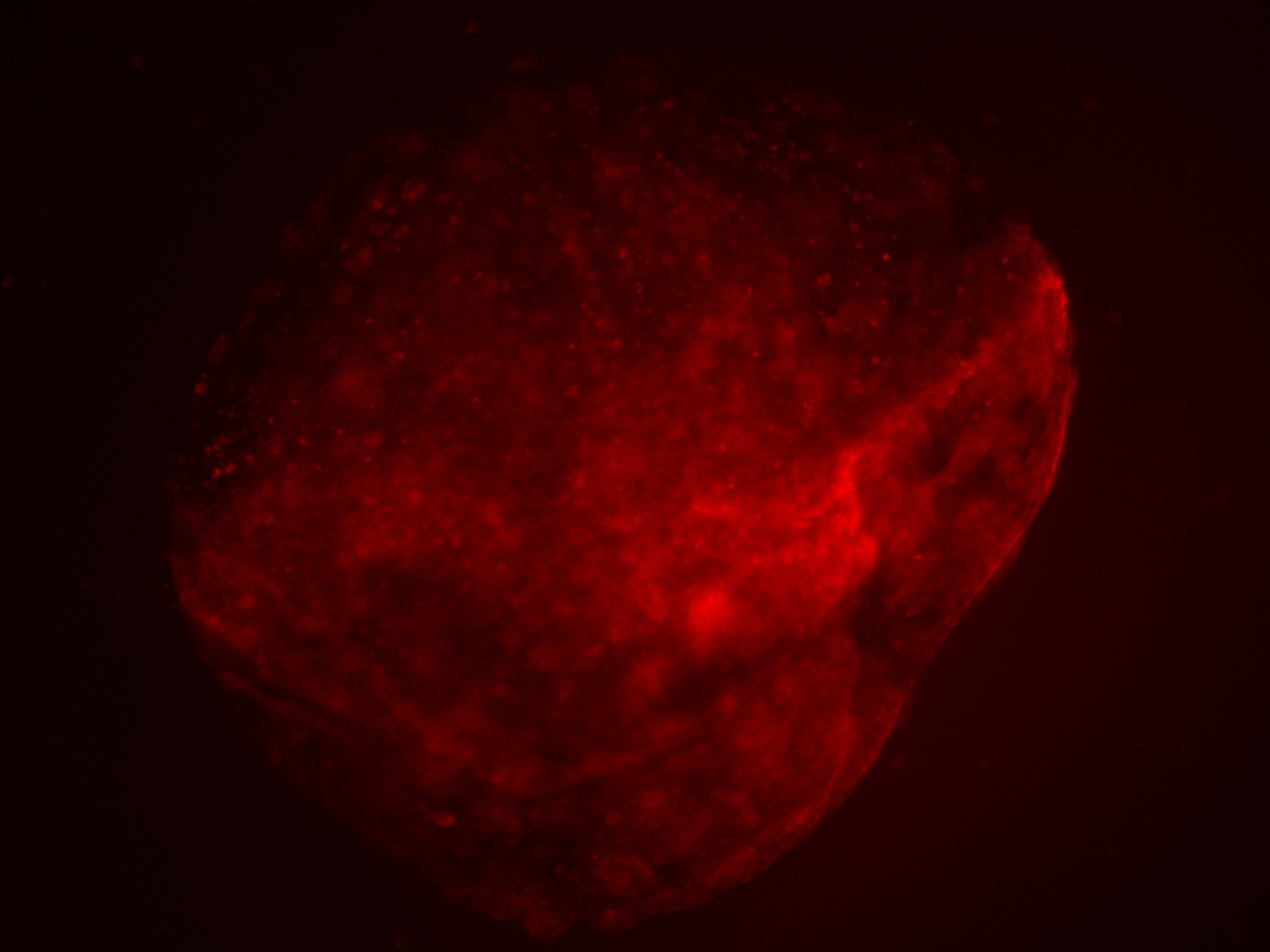

Supplement: Supplementary file 5 — Source data Fig. 2 [file 44318_2025_409_MOESM5_ESM.zip › EMBOJ-2024-118939R-Figure_2_Source_Data-sd/EMBOJ-2024-118939_Fig2E/HAND1-Null_aACTININ.jpg]

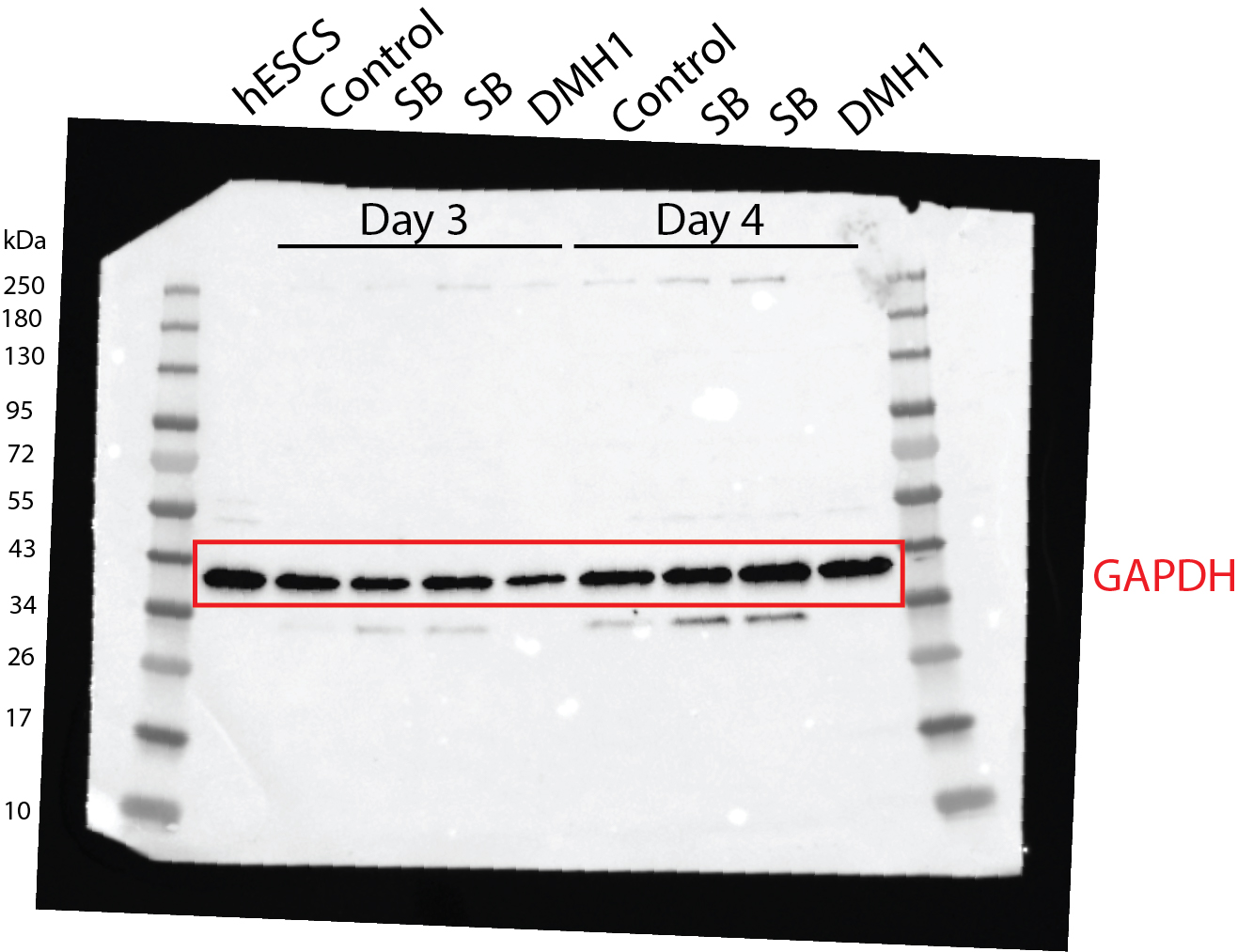

Supplement: Supplementary file 6 — Source data Fig. 5 [file 44318_2025_409_MOESM6_ESM.zip › EMBOJ-2024-118939R-Figure_5_Source_Data-sd/EMBOJ-2024-118939_Fig5A/GAPDH_WB.jpg]

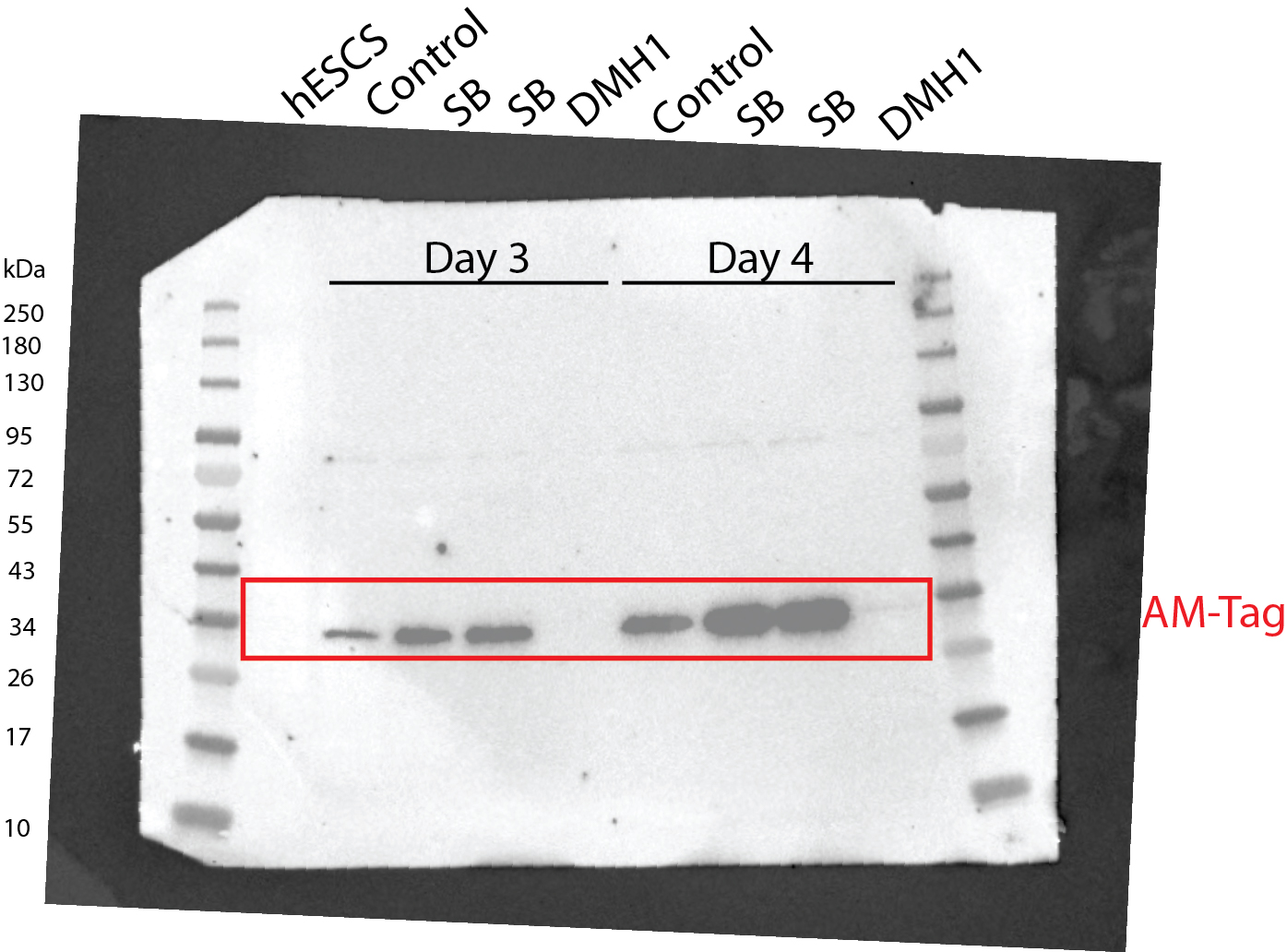

Supplement: Supplementary file 6 — Source data Fig. 5 [file 44318_2025_409_MOESM6_ESM.zip › EMBOJ-2024-118939R-Figure_5_Source_Data-sd/EMBOJ-2024-118939_Fig5A/AMTag_WB.jpg]

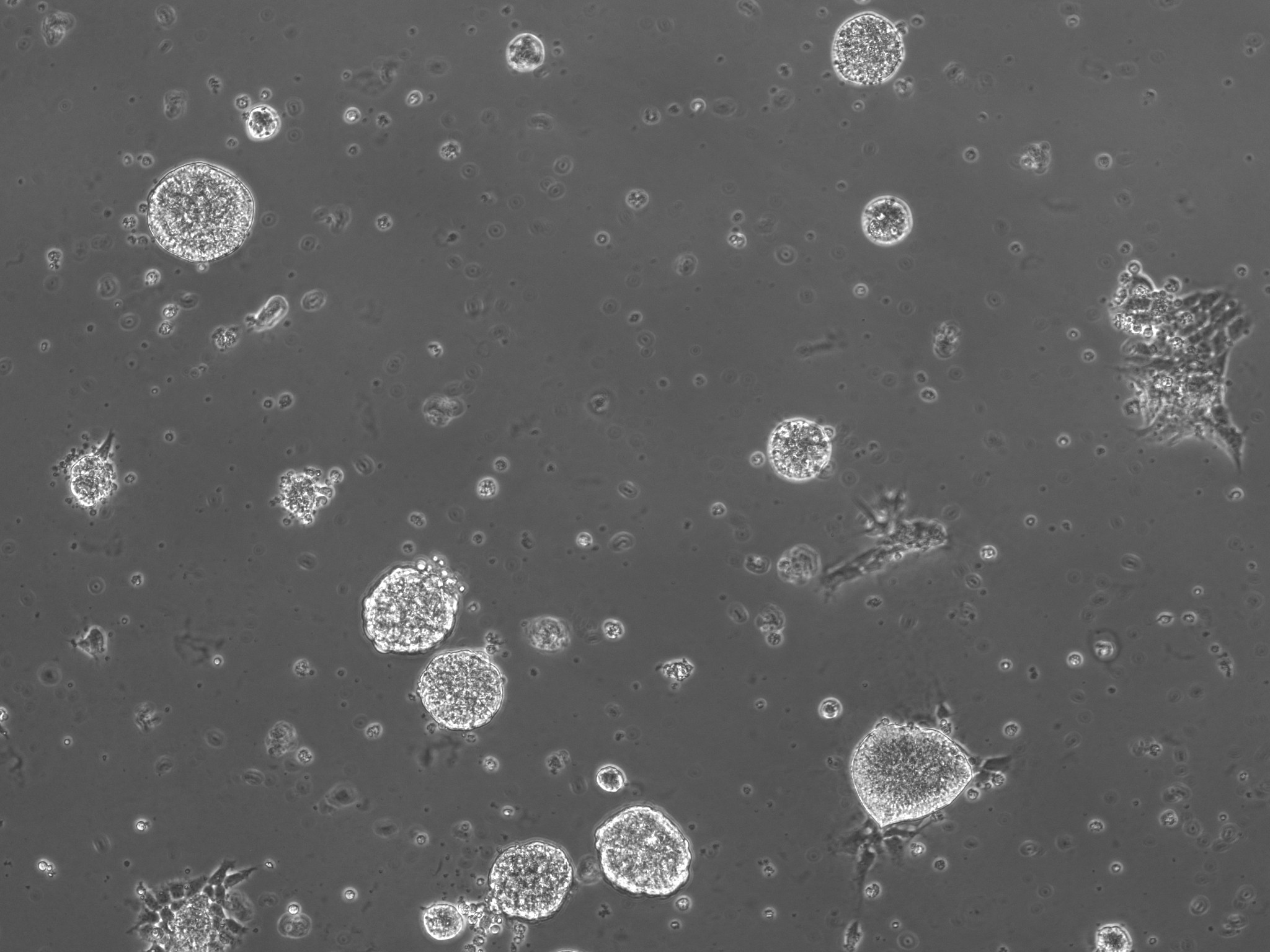

Supplement: Supplementary file 7 — Source data Fig. 6 [file 44318_2025_409_MOESM7_ESM.zip › EMBOJ-2024-118939R-Figure_6_Source_Data-sd/EMBOJ-2024-118939_Fig6F/HAND1-neg_BF.tif]

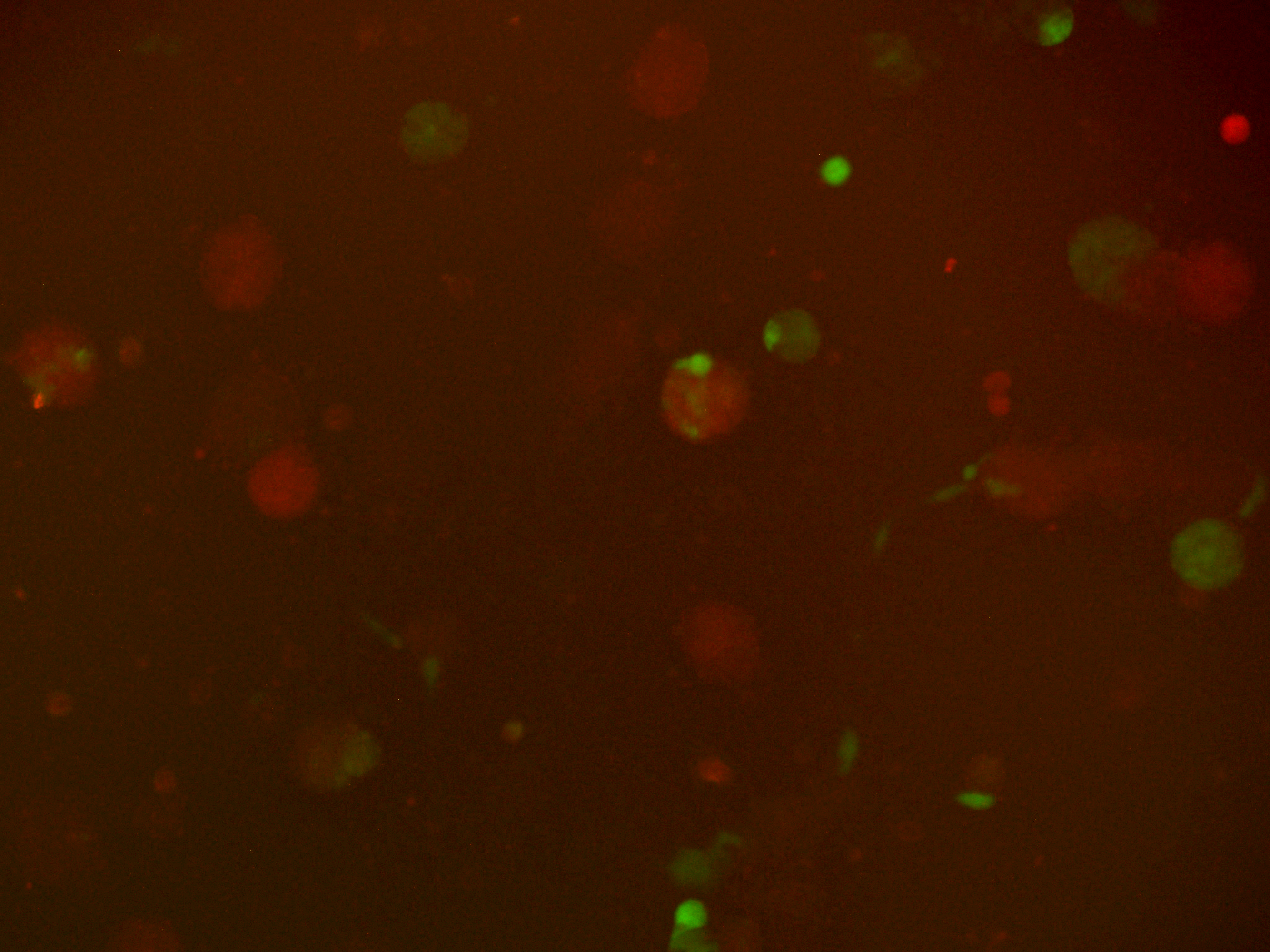

Supplement: Supplementary file 7 — Source data Fig. 6 [file 44318_2025_409_MOESM7_ESM.zip › EMBOJ-2024-118939R-Figure_6_Source_Data-sd/EMBOJ-2024-118939_Fig6F/HAND1-low_overlay.tif]

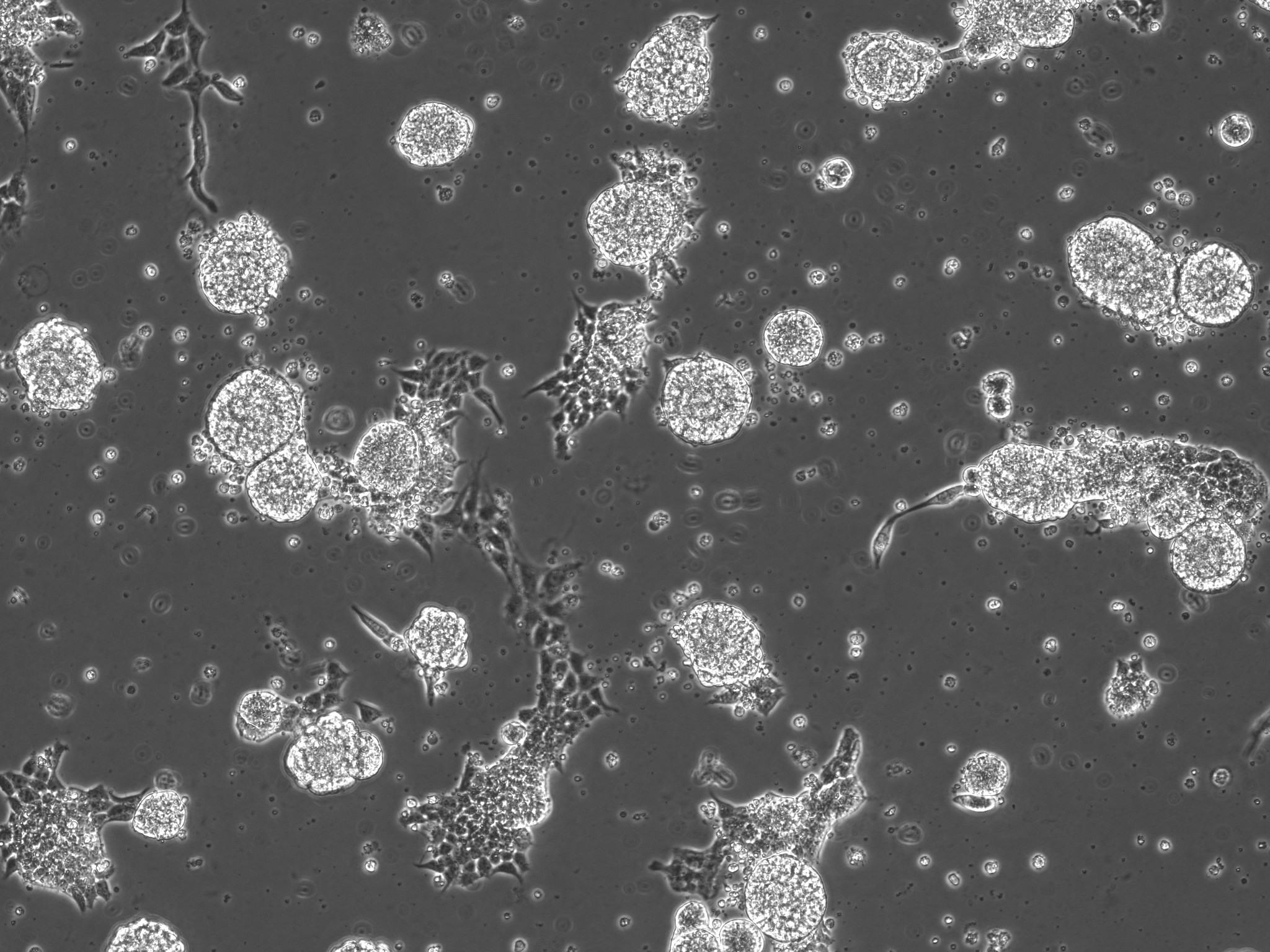

Supplement: Supplementary file 7 — Source data Fig. 6 [file 44318_2025_409_MOESM7_ESM.zip › EMBOJ-2024-118939R-Figure_6_Source_Data-sd/EMBOJ-2024-118939_Fig6F/HAND1-low-BF.tif]

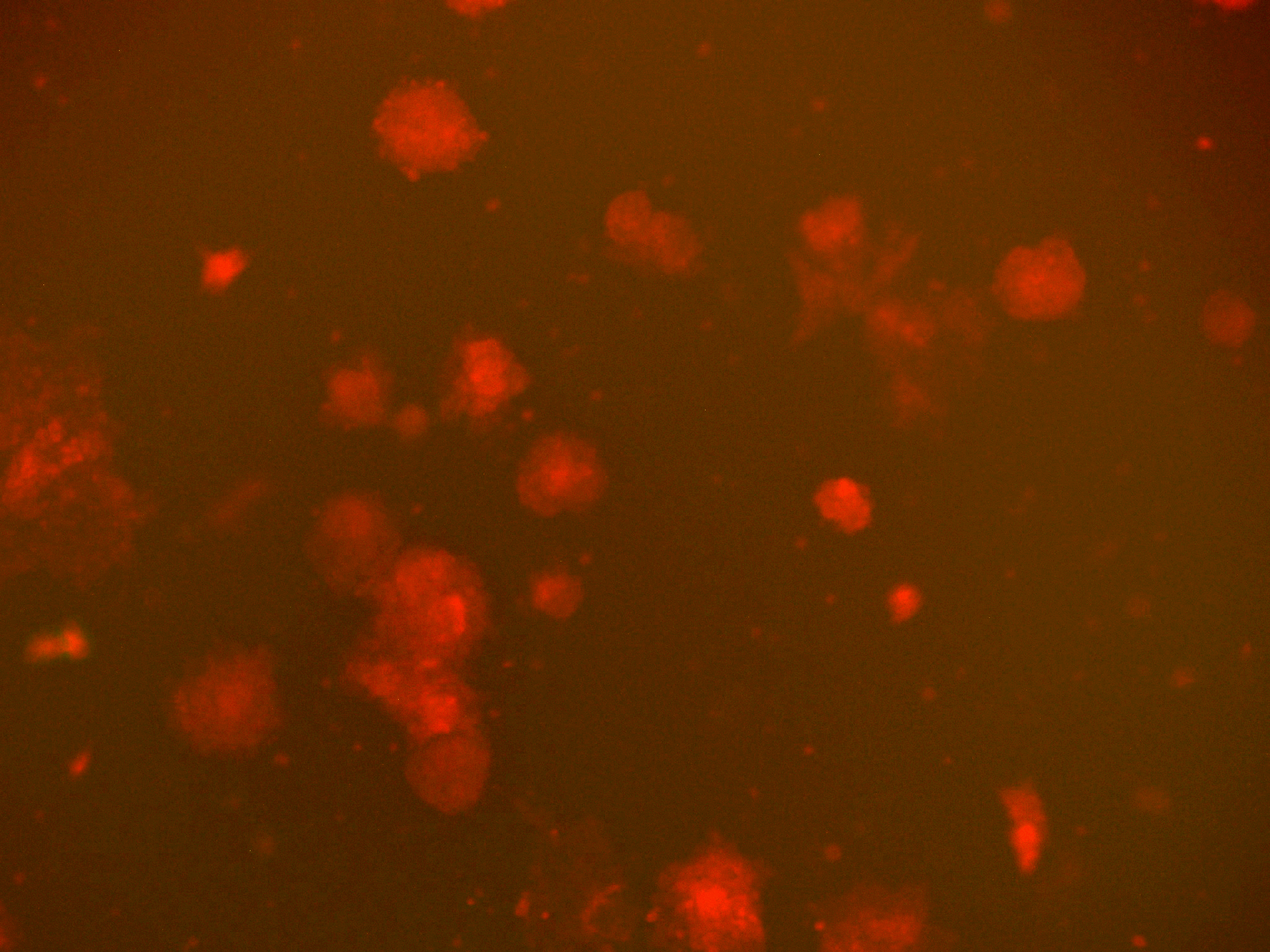

Supplement: Supplementary file 7 — Source data Fig. 6 [file 44318_2025_409_MOESM7_ESM.zip › EMBOJ-2024-118939R-Figure_6_Source_Data-sd/EMBOJ-2024-118939_Fig6F/HAND1-high_overlay.tif]

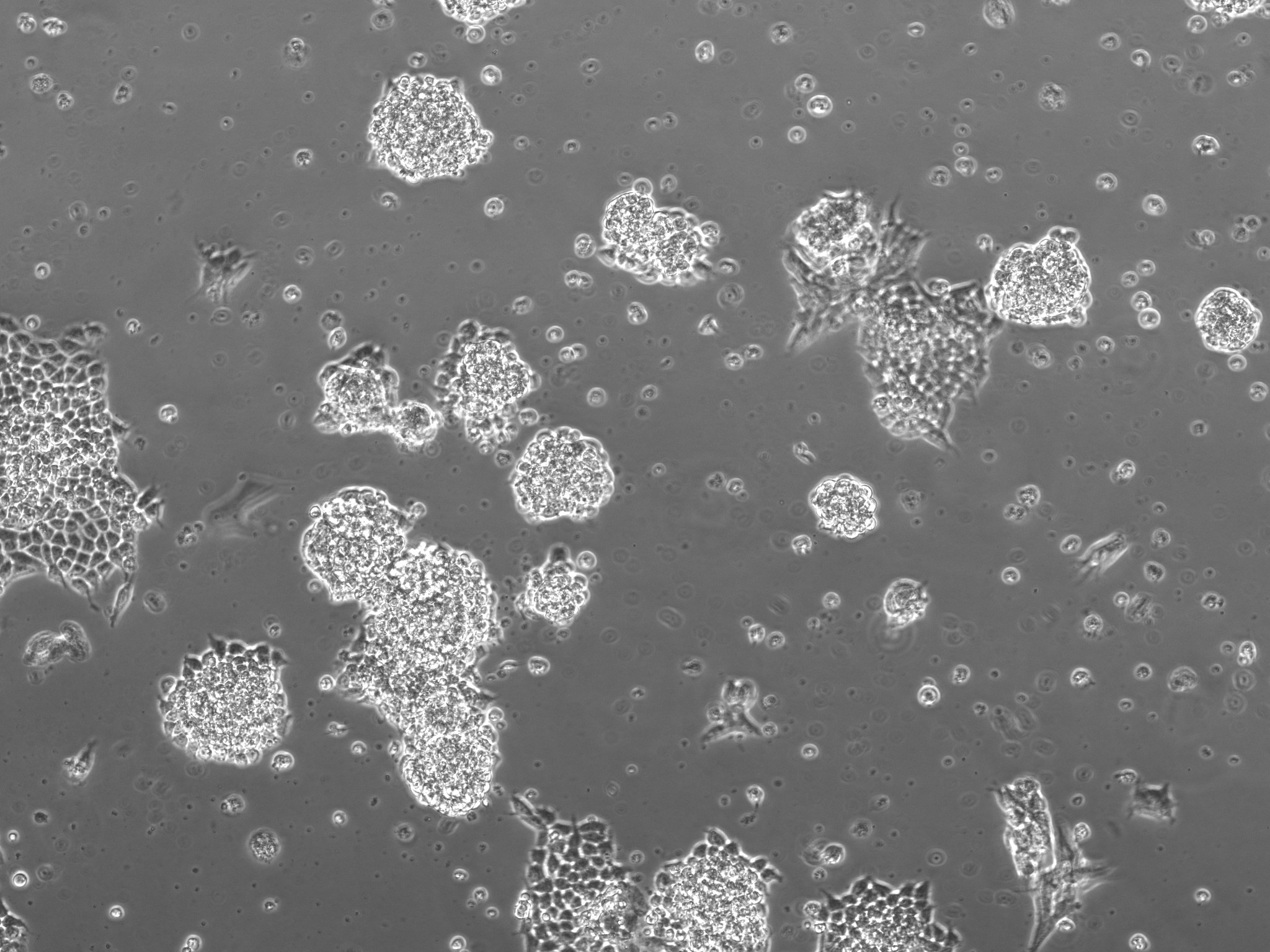

Supplement: Supplementary file 7 — Source data Fig. 6 [file 44318_2025_409_MOESM7_ESM.zip › EMBOJ-2024-118939R-Figure_6_Source_Data-sd/EMBOJ-2024-118939_Fig6F/HAND1-high_BF.tif]

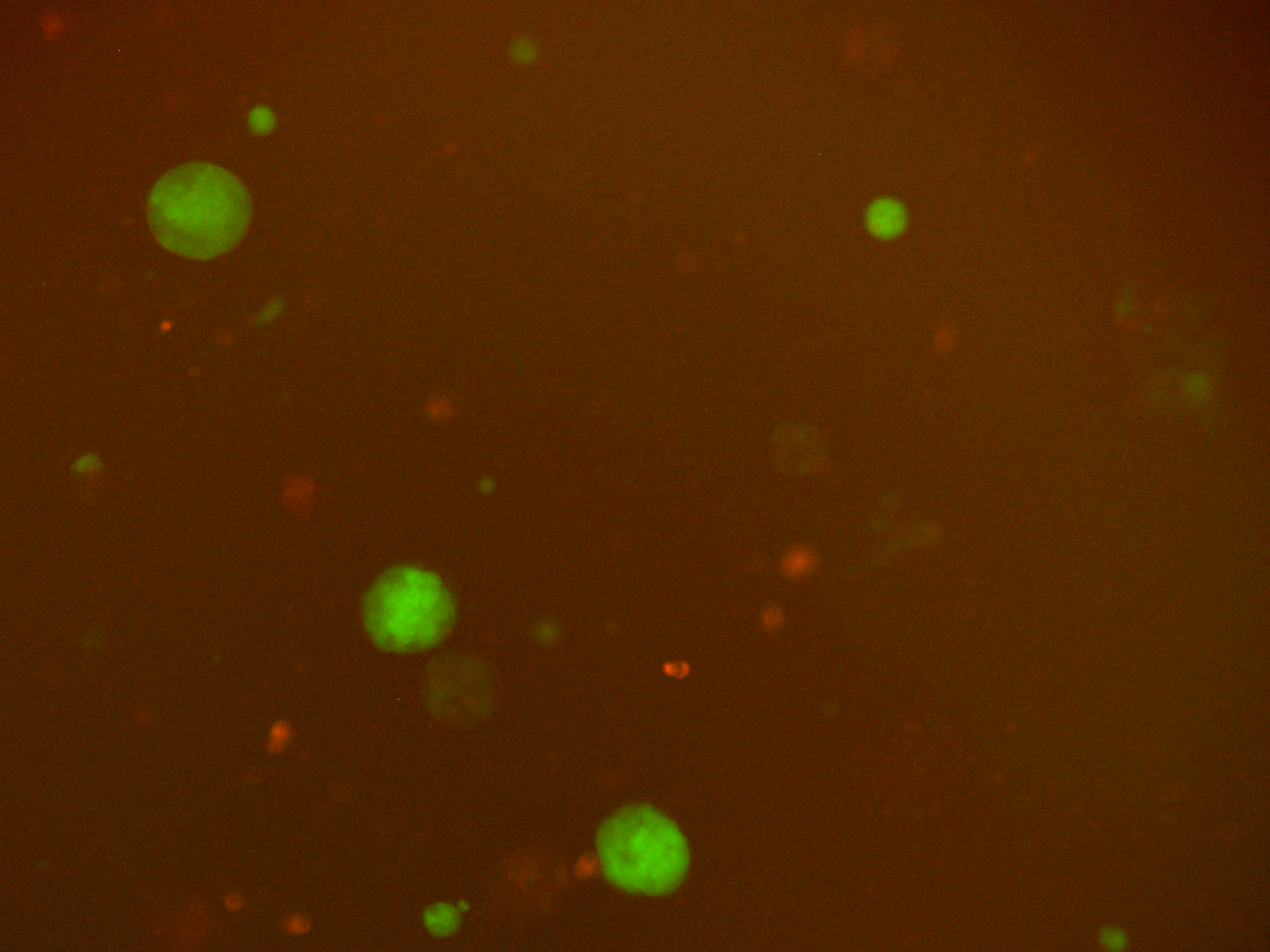

Supplement: Supplementary file 7 — Source data Fig. 6 [file 44318_2025_409_MOESM7_ESM.zip › EMBOJ-2024-118939R-Figure_6_Source_Data-sd/EMBOJ-2024-118939_Fig6F/HAND1-neg_overlay.tif]

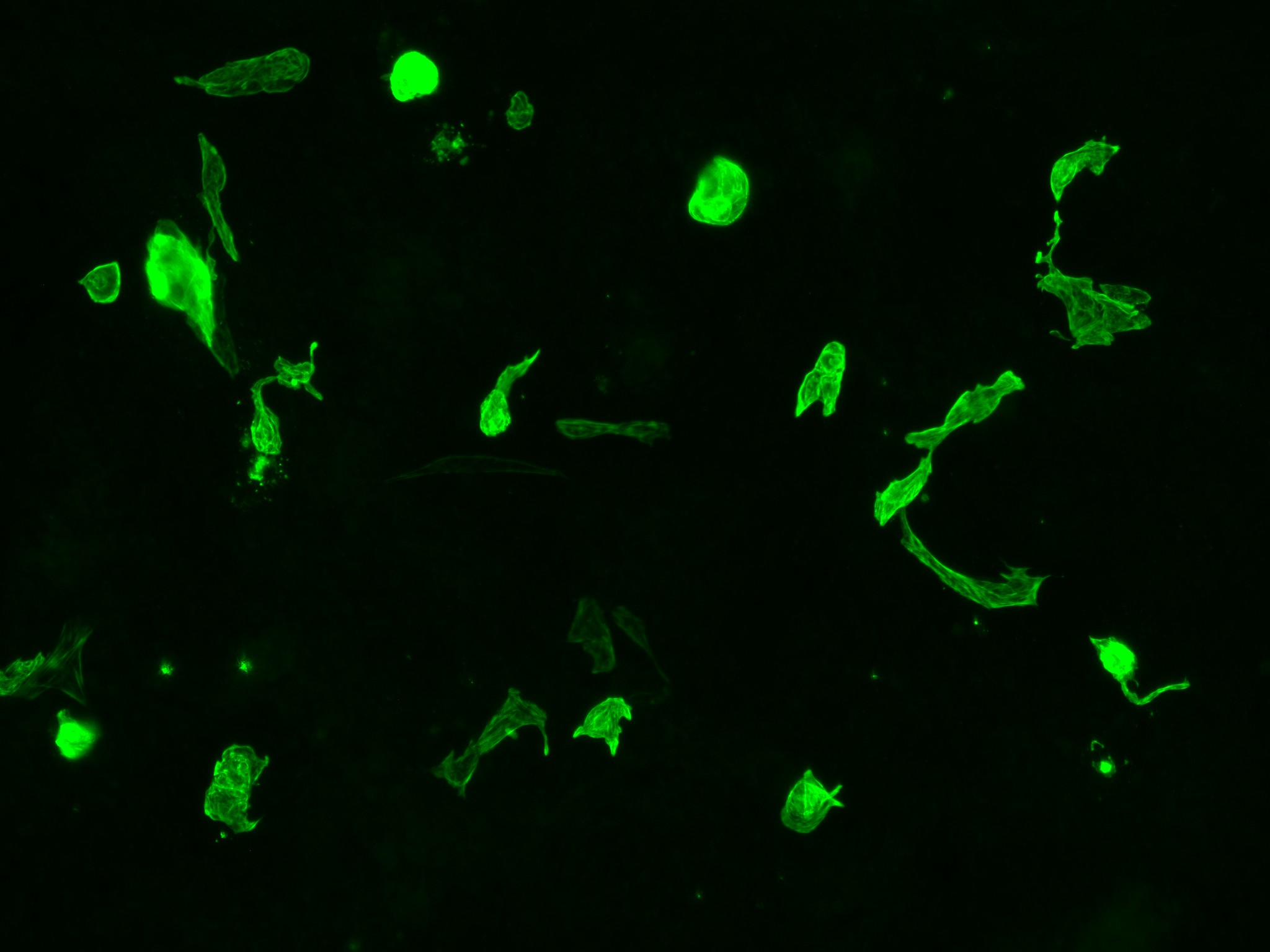

Supplement: Supplementary file 7 — Source data Fig. 6 [file 44318_2025_409_MOESM7_ESM.zip › EMBOJ-2024-118939R-Figure_6_Source_Data-sd/EMBOJ-2024-118939_Fig6H/HAND1-high_ACTN2.jpg]

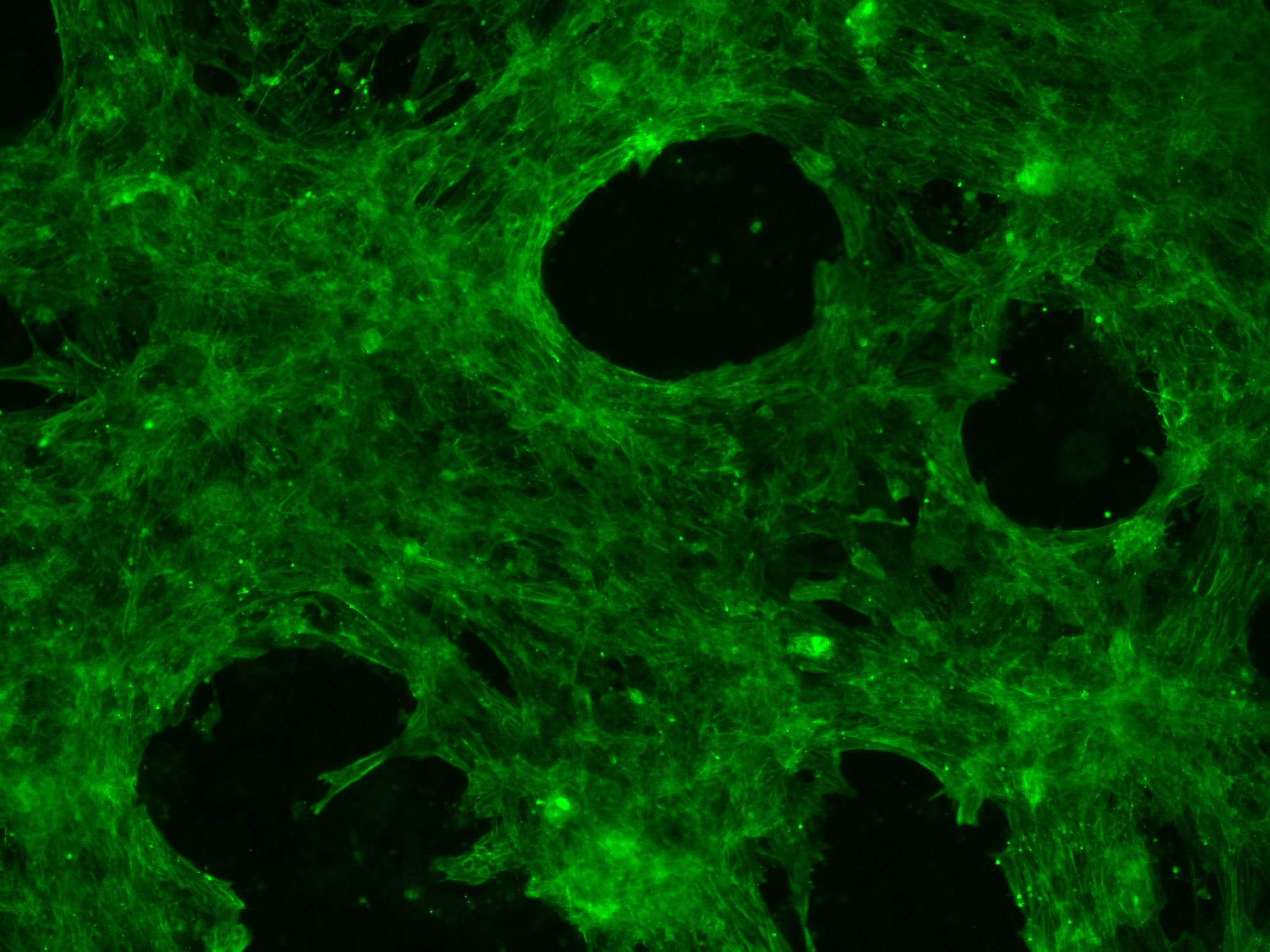

Supplement: Supplementary file 7 — Source data Fig. 6 [file 44318_2025_409_MOESM7_ESM.zip › EMBOJ-2024-118939R-Figure_6_Source_Data-sd/EMBOJ-2024-118939_Fig6H/HAND1-low_ACTN2.jpg]

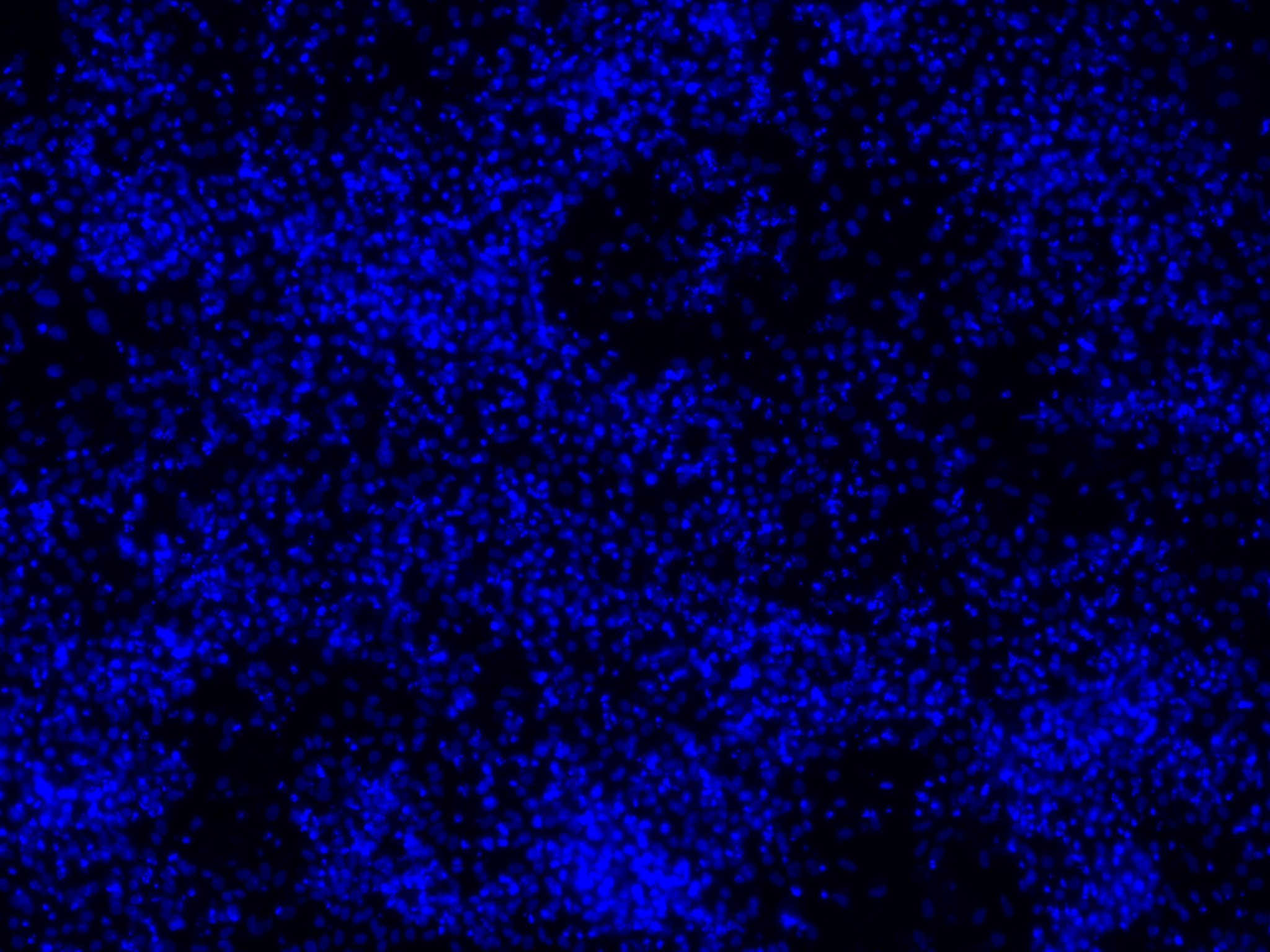

Supplement: Supplementary file 7 — Source data Fig. 6 [file 44318_2025_409_MOESM7_ESM.zip › EMBOJ-2024-118939R-Figure_6_Source_Data-sd/EMBOJ-2024-118939_Fig6H/HAND1-low_DAPI.jpg]

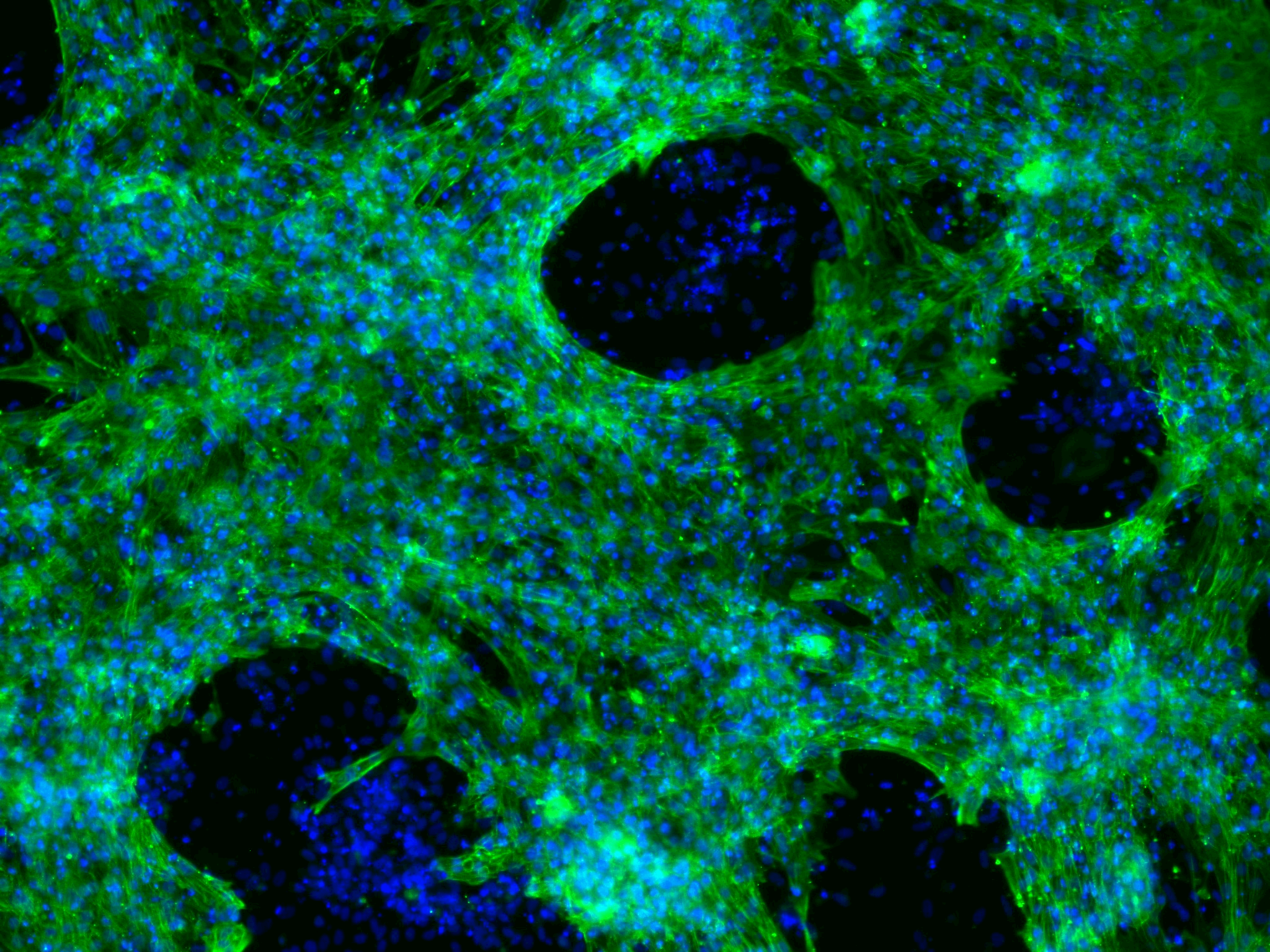

Supplement: Supplementary file 7 — Source data Fig. 6 [file 44318_2025_409_MOESM7_ESM.zip › EMBOJ-2024-118939R-Figure_6_Source_Data-sd/EMBOJ-2024-118939_Fig6H/HAND1-low_overlay.tif]

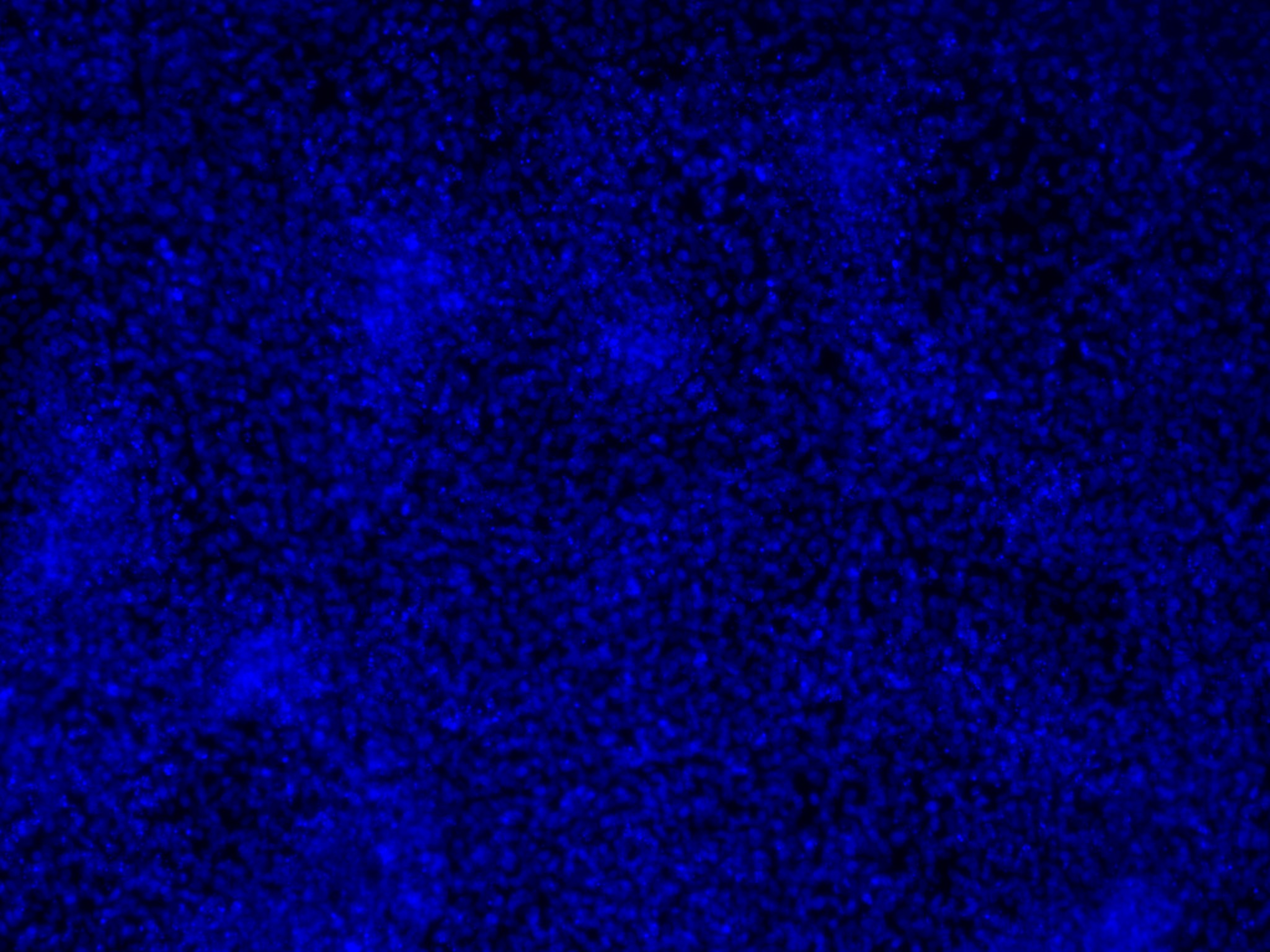

Supplement: Supplementary file 7 — Source data Fig. 6 [file 44318_2025_409_MOESM7_ESM.zip › EMBOJ-2024-118939R-Figure_6_Source_Data-sd/EMBOJ-2024-118939_Fig6H/HAND1-high_DAPI.jpg]

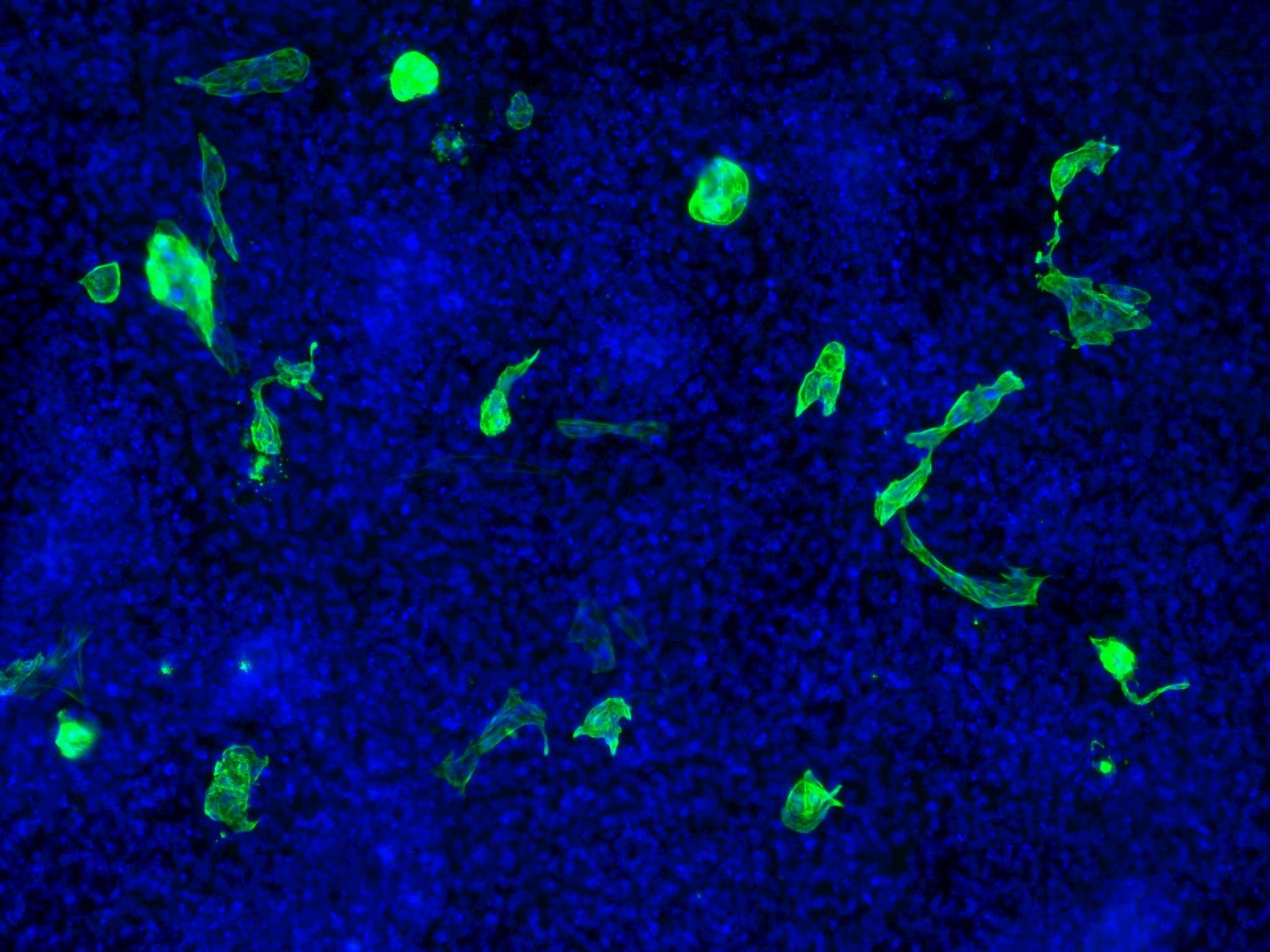

Supplement: Supplementary file 7 — Source data Fig. 6 [file 44318_2025_409_MOESM7_ESM.zip › EMBOJ-2024-118939R-Figure_6_Source_Data-sd/EMBOJ-2024-118939_Fig6H/HAND1-high_overlay.tif]
