# Supplementary figures and images for: HAND1 level controls the specification of multipotent cardiac and extraembryonic progenitors from human pluripotent stem cells (part 2 of 2)
Source: EMBO J. 2025 Mar 31;44(9):2541–65. doi: 10.1038/s44318-025-00409-0 (PMC12048643; doi:10.1038/s44318-025-00409-0)

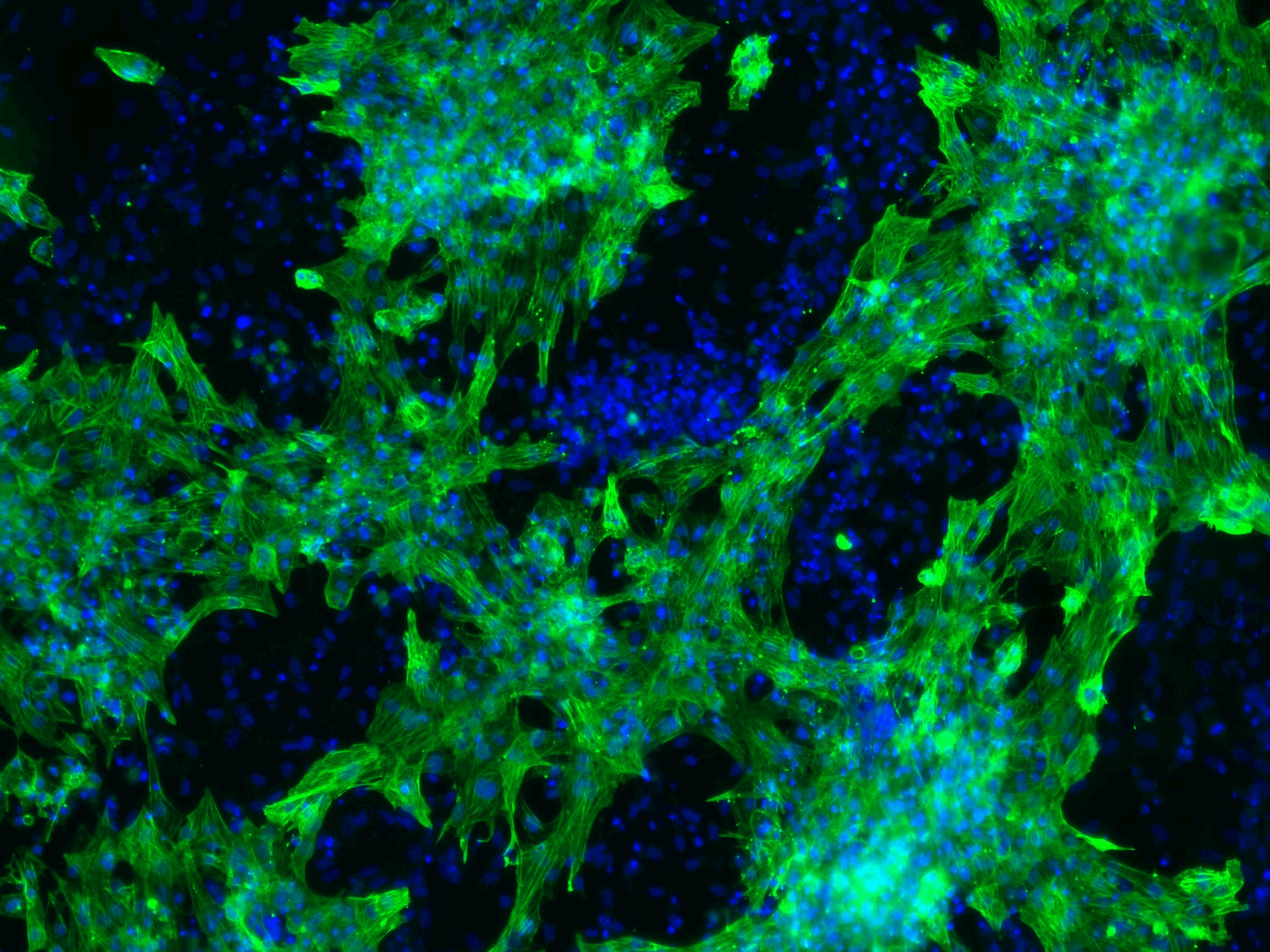

Supplement: Supplementary file 7 — Source data Fig. 6 [file 44318_2025_409_MOESM7_ESM.zip › EMBOJ-2024-118939R-Figure_6_Source_Data-sd/EMBOJ-2024-118939_Fig6H/HAND1-neg_overlay.tif]

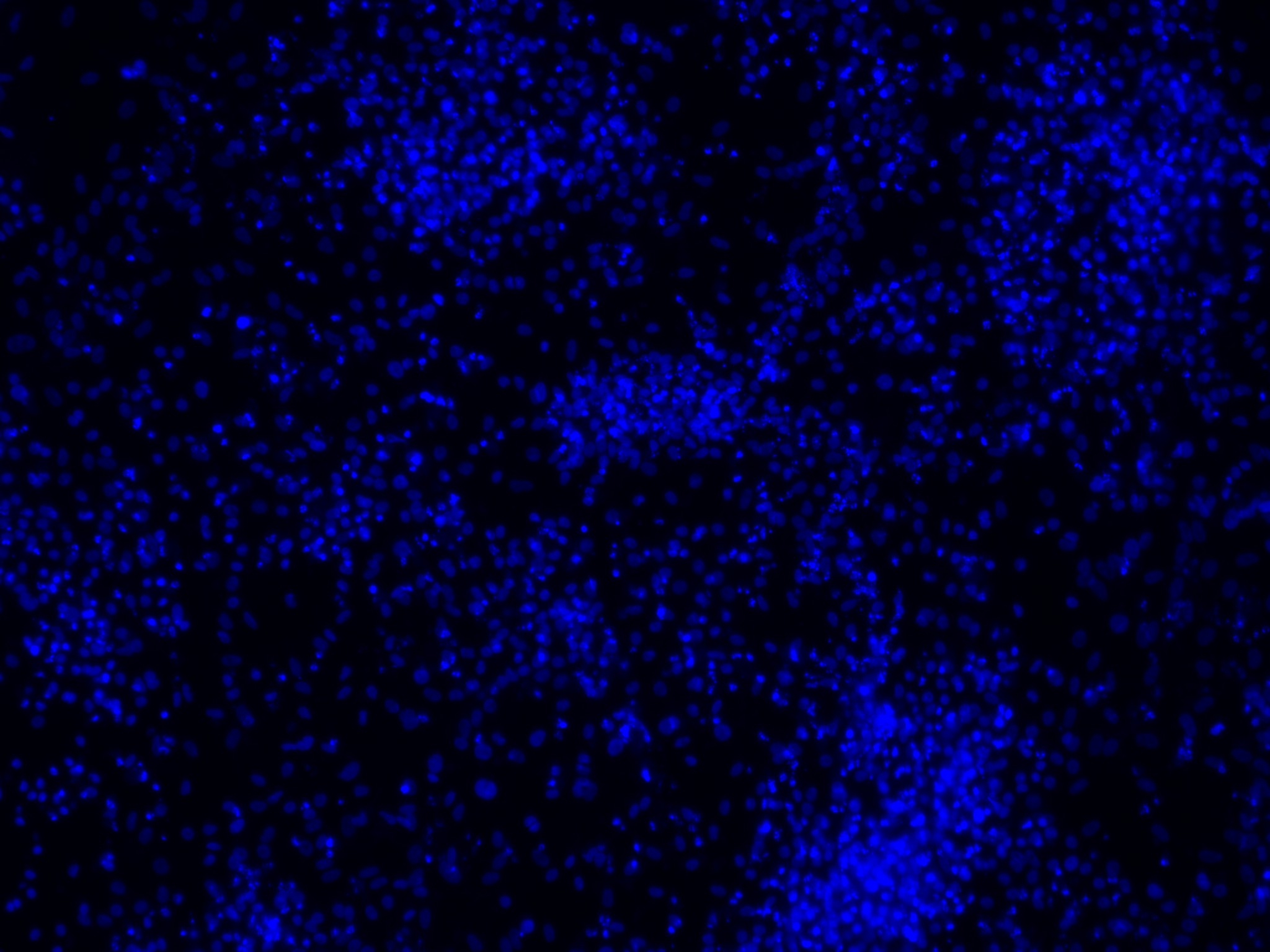

Supplement: Supplementary file 7 — Source data Fig. 6 [file 44318_2025_409_MOESM7_ESM.zip › EMBOJ-2024-118939R-Figure_6_Source_Data-sd/EMBOJ-2024-118939_Fig6H/HAND1-neg_DAPI.jpg]

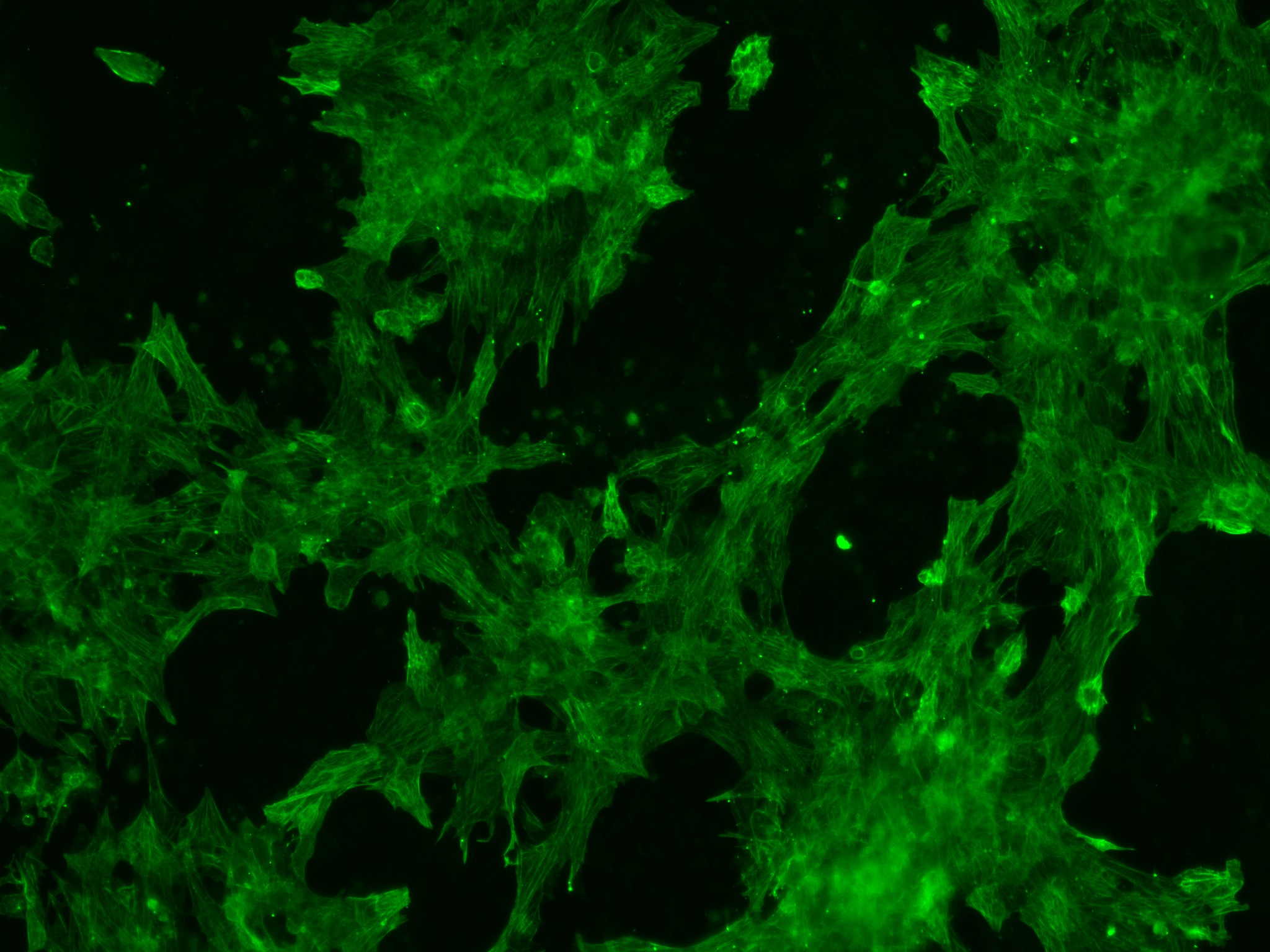

Supplement: Supplementary file 7 — Source data Fig. 6 [file 44318_2025_409_MOESM7_ESM.zip › EMBOJ-2024-118939R-Figure_6_Source_Data-sd/EMBOJ-2024-118939_Fig6H/HAND1-neg_ACNT2.jpg]

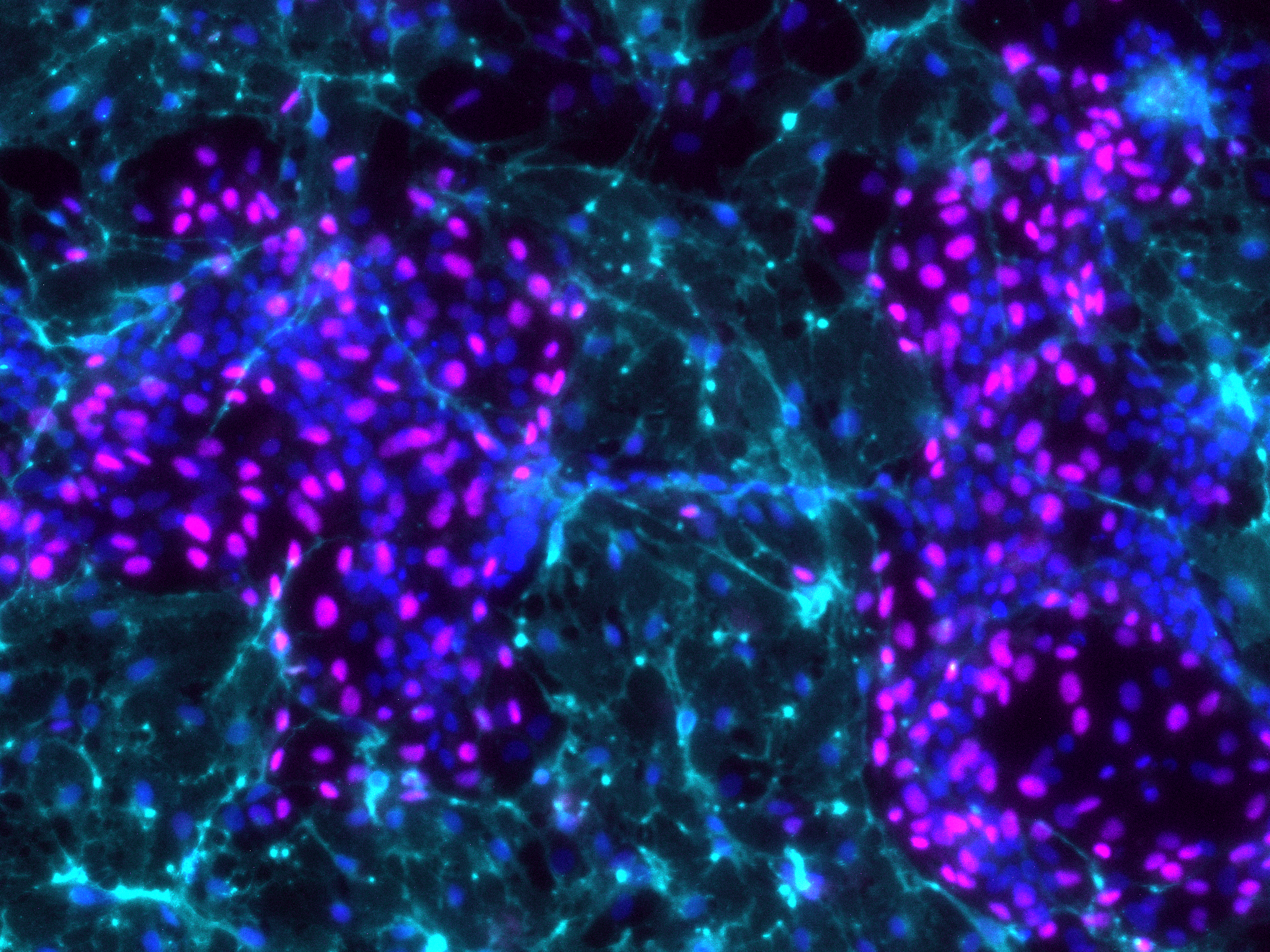

Supplement: Supplementary file 7 — Source data Fig. 6 [file 44318_2025_409_MOESM7_ESM.zip › EMBOJ-2024-118939R-Figure_6_Source_Data-sd/EMBOJ-2024-118939_Fig6G/HAND1-low_overlay.tif]

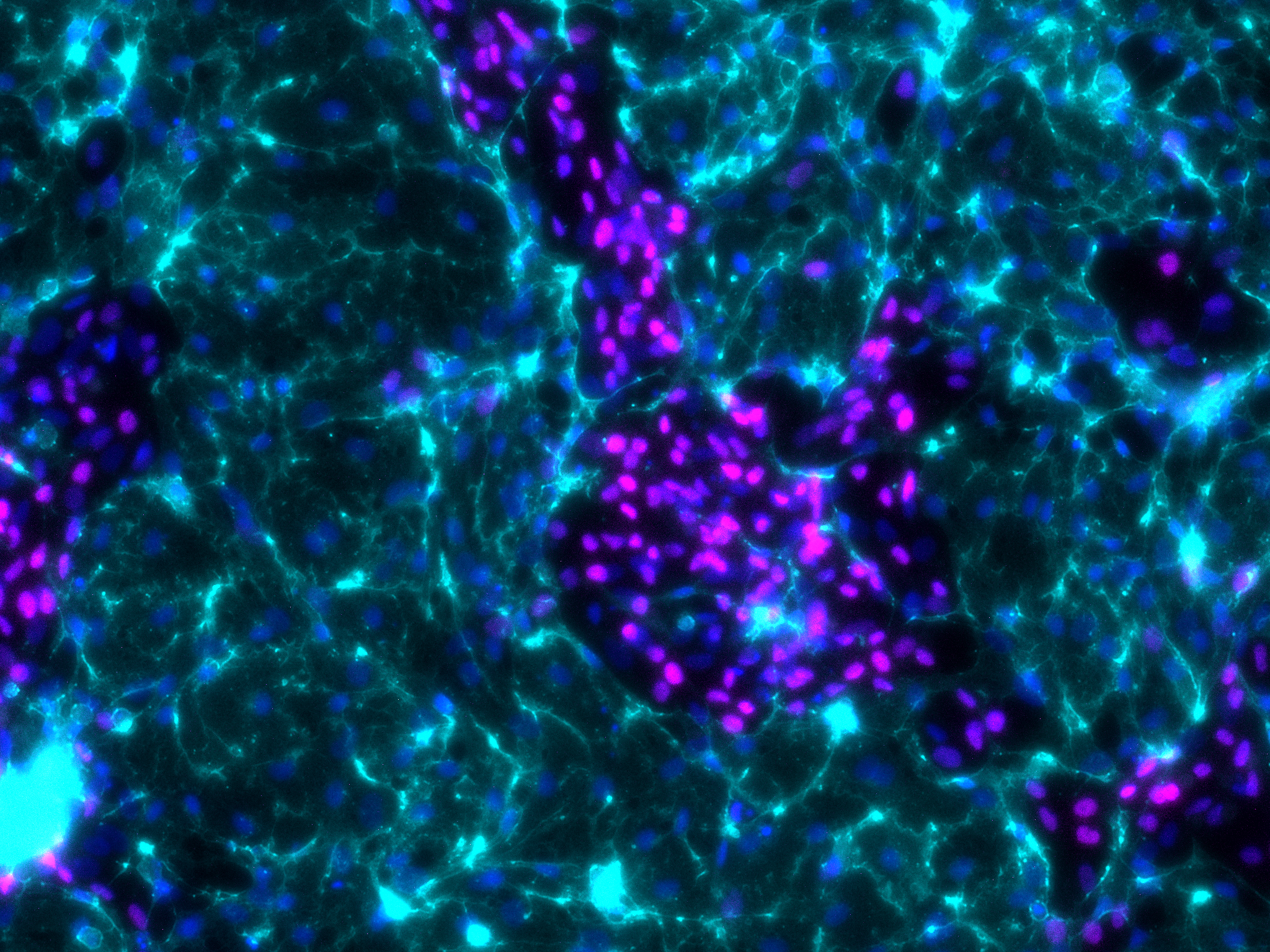

Supplement: Supplementary file 7 — Source data Fig. 6 [file 44318_2025_409_MOESM7_ESM.zip › EMBOJ-2024-118939R-Figure_6_Source_Data-sd/EMBOJ-2024-118939_Fig6G/HAND1-high_overlay.tif]

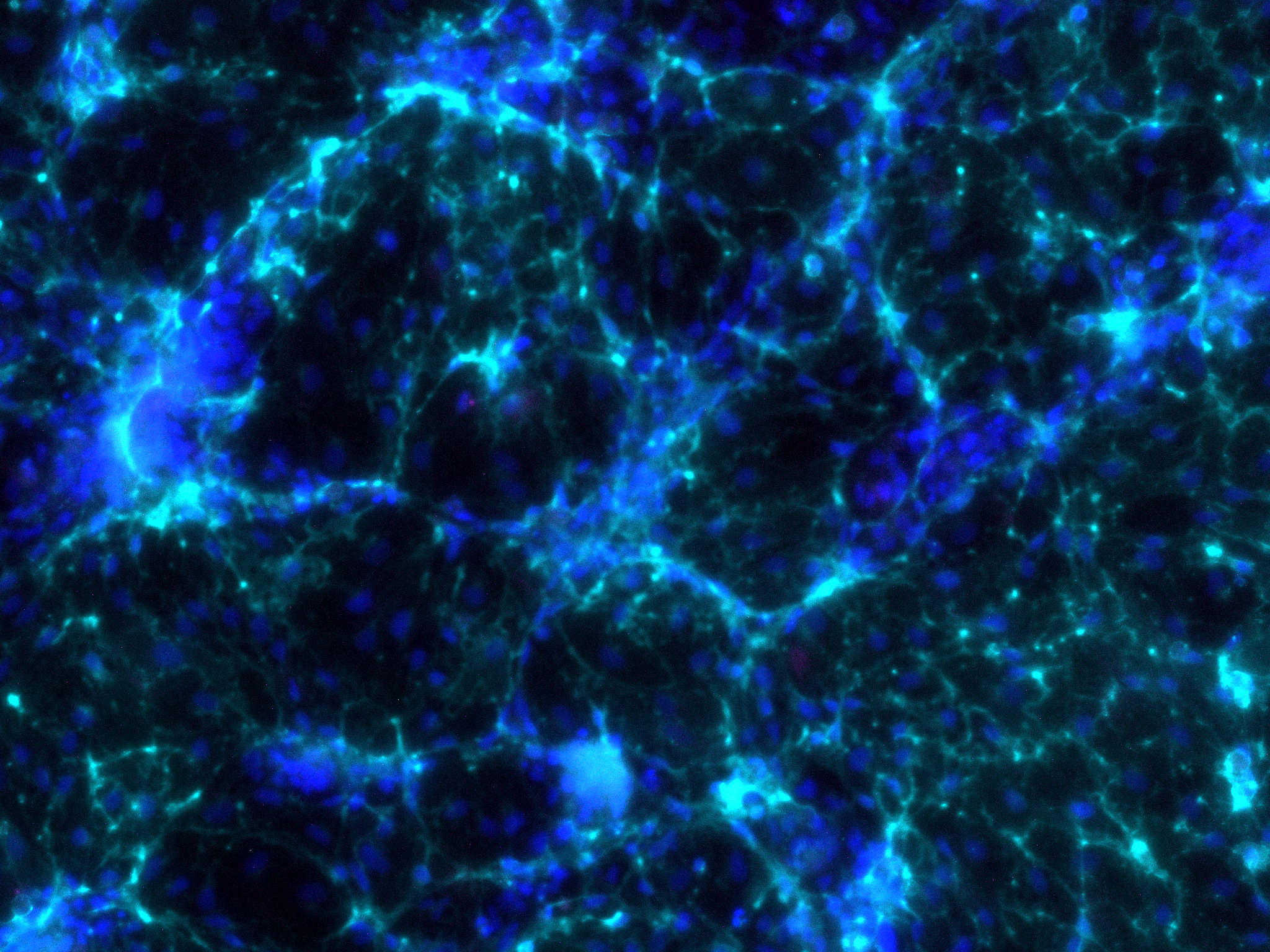

Supplement: Supplementary file 7 — Source data Fig. 6 [file 44318_2025_409_MOESM7_ESM.zip › EMBOJ-2024-118939R-Figure_6_Source_Data-sd/EMBOJ-2024-118939_Fig6G/HAND1-neg_overlay.tif]

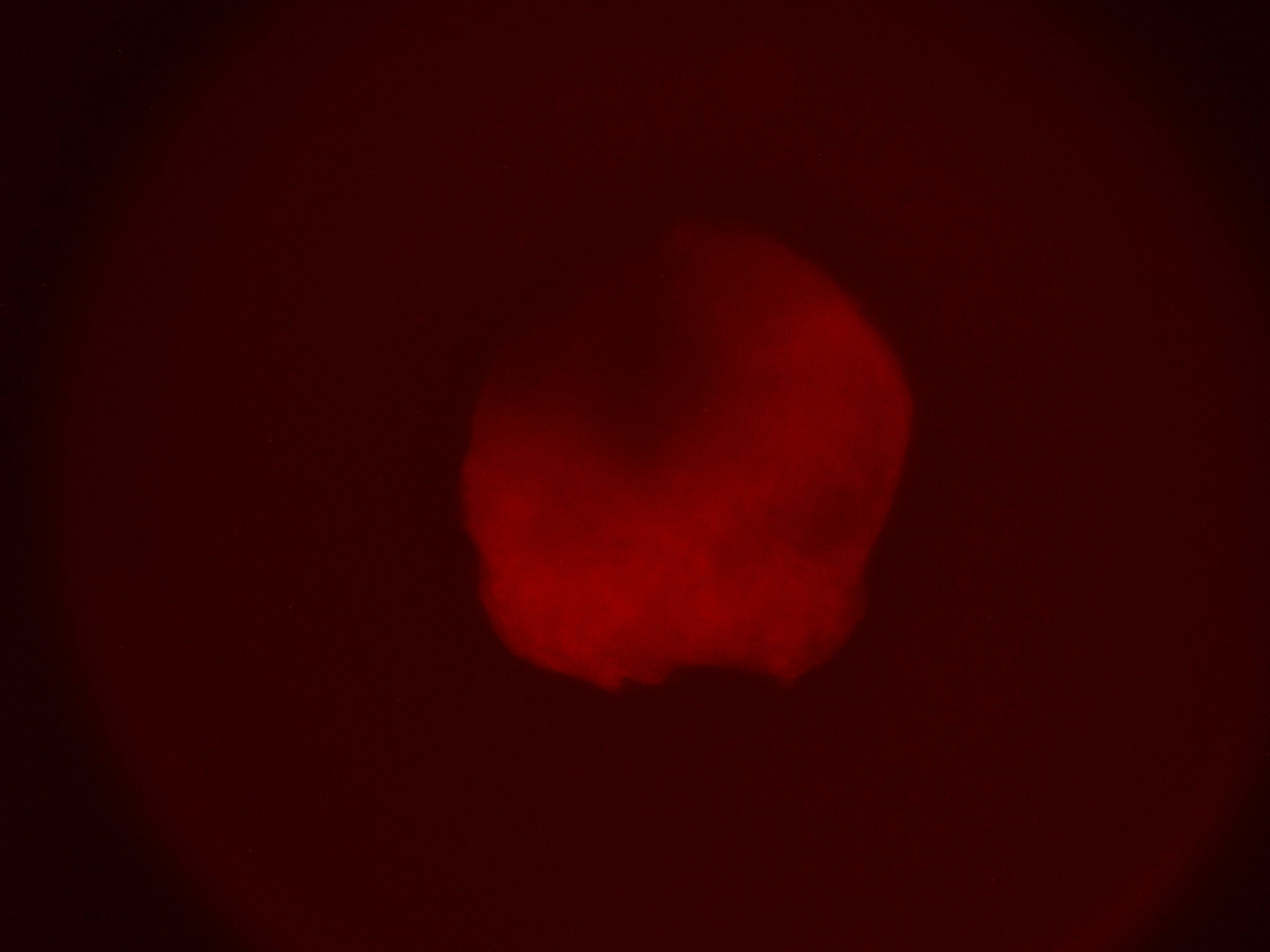

Supplement: Supplementary file 7 — Source data Fig. 6 [file 44318_2025_409_MOESM7_ESM.zip › EMBOJ-2024-118939R-Figure_6_Source_Data-sd/EMBOJ-2024-118939_Fig6B/Veh_Tom.tif]

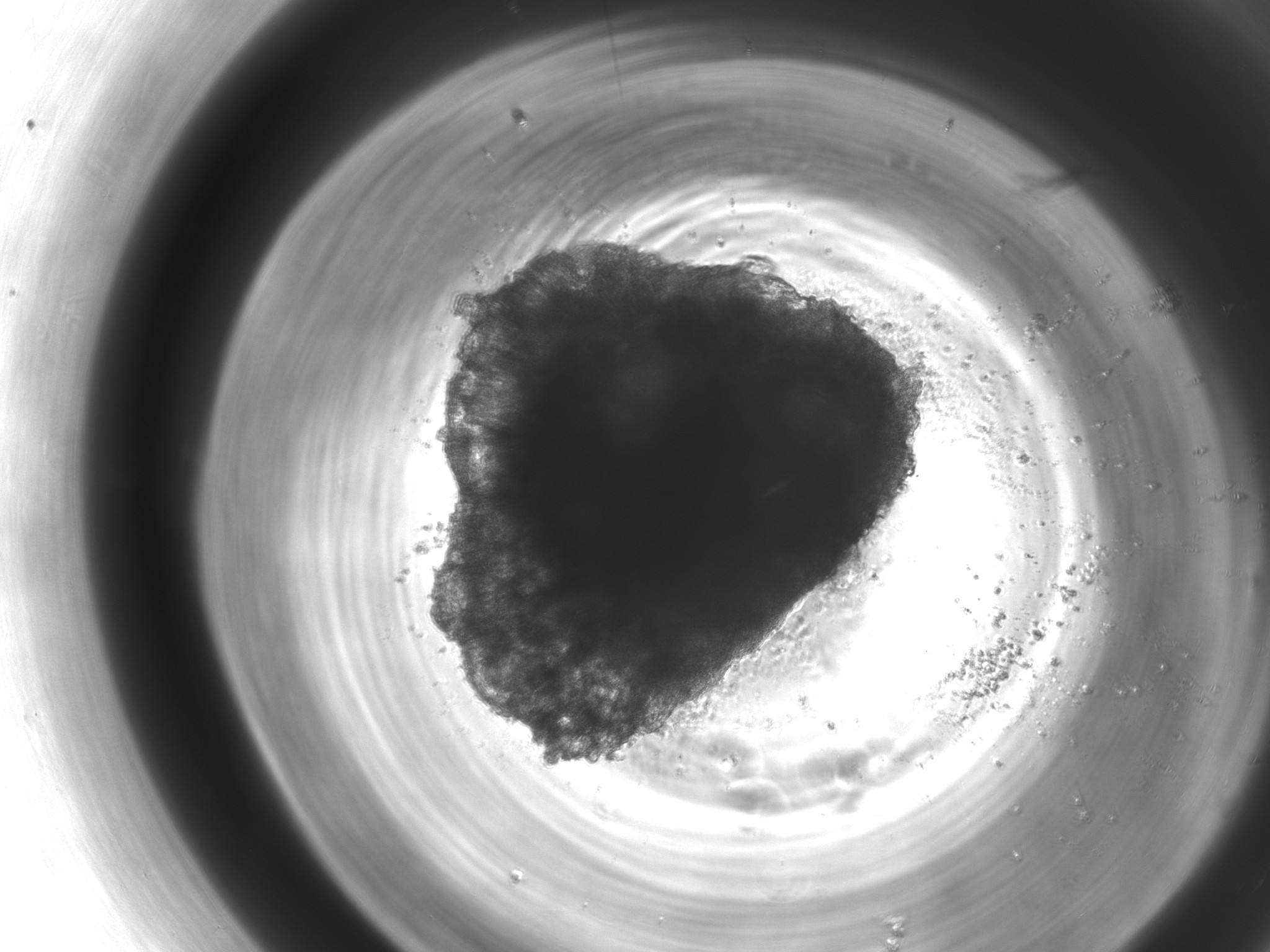

Supplement: Supplementary file 7 — Source data Fig. 6 [file 44318_2025_409_MOESM7_ESM.zip › EMBOJ-2024-118939R-Figure_6_Source_Data-sd/EMBOJ-2024-118939_Fig6B/SB_BF.tif]

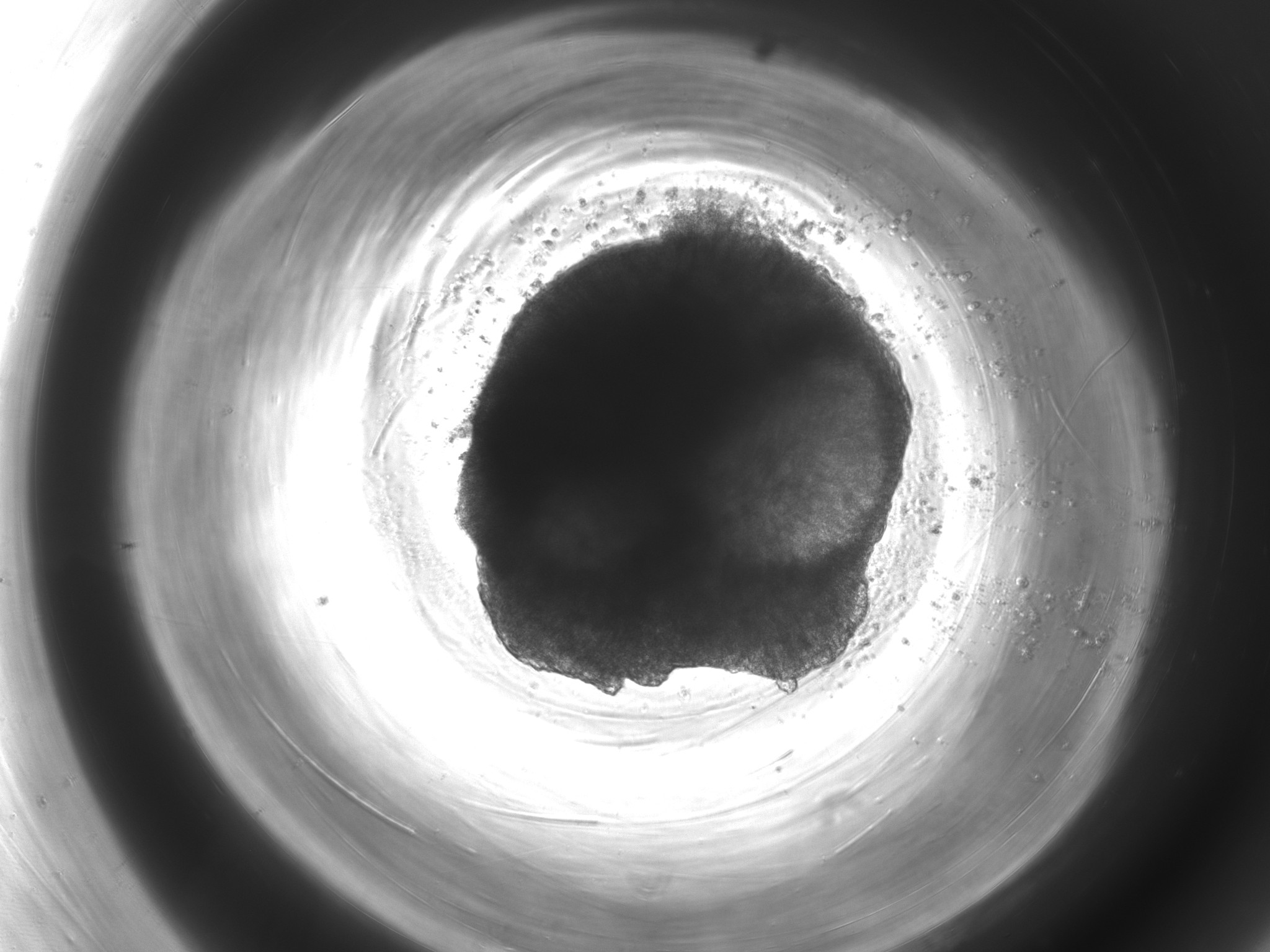

Supplement: Supplementary file 7 — Source data Fig. 6 [file 44318_2025_409_MOESM7_ESM.zip › EMBOJ-2024-118939R-Figure_6_Source_Data-sd/EMBOJ-2024-118939_Fig6B/Veh_BF.tif]

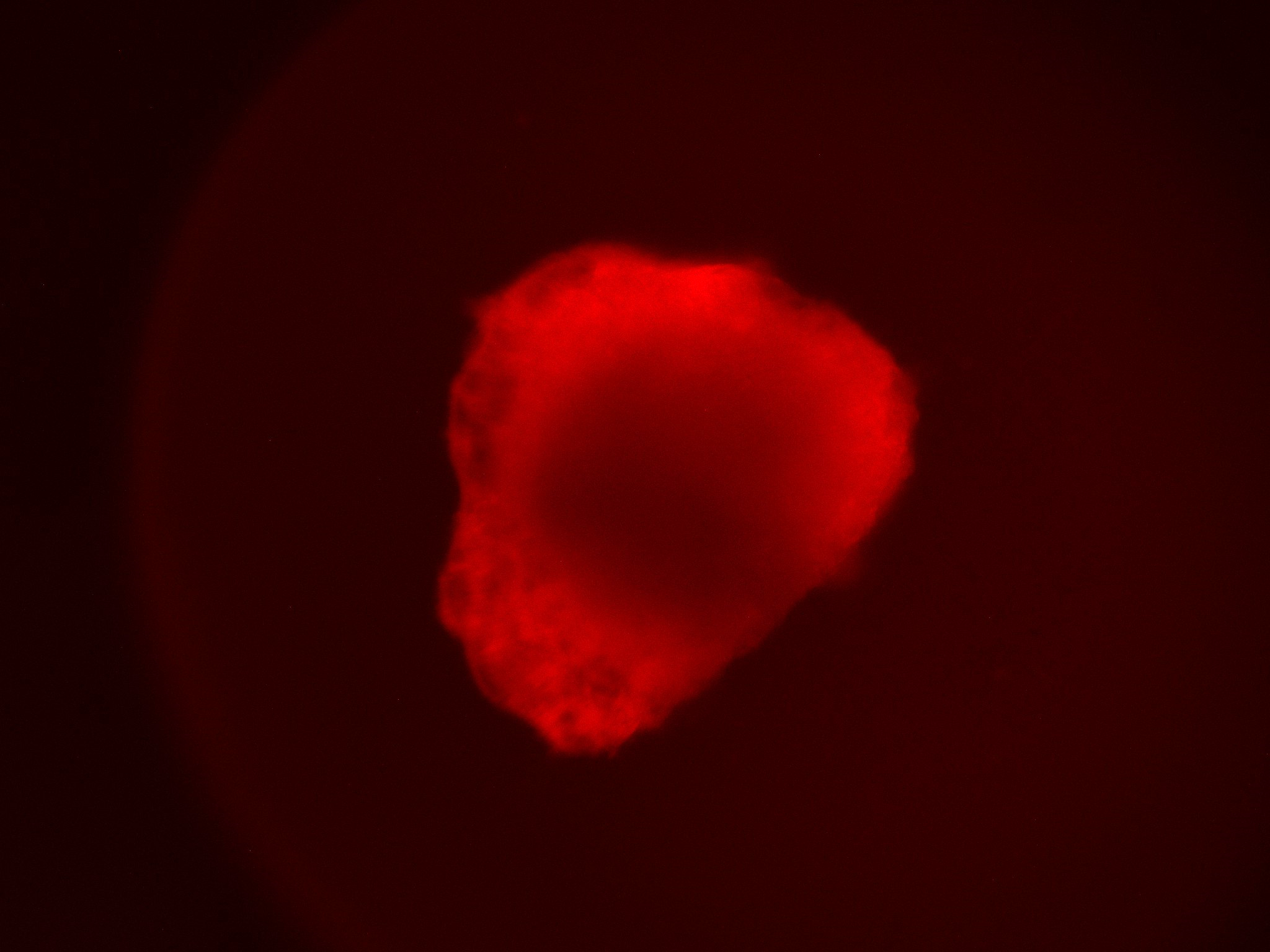

Supplement: Supplementary file 7 — Source data Fig. 6 [file 44318_2025_409_MOESM7_ESM.zip › EMBOJ-2024-118939R-Figure_6_Source_Data-sd/EMBOJ-2024-118939_Fig6B/SB_Tom.tif]

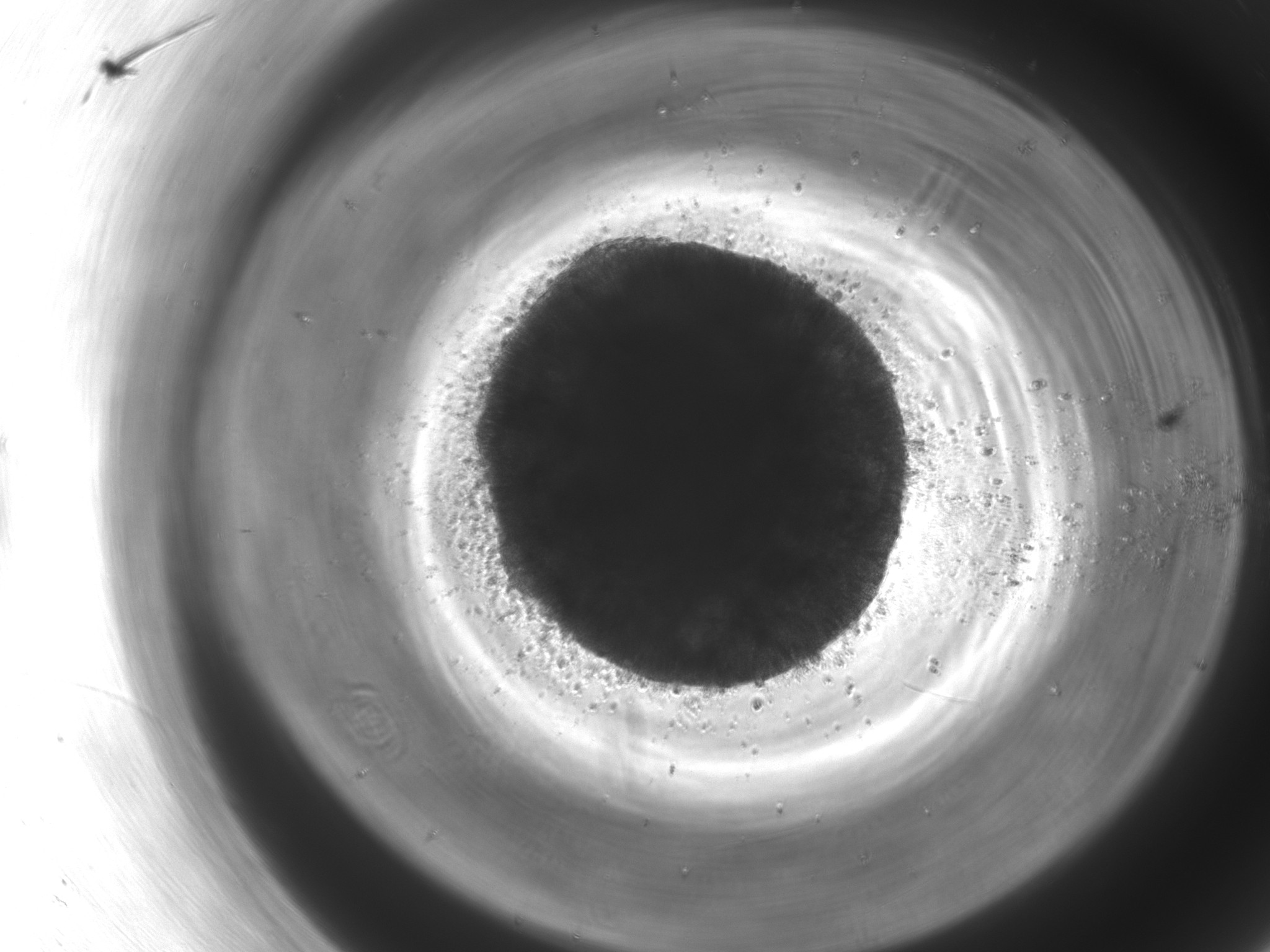

Supplement: Supplementary file 7 — Source data Fig. 6 [file 44318_2025_409_MOESM7_ESM.zip › EMBOJ-2024-118939R-Figure_6_Source_Data-sd/EMBOJ-2024-118939_Fig6B/DMH1_BF.tif]

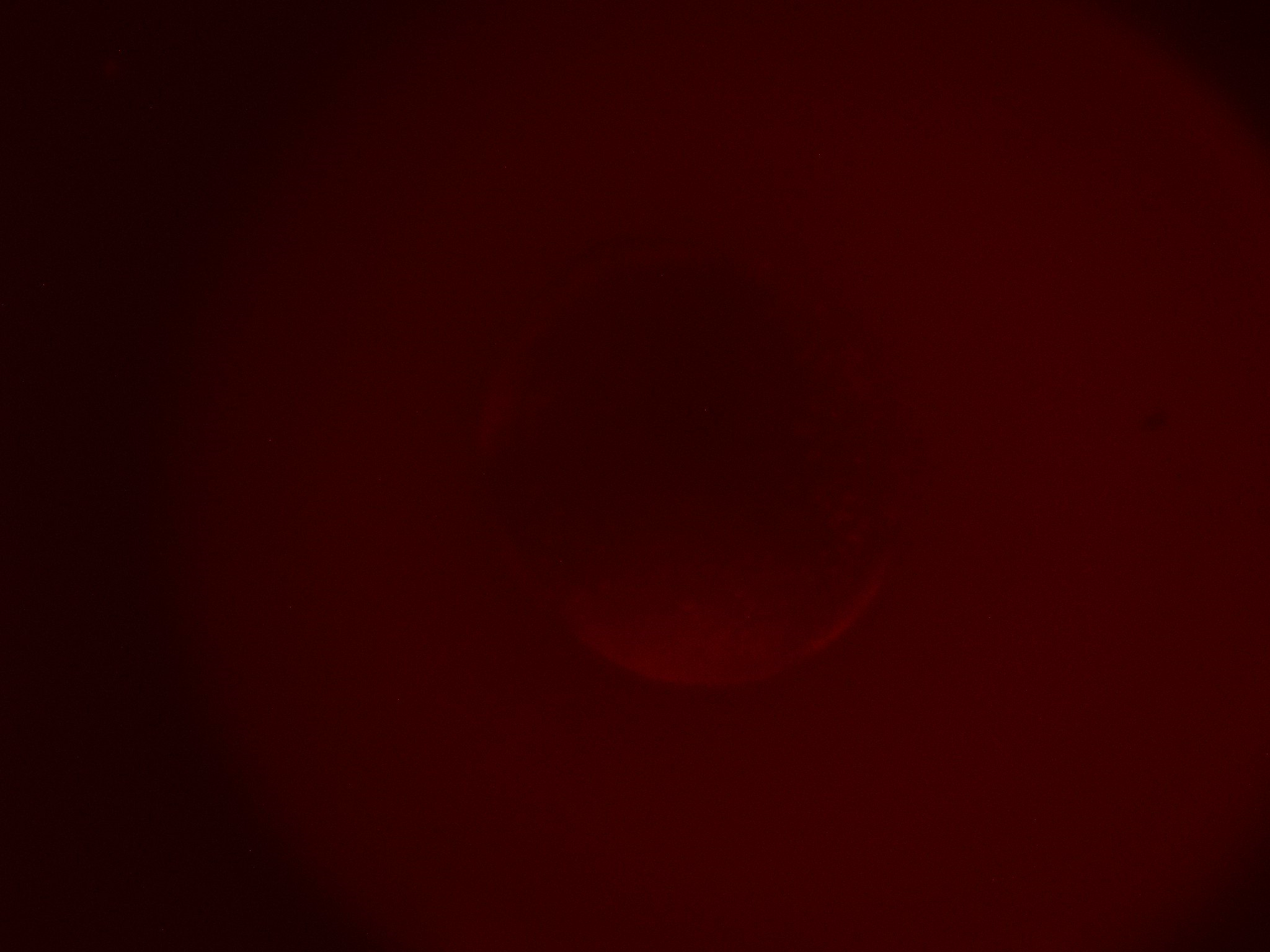

Supplement: Supplementary file 7 — Source data Fig. 6 [file 44318_2025_409_MOESM7_ESM.zip › EMBOJ-2024-118939R-Figure_6_Source_Data-sd/EMBOJ-2024-118939_Fig6B/DMH1_Tom.tif]
