# Supplementary material for: HAND1 level controls the specification of multipotent cardiac and extraembryonic progenitors from human pluripotent stem cells
Source: EMBO J. 2025 Mar 31;44(9):2541–65. doi: 10.1038/s44318-025-00409-0 (PMC12048643; doi:10.1038/s44318-025-00409-0)
Supplement: Supplementary file 8 — Expanded View Figures [file 44318_2025_409_MOESM8_ESM.pdf]

Expanded View Figures

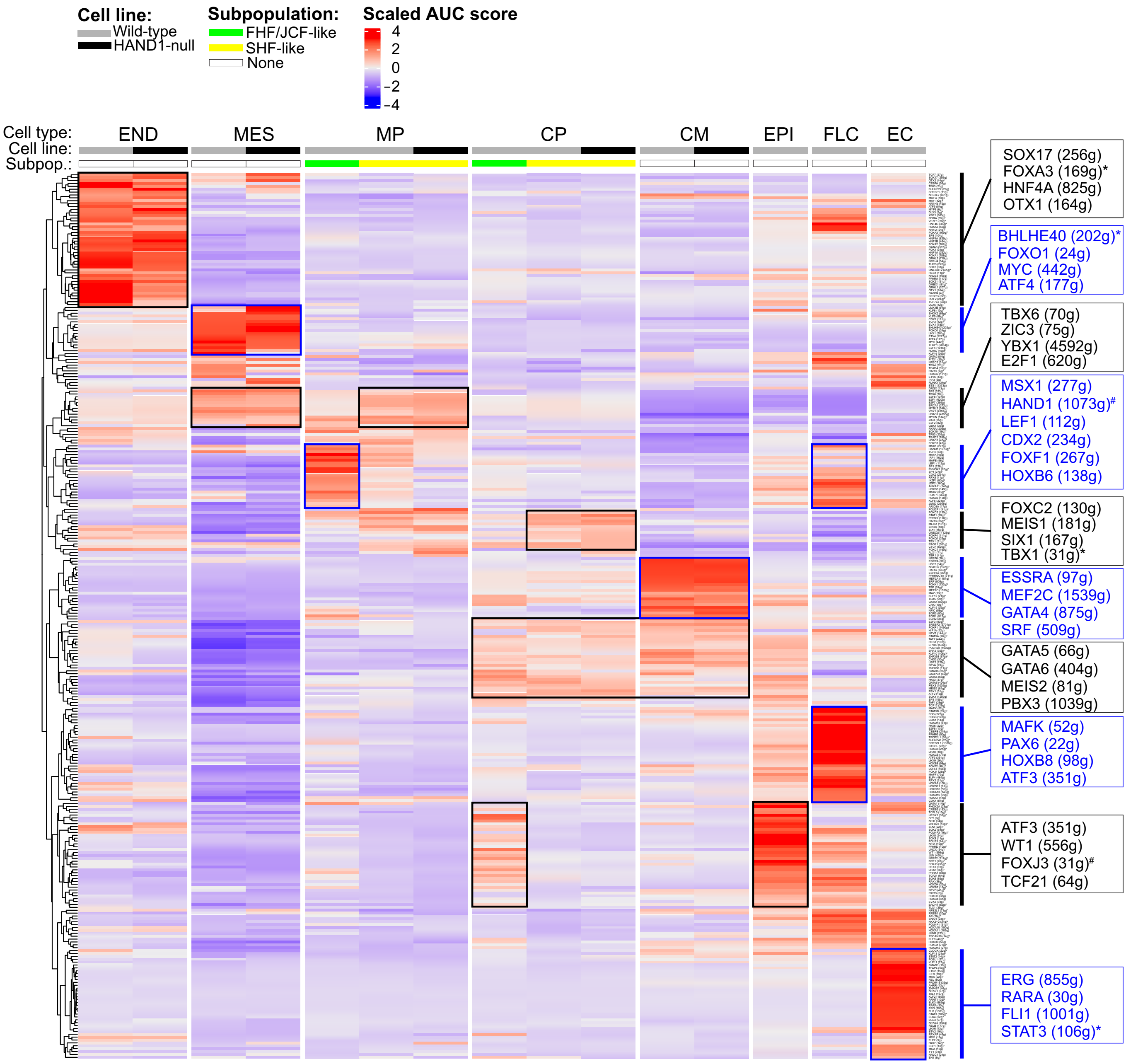

Figure EV1. Regulatory network analysis during differentiation.

Heatmap and hierarchical clustering showing the scaled target gene AUC score of the 307 regulons detected by SCENIC by cell line, cell type, and subpopulation. Clustering is by rows (regulons). AUC values were converted to a z-score and centred on the mean. Select markers present in clusters are highlighted. *Identified in HAND1-null population. #Identified in CPC lineage analysis. AUC area under the curve, CPC cardiac progenitor cell, CM cardiomyocyte, EC endothelial cells, END endoderm, EPI epicardial, FHF first heart field, FLC fibroblast-like cells, JCF juxta-cardiac field, MES mesoderm, MP mesodermal progenitors, SCENIC single-cell regulatory network interference and clustering, SHF second heart field.

 © The Author(s)

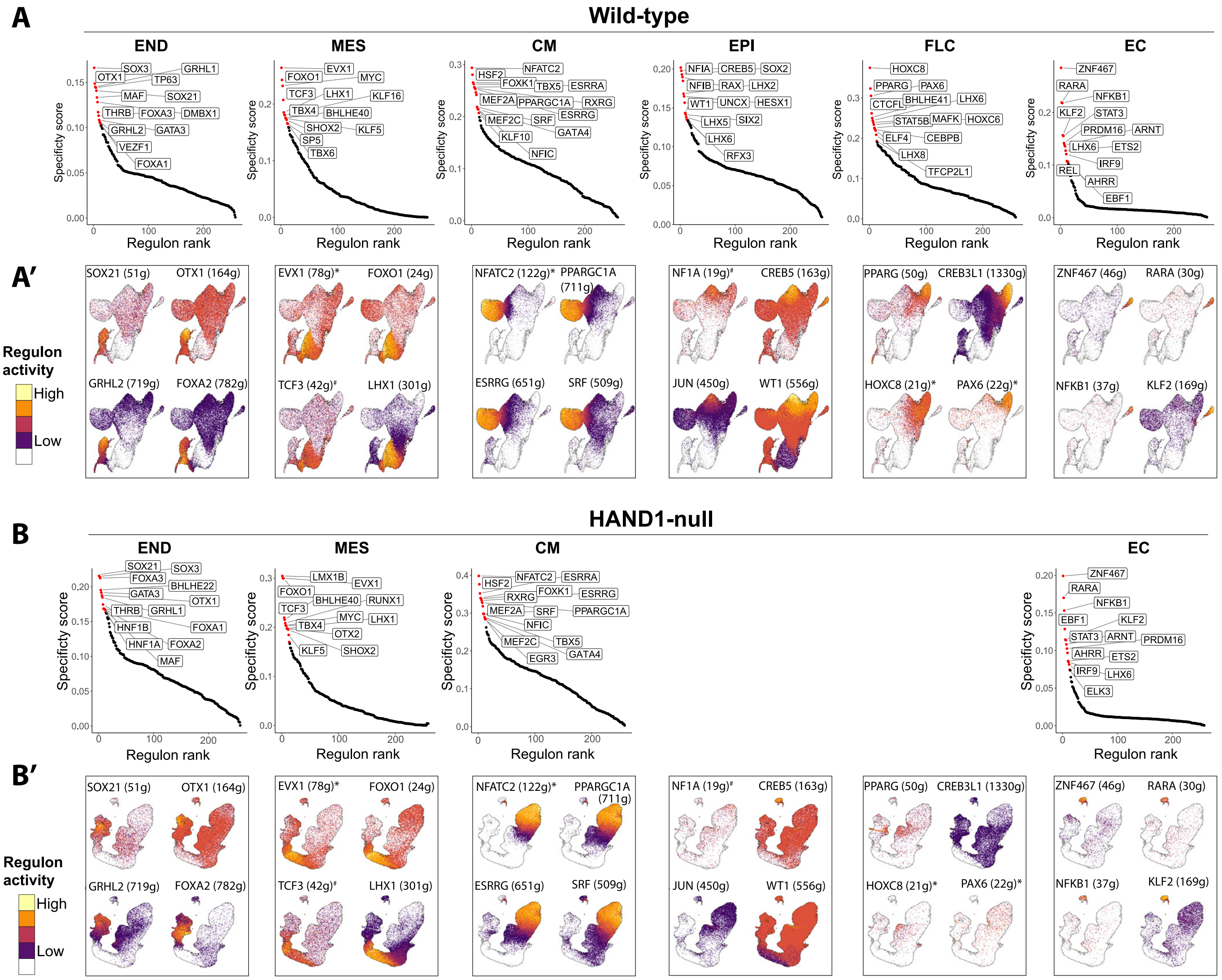

Figure EV2. SCENIC regulon specificity ranking in different populations during differentiation.

(**A**) Regulon specificity scores ranked for different cell types in wild-type and (**B**) in HAND1-null populations. The HAND1-null line did not generate EPI or FLC types. The top 5% are highlighted red and several are labelled. (**A′**) UMAP plots showing the activity of some key cell type-biased regulons in wild-type and in (**B′**) HAND1-null cells. *Identified in HAND1-null population. #Identified in CPC lineage analysis. AUC area under the curve, CM cardiomyocyte, EC endothelial cells, END endoderm, EPI epicardial, MES mesoderm, FLC fibroblast-like cells, SCENIC single-cell regulatory network interference and clustering.

© The Author(s)
